# Author Correction: ATR inhibition augments the efficacy of lurbinectedin in small-cell lung cancer

**DOI:** 10.1038/s44321-024-00029-x

**Published:** 2024-02-16

**Authors:** Christopher W Schultz, Yang Zhang, Rajaa Elmeskini, Astrid Zimmermann, Haiqing Fu, Yasuhisa Murai, Darawalee Wangsa, Suresh Kumar, Nobuyuki Takahashi, Devon Atkinson, Liton Kumar Saha, Chien-Fei Lee, Brian Elenbaas, Parth Desai, Robin Sebastian, Ajit Kumar Sharma, Melissa Abel, Brett Schroeder, Manan Krishnamurthy, Laurel L Bassel, Rajesh Kumar, Nitin Roper, Mirit Aladjem, Frank T Zenke, Zoe Weaver Ohler, Yves Pommier, Anish Thomas

**Affiliations:** 1 Developmental Therapeutics Branch, Center for Cancer Research, National Cancer Institute, National Institutes of Health, Bethesda, MD 20892 USA; 2 Center for Advanced Preclinical Research, Leidos Biomedical Research, Inc, Frederick National Laboratory for Cancer Research, Frederick, MD 21702 USA; 3 Merck KGaA, Biopharma R&D, Translational Innovation Platform Oncology, Frankfurter Street 250, 64293 Darmstadt, Germany; 4 Genetics Branch, Center for Cancer Research, National Cancer Institute, National Institutes of Health, Bethesda, MD 20892 USA; 5 Medical Oncology Branch, National Center for Global Health and Medicine, Tokyo, 162-8655 Japan; 6 EMD Serono Research and Development Institute Inc., Biopharma R&D, Translational Innovation Platform Oncology, Billerica, MA 01821 USA

## Abstract

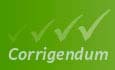

C**orrection to:** *EMBO Molecular Medicine* (2023) 15:e17313. 10.15252/emmm.202217313 | Published online 25 July 2023

By mutual agreement of all authors and by the evidence that the additional author meets the appropriate authorship criteria, Laura L Bassel and their contribution and authorship position will be added to this manuscript.

The authorship will be corrected from:

Christopher W Schultz^1^, Yang Zhang^1^, Rajaa Elmeskini^2^, Astrid Zimmermann^3^, Haiqing Fu^1^, Yasuhisa Murai^1^, Darawalee Wangsa^4^, Suresh Kumar^1^, Nobuyuki Takahashi^1,5^, Devon Atkinson^2^, Liton Kumar Saha^1^, Chien-Fei Lee^6^, Brian Elenbaas^6^, Parth Desai^1^, Robin Sebastian^1^, Ajit Kumar Sharma^1^, Melissa Abel^1^, Brett Schroeder^1^, Manan Krishnamurthy^1^, Rajesh Kumar^1^, Nitin Roper^1^, Mirit Aladjem^1^, Frank T Zenke^3^, Zoe Weaver Ohler^2^, Yves Pommier^1^, Anish Thomas^1^

To:

Christopher W Schultz^1^, Yang Zhang^1^, Rajaa Elmeskini^2^, Astrid Zimmermann^3^, Haiqing Fu^1^, Yasuhisa Murai^1^, Darawalee Wangsa^4^, Suresh Kumar^1^, Nobuyuki Takahashi^1,5^, Devon Atkinson^2^, Liton Kumar Saha^1^, Chien-Fei Lee^6^, Brian Elenbaas^6^, Parth Desai^1^, Robin Sebastian^1^, Ajit Kumar Sharma^1^, Melissa Abel^1^, Brett Schroeder^1^, Manan Krishnamurthy^1^, Laurel L Bassel^2^, Rajesh Kumar^1^, Nitin Roper^1^, Mirit Aladjem^1^, Frank T Zenke^3^, Zoe Weaver Ohler^2^, Yves Pommier^1^, Anish Thomas^1^

Author Statement:

Laura L Bassel, pathologist at the Center for Advanced Preclinical Research, Leidos Biomedical Research, Inc, Frederick National Laboratory for Cancer Research, Frederick, MD, USA, contributed to the above cited paper by quantifying the IHC stain and necrosis in (panel E in emmm202217313-fig-0005ev): Reference figure: emmm202217313-fig-0005ev-m.jpg-EMBO Molecular Medecine 2023.jpg.

