# Supplementary material for: ATR inhibition augments the efficacy of lurbinectedin in small‐cell lung cancer
Source: EMBO Mol Med. 2023 Jul 25;15(8):e17313. doi: 10.15252/emmm.202217313 (PMC10405061; doi:10.15252/emmm.202217313)
Supplement: Supplementary file 1 — Appendix [file EMMM-15-e17313-s010.pdf]

ATR inhibition augments the efficacy of lurbinectedin in small cell lung cancer

Christopher W. Schultz *et al* 2023

APPENDIX

Table of Contents:

Appendix Figure S1 Legend: 2

Appendix Figure S1: 3

Appendix Figure S2 Legend: 4

Appendix Figure S2: 5

Appendix Fig. S1:

A-C) In two SCLC cell lines lurbinectedin treatment increased γH2AX induction which was reduced with berzosertib co-treatment. γH2AX induction was assessed using immunofluorescence in DMS 114 (A) and NCI-H446 (B) cells treated +/- 1 nM lurbinectedin +/- 2μM berzosertib for 6 hours. Quantification for NCI-H446 is displayed in (C) and quantification for DMS 114 is in Figure 2C. Quantification is from 100-150 cells per treatment, with error bars indicating SEM, comparisons were made using an unpaired two-tailed student's t test in PRISM, biological replicates = 3.

D) Lurbinectedin treatment caused an increase in γH2AX levels in 2/4 cell lines, while berzosertib treatment either alone or in combination with lurbinectedin causes a decrease in γH2AX. Cell lines were treated +/- 1 nM lurbinectedin +/- 2μM berzosertib for 6 hours. Quantification is indicative of γH2AX normalized to H3 levels vs control levels for each cell line. The green cell lines here were later determined to have greater synergy, while the red cell lines indicate cell lines later determined to be less synergistic.

E) Berzosertib caused an abnormal division phenotype, while only the combination of lurbinectedin and berzosertib caused a significant number of chromosomal breaks. DMS 114 cells were treated +/- 1 nM lurbinectedin +/- 2 μM berzosertib for 6 hours followed by metaphase spread analysis assessing chromosome breaks and abnormal metaphase spread. Data are indicative of three separate experiments with 150-200 metaphases assessed per experiment, error bars = SD, p values indicate unpaired two-tailed student's t tests in PRISM.

F) Representative images of normal (left) and abnormal chromosomal spread (right) as caused by berzosertib in representative images from Fig. S2D.

G) Barasertib re-capitulated the abnormal division phenotype caused by berzosertib in DMS 114 cells. DMS 114 cells were treated with 300nM barasertib for 6 hours with the final two 2 hours of treatment also including nocodazole. Cell chromosomal structure and integrity was then assessed using mitotic spread.

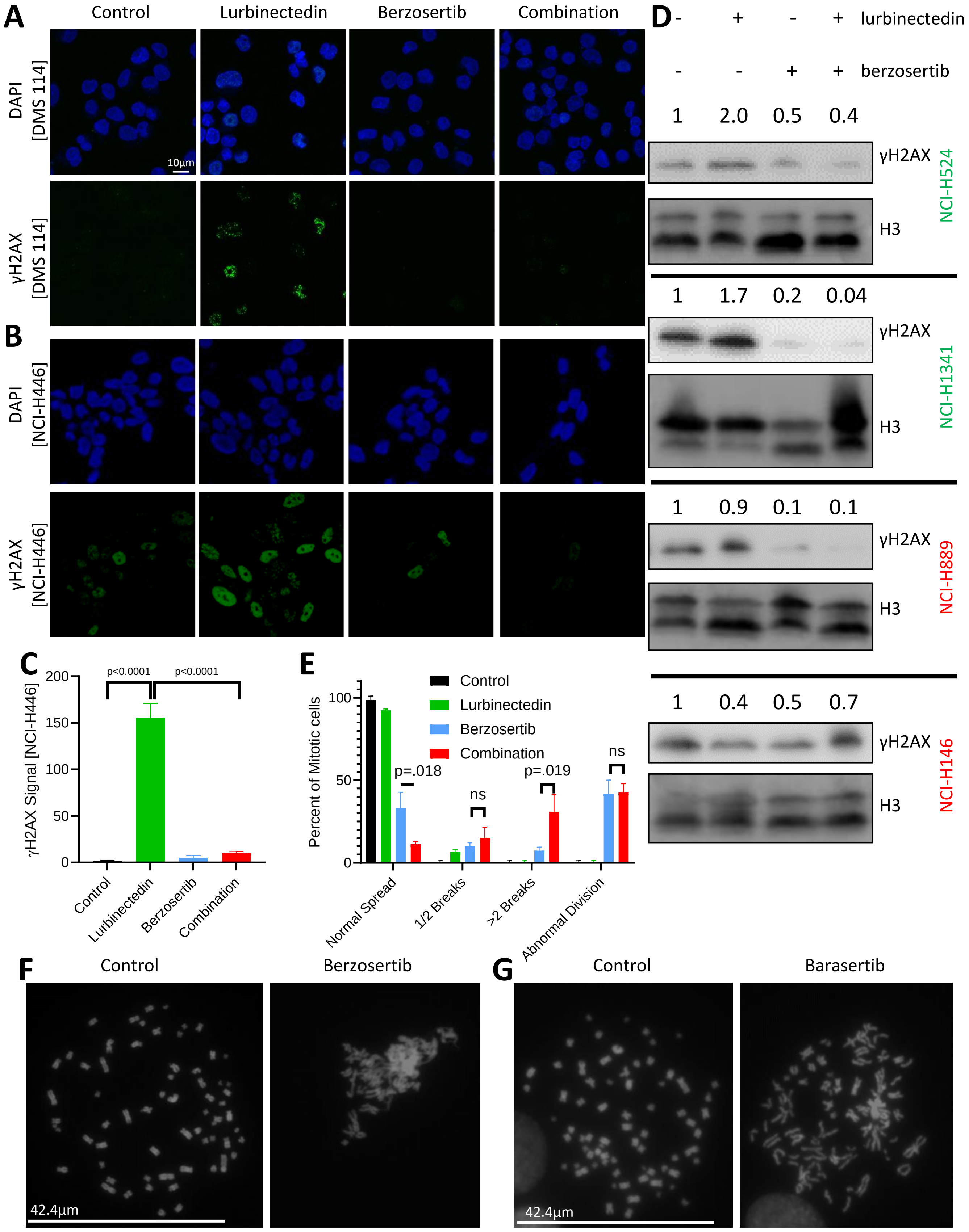

A
Control
Lurbinectedin
Berzosertib
Combination
DAPI [DMS 114]
γH2AX [DMS 114]
10µm
B
DAPI [NCI-H446]
γH2AX [NCI-H446]
C
γH2AX Signal [NCI-H446]
p<0.0001
p<0.0001
200
150
100
50
0
Control
Lurbinectedin
Berzosertib
Combination
D
- + - + lurbinectedin
- - + + berzosertib
1 2.0 0.5 0.4
γH2AX
H3
NCI-H524
1 1.7 0.2 0.04
γH2AX
H3
NCI-H1341
1 0.9 0.1 0.1
γH2AX
H3
NCI-H889
1 0.4 0.5 0.7
γH2AX
H3
NCI-H146
E
Percent of Mitotic cells
Control
Lurbinectedin
Berzosertib
Combination
p=.018
ns
p=.019
ns
100
50
0
Normal Spread
1/2 Breaks
>2 Breaks
Abnormal Division
F
Control
Berzosertib
42.4µm
G
Control
Barasertib
42.4µm

Appendix Fig. S2:

A) *ASCL1* expression positively correlated with NE score in patient tumor samples (positive control) while *MYC* expression negatively correlated with NE score in the same dataset. In the NCI dataset of 100 SCLC patients NE score was analyzed using SSGSEA. Pearson correlation was assessed in PRISM.

B) Similar to figure 3H, high MYC family member expression negatively associated with *CDKN1A* (p21) expression in a CDX SCLC datasets, and trended towards negative association in a smaller CDX dataset indicating that Non-NE differentiation may reduce *CDKN1A* (p21) expression. *MYC*, *MYCL* and *MYCN* expression were z scored (within each database) and the max MYC family member z score expression was determined for each sample. Those samples which were greater than one standard deviation above average were considered to be high MYC family member expressing. Comparisons between groups were made using unpaired two-tailed student's t tests in PRISM.

C,D) *MYC* expression was assessed against *CDKN1A* and NE differentiation score in a panel of 67 SCLC cell lines and they were found to be inversely correlated. Pearson correlation was assessed in PRISM.

E) Out of 50 hallmark pathway genesets, MYC Targets V1 was the most predictive of resistance to first line platinum-based chemotherapy followed by DNA repair and MYC Targets V2. We assessed enrichment of Hallmark pathways in 100 SCLC patient samples using SSGSEA, platinum resistance was defined as a response to carboplatin etoposide treatment of <90 days.

F,G) MYC high patients were more likely to be platinum resistant. 100 patient samples/records from the NCI SCLC dataset were assessed. Platinum sensitivity was defined as recurrence or relapse < 90 days after completion of first line platinum-based chemotherapy. Patients were stratified based on median (F) and quartile (G) MYC targets V1 enrichment. We utilized Fisher's exact t tests to determine significance for these samples in PRISM. Hazard ratios were calculated using odd's ratio in PRISM for F and G were 0.2130 and 0.1830 for low MYC vs high MYC respectively indicating a ~5 fold decreased risk for resistance to platinum based therapy in low MYC patients.

H) In our panel of 9 cell lines the combination efficacy (IC_{50} of lurbinectedin + 500 nM BRZ) negatively correlated with MYC Targets V2 enrichment. MYC Targets V2 was calculated using SSGSEA, Pearson correlation was assessed in PRISM.

I,J) In both the NCI dataset of SCLC 100 (I) and the George patient Dataset (J) NE score negatively correlated with ERCC5 expression. Pearson correlation was assessed in PRISM.

K) Both lurbinectedin and combination treatment were toxic in a large proportion of mice. Mouse body weights corresponding to figure 4A, mice which required sacrifice due to toxicity (body weight loss or other) are quantified in the top right in red.

L) We compared variables assessed with RNA-sequencing data of tumor models PDX-06 vs PDX-03 as well as aggressiveness as approximated by the time to reach $1000mm^3$ for initially transplanted fresh tumor samples from patients (P0). These data are part of initial screening data for our PDX models generated and represent 1 RNA seq sample from 1 passage 1 tumor from each model. NE score was calculated using SSGSEA.

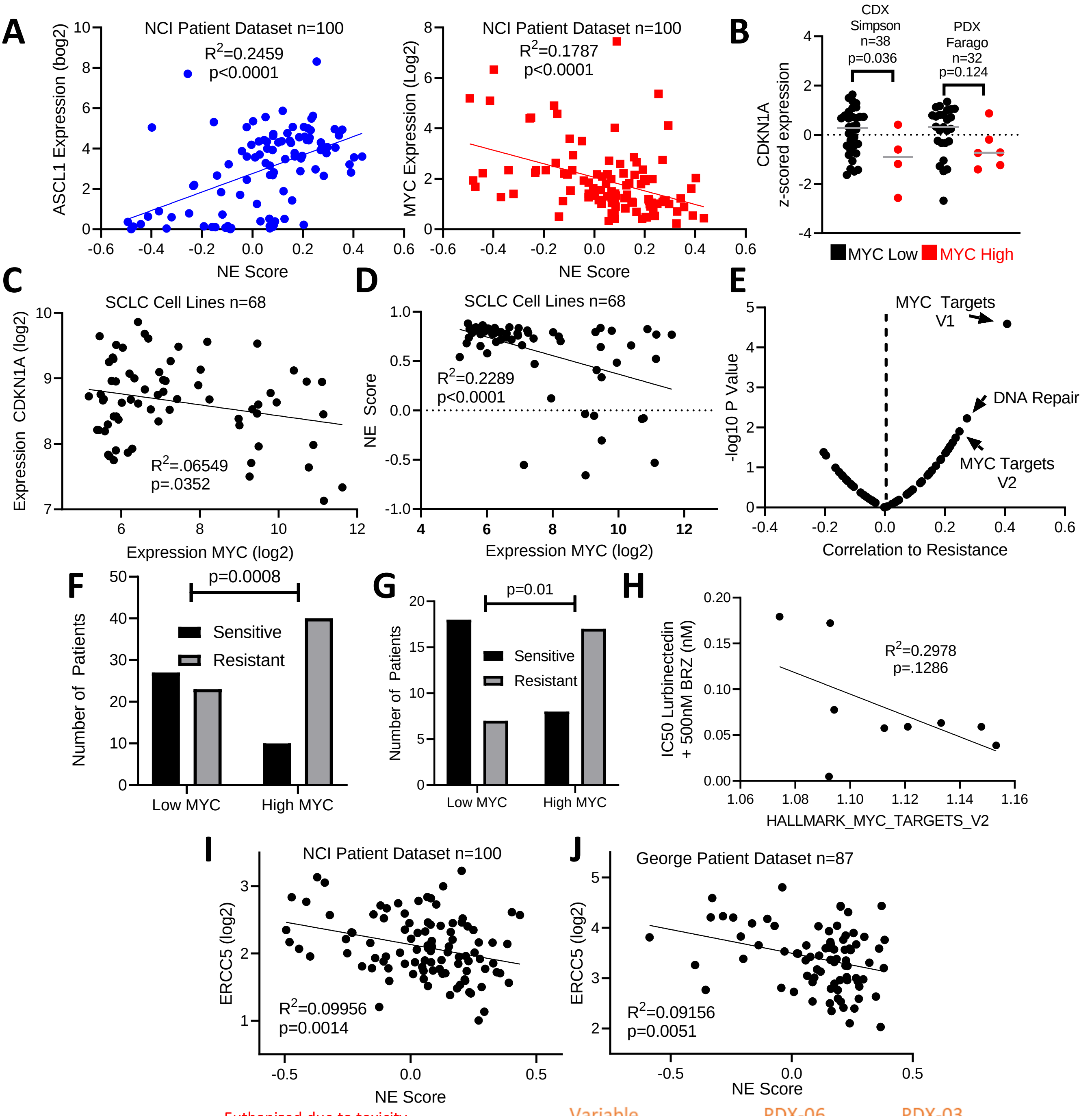

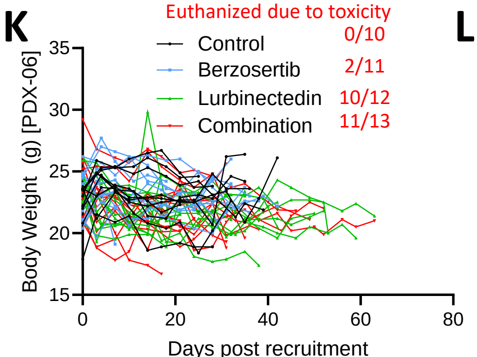

L

Variable	PDX-06	PDX-03
Days to reach 1000mm3 P0	102	65
ASCL1	5.18	0.10
NEUROD1	2.11	0.07
POU2F3	0.17	6.02
YAP1	2.19	2.01
CDKN1A	4.87	3.92
ERCC5	2.35	2.58
SLFN11	3.95	2.06
NE Score	0.264	-0.288
