# Supplementary material for: ATR inhibition augments the efficacy of lurbinectedin in small‐cell lung cancer
Source: EMBO Mol Med. 2023 Jul 25;15(8):e17313. doi: 10.15252/emmm.202217313 (PMC10405061; doi:10.15252/emmm.202217313)
Supplement: Supplementary file 2 — Expanded View Figures PDF [file EMMM-15-e17313-s008.pdf]

Expanded View Figures

Figure EV1. Lurbinectedin causes γH2AX induction and reduced EDU incorporation and ATR inhibition can ameliorate these effects.

A The majority of SCLC cell lines were significantly more sensitive to lurbinectedin as compared to topotecan. IC_{50}s for topotecan and lurbinectedin were assessed in nine SCLC cell lines (biological replicates = 2, technical replicates = 3).

B Lurbinectedin induced the ATR (yellow/gold), ATM (green), and DNA-PK (blue) damage repair pathways as well as inducing γH2AX (DNA damage marker) in all three cell lines. DMS 114, NCI-H146, and NCI-H889 SCLC cell lines were treated with lurbinectedin at various concentrations for 2, 6, and 24 h. DMS 114 cells are lacking 24 h lurbinectedin concentrations of 10 and 100 nM due to excessive cell death under these conditions in the DMS 114 cell line.

C Lurbinectedin caused the greatest increase in γH2AX induction in S-phase cells in NCI-H1341 cells. NCI-H1341 cells (SCLC cell line) were treated with 1 nM lurbinectedin from 1 to 6 h and γH2AX induction was assessed using FACS, four technical replicates of 10,000 cells for each timepoint were assessed; biological replicates = 3. Cell cycle was assessed using propidium iodide.

D–G The average γH2AX signal increased with the addition of berzosertib to lurbinectedin (likely due to a small portion of cells as demonstrated in Fig 2B) while the median decreased indicating the majority of cells displayed decreased γH2AX signal. DMS 114 and NCI-H1341 cells were treated with ±1 nM lurbinectedin ±2 μM berzosertib for 6 h, for the last hour EDU was added. S-phase cells were selected using PI content in FlowJo. γH2AX signal of S-phase cells is represented with a line at the median. (F) Average and median of ~10,000 cells for all groups were assessed and compared to control for both NCI-H1341 and DMS 114, biological replicates = 3. (G) NCI-H1341 cells displayed decreased EDU signal with the addition of lurbinectedin, this was partially rescued with cotreatment with berzosertib. Comparisons were made using an unpaired two-tailed Student's *t*-test in PRISM; DMS 114 data are displayed in Fig 2B.

H, I DMS 114 cells were treated with siRNA against ATR or Control siRNA. Three days later, they were treated with ±1 nM lurbinectedin ±2 μM berzosertib for 6 h, for the last hour EDU was added. γH2AX and EdU signal of S-phase cells is represented with a line at the median. A total of 10,000 cells for each condition were assessed, biological replicates = 2.

Source data are available online for this figure.

© 2023 The Authors

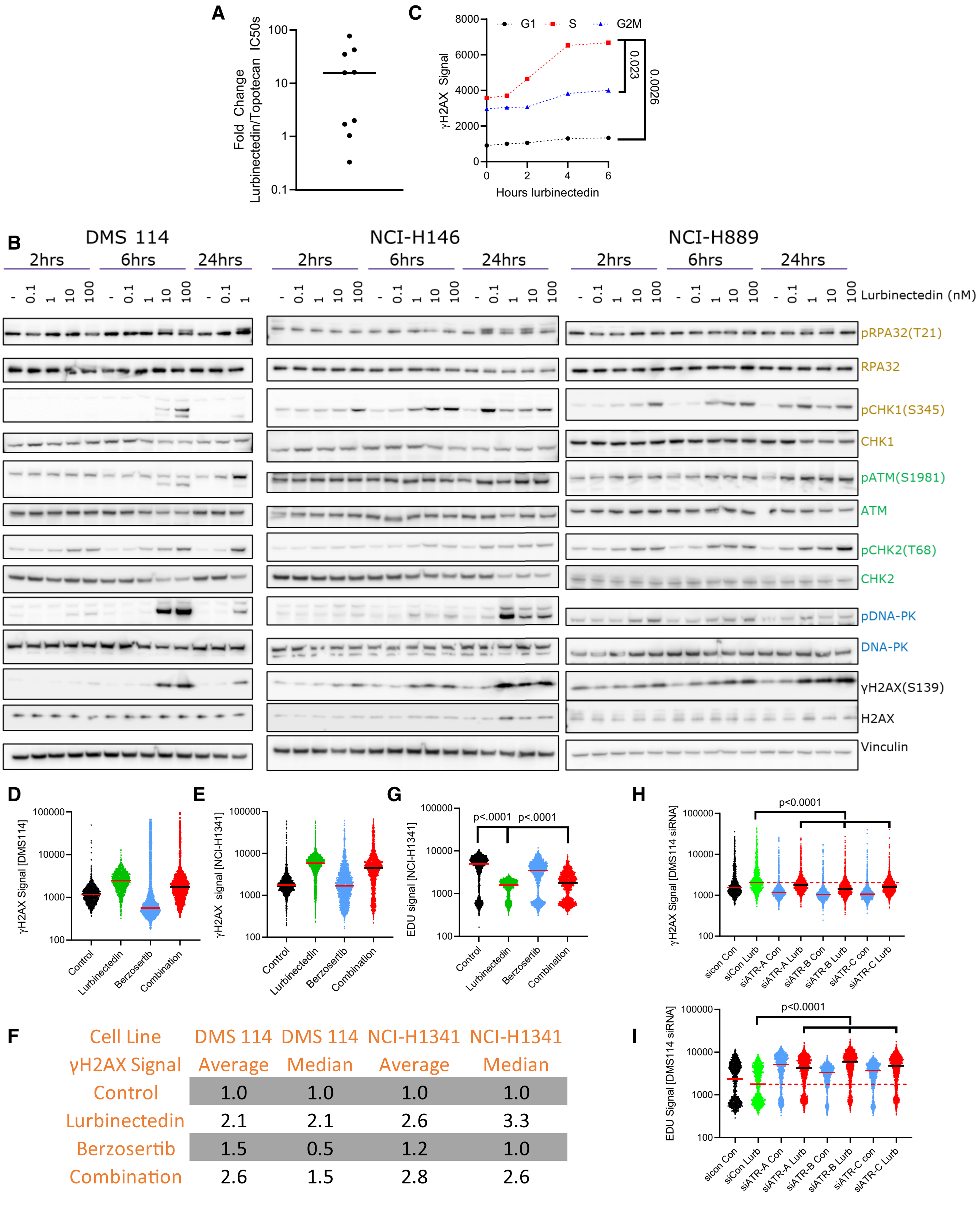

Cell Line	DMS 114	DMS 114	NCI-H1341	NCI-H1341
γH2AX Signal	Average	Median	Average	Median
Control	1.0	1.0	1.0	1.0
Lurbinectedin	2.1	2.1	2.6	3.3
Berzosertib	1.5	0.5	1.2	1.0
Combination	2.6	1.5	2.8	2.6

Figure EV1.

Figure EV2. XPG and XPA are required for combination efficacy, while ATR inhibition can rescue SLFN11-KO induced lurbinectedin resistance.

A, B BRCA2 KO led to increased lurbinectedin efficacy while XPG and XPA KO decreased efficacy, none of the knockouts displayed significantly different sensitivity to berzosertib. Lurbinectedin and berzosertib were treated in wild-type, BRCA2-KO, ERCC5-KO, and XPA-KO knockout DT40 cells at varying concentrations for 72 h, technical replicates = 3 and biological replicates = 3.

C, D Berzosertib inhibited HR competency at significantly lower concentrations (IC50 95%CI 0.2571 to 0.5847 μM) than required for berzosertib to inhibit viability (IC50 95%CI 2.285 to 3.016 μM). To assess HR competency, U2OS cells were stably transfected with a plasmid-expressing truncated GFP. Upon doxycycline treatment, this truncated version is cleaved, and cells which are HR competent are selectively able to repair the plasmid allowing for the expression of full-length GFP. Berzosertib impact on HR after accounting for changes to viability was plotted against berzosertib concentration. Each sample was assessed with > 10,000 cells, biological replicates = 3. IC50s were calculated utilizing the normalized variable slope model in PRISM.

E, F BRCA2-KO marginally reduced synergy of lurbinectedin and berzosertib while XPG-KO and XPA-KO reduced synergy to a greater extent. XPG-KO and XPA-KO cells maintain resistance to lurbinectedin even in the presence of berzosertib, indicating that berzosertib cannot rescue NER deficiency-induced resistance. The synergy of berzosertib and lurbinectedin across DT40 cells with wild-type, BRCA2-KO, ERCC5-KO, or XPA-KO was assessed after 72 h of treatment in a 10 × 10 matrix format; technical replicates = 3, biological replicates = 3. In (F), we demonstrate efficacy of lurbinectedin with 500 nM berzosertib (from the matrix data) as a marker of efficacy of the combination.

G–J We determined that SLFN11-KO in two DMS 114 models reduced lurbinectedin sensitivity (G) while not significantly effecting berzosertib sensitivity (H). SLFN11-KO led to increased synergy in both models (I). Accordingly, the addition of berzosertib rescued SLFN11-induced resistance in both models as lurbinectedin IC50s in all models in the presence of 500 nM berzosertib were similar (J). DMS 114 WT and two SLFN11 clones were plated in 10 × 10 matrix formats and lurbinectedin and berzosertib synergy was assessed, replicated = 3, *n* = 3.

 © 2023 The Authors

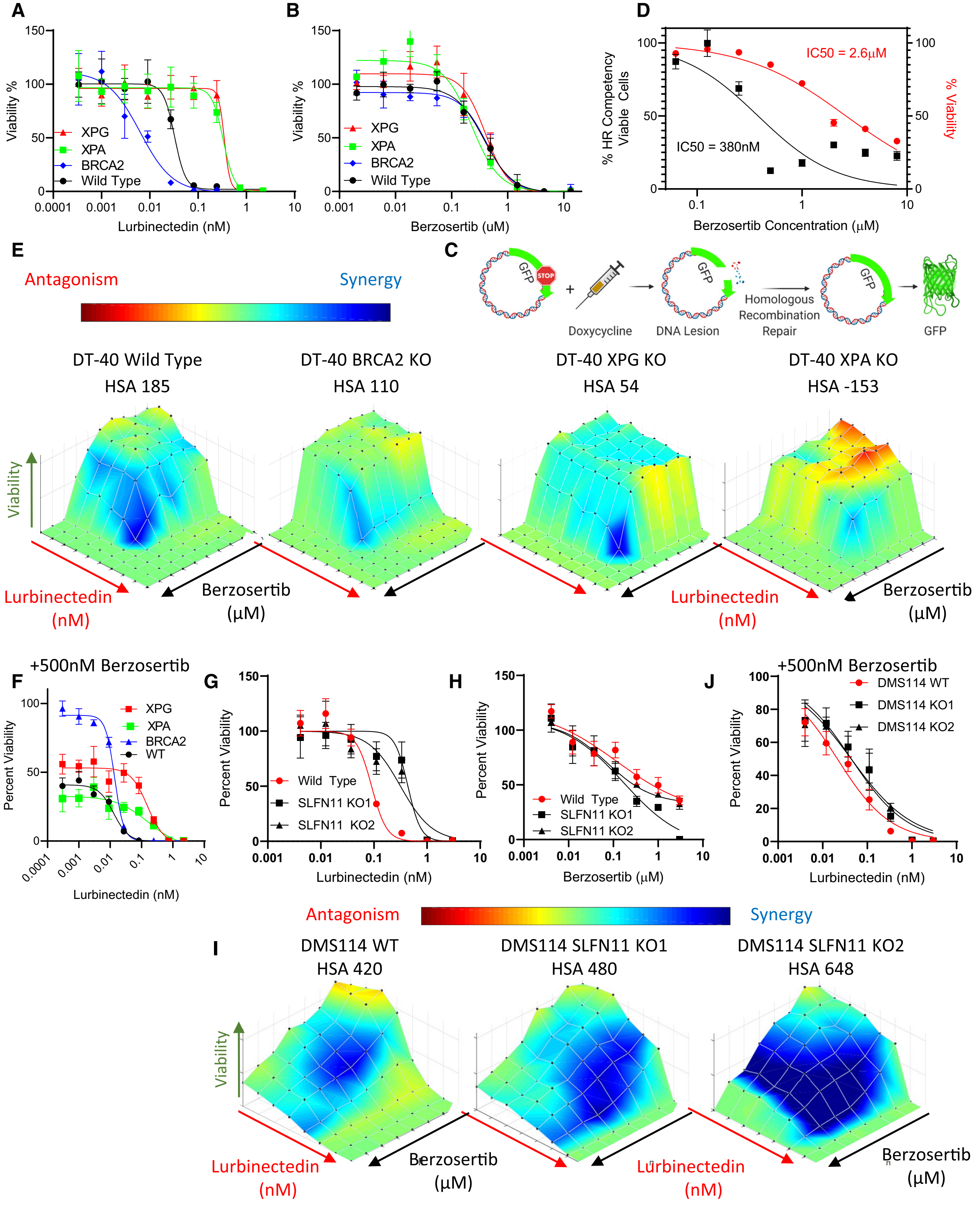

A
Viability %
XPG
XPA
BRCA2
Wild Type
Lurbinectedin (nM)
B
Berzosertib (uM)
D
% HR Competency Viable Cells
% Viability
IC50 = 2.6μM
IC50 = 380nM
Berzosertib Concentration (μM)
C
GFP
STOP
Doxycycline
DNA Lesion
Homologous Recombination Repair
E
Antagonism
Synergy
DT-40 Wild Type HSA 185
DT-40 BRCA2 KO HSA 110
DT-40 XPG KO HSA 54
DT-40 XPA KO HSA -153
Viability
Lurbinectedin (nM)
Berzosertib (μM)
F
+500nM Berzosertib
Percent Viability
WT
G
Wild Type
SLFN11 KO1
SLFN11 KO2
H
Berzosertib (μM)
J
DMS114 WT
DMS114 KO1
DMS114 KO2
I
DMS114 WT HSA 420
DMS114 SLFN11 KO1 HSA 480
DMS114 SLFN11 KO2 HSA 648

Figure EV2.

Figure EV3. G1 arrest reduces combination synergy.

A The efficacy and synergy of lurbinectedin and berzosertib were assessed across nine cell lines in a 10 × 10 matrix format, replicates = 3, n = 1. We determined IC50s for lurbinectedin and berzosertib utilizing an unnormalized variable slope model in PRISM. RNA values for all genes investigated for these cell lines are publicly available at https://discover.nci.nih.gov/rsconnect/cellminercdb. These data represent the statistics for simple linear regression as assessed in PRISM when plotting variable 1 against variable 2. Overall, these correlations are nonsignificant, however, SLFN11 expression trended toward negatively associating with lurbinectedin IC_{50}s in the nine cell lines.

B Lurbinectedin efficacy (lurbinectedin IC50), synergy between lurbinectedin and berzosertib (HSA), and combination efficacy (lurbinectedin IC50 in the presence of 500 nm berzosertib) were assessed. Here, we demonstrate different conditions between cells that are resistant (red/alive) and sensitive (purple/dead) to either lurbinectedin alone or lurbinectedin–berzosertib in order to differentiate these terms. Importantly, the combination can be effective without synergy (i.e., the third line), meaning these terms cannot be utilized interchangeably.

C Synergistic cell lines (NCI-H1341, NCI-H841) as compared to less synergistic cell lines (NCI-H146, NCI-H899) displayed increased S-phase accumulation after lurbinectedin treatment, which could be ameliorated with berzosertib cotreatment. NCI-H1341, NCI-H841 NCI-H146, and NCI-H899 were treated with ±1 nM lurbinectedin ±2 μM berzosertib for 24 h and assessed using PI to determine cell cycle with flow cytometry analysis in FlowJo. Each treatment group for each cell line had 10,000 cells, technical replicates = 4, and biological replicates = 3.

D p21 protein normalized to control tubulin across two independent experiments with fresh samples correlated with log2 *CDKN1A* RNA expression. We assessed p21 protein expression by western blot in nine cell lines; error bars represent SD. Pearson correlation was assessed in PRISM.

E Cells that were halted in G1 using a double thymidine block led to a decrease in sensitivity to lurbinectedin, and to the combination of lurbinectedin and berzosertib. Here, cells treated with berzosertib, lurbinectedin, and the combination without thymidine are normalized to thymidine-free and untreated control, and cells treated with berzosertib, lurbinectedin, and the combination along with thymidine are normalized to thymidine-alone control cells. NCI-H841 (SCLC cell line chosen largely due to not being sensitive to loss of viability upon thymidine arrest) cells underwent a double-thymidine block-enforcing G1 arrest. Cells were treated with ±lurbinectedin 1 nM ± 2 μM berzosertib ± enforcement of thymidine arrest and collected after 72 h, replicates = 4, n = 3, P-values indicate unpaired two-tailed Student's t-test in PRISM, error bars are representative of SD.

F p21 overexpression in DMS 114 cells led to a decrease in lurbinectedin–berzosertib combination synergy; DMS 114 cells were treated with mock transfection or with overexpression of Flag-tagged wild-type p21 (Addgene Plasmid #16240). Cells were split at 1,000 cells/well into 384-well plates after 24 h and then treated the following day with lurbinectedin and berzosertib in a 10 × 6 matrix format, cells were collected after 72 h, and synergy was assessed; technical replicates = 4, biological replicates = 2.

 © 2023 The Authors

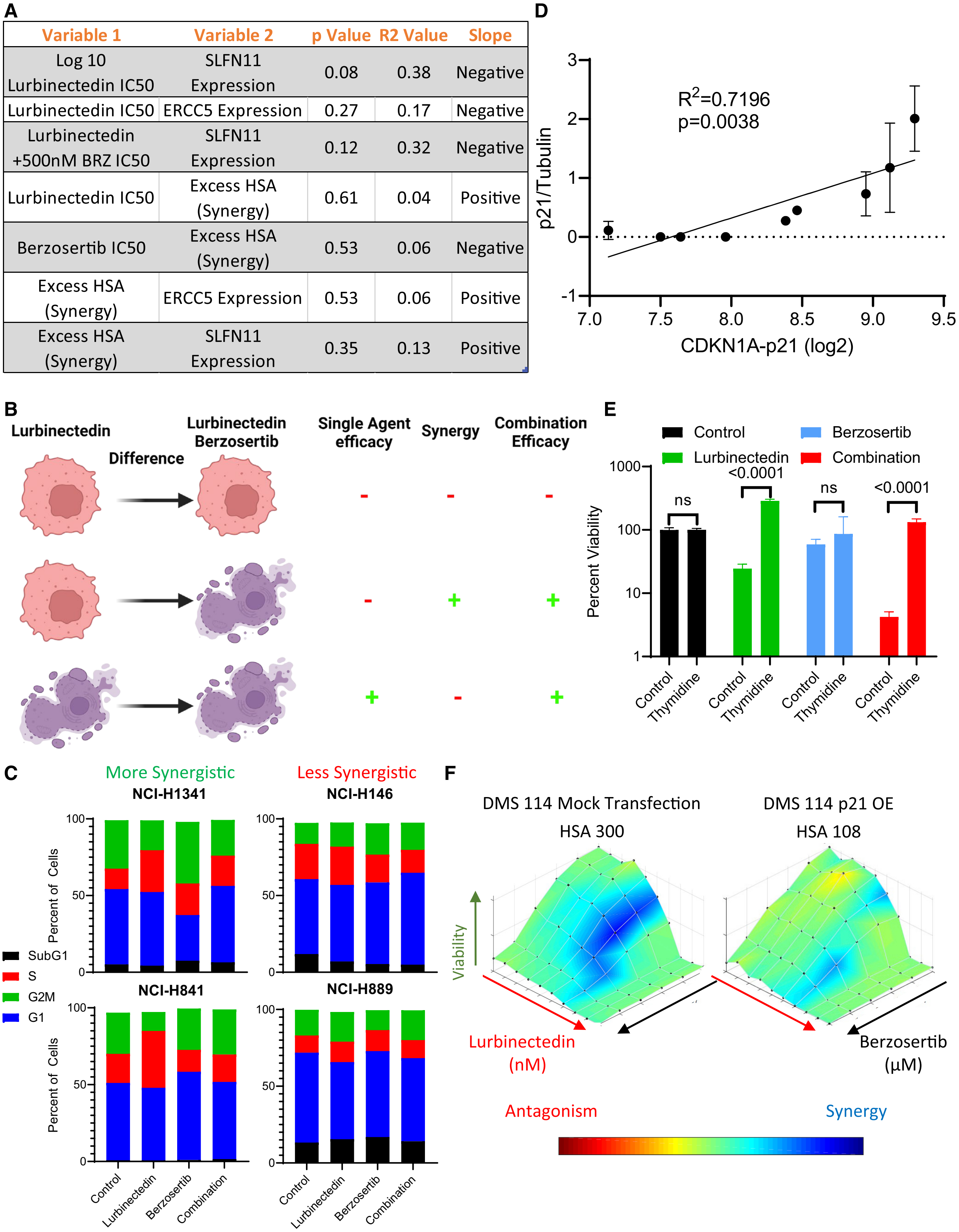
A
Variable 1 | Variable 2 | p Value | R2 Value | Slope
Log 10 Lurbinectedin IC50 | SLFN11 Expression | 0.08 | 0.38 | Negative
Lurbinectedin IC50 | ERCC5 Expression | 0.27 | 0.17 | Negative
Lurbinectedin +500nM BRZ IC50 | SLFN11 Expression | 0.12 | 0.32 | Negative
Lurbinectedin IC50 | Excess HSA (Synergy) | 0.61 | 0.04 | Positive
Berzosertib IC50 | Excess HSA (Synergy) | 0.53 | 0.06 | Negative
Excess HSA (Synergy) | ERCC5 Expression | 0.53 | 0.06 | Positive
Excess HSA (Synergy) | SLFN11 Expression | 0.35 | 0.13 | Positive
D
R2=0.7196
p=0.0038
p21/Tubulin
CDKN1A-p21 (log2)
B
Lurbinectedin
Difference
Lurbinectedin Berzosertib
Single Agent efficacy
Synergy
Combination Efficacy
E
Control
Lurbinectedin
Berzosertib
Combination
Percent Viability
ns
<0.0001
ns
<0.0001
Control
Thymidine
C
More Synergistic
NCI-H1341
Less Synergistic
NCI-H146
NCI-H841
NCI-H889
Percent of Cells
SubG1
S
G2M
G1
Control
Lurbinectedin
Berzosertib
Combination
F
DMS 114 Mock Transfection
HSA 300
DMS 114 p21 OE
HSA 108
Viability
Lurbinectedin (nM)
Berzosertib (μM)
Antagonism
Synergy

Figure EV3.

Figure EV4. TP53 deficient cells may be more sensitive to lurbinectedin-berzosertib combination.

A Lurbinectedin treatment caused a decrease in p21 in NCI-H899 (p53 mutant) while it caused an increase in p21 in NCI-H146 (p53 wild-type) as assessed by immunoblotting. Cells were treated with ±1 nM lurbinectedin for 6 h and collected.

B, C The ATM inhibitor KU60019 increased synergy and increased combination efficacy of lurbinectedin and berzosertib in NCI-H146 cells (p53 wild = type) while not increasing synergy or combination efficacy to as great as an extent in NCI-H889 (p53 mutant). H889 and NCI-H146 cells were treated with a combination of lurbinectedin and berzosertib in an 8 × 8 matrix format ±2 μM KU60019 for 72 h, technical replicates = 4 and biological replicates = 2.

D, E PDX 592484 displayed greater synergy of lurbinectedin with berzosertib as compared to the PDX 456648 as displayed by the greater increase in lurbinectedin sensitivity with the addition of berzosertib. Patient-derived xenograft organoids were dissociated and plated in 384-well plates and treated with lurbinectedin ± 500 nM berzosertib for 72 h, technical replicates = 3, and biological replicates = 2.

F, G The more synergistic organoid model (PDX 592484) expressed less *CDKN1A* (p21) and *ERCC5* (XPG) potentially explaining the not only increased synergy but also decreased overall efficacy of lurbinectedin even in the presence of berzosertib as compared to PDX 456648. Patient-derived xenograft organoids samples were assessed using RNA seq, data are shown as TMM FPKM; PDX456648 technical replicates = 6 and PDX 592484 technical replicates = 4 for RNA seq; we utilized unpaired Student's *t*-tests in PRISM to compare groups.

H Neuroendocrine scores inversely associated with synergy in our nine cell lines, with NE cells displaying decreased synergy as compared to non-NE cells. Neuroendocrine scores and SCLC subtype clustering data (ASCL1, NEUROD1, POU2F3, and YAP1) are publicly available at https://discover.nci.nih.gov/rsconnect/cellminercdb, and HSA was determined as described previously. Pearson correlation was determined using PRISM.

 © 2023 The Authors

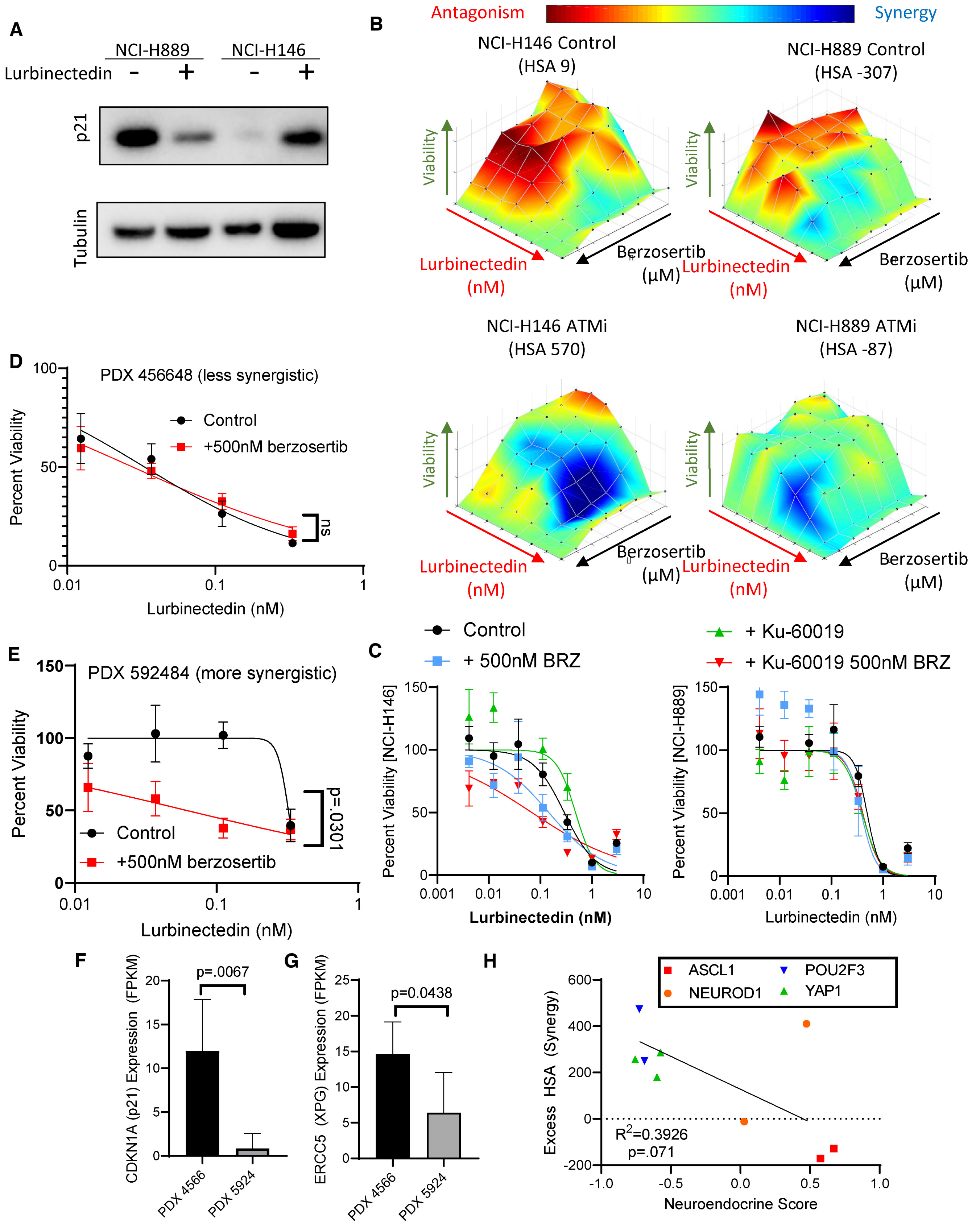
A
NCI-H889
NCI-H146
Lurbinectedin - + - +
p21
Tubulin
B
Antagonism
Synergy
NCI-H146 Control
(HSA 9)
NCI-H889 Control
(HSA -307)
NCI-H146 ATMi
(HSA 570)
NCI-H889 ATMi
(HSA -87)
Viability
Lurbinectedin (nM)
Berzosertib (µM)
D
PDX 456648 (less synergistic)
Control
+500nM berzosertib
Percent Viability
Lurbinectedin (nM)
ns
E
PDX 592484 (more synergistic)
p=.0301
C
Control
+ 500nM BRZ
+ Ku-60019
+ Ku-60019 500nM BRZ
Percent Viability [NCI-H146]
Percent Viability [NCI-H889]
F
CDKN1A (p21) Expression (FPKM)
p=.0067
PDX 4566
PDX 5924
G
ERCC5 (XPG) Expression (FPKM)
p=0.0438
H
ASCL1
POU2F3
NEUROD1
YAP1
Excess HSA (Synergy)
Neuroendocrine Score
R^2=0.3926
p=.071

Figure EV4.

Figure EV5. Berzosertib and lurbinectedin efficacy and synergy *in vivo*.

A In the PDX-06 model, lurbinectedin appeared to induce p-CHK1 activation which was reduced with berzosertib cotreatment (quantified in Fig 4B). PDX-06 tumors were collected 24 h after being dosed with the indicated drugs. We assessed targets in the ATR (yellow/gold), ATM (green), and DNA-PK (blue) damage repair pathways as well as γH2AX (DNA damage marker).

B Lurbinectedin and berzosertib were less effective in the more aggressive mouse model of SCLC PDX-03 as compared to PDX-06 (Fig 4A). PDX-03 was treated the same as PDX-06 with lurbinectedin (0.18 mg/kg IV, with an increased volume of 200 μl vs. 100 μl) and berzosertib (20 mg/kg IP) in a format mirroring our clinical trial with topotecan/berzosertib in SCLC, lurbinectedin day 1, and berzosertib days 2 and 5 of a 7-day cycle. To approximate efficacy across an experiment with significant toxicity, we assessed growth rate as tumor size at animal death due to either toxicity or progression of tumor minus tumor volume at date of initiation, divided by days between initiation and final assessment of tumor for a growth rate.

C The PDX-03 model was more aggressive than the PDX-06 model as demonstrated by comparing rate of growth for the control arms for both experiments. PDX-06 mice = 10, PDX-03 mice = 11, and an unpaired two-tailed Student's *t*-test was used to compare rates.

D Lurbinectedin and the combination were less toxic in PDX-03 as compared to PDX-06, likely due to the increased volume for dosing of lurbinectedin, however, several mice still required sacrifice. Mouse body weights corresponding to Appendix Fig S7B, mice which required sacrifice due to toxicity (body weight loss or other) are quantified to the right in red.

E The combination of lurbinectedin and berzosertib overall appeared to decrease replication and increase markers of cell death. We assessed markers of replication (ki-67) and cell death (cleaved caspase-3 and necrosis) as assessed by IHC and H&E staining from tumors after collection from all mice possibly from our PDX-03 efficacy study (Appendix Fig S7B). Cleaved caspase and ki-67 are reported as percent positive of cells, while necrosis is reported as percent area. Lines represent median of groups as do numbers above.

F, G Similar to Fig 4D to determine differences in synergy with different scheduling regiments of lurbinectedin and berzosertib, we treated NCI-446 cells in a 10 × 10 matrix format; technical replicates = 3, biological replicates = 1. All groups were treated for 24 h with lurbinectedin, while group 1 was pretreated with berzosertib, group 2 was cotreated with berzosertib, group 3 was co- and posttreated with berzosertib, and group 4 was posttreated with berzosertib for 24 h. At the end of 4 days, cells were collected and synergy was assessed across the matrixes. We determined that group 3 (co- and posttreatment) displayed the greatest degree of synergy, mirroring our results with DMS 114 cells in Fig 4D. We also assessed synergy of DMS 114 cells and our PDX-06 cell line in a 12 × 3 matrix format, technical replicates = 4 and biological replicates = 2, and determined that PDX-06 cells appeared to have less synergy than DMS 114 cells (F) and were more sensitive to lurbinectedin (G).

H The most effective schedule in the DMS 114 xenograft model was treating with lurbinectedin and berzosertib cotreated and posttreated (lurbinectedin day 1, berzosertib days 1, 2) mirroring our *in vitro* results. The most effective scheduling model is shown in Fig 4F, error bars are representative of SEM, mice = 10 per group.

I In the DMS 114 mouse model of SCLC, lurbinectedin day 1 and berzosertib days 1 and 2 were nontoxic. Mouse body weights from the PDX experiment are demonstrated in Appendix Fig S1H. The number of mice sacrificed due to toxicity in each group is also displayed.

J, K Concentrations of berzosertib and lurbinectedin in mouse plasma were assessed in mice utilizing mass spectrometry after retro-orbital collection of blood at the indicated time points after dosing with lurbinectedin (0.18 mg/kg intravenous) and berzosertib (50 mg/kg oral), mice = 5 per group; error bars are representative of SD.

 © 2023 The Authors

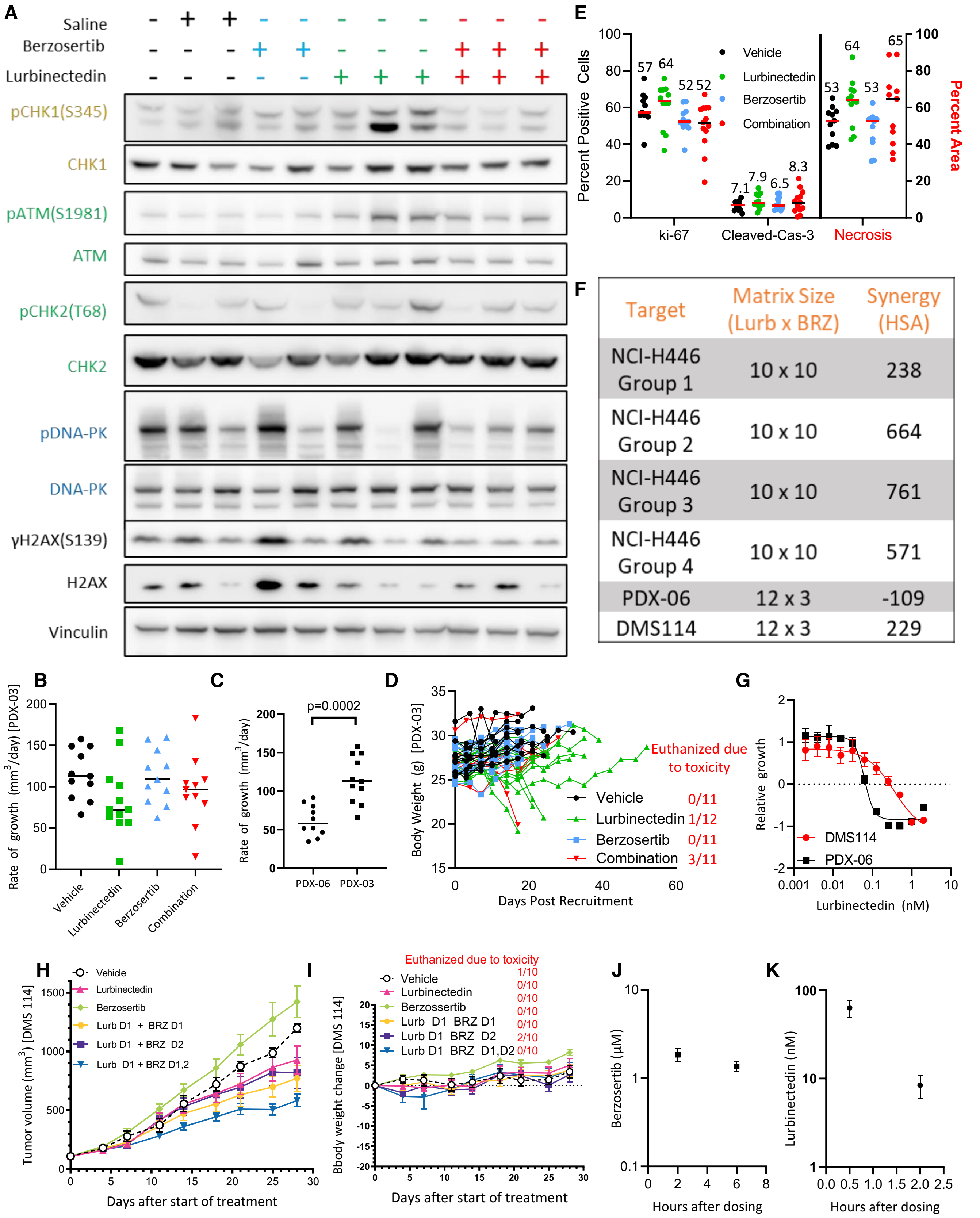
A
Saline
Berzosertib
Lurbinectedin
pCHK1(S345)
CHK1
pATM(S1981)
ATM
pCHK2(T68)
CHK2
pDNA-PK
DNA-PK
γH2AX(S139)
H2AX
Vinculin
E
Percent Positive Cells
Percent Area
Vehicle
Lurbinectedin
Berzosertib
Combination
57 64 52 52
7.1 7.9 6.5 8.3
64 65 53 53
ki-67
Cleaved-Cas-3
Necrosis
F
Target
Matrix Size (Lurb x BRZ)
Synergy (HSA)
NCI-H446 Group 1 10 x 10 238
NCI-H446 Group 2 10 x 10 664
NCI-H446 Group 3 10 x 10 761
NCI-H446 Group 4 10 x 10 571
PDX-06 12 x 3 -109
DMS114 12 x 3 229
B
Rate of growth (mm3/day) [PDX-03]
Vehicle
Lurbinectedin
Berzosertib
Combination
C
p=0.0002
Rate of growth (mm3/day)
PDX-06
PDX-03
D
Body Weight (g) [PDX-03]
Euthanized due to toxicity
Vehicle 0/11
Lurbinectedin 1/12
Berzosertib 0/11
Combination 3/11
Days Post Recruitment
G
Relative growth
DMS114
PDX-06
Lurbinectedin (nM)
H
Tumor volume (mm3) [DMS 114]
Vehicle
Lurbinectedin
Berzosertib
Lurb D1 + BRZ D1
Lurb D1 + BRZ D2
Lurb D1 + BRZ D1,2
Days after start of treatment
I
Euthanized due to toxicity
Vehicle 1/10
Lurbinectedin 0/10
Berzossertib 0/10
Lurb D1 BRZ D1 0/10
Lurb D1 BRZ D2 0/10
Lurb D1 BRZ D1,D2 2/10
Bbody weight change [DMS 114]
Days after start of treatment
J
Berzosertib (µM)
Hours after dosing
K
Lurbinectedin (nM)
Hours after dosing

Figure EV5.
