# Supplementary material for: ATR inhibition augments the efficacy of lurbinectedin in small‐cell lung cancer
Source: EMBO Mol Med. 2023 Jul 25;15(8):e17313. doi: 10.15252/emmm.202217313 (PMC10405061; doi:10.15252/emmm.202217313)
Supplement: Supplementary file 5 — Table EV3 [file EMMM-15-e17313-s013.pdf]

EV Table 3

	Combination Synergy				Combination Efficacy			
	Synergy	CDKN1A (RNA)	NE score	MYC (RNA)	SLFN11 (RNA)	Lurbinectedin efficacy	ERCC5 (RNA)	Combination efficacy
Combination efficacy	-0.4	0.3	0.2	-0.6	-0.6	0.6	-0.8	1.0
ERCC5 (RNA)	0.2	-0.4	0.0	0.3	0.5	-0.4	1.0	
Lurbinectedin efficacy	0.2	-0.4	-0.1	0.1	-0.5	1.0		
SLFN11 (RNA)	0.4	0.0	-0.7	0.1	1.0			
MYC (RNA)	0.6	-0.5	-0.2	1.0				
NE score	-0.6	0.3	1.0					
CDKN1A (RNA)	-0.8	1.0						
Synergy	1.0							

Absolute Correlation: 0–1

Expanded View Table 3: We have determined that synergy of lurbinectedin and berzosertib is governed by *CDKN1A* (p21) which in turn is regulated by NE differentiation and *MYC* expression. The efficacy of the combination (IC50 of lurbinectedin in the presence of 500nM berzosertib) correlates most strongly with *ERCC5* (XPG) expression followed by lurbinectedin solo agent efficacy (ic50) and *SLFN11* expression. Here the numbers indicate Pearson correlation values of variables from our panel of nine cell lines while the color scale is indicative of absolute Pearson correlation values. As synergy is denoted as HSA values and combination efficacy is representative of ic50 values of lurbinectedin in the presence of 500nM berzosertib, positive correlation with synergy indicates greater synergy while negative correlation with ic50 values indicates greater efficacy (i.e. MYC RNA correlates positively with synergy and negatively with ic50 values indicating with greater MYC expression there is more synergy and increased efficacy of the combination).
