# Supplementary material for: ATR inhibition augments the efficacy of lurbinectedin in small‐cell lung cancer
Source: EMBO Mol Med. 2023 Jul 25;15(8):e17313. doi: 10.15252/emmm.202217313 (PMC10405061; doi:10.15252/emmm.202217313)
Supplement: Supplementary file 6 — Source Data for Expanded View [file EMMM-15-e17313-s012.zip › Source_data_fig_EV1_A.pptx]

## Slide 1
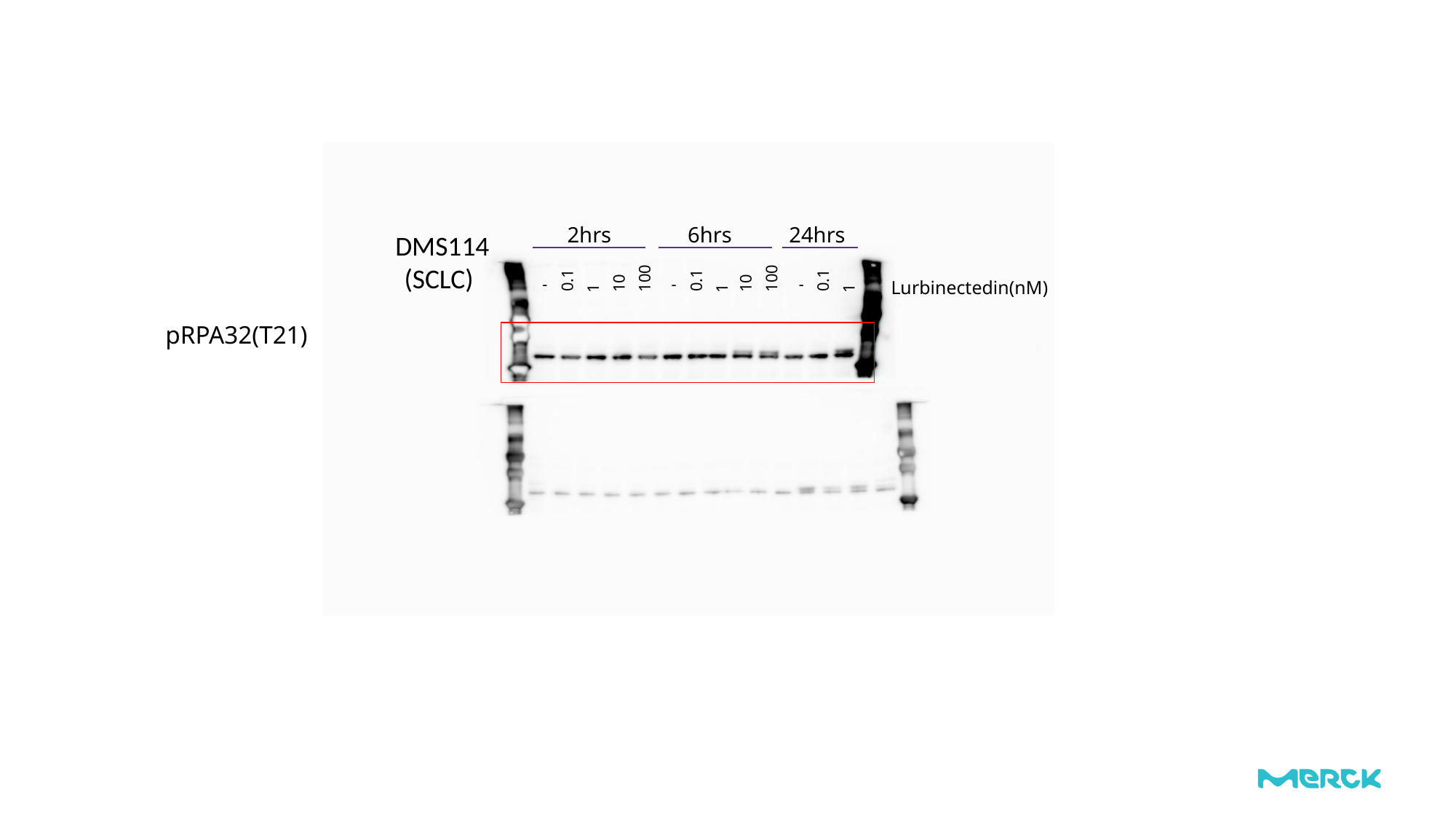

2hrs
6hrs
24hrs
DMS114
(SCLC)
100
100
0.1
0.1
0.1
Lurbinectedin(nM)
10
10
-
-
-
1
1
1
pRPA32(T21)

## Slide 2
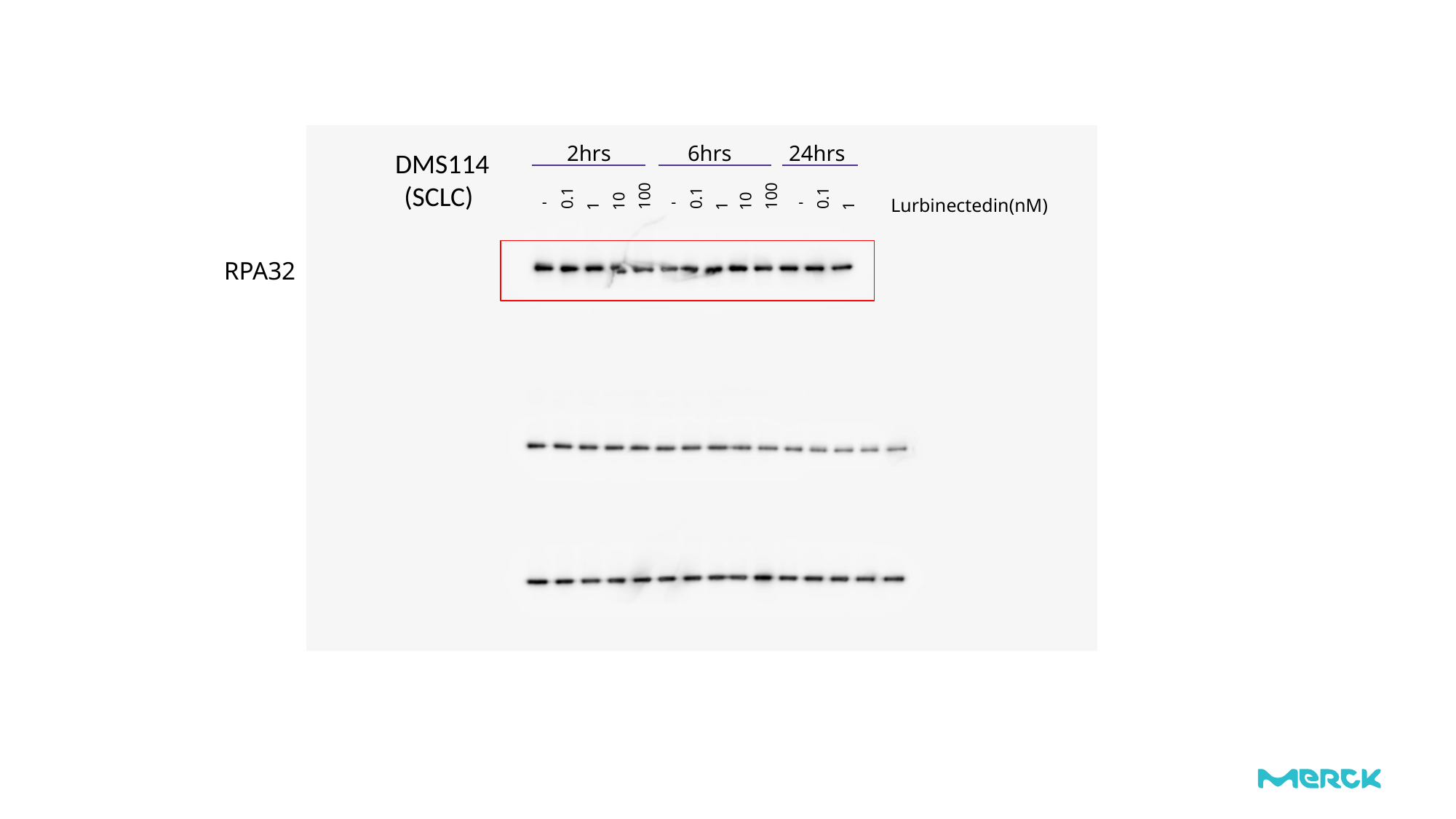

2hrs
6hrs
24hrs
DMS114
(SCLC)
100
100
0.1
0.1
0.1
Lurbinectedin(nM)
10
10
-
-
-
1
1
1
RPA32

## Slide 3
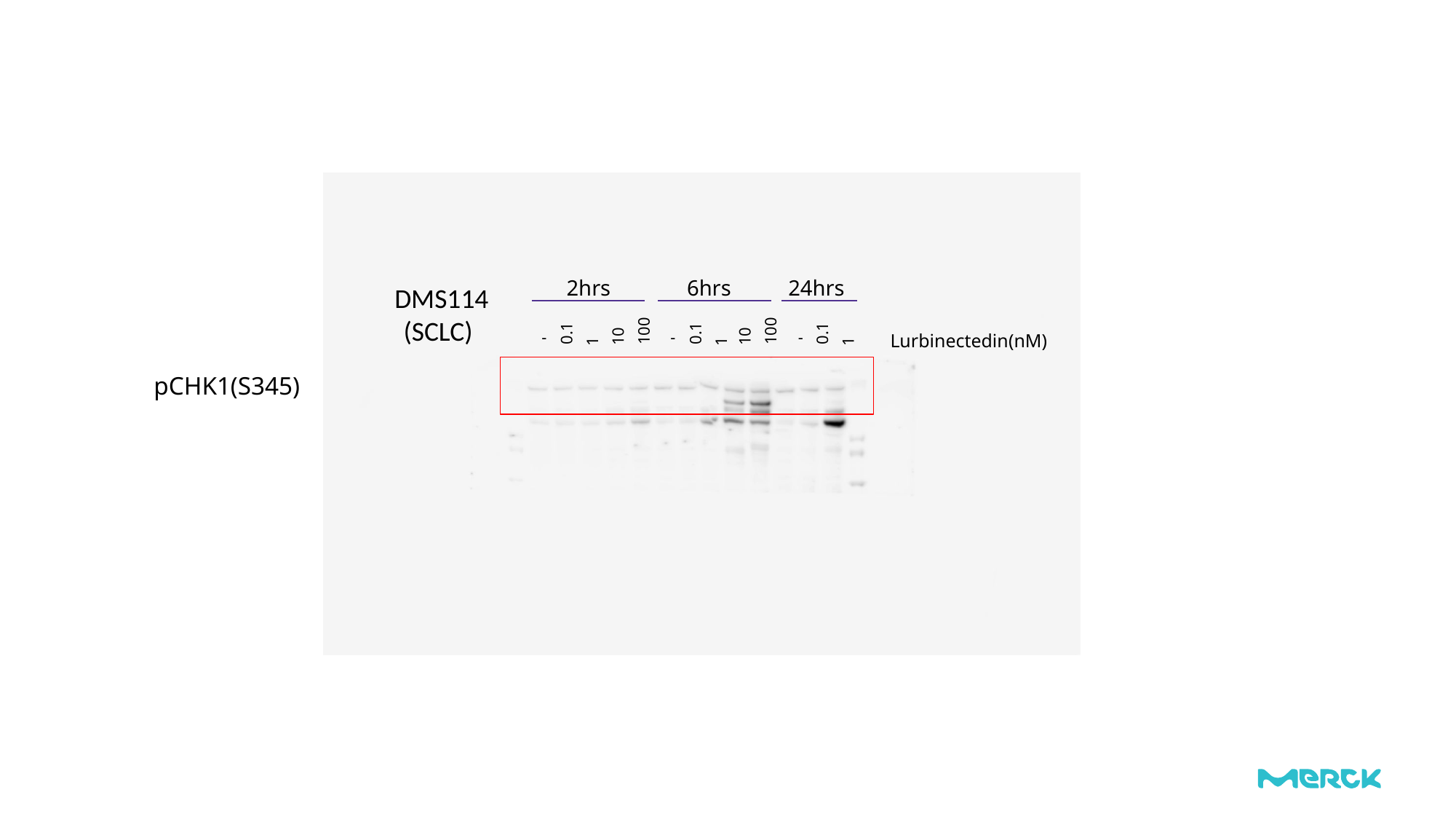

2hrs
6hrs
24hrs
DMS114
(SCLC)
100
100
0.1
0.1
0.1
Lurbinectedin(nM)
10
10
-
-
-
1
1
1
pCHK1(S345)

## Slide 4
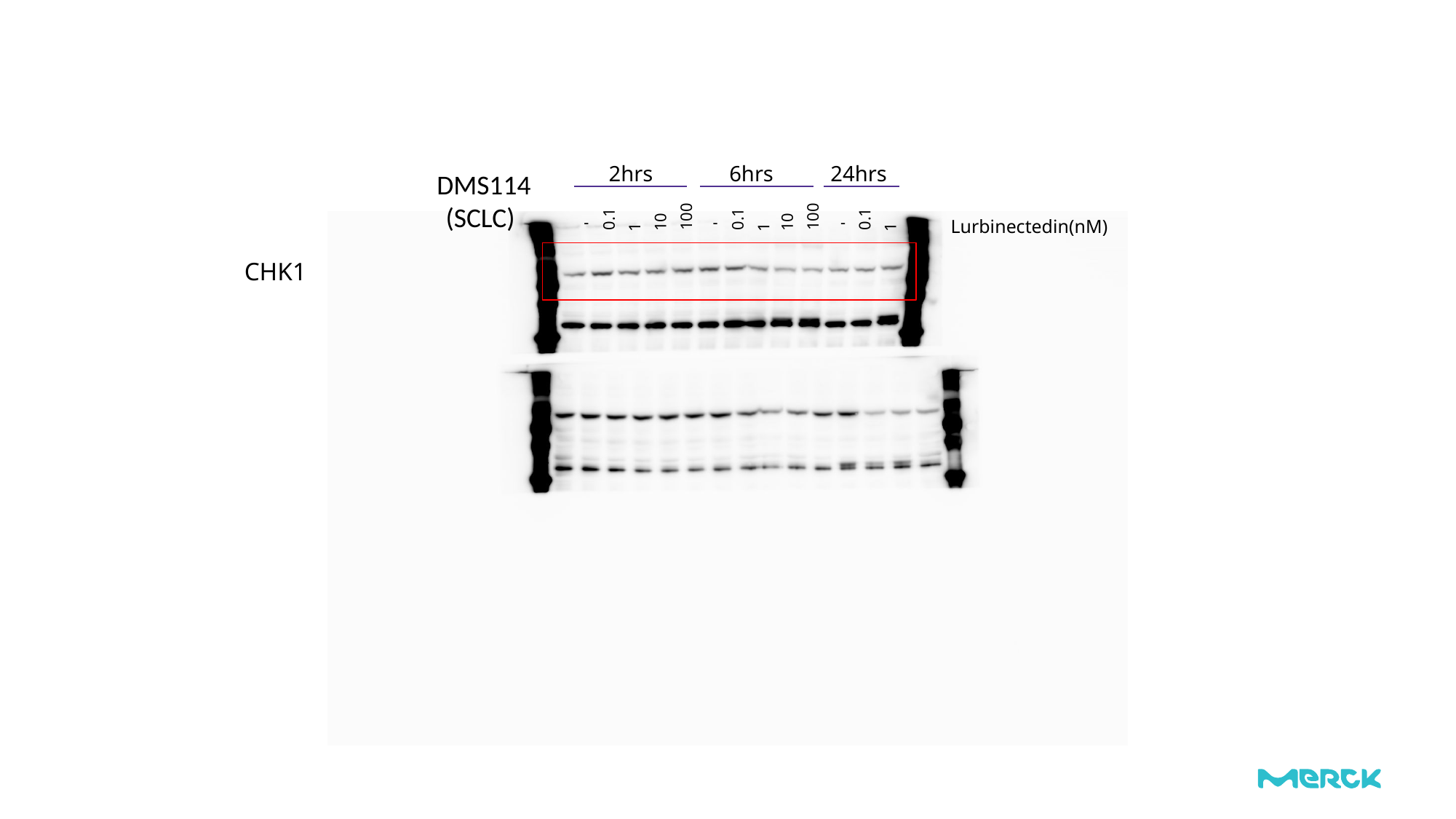

2hrs
6hrs
24hrs
DMS114
(SCLC)
100
100
0.1
0.1
0.1
Lurbinectedin(nM)
10
10
-
-
-
1
1
1
CHK1

## Slide 5
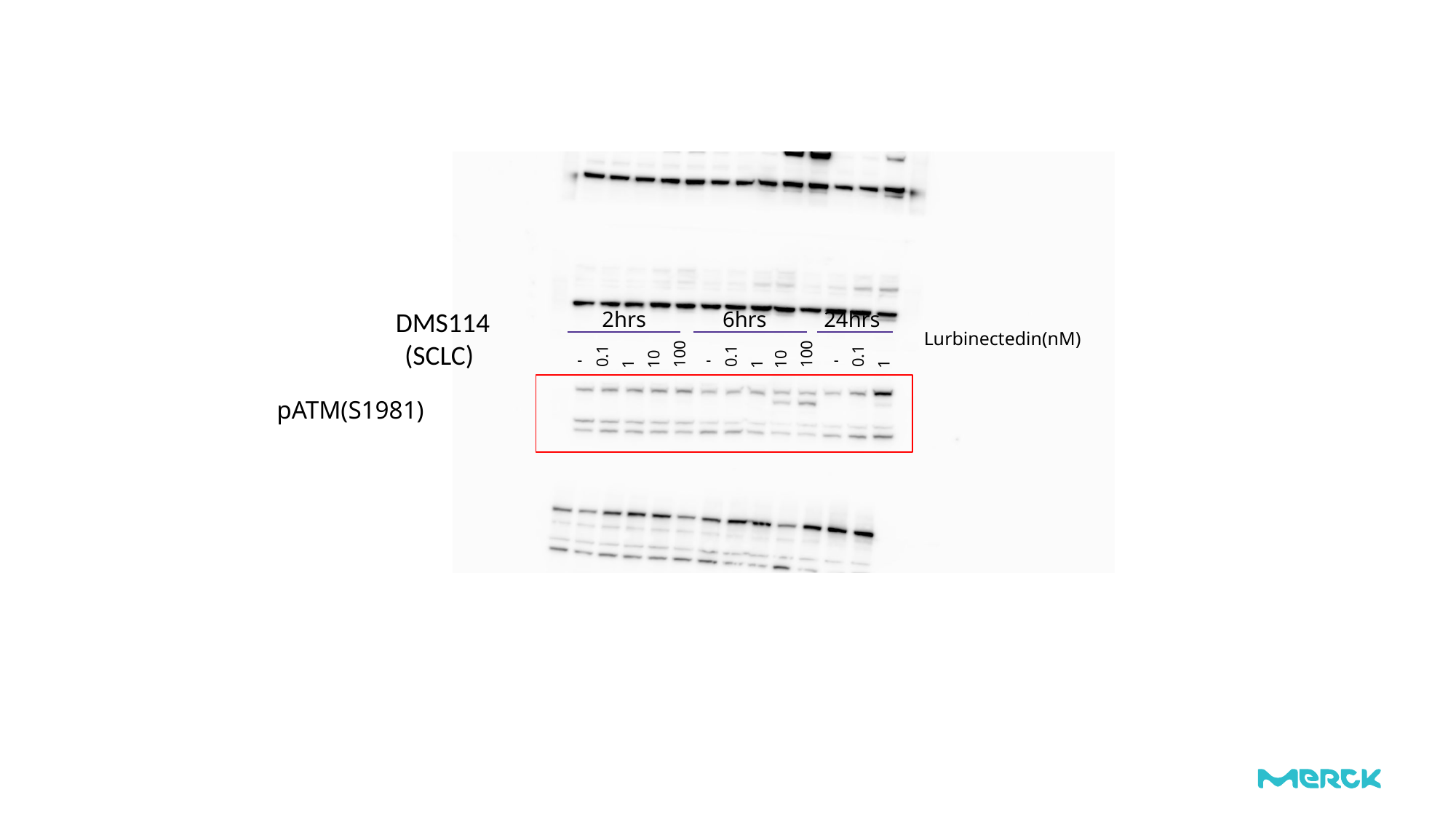

DMS114
(SCLC)
2hrs
6hrs
24hrs
Lurbinectedin(nM)
100
100
0.1
0.1
0.1
10
10
-
-
-
1
1
1
pATM(S1981)

## Slide 6
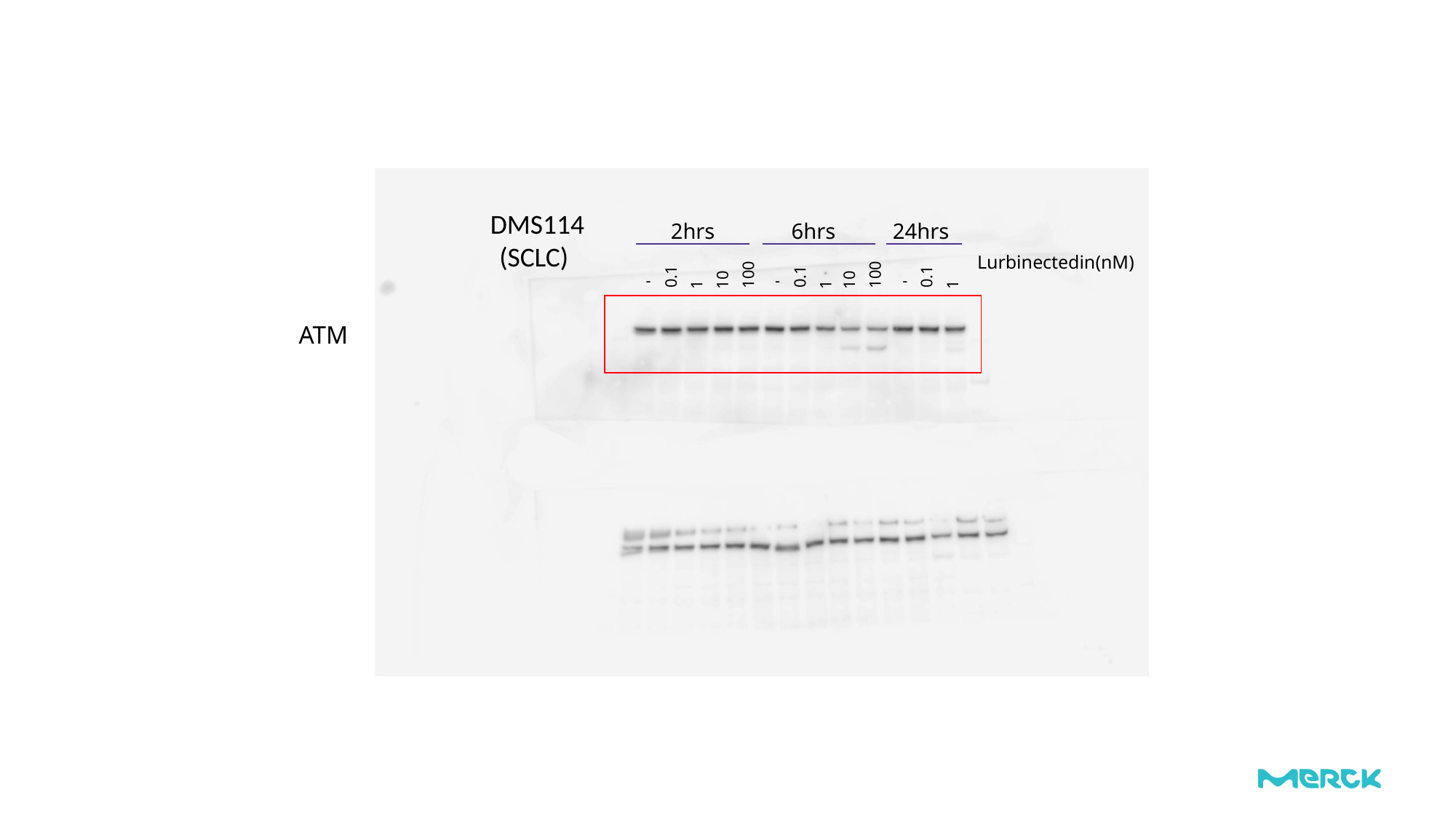

DMS114
(SCLC)
2hrs
6hrs
24hrs
Lurbinectedin(nM)
100
100
0.1
0.1
0.1
10
10
-
-
-
1
1
1
ATM

## Slide 7
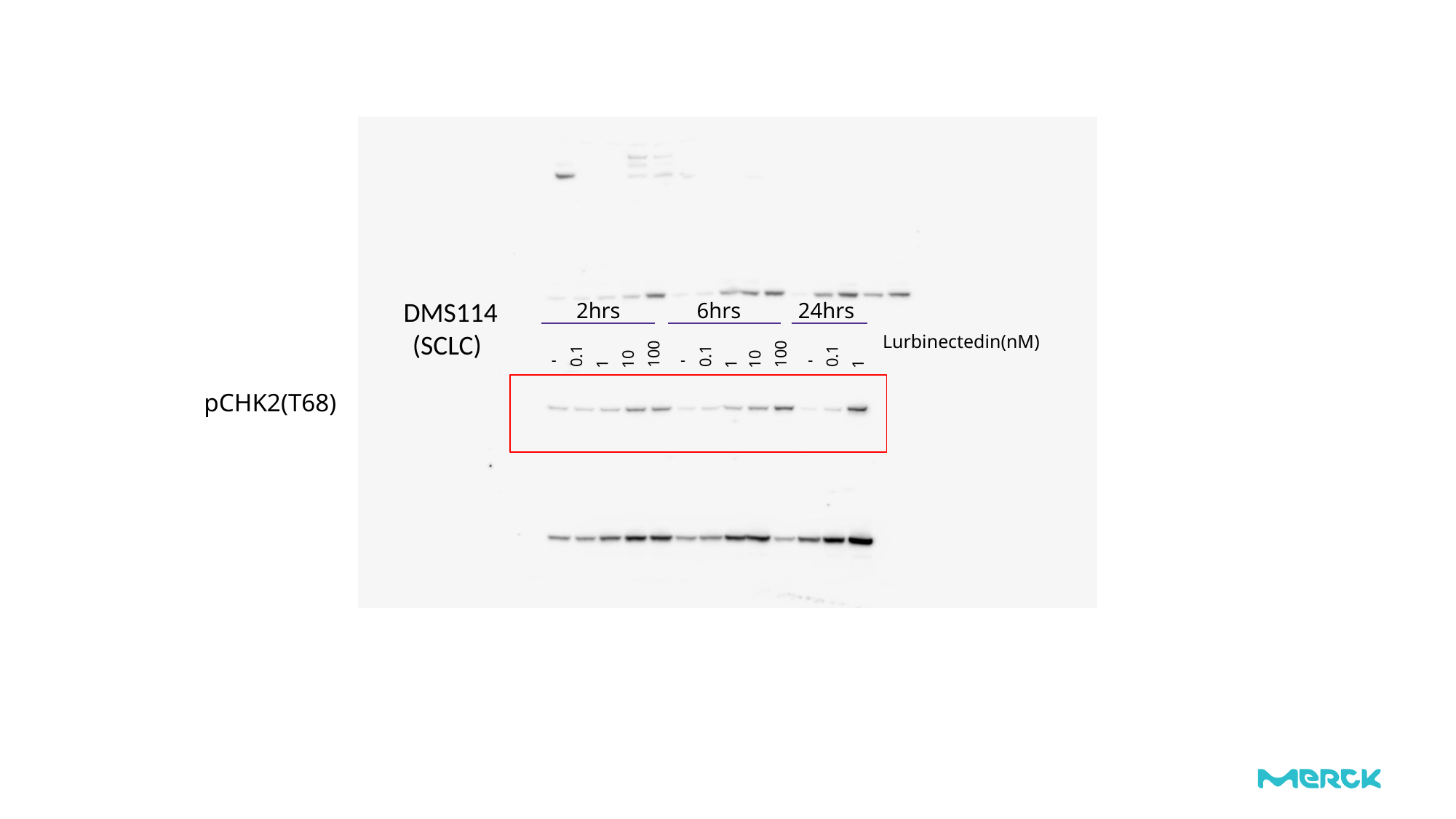

DMS114
(SCLC)
2hrs
6hrs
24hrs
Lurbinectedin(nM)
100
100
0.1
0.1
0.1
10
10
-
-
-
1
1
1
pCHK2(T68)

## Slide 8
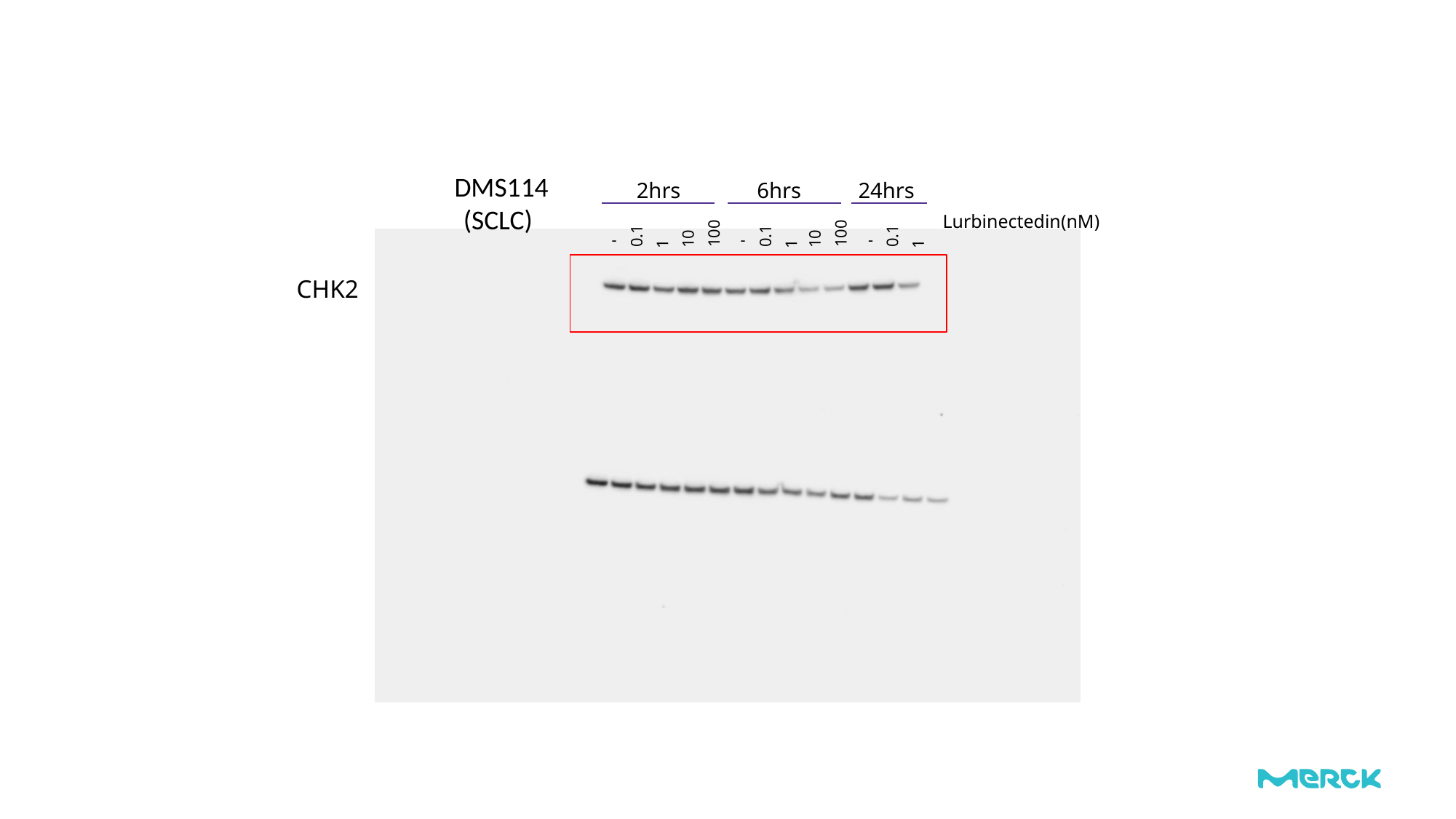

DMS114
(SCLC)
2hrs
6hrs
24hrs
Lurbinectedin(nM)
100
100
0.1
0.1
0.1
10
10
-
-
-
1
1
1
CHK2

## Slide 9
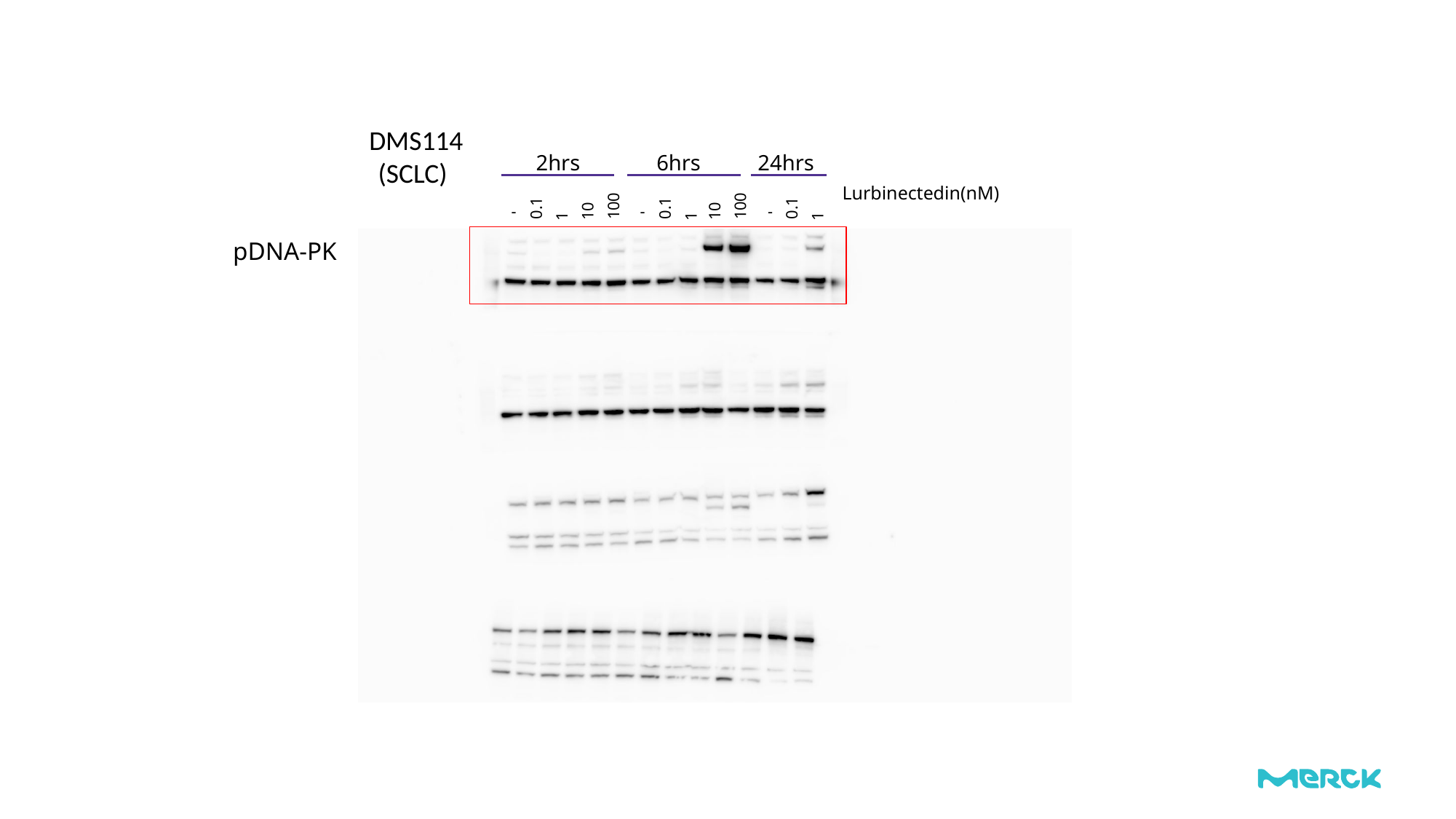

DMS114
(SCLC)
2hrs
6hrs
24hrs
Lurbinectedin(nM)
100
100
0.1
0.1
0.1
10
10
-
-
-
1
1
1
pDNA-PK

## Slide 10
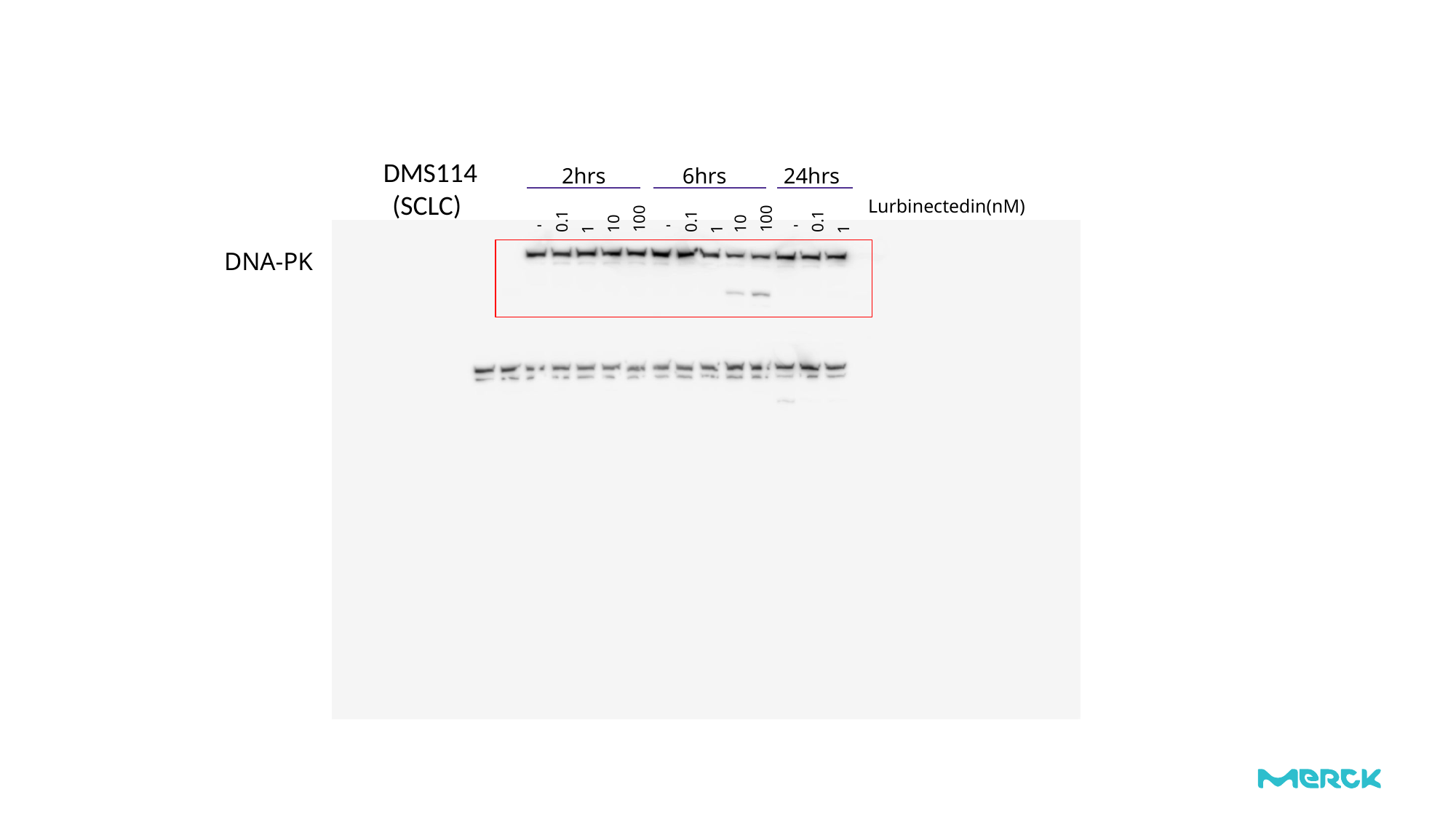

DMS114
(SCLC)
2hrs
6hrs
24hrs
Lurbinectedin(nM)
100
100
0.1
0.1
0.1
10
10
-
-
-
1
1
1
DNA-PK

## Slide 11
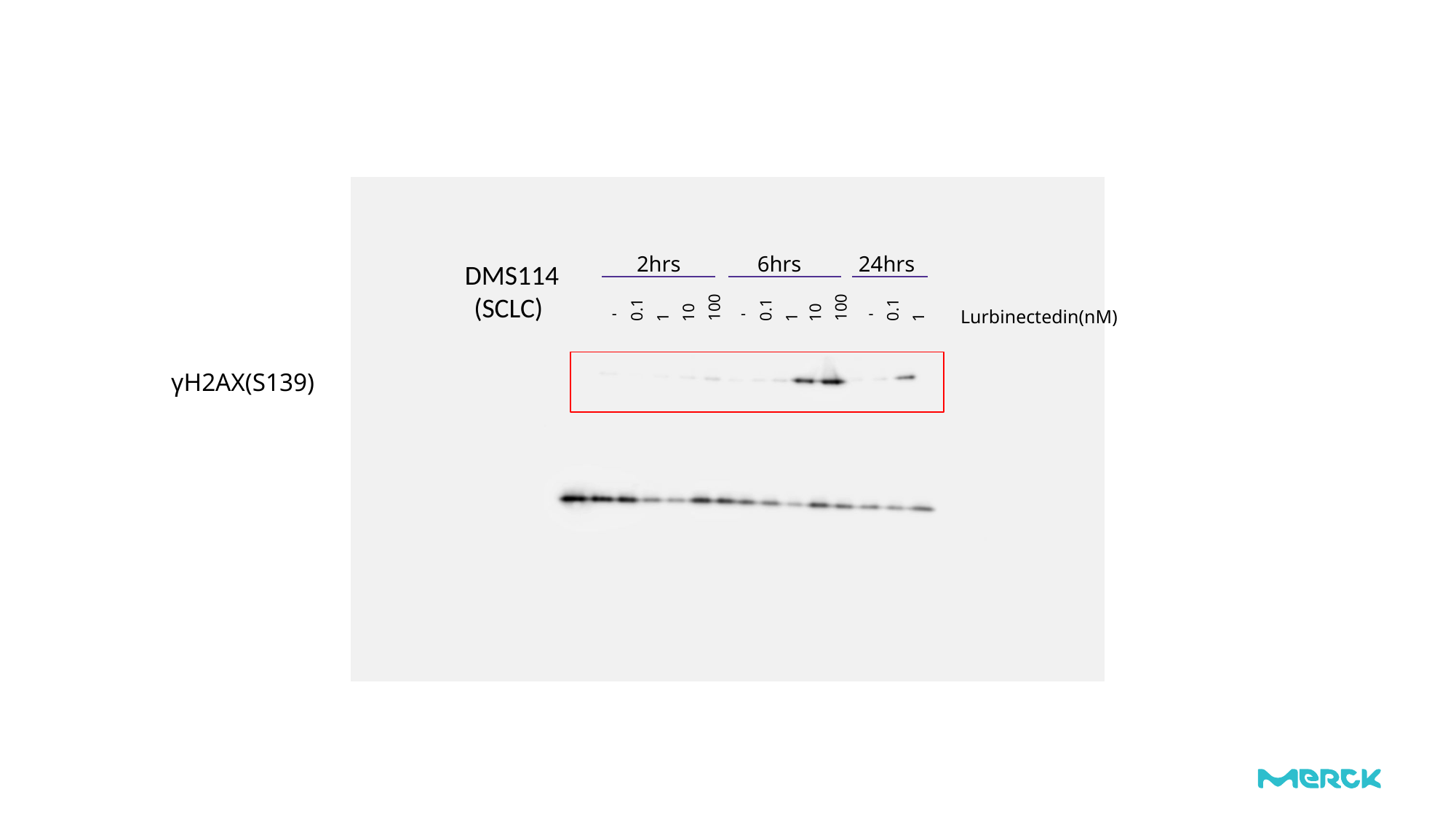

2hrs
6hrs
24hrs
DMS114
(SCLC)
100
100
0.1
0.1
0.1
Lurbinectedin(nM)
10
10
-
-
-
1
1
1
γH2AX(S139)

## Slide 12
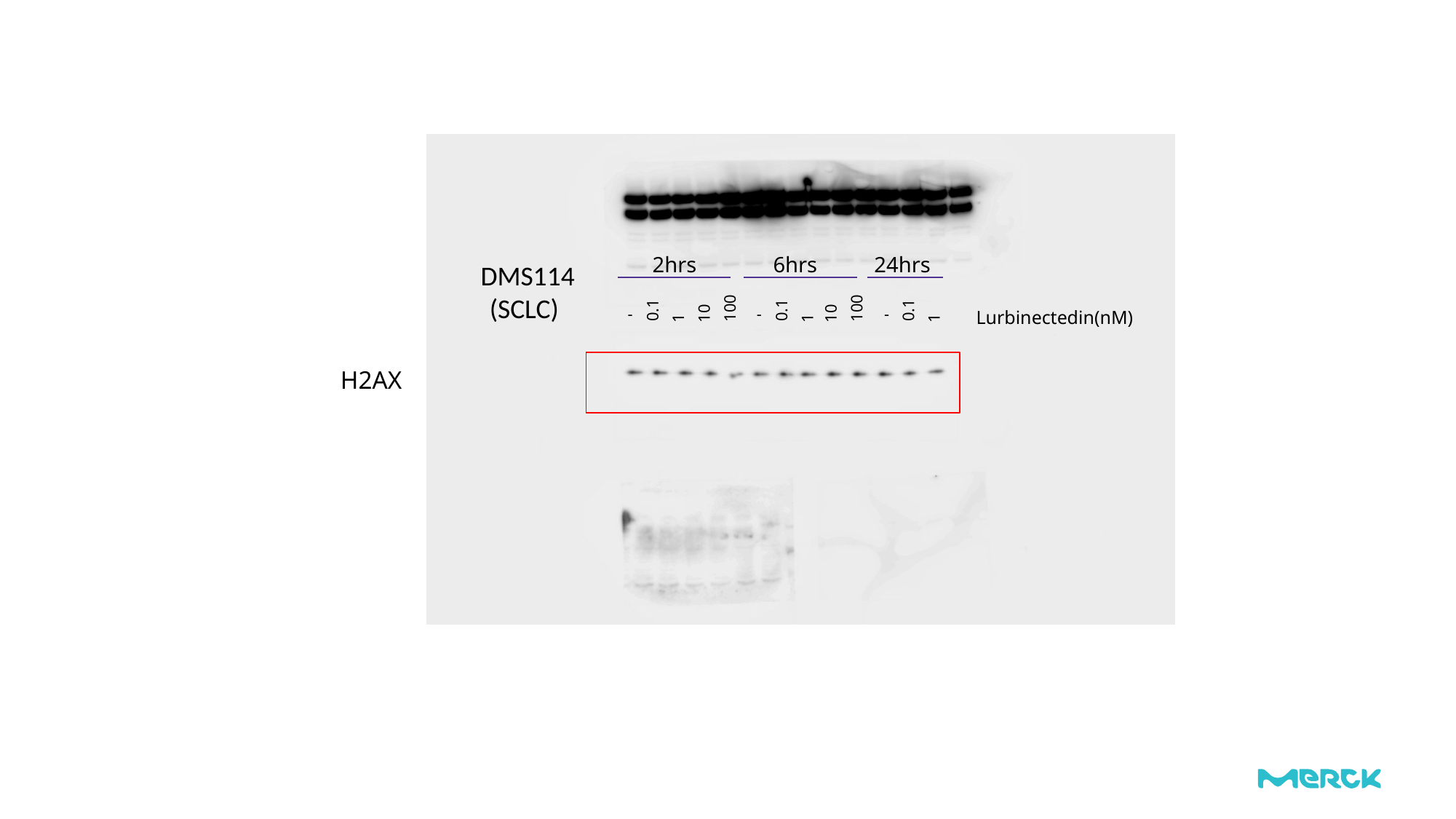

2hrs
6hrs
24hrs
DMS114
(SCLC)
100
100
0.1
0.1
0.1
Lurbinectedin(nM)
10
10
-
-
-
1
1
1
H2AX

## Slide 13
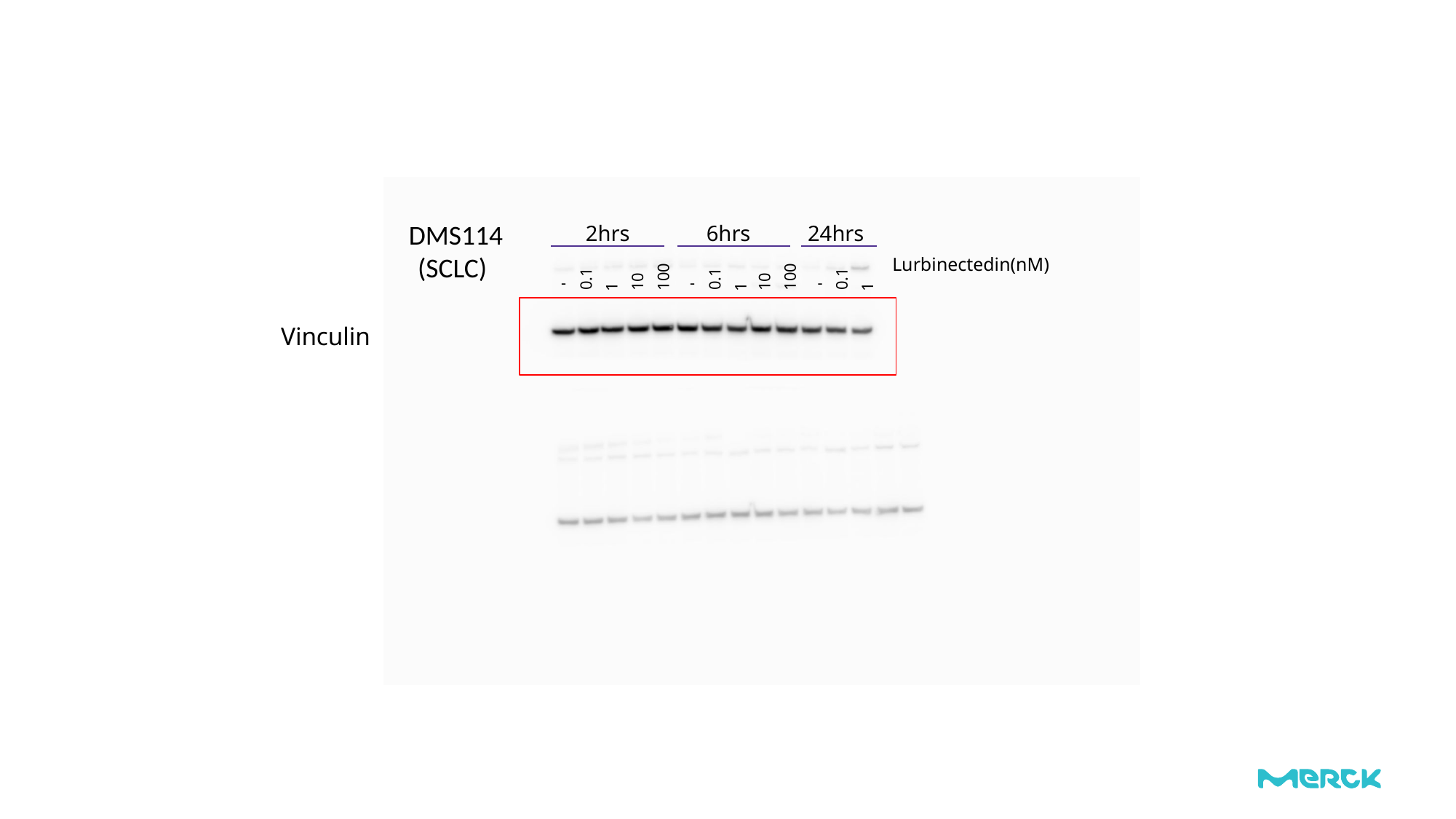

DMS114
(SCLC)
2hrs
6hrs
24hrs
Lurbinectedin(nM)
100
100
0.1
0.1
0.1
10
10
-
-
-
1
1
1
Vinculin
