# Supplementary material for: ATR inhibition augments the efficacy of lurbinectedin in small‐cell lung cancer
Source: EMBO Mol Med. 2023 Jul 25;15(8):e17313. doi: 10.15252/emmm.202217313 (PMC10405061; doi:10.15252/emmm.202217313)
Supplement: Supplementary file 7 — PDF+ [file EMMM-15-e17313-s003.pdf]

Article

SOURCE DATA TRANSPARENT PROCESS OPEN ACCESS

EMBO Molecular Medicine

ATR inhibition augments the efficacy of lurbinectedin in small-cell lung cancer

Christopher W Schultz[1], Yang Zhang[1], Rajaa Elmeskini[2], Astrid Zimmermann[3], Haiqing Fu[1], Yasuhisa Murai[1], Darawalee Wangsa[4], Suresh Kumar[1], Nobuyuki Takahashi[1,5], Devon Atkinson[2], Liton Kumar Saha[1], Chien-Fei Lee[6], Brian Elenbaas[6], Parth Desai[1], Robin Sebastian[1], Ajit Kumar Sharma[1], Melissa Abel[1], Brett Schroeder[1], Manan Krishnamurthy[1], Rajesh Kumar[1], Nitin Roper[1], Mirit Aladjem[1], Frank T Zenke[3], Zoe Weaver Ohler[2], Yves Pommier[1] & Anish Thomas[1,*]

Abstract

Small-cell lung cancer (SCLC) is the most lethal type of lung cancer. Specifically, MYC-driven non-neuroendocrine SCLC is particularly resistant to standard therapies. Lurbinectedin was recently approved for the treatment of relapsed SCLC, but combinatorial approaches are needed to increase the depth and duration of responses to lurbinectedin. Using high-throughput screens, we found inhibitors of ataxia telangiectasia mutated and rad3 related (ATR) as the most effective agents for augmenting lurbinectedin efficacy. First-in-class ATR inhibitor berzosertib synergized with lurbinectedin in multiple SCLC cell lines, organoid, and *in vivo* models. Mechanistically, ATR inhibition abrogated S-phase arrest induced by lurbinectedin and forced cell cycle progression causing mitotic catastrophe and cell death. High *CDKN1A*/p21 expression was associated with decreased synergy due to G1 arrest, while increased levels of *ERCC5*/XPG were predictive of increased combination efficacy. Importantly, MYC-driven non-neuroendocrine tumors which are resistant to first-line therapies show reduced *CDKN1A*/p21 expression and increased *ERCC5*/XPG indicating they are primed for response to lurbinectedin–berzosertib combination. The combination is being assessed in a clinical trial NCT04802174.

Keywords ATR inhibitor; biomarker; neuroendocrine; SCLC; synergy
Subject Categories Cancer; Respiratory System
DOI 10.15252/emmm.202217313 | Received 16 December 2022 | Revised 12 June 2023 | Accepted 19 June 2023 | Published online 25 July 2023
EMBO Mol Med (2023) 15: e17313

Introduction

Despite major advances in targeting oncogenes and the immune inhibitory checkpoints, most cancer patients die from chemotherapy-resistant disease. Multiple resistance mechanisms to chemotherapy have been described. For DNA-damaging chemotherapies, reduced intracellular drug intake, intracellular inactivation of the agent, increased DNA repair, activation of alternative DNA repair pathways, and impaired apoptotic signaling are among the most common resistance mechanisms (Housman *et al*, 2014).

Targeting DNA repair pathways using combination strategies represents a rational approach to overcome chemotherapy resistance. The ataxia telangiectasia–mutated and rad3-related (ATR) kinase is a master regulator of DNA damage response that plays a key role in stabilizing the genome when DNA replication is compromised (Ciccia & Elledge, 2010). ATR is activated by regions of single-stranded DNA (ssDNA), commonly generated as a result of DNA replication stress produced pharmacologically or by oncogene activation (Saldivar *et al*, 2018). Once activated, ATR functions to safeguard genomic integrity and safeguard replication by slowing the progression of replication forks, inhibiting distal replication origin firing, ensuring sufficient supply of deoxynucleotides, and promoting cell cycle arrest primarily by activation of intra-S and -G2/M cell cycle checkpoints (Saldivar *et al*, 2018). Accordingly, ATR inhibition leads to the loss of the S and G2/M checkpoints, allowing cells with damaged DNA to progress prematurely into M-phase, leading to mitotic catastrophe and cell death (Saldivar *et al*, 2018; Jo *et al*, 2021b). As such, multiple potent and selective ATR inhibitors are in preclinical and clinical development for

1 Developmental Therapeutics Branch, Center for Cancer Research, National Cancer Institute, National Institutes of Health, Bethesda, MD, USA
2 Center for Advanced Preclinical Research, Leidos Biomedical Research, Inc, Frederick National Laboratory for Cancer Research, Frederick, MD, USA
3 Translational Innovation Platform Oncology, Merck KGaA, Biopharma R&D, Darmstadt, Germany
4 Genetics Branch, Center for Cancer Research, National Cancer Institute, National Institutes of Health, Bethesda, MD, USA
5 Medical Oncology Branch, National Center for Global Health and Medicine, Tokyo, Japan
6 Translational Innovation Platform Oncology, EMD Serono Research and Development Institute Inc., Biopharma R&D, Billerica, MA, USA
*Corresponding author. Tel: +240 760 7343; E-mail: anish.thomas@nih.gov

© 2023 The Authors. Published under the terms of the CC BY 4.0 license.
This article has been contributed to by U.S. Government employees and their work is in the public domain in the USA.

cancer therapy (Thomas *et al*, 2018, 2021; Yap *et al*, 2020; Kim *et al*, 2021; Jo *et al*, 2021b).

SCLC is a neuroendocrine (NE) tumor characterized by near-universal bi-allelic loss of tumor suppressors *TP53* and *RB1*. *MYC* family genes are amplified, often on extrachromosomal DNA (ecDNA), in ~20% of SCLC and are overexpressed in ~50% of SCLC (George *et al*, 2015; Balanis *et al*, 2019; Pongor *et al*, 2023). Most patients are diagnosed with widely metastatic disease and are treated with a combination regimen of platinum, etoposide, and immunotherapy. Despite initial responses, risk of relapse is high with > 90% of patients progressing within 2 years (Paz-Ares *et al*, 2019). Second-line treatment options include topotecan and lurbinectedin, but the depth and duration of responses are modest and most relapsed tumors do not respond to additional chemotherapy. Furthermore, SCLCs have few targetable alterations (George *et al*, 2015) and tend not to respond to therapies targeted at somatic mutations (Lopez-Chavez *et al*, 2015). Recent studies have revealed heterogeneity of the SCLC neuroendocrine cell state, with tumors consisting of cells with NE and non-neuroendocrine (non-NE) features (Zhang *et al*, 2018). SCLC heterogeneity increases over the course of treatment, with an increase in chemoresistant non-NE cells evolving over time (Wagner *et al*, 2018; Ireland *et al*, 2020). Importantly, NE differentiation is emerging as a potential predictor of response to therapy (Rudin *et al*, 2019; Gay *et al*, 2021; Roper *et al*, 2021; Thomas *et al*, 2021).

Lurbinectedin is a synthetic alkylating derivative of trabectedin that was recently approved for SCLC patients with disease progression on or after platinum-based chemotherapy (Trigo *et al*, 2020). Lurbinectedin covalently binds to DNA-forming adducts that irreversibly stall elongating RNA polymerase II (Pol II) on the DNA template, generating DNA double-strand breaks (DSBs; Takebayashi *et al*, 2001; Santamaria Nunez *et al*, 2016). Here, we report an exquisite, NE differentiation-dependent synergistic interaction between the ATR inhibitor berzosertib and lurbinectedin. Mechanistically, lurbinectedin induces DSBs and activates ATR causing cell cycle arrest, allowing for repair and resistance, a process that is inhibited with berzosertib cotreatment. The combination produced greater synergy in MYC-high, chemoresistant, non-NE SCLC. Notably, these findings form the basis for a clinically actionable combination of a DNA repair inhibitor and a DNA-damaging agent to overcome SCLC chemoresistance.

Results

Drug screen identifies synergy of ATR inhibitors with lurbinectedin

To identify agents synergistically cytotoxic with lurbinectedin, we leveraged a previously reported drug screen in the SCLC cell line NCI-H446 wherein lurbinectedin was combined with 43 FDA-approved drugs or agents in late stages of clinical development (Thomas *et al*, 2021). The compound library included DNA-damaging agents and drugs that target mechanistically diverse pathways including DNA synthesis/metabolism, cell cycle or DNA damage repair, apoptosis, and chromatin remodeling (Fig 1A). Synergy was assessed using the highest single-agent (HSA) model (Berenbaum, 1989); positive values denote synergy and negative values antagonism. Lurbinectedin was most synergistic with DNA-damaging agents, drugs targeting cell cycle/DNA damage repair, and chromatin remodeling agents. In contrast, combinations with inhibitors of DNA synthesis and DNA metabolism were antagonistic, with the least synergy observed with the dihydrofolate reductase inhibitor pralatrexate (HSA −524.6) and the DNA polymerase inhibitor cytarabine (HSA −235.7; Table EV1).

Maximal cytotoxic synergy with lurbinectedin was observed for elimusertib (BAY1895344, HSA 881.2) and berzosertib (M6620, VX-970, VE-822, HSA 881.4) inhibitors of ATR, the main transducer of replication stress signaling and regulator of the cell cycle in response to DNA damage (Fig 1B and Table EV1). Both ATR inhibitors displayed greater synergy with lurbinectedin as compared to the topoisomerase II inhibitor doxorubicin (224.4 HSA, Table EV1). Doxorubicin was previously reported to be synergistic with lurbinectedin in preclinical models, however, this combination failed to improve survival compared with standard of care in patients with relapsed SCLC (Helwik, 2021). In a reciprocal screen, lurbinectedin was among the top four agents that showed maximal synergy with berzosertib (Fig 1C and Table EV2). The other top hits were inhibitors of key proteins involved in maintaining genomic stability including ataxia telangiectasia mutated (ATM, AZD-0156; Riches *et al*, 2020), WEE1 (MK-1775; Rajeshkumar *et al*, 2011), and topoisomerase I (TOP1; topotecan; Table EV2). The combination of topotecan and berzosertib is being examined in clinical trials (Thomas *et al*, 2018, 2021; NCT04768296, NCT03896503). Notably,

Figure 1. Lurbinectedin and berzosertib synergize in SCLC.

A A synergy screen was previously performed with 43 agents targeting multiple pathways in combination with each other. NCI-H446 SCLC cells were treated with drugs in a 10 × 10 matrix format, viability was assessed using Cell Titer Glo, and synergy was assessed using highest single-agent (HSA).

B Lurbinectedin synergy was ranked based on HSA synergy values. The highest synergy was observed with two ATR inhibitors, berzosertib and elimusertib, both of which displayed more synergy than doxorubicin.

C Berzosertib synergy with therapeutics ranked based on synergy. Topotecan and lurbinectedin both strongly synergized with berzosertib.

D, E Lurbinectedin and berzosertib synergized in SCLC cell lines NCI-H524 and DMS 114. Synergy of lurbinectedin and berzosertib was assessed by treating these drugs in a 10 × 10 matrix format for 72 h in NCI-H524 (D) (HSA 410.7) and DMS 114 cells (E) (HSA 286.3). Synergy was calculated by adding HSA across all combinations in the matrix (100 combinations, replicates = 3, $n = 1$). Synergy is denoted by blue and antagonism in red.

F Multiple DNA damage response pathways were activated by lurbinectedin. Treatment with the ATR inhibitor berzosertib specifically inhibited the activation of ATR and its downstream target CHK1 (both targets indicated by red box). DMS 114 cells were treated with lurbinectedin (1 nM) ± berzosertib (1 μM) for 6 or 24 h, and targets were assessed by immunoblotting.

G Lurbinectedin activated all three key DNA damage response proteins, DNA-PK, ATR, and ATM. Berzosertib is effective at inhibiting the activation of ATR and downstream ATR target CHK1 (indicated by red box), with less notable effects on other DNA damage repair pathways.

Source data are available online for this figure.

© 2023 The Authors

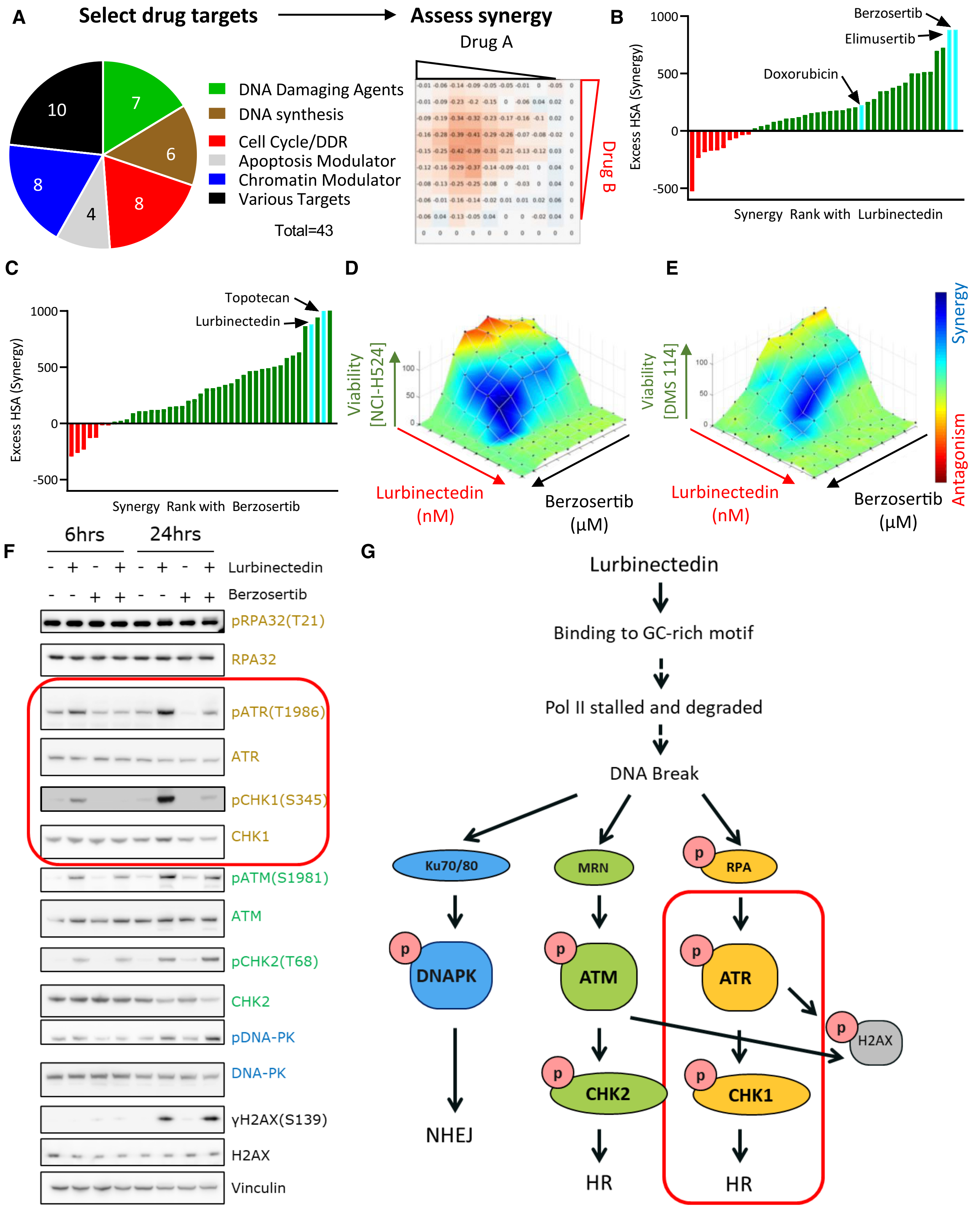
A
Select drug targets
Assess synergy
Drug A
Drug B
DNA Damaging Agents
DNA synthesis
Cell Cycle/DDR
Apoptosis Modulator
Chromatin Modulator
Various Targets
Total=43
B
Berzosertib
Elimusertib
Doxorubicin
Excess HSA (Synergy)
Synergy Rank with Lurbinectedin
C
Topotecan
Lurbinectedin
Excess HSA (Synergy)
Synergy Rank with Berzosertib
D
Viability [NCI-H524]
Lurbinectedin (nM)
Berzosertib (µM)
E
Viability [DMS 114]
Lurbinectedin (nM)
Berzosertib (µM)
Synergy
Antagonism
F
6hrs
24hrs
Lurbinectedin
Berzosertib
pRPA32(T21)
RPA32
pATR(T1986)
ATR
pCHK1(S345)
CHK1
pATM(S1981)
ATM
pCHK2(T68)
CHK2
pDNA-PK
DNA-PK
γH2AX(S139)
H2AX
Vinculin
G
Lurbinectedin
Binding to GC-rich motif
Pol II stalled and degraded
DNA Break
Ku70/80
MRN
RPA
DNAPK
ATM
ATR
H2AX
CHK2
CHK1
NHEJ
HR
HR

Figure 1.

lurbinectedin was more potent than topotecan in seven of nine SCLC cell lines tested and thus may have improved efficacy (Fig EV1A). The synergy of lurbinectedin and berzosertib was also confirmed in additional SCLC cell lines NCI-H524 (HSA 410.7) and DMS 114 (HSA 286.3; Fig 1D and E).

The combination of lurbinectedin and berzosertib causes mitotic catastrophe

Lurbinectedin treatment induced activation of ATR, ATM- and DNA-dependent protein kinase (DNA-PK), primary kinases that regulate DNA repair, and γH2AX, a marker of DNA DSBs (Blackford & Jackson, 2017; Fig EV1B). The addition of berzosertib reduced the activation of ATR and its downstream target CHK1, with less notable impact on ATM/CHK2 or DNA-PK (Fig 1F). These results confirm that lurbinectedin-induced DNA damage activates multiple DNA damage sensing and repair pathways, but berzosertib specifically reduces the activation of the ATR-CHK1 axis (Fig 1G).

Given the critical role of ATR and its downstream target CHK1 in initiating cell cycle arrest in response to DNA damage, we investigated whether the synergy of lurbinectedin and berzosertib was cell cycle dependent. Lurbinectedin-induced DNA damage as measured by γH2AX occurred predominantly in the S-phase of cycling cells (Figs 2A and EV1C). Berzosertib alone or in combination with lurbinectedin reduced γH2AX in the majority of cells, but in a small fraction (~5%) of the population caused a drastic increase in γH2AX. Therefore, when assessed using flow cytometry, cotreatment with berzosertib reduced lurbinectedin-dependent S-phase γH2AX induction in the majority of cells leading to a decrease in median γH2AX signal in S-phase cells, but consistent with our immunoblotting (Fig 1F) caused an overall increase in average γH2AX signal in DMS 114 cells (Figs 2B and EV1D–F). We assessed γH2AX induction using immunostaining and immunoblotting in additional cell lines and found berzosertib overall inhibited lurbinectedin-dependent γH2AX induction (Fig 2C and Appendix Fig S1A–D). Together, the immunoblotting, immunostaining, and flow cytometry analyses indicated that berzosertib cotreatment reduces lurbinectedin-induced γH2AX formation. Reduced γH2AX signal in the majority of cells was not due to a decrease in DNA damage as the combination of lurbinectedin and berzosertib induced more DNA breaks as assessed by alkaline comet assay than either agent alone (Fig 2D and E). Treatment with lurbinectedin reduced DNA replication as indicated by a decrease in 5-ethynyl-2′-deoxyuridine (EdU) incorporation, an effect which was largely rescued by treatment with berzosertib (Figs 2F and EV1G). Lurbinectedin-induced increase in γH2AX and decrease in EdU incorporation were inhibited by siRNA against ATR, suggesting that these responses are indeed ATR dependent (Fig EV1H and I).

Next, we used molecular combing (DNA fiber assays) to visualize single replicons and measure the impact of lurbinectedin and ATR inhibition on replication dynamics. Control DMS 114 cells displayed roughly equal distribution of initiation (27%), termination (39%), and unidirectional (34%) forks. Lurbinectedin treatment suppressed replication fork initiation by 3-fold while slightly increasing termination and unidirectional forks (1.2-fold and 1.3-fold, respectively). These results were in agreement with our previous data indicating lurbinectedin treatment inhibited DNA synthesis. Berzosertib treatment increased initiation forks by 1.8-fold compared with control, consistent with previous work demonstrating that ATR inhibition causes the unscheduled firing of dormant origins (Jo *et al*, 2021b) with a minimal impact on termination and unidirectional forks. In combination, berzosertib treatment rescued

Figure 2. Berzosertib causes continued cell cycle progression and induces mitotic catastrophe in the presence of lurbinectedin.

A Lurbinectedin induced changes to γH2AX across different phases of the cell cycle, with the greatest degree of activation in S-phase. DMS 114 cells were treated with 1 nM lurbinectedin for 1 to 6 h and γH2AX induction was assessed using flow cytometry; four replicates of 10,000 cells for each timepoint were assessed, and error bars represent SEM, $n = 2$. Cell cycle was assessed using propidium iodide.

B Lurbinectedin treatment increased γH2AX induction, while berzosertib treatment caused a small portion of cells to display increased γH2AX signal. Berzosertib cotreatment with lurbinectedin caused a decrease in γH2AX accumulation for the majority of cells leading to a decreased median accumulation of γH2AX, however, an increase in γH2AX in a smaller portion of cells led to an increased average accumulation (averages and medians are shown in Fig EV1D–F). DMS 114 cells were treated with ±1 nM lurbinectedin ±2 μM berzosertib for 6 h γH2AX induction was assessed using flow cytometry. 10 mM EdU was added for the last hour prior to collection (assessed Fig 2F). Numbers on the graph indicate the average percent of cells across four replicates of 10,000 cells with low, medium, or high γH2AX, $n = 3$.

C Lurbinectedin treatment increased γH2AX induction, which was reduced with berzosertib treatment. γH2AX induction was assessed using immunofluorescence in DMS 114 cells were treated with ±1 nM lurbinectedin ±2 μM berzosertib for 6 h. Quantification is from 100 to 150 cells per treatment, with error bars indicating SEM, comparisons were made using an unpaired two-tailed Student's *t*-test in PRISM, $n = 3$. Representative images for this experiment are in Appendix Fig S2A.

D, E Combination of lurbinectedin and berzosertib caused DNA damage. DMS 114 cells were treated with ±1 nM lurbinectedin ±2 μM berzosertib for 6 h and total DNA damage was assessed using an alkaline comet assay. These data represent average tail length for each group with 150–250 cells measured per group, $n = 3$ error bars represent SEM, *P*-values represent unpaired two-tailed Student's *t*-tests performed in PRISM.

F Lurbinectedin treatment caused a decrease in DNA replication as assessed by EdU incorporation while berzosertib cotreatment largely rescued this phenotype. Numbers represent the average percent of cells across four replicates with high EdU incorporation indicating functional DNA replication, $n = 3$. These cells are the same as assessed in Fig 2B.

G Lurbinectedin treatment inhibited fork initiation, an effect that was rescued by the addition of berzosertib. DMS 114 cells were treated with ±1 nM lurbinectedin ±2 μM berzosertib for 6 h, and using DNA combing, we assessed motifs of incorporation of IdU and CldU indicating initiation, termination, or unidirectional DNA forks. Forks were assessed and verified individually to avoid algorithmic assessment, with 100–200 forks quantified per group $n = 2$.

H, I Berzosertib treatment alone caused a small portion of cells to undergo mitotic catastrophe, however, cotreatment of lurbinectedin and berzosertib caused a large portion of cells to undergo mitotic catastrophe. DMS 114 cells were treated with ±1 nM lurbinectedin ±2 μM berzosertib for 6 h with the final 3 h being in the presence of nocodazole followed by release from all drugs and 45 min of continued growth. Cells that had undergone mitotic catastrophe were assessed as those which were multinucleated. Representative images (H) and quantification of percent of cells that underwent mitotic catastrophe (I). Graph represents average of three separate experiments with statistics determined from assessing 100 nuclei per group, error bars represent SEM, and *P*-values are indicative of unpaired two-tailed Student's *t*-tests performed in PRISM.

Source data are available online for this figure.

 © 2023 The Authors

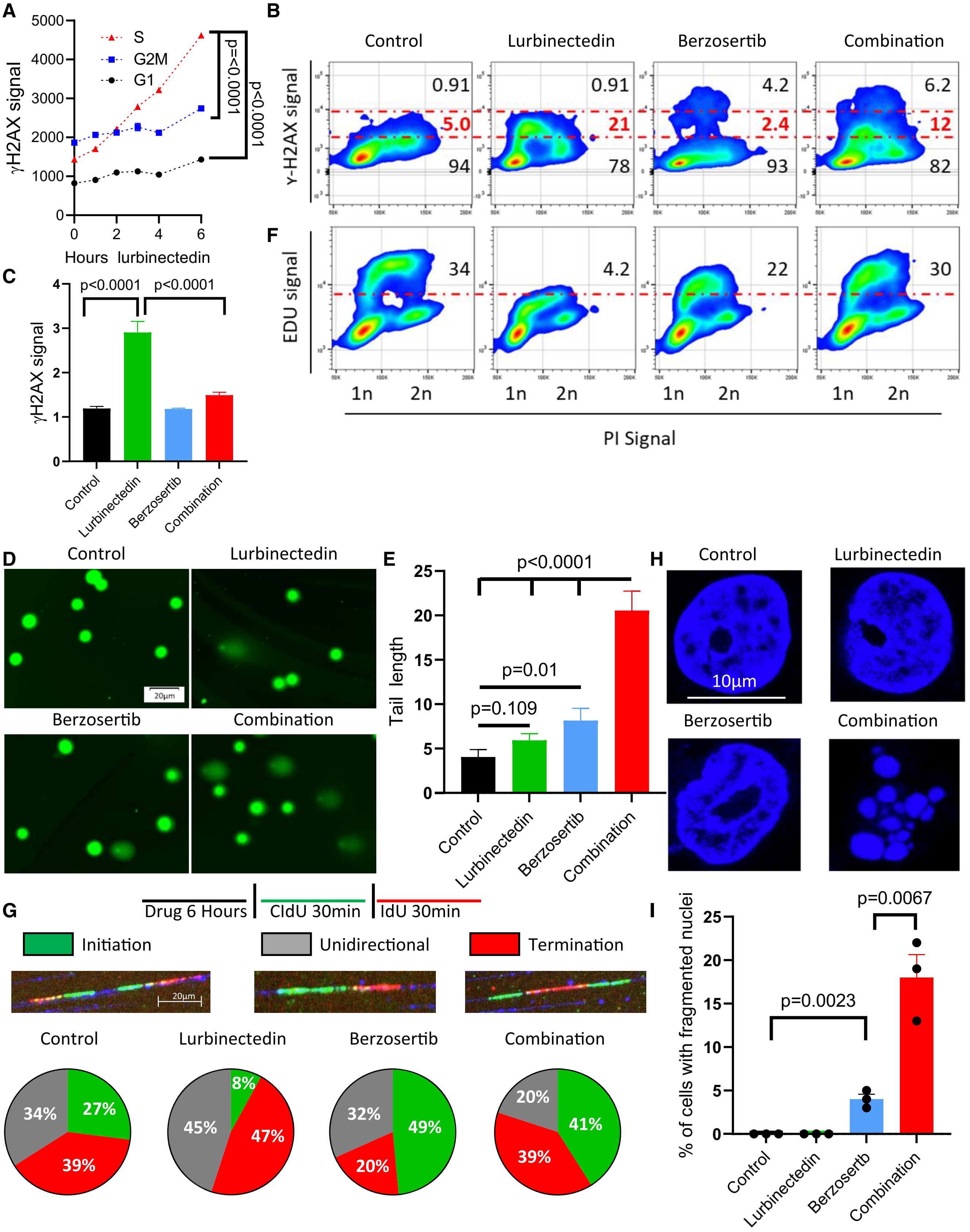
A
γH2AX signal
S
G2M
G1
p=<0.0001
p<0.0001
Hours lurbinectedin
B
Control
Lurbinectedin
Berzosertib
Combination
γ-H2AX signal
0.91 5.0 94
0.91 21 78
4.2 2.4 93
6.2 12 82
F
EDU signal
34
4.2
22
30
1n 2n
PI Signal
C
p<0.0001 p<0.0001
γH2AX signal
Control
Lurbinectedin
Berzosertib
Combination
D
Control
Lurbinectedin
Berzosertib
Combination
20µm
E
p<0.0001
p=0.01
p=0.109
Tail length
Control
Lurbinectedin
Berzosertib
Combination
H
Control
Lurbinectedin
10µm
Berzosertib
Combination
G
Drug 6 Hours
CldU 30min
IdU 30min
Initiation
Unidirectional
Termination
20µm
Control
Lurbinectedin
Berzosertib
Combination
34% 27% 39%
45% 8% 47%
32% 49% 20%
20% 41% 39%
I
p=0.0067
p=0.0023
% of cells with fragmented nuclei
Control
Lurbinectedin
Berzosertb
Combination

Figure 2.

the suppression of replication fork initiation caused by lurbinectedin, with a 5-fold increase in initiation forks as compared to lurbinectedin treatment, also reducing termination and unidirectional forks to a lesser extent (0.83-fold and 0.44-fold, respectively; Fig 2G; Iyer & Rhind, 2017). These results demonstrated that berzosertib leads to unscheduled replication origin firing and continued DNA replication even in the presence of lurbinectedin-induced DNA damage.

In addition to its crucial role in DNA damage and replication stress response, ATR also promotes accurate chromosome segregation during mitosis (Blackford & Jackson, 2017). We assessed metaphase spreads to determine drug treatment-induced changes to chromosomal integrity during mitosis. Berzosertib monotherapy led to abnormal metaphase spreads, with individual chromosomes failing to segregate appropriately (Appendix Fig S1E and F). This phenotype was recapitulated on treatment with barasertib, an inhibitor of Aurora Kinase B, a downstream target of ATR critical for accurate chromosomal segregation (Appendix Fig S1G). Lurbinectedin alone had little effect on chromosomal integrity at metaphase. Lurbinectedin–berzosertib combination treatment did not impact the frequency of segregation defects caused by berzosertib treatment, but the combination significantly increased the percentage of mitotic cells with multiple breaks in chromosomes (Appendix Fig S1E). We assessed postmitotic viability in order to determine if the chromosome breaks induced by cotreatment affected mitotic competence. Both lurbinectedin and berzosertib had little effect on postmitotic viability by themselves. The combination, however, significantly increased postmitotic death as indicated by multinucleation (Fig 2H and I). Together, these results demonstrate that berzosertib augments DNA damage caused by lurbinectedin and allows cells to progress to mitosis with unrepaired DNA damage, ultimately leading to mitotic catastrophe and cell death.

ERCC5/XPG and SLFN11 are critical determinants of response to lurbinectedin

Similar to the structurally related trabectedin, adducts generated by binding lurbinectedin to the DNA minor groove are recognized by the nucleotide excision repair (NER) pathway. Lurbinectedin then binds NER complex member *ERCC5*/XPG, trapping the complex on the DNA, ultimately leading to the formation of irreversible single-strand breaks, and consequently cell death (Takebayashi *et al*, 2001; Romano *et al*, 2013). Homologous recombination (HR) is critical for the repair of lurbinectedin-induced DNA damage, and loss of the HR pathway increases sensitivity to lurbinectedin (Romano *et al*, 2013). Isogeneic models using the chicken B cell line DT40 cells have previously been utilized to demonstrate the importance of NER and HR pathways for lurbinectedin efficacy (Romano *et al*, 2013). We examined the contribution of these DNA repair mechanisms to the efficacy of lurbinectedin, berzosertib, and the combination utilizing isogenic DT40 models. Consistent with previous findings, DT40 cells with knockout (KO) of the NER pathway mediators *ERCC5*/XPG and XPA were approximately 10-fold more resistant to lurbinectedin. Alternatively, *BRCA2*-KO DT40 cells were approximately 6-fold more sensitive to lurbinectedin (Fig EV2A). Berzosertib efficacy was not significantly altered in the knockouts (Fig EV2B).

ATR signaling and ATR-induced cell cycle arrest are critical for efficient HR repair (Blackford & Jackson, 2017). To confirm that berzosertib was inhibiting HR competency, we utilized U2OS cells with a stably integrated GFP HR reporter (Weinstock *et al*, 2006). We confirmed that berzosertib inhibited HR competency at 7-fold lower concentrations than that required for inhibiting viability (Fig EV2C and D). If lurbinectedin-berzosertib synergy was due to reduced HR competency from berzosertib, synergy would be decreased in the *BRCA2*-KO. Supporting this hypothesis, *BRCA2*-KO cells displayed slightly decreased synergy. Consistent with the importance of the NER pathway for lurbinectedin efficacy, synergy was even more markedly reduced with *XPG*-KO and *XPA*-KO cells (Fig EV2E). To standardize the assessment of overall combination efficacy, we defined lurbinectedin IC_{50} in the presence of 500 nM of berzosertib (a concentration where lurbinectedin IC_{50}s are reduced in all synergistic models tested and HR is inhibited) as representative of overall combination efficacy. Lurbinectedin–berzosertib combination displayed similar combination efficacy in the *BRCA*-2 KO cell line as compared to control. However, the *XPG*-KO and *XPA*-KO cell lines maintained resistance even in the presence of 500 nM berzosertib leading to reduced combination efficacy (Fig EV2F). These results show that lurbinectedin–berzosertib synergy is only partially mediated by berzosertib-inhibiting HR competency, and that the efficacy of lurbinectedin alone and combination efficacy are both dependent on NER competency.

A recent report has indicated that cells with high expression of SLFN11, another important mediator of DNA damage response, are significantly more sensitive to lurbinectedin (Kundu *et al*, 2021). SLFN11 destabilizes paused replication forks following DNA damage causing cell death. Consequently, loss of SLFN11 leads to broad resistance to a variety of DNA-damaging therapeutics (Jo *et al*, 2021a). We found that SLFN11-KO DMS 114 cells were approximately 4-fold more resistant to lurbinectedin than parental cells, while there was little difference in sensitivity to berzosertib (Fig EV2G and H). Consistent with previous findings demonstrating the ability of ATR inhibition to re-sensitize SLFN11-KO cells to DNA-damaging agents (Jo *et al*, 2021a), synergy was increased in SLFN11-KO cells, and SLFN11-KO cells displayed similar combination efficacy as the control cells (Fig EV2I and J). As berzosertib rescued lurbinectedin efficacy in SLFN11-KO cells and not in XPG-KO cells, we conclude that *ERCC5*/XPG is mechanistically required for lurbinectedin activity even in the presence of an ATR inhibitor, whereas SLFN11 is not. These results are consistent with SLFN11 inducing lethal replication fork instability in response to DNA-damaging agents independently of ATR and acting in parallel with the ATR-mediated S-phase checkpoint (Murai *et al*, 2016).

Next, we assessed the efficacy of lurbinectedin, berzosertib, and the combination of both agents in a 10 × 10 matrix format (100 conditions) across a panel of nine SCLC cell lines spanning the spectrum of NE differentiation and lineage transcription factors (Table 1; Rudin *et al*, 2019). Lurbinectedin was highly potent across SCLC cell lines with IC_{50}s ranging from 0.01 to 0.38 nM, while berzosertib IC_{50}s ranged from 0.34 to 5.04 μM (Table 1). Consistent with findings in isogenic models described above, we observed trends toward increased sensitivity to lurbinectedin in cells with high *SLFN11* or *ERCC5*/XPG expression (Fig EV3A). We assessed the IC_{50} of lurbinectedin in the presence of 500 nM berzosertib, a concentration that effectively inhibits HR, and has little effect on viability by itself in most models. Increased *ERCC5*/XPG expression significantly correlated with improved combination efficacy, while higher *SLFN11*

 © 2023 The Authors

Table 1. Critical variables for lurbinectedin and berzosertib efficacy and synergy in SCLC cell lines.

Cell line	p53 mutation status	RB mutation status	SCLC subtype	NE score	Excess HSA (Synergy)	CDKN1A (RNA)	MYC (RNA)	ERCC5 (RNA)	SLFN11 (RNA)	BRZ IC50 (μM)	Lurb IC50 (nM)	Lurb + 500 nM BRZ IC50 (nM)
NCI-H211	Mutated	Wild-type	POU2F3	−0.699	474.0	7.6	10.8	7.1	6.116	1.001	0.099	0.039
NCI-H524	Mutated	Mutated	NEUROD1	0.264	410.7	7.1	11.2	7.1	4.427	1.270	0.215	0.059
DMS 114	Mutated	Wild-type	YAP1	−0.587	286.3	7.5	9.3	8.1	7.810	0.338	0.157	0.058
NCI-H841	Mutated	Wild-type	YAP1	−0.638	257.1	8.0	9.5	6.0	4.937	5.036	0.383	0.172
NCI-H1048	Mutated	Mutated	POU2F3	−0.699	250.1	8.4	9.0	7.5	8.929	0.434	0.010	0.005
NCI-H1341	Wild-type	Wild-type	YAP1	−0.333	180.6	9.0	10.7	6.9	7.839	1.053	0.123	0.063
NCI-H446	Mutated	Mutated	NEUROD1	0.000	−11.9	9.1	10.4	6.8	4.692	0.507	0.089	0.059
NCI-H146	Wild-type	Mutated	ASCL1	0.395	−128.3	8.5	9.5	7.7	4.625	3.213	0.143	0.078
NCI-H889	Mutated	Wild-type	ASCL1	0.479	−171.3	9.3	5.8	6.0	4.679	2.208	0.108	0.179

Pertinent values from the nine SCLC cell lines utilized in this work. All RNA values are given as log 2 values and these along with NE scores, and RB (RB1 and RB2 both assessed, all mutations in RB1) and p53 mutation status are publicly available at https://discover.nci.nih.gov/rsconnect/cellminercdb.

expression trended toward increased combination efficacy (Figs 3A and EV3A, Table 1 and Table EV3). Overall, studies in isogenic systems and human SCLC cell lines demonstrate that SLFN11, HR, and *ERCC5*/XPG are determinants for the response to lurbinectedin monotherapy, with *ERCC5*/XPG being a critical determinant of response to the lurbinectedin–berzosertib combination, that is, combination efficacy (Table EV3 and Fig EV3B).

The G1/S checkpoint is a critical determinant of lurbinectedin–berzosertib synergy; consequently, *CDKN1A*/p21 is a determinant of reduced synergy

The lurbinectedin–berzosertib combination was synergistically cytotoxic in six of nine SCLC cell lines assessed (HSA 180.5 to 474.0) and additive (HSA −11.9 where 0 is additive) or antagonistic (HSA −128.3 to −171.3) in the others (Table 1). Synergy, that is, HSA or the difference between lurbinectedin IC_{50}s +/− berzosertib, did not correlate with sensitivity to either drug or to the expression of *SLFN11* or *ERCC5*/XPG (Fig EV3A). As lurbinectedin and berzosertib greatly affected DNA replication and mitotic division, we assessed whether synergy was dependent on cell cycle dynamics. Cell lines with higher synergy (NCI-H841 and NCI-H1341) upon exposure to lurbinectedin had significantly greater S-phase arrest which was ameliorated with cotreatment with berzosertib as compared to less synergistic cell lines (NCI-H146 and NCI-H889; Figs 3B and C, and EV3C). Therefore, we hypothesized that cells deficient in the G1/S checkpoint could be uniquely sensitive to the combination of lurbinectedin–berzosertib.

SCLC is characterized by loss of RB1 (George *et al*, 2015), the predominant regulator of G1/S transition leading to increased reliance on cyclin-dependent kinase inhibitor p21 (*CDKN1A*) for control of the G1/S checkpoint (Hauge *et al*, 2019). High expression of *CDKN1A* RNA was associated with reduced lurbinectedin–berzosertib synergy (Fig 3D, and Table 1 and Table EV3). Furthermore, p21 protein expression, which was highly correlated with *CDKN1A* RNA expression (Fig EV3D), was also associated with decreased synergy (Fig 3E and F). Supporting our hypothesis that p21-mediated G1 arrest could lead to reduced sensitivity to lurbinectedin–berzosertib combination, thymidine-enforced G1 cell cycle arrest led to a significant reduction in efficacy of the combination (Fig EV3E). Small interfering RNA (siRNA) knockdown of p21 in the least synergistic cell line NCI-H889 resulted in a significant increase in synergy (Fig 3G), while overexpression of p21 in the synergistic DMS 114 cell line led to a decrease in synergy (Fig EV3F). These data are consistent with previous work in which p21 levels predicted reduced sensitivity to agents targeting downstream targets of ATR, CHK1, and Wee1 (Hauge *et al*, 2019).

ATM-dependent activation of p53 followed by p53-dependent p21 upregulation is a canonical pathway that regulates p21 expression in response to DNA damage (Smith *et al*, 2020). *TP53*/p53 is mutated in most SCLC tumors (George *et al*, 2015), suggesting frequent abrogation of the ATM-p53-p21 axis in SCLC. To determine the impact of the ATM-p53-p21 pathway on lurbinectedin–berzosertib synergy, we utilized two cell lines that exhibited low synergy and high expression of *CDKN1A* which were *TP53* mutated (NCI-H889) or *TP53* intact (NCI-H146). Upon lurbinectedin treatment, p21 protein levels were reduced in NCI-H889 cells, whereas NCI-H146 cells displayed an increase in p21 (Fig EV4A). P21 decrease in NCI-H889 cells is expected as lurbinectedin inhibits RNA Pol-II and, due to the rapid turnover rate of p21 protein and RNA, inhibition of RNA Pol-II causes rapid decreases in p21 (Al-Haj *et al*, 2012). Increased p21 in NCI-H146 cells is consistent with the ability of both DNA-damaging agents and transcription inhibitors to increase p21 expression in an ATM-p53-p21 axis-dependent manner (Smith *et al*, 2020). Consistent with the importance of ATM in the *TP53*-intact setting, addition of the ATM inhibitor KU60019 (Golding *et al*, 2009) significantly increased the synergy of lurbinectedin–berzosertib in NCI-H146 cells. Conversely, in NCI-H889 cells, ATM inhibition was less effective at increasing synergy (Fig EV4B and C).

Together, our *in vitro* data show that high *CDKN1A*/P21 expression irrespective of *TP53* status predicts decreased synergy of lurbinectedin–berzosertib, that is, less impact on lurbinectedin IC_{50}s with the addition of berzosertib, while higher *ERCC5*/XPG expression predicts greater sensitivity, that is, decreased lurbinectedin IC_{50}s in the presence of berzosertib. We confirmed these results in two small-cell organoid models (Fig EV4D–G). We propose

© 2023 The Authors

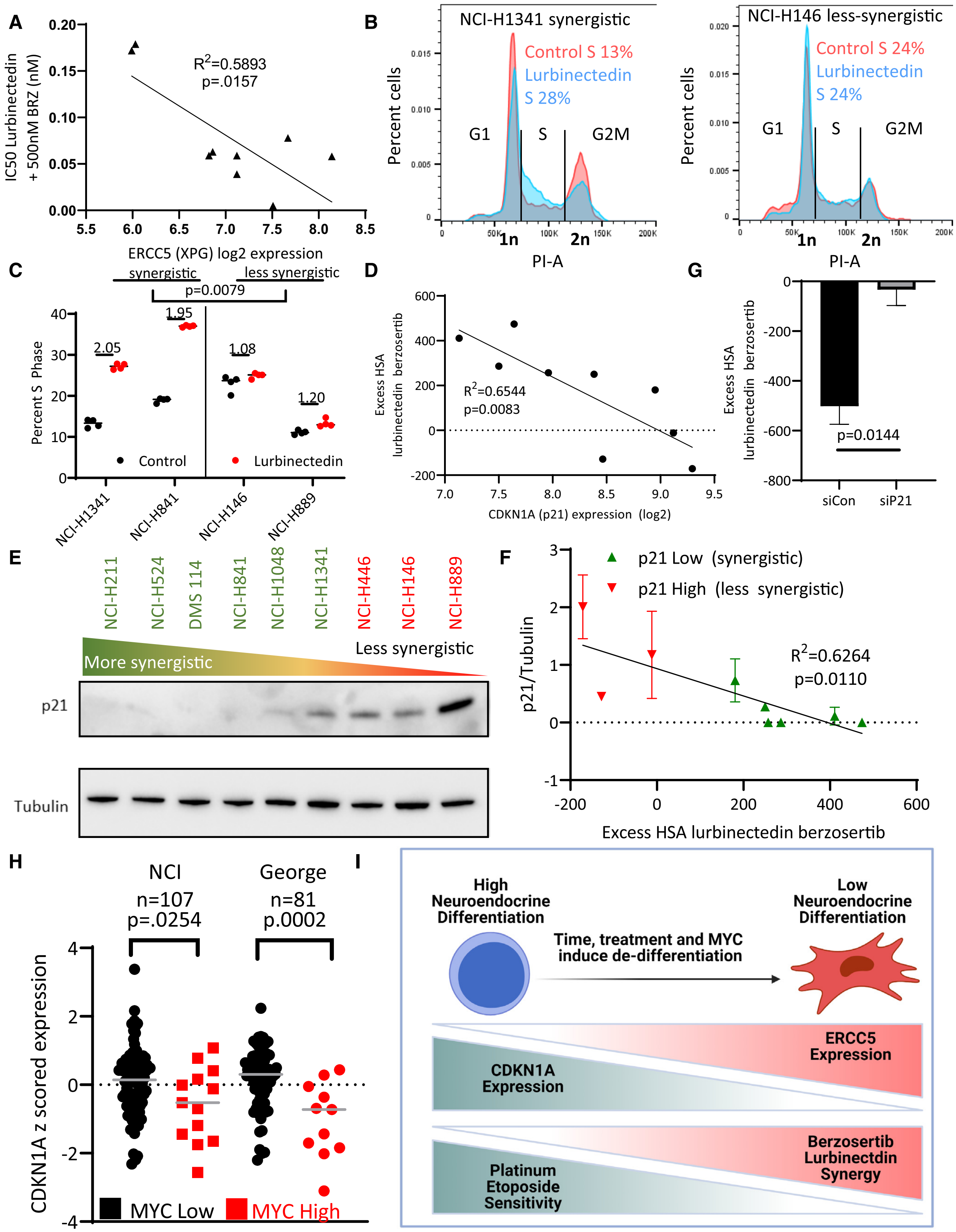

Figure 3.

© 2023 The Authors

◀ **Figure 3. p21 inhibits lurbinectedin and berzosertib synergy, with greater synergy expected in recalcitrant MYC-driven non-NE cells.**

A *ERCC5* (XPG) expression inversely correlated with combination efficacy, that is, when the expression of *ERCC5* (XPG) was high, the combination was more effective. The IC_{50} of lurbinectedin in the presence of 500 nM berzosertib (combination efficacy) was determined in nine cell lines after assessing all nine lines using a 10 × 10 matrix of lurbinectedin and berzosertib combinations (replicates = 3, *n* = 1); Pearson correlation was assessed in PRISM.

B, C Lurbinectedin increased S-phase arrest as assessed by propidium iodide staining. Synergistic cell lines had a greater increase in S-phase than less synergistic lines. Synergistic (NCI-H841 and NCI-H1341) and less synergistic (NCI-H146 and NCI-H889) cell lines were treated with ±1 nM lurbinectedin ±2 μM berzosertib. Quantification in (C) is representative of four technical replicates of 10,000 cells per group biological replicates, *n* = 3, with fold change in S-phase cells being compared between synergistic and less synergistic cell lines using an unpaired two-tailed Student's *t*-test in PRISM. These data are also described in Appendix Fig S4C.

D *CDKN1A* (p21) RNA expression inversely correlated with synergy of lurbinectedin and berzosertib, indicating that *CDKN1A* (p21) could potentially inhibit synergy. Pearson correlation was assessed in PRISM.

E, F Synergy was lower in cells that displayed higher expression of p21 protein. (E) Cell lines were ordered by HSA synergy score (most-to-least synergy, left to right), and p21 protein expression was assessed by immunoblotting. (F) Quantitation of p21 protein compared to control tubulin across two biological replicates, error bars represent SD; Pearson correlation was assessed in PRISM.

G Knockdown of CDKN1A (p21) in NCI-H889 cells led to increased synergy. NCI-H889 cells were treated with siRNA against control or *CDKN1A* (p21), followed by dosing in a 10 × 10 matrix format in triplicate with lurbinectedin and berzosertib and collection after 72 h. HSA synergy was determined and summed across the 10 × 10 matrix, graph represents the average of three independent experiments of 10 × 10 matrixes in triplicate (technical replicates), error bars = SD, and unpaired two-tailed Student's *t*-test performed in PRISM.

H High MYC family member patient samples had lower CDKN1A (p21) expression consistent with high MYC family member expression causing decrease in CDKN1A (p21). In two independent SCLC datasets, *MYC*, *MYCL*, and *MYCN* expressions were z-scored (within each database) and the max MYC family member z-score expression was determined for each sample. Those samples which were greater than 1 SD above average were considered to be high MYC family member expressing. *P*-values are indicative of unpaired two-tailed Student's *t*-test assessed in PRISM.

I As SCLC progresses, cancer cells progress from a NE-differentiated state to a non-NE state. These non-NE cells have a lower expression of *CDKN1A* (p21) and a higher expression of *ERCC5* (XPG). These are characteristics that make them less responsive to the standard first-line platinum/etoposide regimen, however, this should make them more sensitive to the combination of lurbinectedin and berzosertib.

Source data are available online for this figure.

CDKN1A/P21 as the primary negative determinant of synergy for lurbinectedin–berzosertib while *ERCC5*/XPG expression is required for lurbinectedin-induced DNA damage and determines combination efficacy (Table EV3).

MYC expression and non-NE differentiation are associated with lurbinectedin–berzosertib synergy

SCLC is characterized by NE differentiation which decreases as tumors progress and following chemotherapy (Wagner *et al*, 2018; Ireland *et al*, 2020). NE differentiation as characterized by a previously validated 50-gene signature (Zhang *et al*, 2018) negatively associated with lurbinectedin–berzosertib synergy, with the highest synergy observed in non-NE SCLC cells (Fig EV4H). MYC, as a driver of both non-NE differentiation and decreased *CDKN1A* expression (Fiorentino *et al*, 2016; Ireland *et al*, 2020), could potentially cause the increased synergy of lurbinectedin–berzosertib in non-NE cells. In metastatic small-cell patient tumors, *MYC* expression was negatively correlated with NE differentiation (Appendix Fig S2A). In our metastatic dataset along with additional primary SCLC patient tumor, circulating tumor cell-derived xenograft, and patient-derived xenograft (PDX) datasets, samples overexpressing *MYC* paralogs (*MYC*, *MYCL*, or *MYCN*) consistently displayed significantly lower *CDKN1A* expression (Fig 3H and Appendix Fig S2B). Similar results were observed in SCLC cell lines where *MYC* expression negatively correlated with *CDKN1A* expression and NE differentiation (Appendix Fig S2C and D).

Confirming the recalcitrance of high MYC non-NE SCLC tumors (Alves Rde *et al*, 2014), of 50 hallmark gene sets, we found that the 2 gene sets predictive of MYC activity, MYC-targets-V1 and MYC-targets-V2, were, respectively, the first and third most highly associated with platinum resistance in SCLC patients (Horita *et al*, 2015; Liberzon *et al*, 2015; Appendix Fig S2E). MYC-targets-V1 significantly differentiated platinum/etoposide sensitivity (Appendix Fig S2F and G), with MYC-low patients displaying hazard ratios of 0.2130 and 0.1830 at the median and quartile levels, respectively, for platinum resistance (response duration of < 90 days to carboplatin etoposide therapy). These data indicate that MYC-low SCLCs were ~5-fold less likely to be resistant to carboplatin/etoposide therapy as compared to MYC-high tumors. Interestingly, SCLC cell lines with enrichment of MYC-targets-V2 were associated with increased lurbinectedin–berzosertib combination efficacy (Appendix Fig S2H). NE differentiation negatively correlated with *ERCC5*/XPG expression in multiple clinical datasets, with non-NE tumors displaying higher *ERCC5*/XPG expression (Appendix Fig S2I and J). Together, these data indicate that MYC-driven, non-NE tumors are resistant to platinum-based chemotherapy, but they may be uniquely sensitive to lurbinectedin–berzosertib combination due to high *ERCC5*/XPG and low *CDKN1A*/p21 expression (Fig 3I and Table EV3).

In vivo synergy and schedule dependence of lurbinectedin–berzosertib combination

In our previous work and ongoing clinical trials, we have observed efficacy using a dosing regimen in which topotecan is treated daily for 5 days while berzosertib is dosed on days 2 and 5 of 7-day cycles (Thomas *et al*, 2021). Adjusting this model for the standard dosing of lurbinectedin, we assessed the efficacy of lurbinectedin and berzosertib in mouse models of SCLC dosing lurbinectedin on day 1, followed by berzosertib on days 2 and 5 of 7-day cycles. In a patient-derived xenograft (PDX) model of SCLC, PDX-06, lurbinectedin was extremely effective by itself almost completely inhibiting tumor growth. Likely due to the high efficacy of lurbinectedin alone, we observed minimal increased activity with the addition of berzosertib (Fig 4A). We assessed a separate cohort of mice treated in the same manner for target engagement 24 h after dosing and found

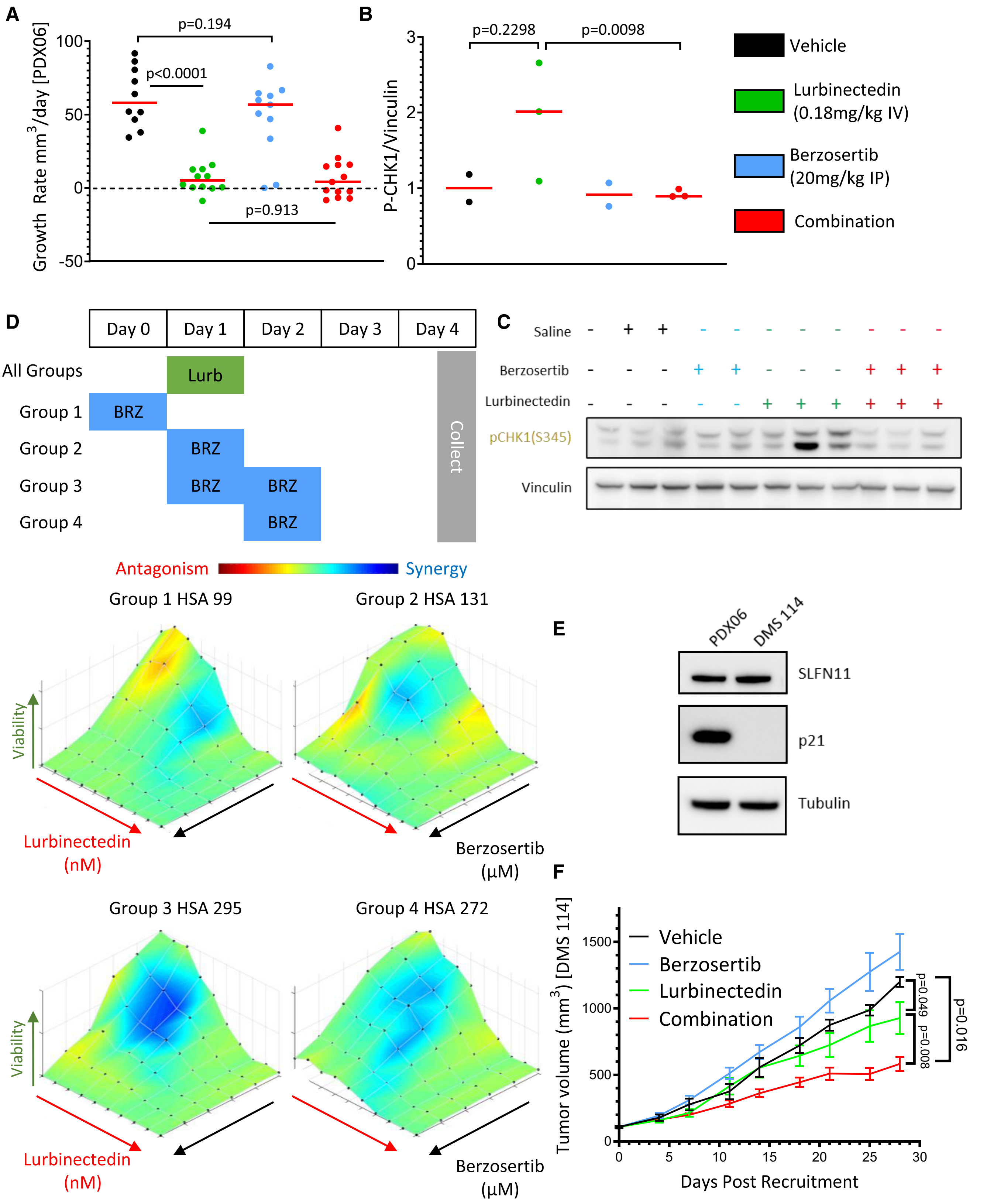

Figure 4.

© 2023 The Authors

Figure 4. Berzosertib improves lurbinectedin efficacy *in vivo*.

A Lurbinectedin was very efficacious and the addition of berzosertib did not significantly improve the almost complete inhibition of tumor growth caused by lurbinectedin in a PDX mouse model of SCLC (PDX-06), n = 10–13 mice per group, red bar indicates median, and *P*-values are indicative of unpaired two-tailed Student's *t*-test calculated in PRISM. Tumor-bearing mice were treated with lurbinectedin (0.18 mg/kg IV) and berzosertib (20 mg/kg IP) in a format mirroring our clinical trial with topotecan/berzosertib in SCLC, lurbinectedin day 1, and berzosertib days 2 and 5 of a 7-day cycle. Tumor growth rates were calculated as the difference between the initial tumor size and the final tumor size after death of the mouse due to toxicity or progression of tumor divided by the total number of days treated.

B, C We determined that lurbinectedin trended towards increasing p-chk1 while berzosertib cotreatment significantly reduced p-chk1 activation *in vivo* indicating target engagement. Tumors from mice with the same tumor type (PDX-06) as in (A) were collected 24 h after being dosed with the indicated drugs. *P*-values are indicative of unpaired two-tailed Student's *t*-test calculated in PRISM; western blot for (C) is also shown in Appendix Fig S2A.

D Lurbinectedin synergy was maximal in DMS 114 cells when treated on day 1 with lurbinectedin and days 1 and 2 with berzosertib. DMS 114 cells were treated with lurbinectedin and berzosertib in a 10 × 6 matrix format replicates = 4, n = 1. All groups were treated for 24 h with lurbinectedin, while group 1 was pretreated with berzosertib, group 2 was cotreated with berzosertib, group 3 was co- and posttreated with berzosertib, and group 4 was posttreated with berzosertib for 24 h. At the end of 5 days, cells were collected and synergy was assessed across the matrixes.

E PDX-06 (less synergistic) and DMS 114 (more synergistic) cells had equivalent SLFN11, while DMS 114 had less p21. p21 and SLFN11 protein expression was assessed using immunoblotting.

F Berzosertib cotreatment improved lurbinectedin efficacy in a DMS 114 xenograft mouse model of SCLC. Lurbinectedin was dosed at 0.18 mg/kg (intravenous) and berzosertib at 50 mg/kg (oral) for four 7-day cycles in dosing regiments consistent with (D) n = 10 mice per group. Consistent with our results in (D), the greatest degree of increased and overall efficacy was seen in group 3 (lurbinectedin day 1, berzosertib days 1 and 2), *P*-values are indicative of paired two-tailed Student's *t*-test calculated in PRISM, and error bars are representative of SEM. The other groups are displayed in Appendix Fig S7H and I.

Source data are available online for this figure.

that although results were variable, lurbinectedin caused an increase in p-CHK1, a downstream target of ATR, while berzosertib cotreatment reduced p-CHK1 activation (Figs 4B and C, and EV5A). Even though in initial testing, mice treated for a single cycle displayed no toxicity, the combination was toxic with repeated dosing. In particular, significant damage at tail veins of lurbinectedin and combination dosed mice was observed, and the majority of these mice were sacrificed due to weight loss (Appendix Fig S2K).

To better assess combination efficacy, we utilized a more aggressive PDX model PDX-03. PDX-03 displayed reduced *CDKN1A*/p21 and *SLFN11* expression and increased aggressiveness as compared to PDX-06, and thus we expected it to be more resistant to lurbinectedin and to display increased synergy for the combination (Appendix Fig S2L). To reduce toxicity, we doubled the volume of lurbinectedin dosed for tail vein injections (100–200 μl). We found in this model that lurbinectedin and combination efficacy were significantly reduced, potentially due to increased overall tumor aggressiveness in the PDX-03 model (Fig EV5B and C, and Appendix Fig S2L). Although toxicity was reduced in the PDX-03 model, several mice in lurbinectedin and combination treatment arms were sacrificed due to toxicity (Fig EV5D). While the combination showed little increased efficacy as compared to lurbinectedin alone, we found trends toward decreased marker of proliferation ki-67 and increased cleaved caspase-3 and necrosis in tumors treated with the combination as compared to either lurbinectedin or berzosertib alone (Fig EV5E). The heightened toxicity of lurbinectedin and combination may potentially be due to the strain of mice, as severe combined immunodeficient (SCID) are inherently more sensitive to DNA-damaging agents (Biedermann *et al*, 1991), we thus decided to utilize a nude mouse model which would likely have reduced toxicity.

We assessed in two cell lines (NCI-H446 and DMS 114) whether dosing sequence was critical for lurbinectedin–berzosertib synergy by dosing berzosertib before, after, or at the same time as lurbinectedin (lurbinectedin day 1 and berzosertib day(s) 0/1/2/1 + 2). The greatest synergy was observed when berzosertib was cotreated with lurbinectedin and then maintained after the removal of lurbinectedin (Figs 4D and EV5F). As DMS 114 cells had a lower NE score than NCI-H446 cells, we chose the DMS 114 cells to represent the recalcitrant non-NE subtype for our mouse model. We were able to derive a cell line from the NE PDX mouse model PDX-06, although unfortunately, PDX-03 cells did not take to culture as well. We determined that PDX-06 cells expressed similar levels of SLFN11, but significantly higher p21 than the non-NE DMS 114 cells (Fig 4E). Consistent with our findings that p21 is a critical determinant of synergy, although DMS 114 cells were more resistant to lurbinectedin alone, they displayed significantly higher synergy for the lurbinectedin–berzosertib combination than PDX-06 cells (Fig EV5F and G). In a xenograft model of DMS 114 cells in nude mice, the combination of berzosertib and lurbinectedin was significantly more effective as compared to either agent alone (Fig 4F). Consistent with our *in vitro* data, the greatest degree of synergy and antitumor activity was observed with lurbinectedin treatment on day 1 and berzosertib dosed on days 1 and 2. This combination was also well tolerated in nude mice as 0/10 mice succumbed to toxicity as compared to 11/13 and 3/11 in the PDX-06 and PDX-03 combination arms of the SCID models, respectively (Figs 4F, and EV5H and I). Of note, berzosertib administered after lurbinectedin was the least synergistic in the DMS 114 xenograft model (Fig EV5H) and suboptimal in both cell lines (Figs 4D and EV5F). As both the PDX-06 and PDX-03 models were treated with lurbinectedin first followed by berzosertib, the low synergy observed in these models could be due to the sequential model of dosing. Notably, plasma drug levels achieved in the xenograft experiments were comparable to those of human patients (Fig EV5J and K; Fudio *et al*, 2021; Thomas *et al*, 2021).

Overall, lurbinectedin causes DNA damage in an *ERCC5*/XPG-dependent manner, at which point cells can either be arrested in a *CDKN1A*/p21 (G1-phase checkpoint) or ATR (S-phase checkpoint)-dependent manner. Cells arrested in S-phase due to ATR activation will either die via SLFN11-dependent lethal replication fork instability or resolve DNA damage through HR and continue through the cell cycle. The addition of berzosertib causes cells that would normally halt cell cycle progression in S-phase to continue through the

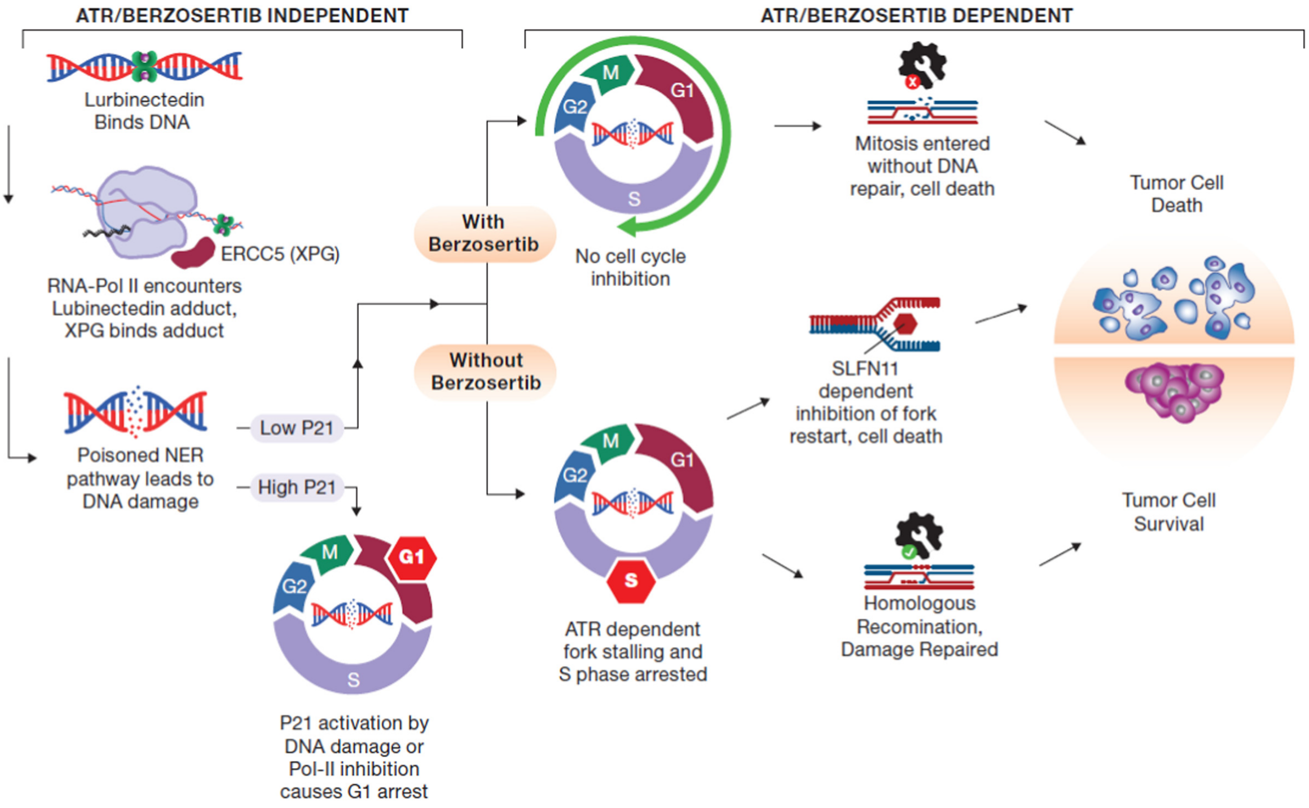

Figure 5. Berzosertib synergizes with lurbinectedin by inhibiting S-phase arrest and inducing mitotic catastrophe.

Lurbinectedin binds to DNA and then induces DNA damage through nucleotide excision repair poisoning in an XPG (*ERCC5*)-dependent manner. P21 (*CDKN1A*) activation can lead to G1 arrest in cells, however, those cells which have low *CDKN1A* expression are halted in S-phase in an ATR-dependent manner. Cells paused in S-phase either die through inability to restart fork progression in a SLFN11-dependent manner, or the DNA damage is repaired through homologous recombination. ATR inhibition with berzosertib treatment leads to a loss of the intra-S-phase checkpoint and thus cells enter mitosis with unrepaired DNA damage and ultimately undergo mitotic catastrophe. XPG and p21 are determinants of response even in the presence of berzosertib as they are required for the initial DNA damage or G1 arrest, respectively, processes which are upstream of ATR-dependent S-phase arrest.

cell cycle despite DNA damage and undergo mitotic catastrophe. XPG-dependent induction of DNA damage or p21-dependent G1 arrest occur upstream of ATR activation and thus are unaffected by berzosertib treatment. As such, *ERCC5*/XPG and *CDKN1A*/p21 are both critical determinants of lurbinectedin–berzosertib efficacy (Fig 5 and Table 1 and Table EV3).

Discussion

SCLC is a recalcitrant disease, and the majority of patients die of chemotherapy-resistant disease. We determined that the ATR inhibitor berzosertib strongly synergized with approved second-line chemotherapeutic lurbinectedin in SCLC, particularly in MYC-high, chemoresistant, non-NE models. Combination synergy is dependent on the ability of lurbinectedin to induce DNA damage, and berzosertib to augment this damage by inhibiting cell cycle checkpoints leading to mitotic catastrophe. We identified two main factors which determine response to the combination. First, the ability of lurbinectedin to cause DNA damage is reliant upon NER, and in particular, the expression of *ERCC5*/XPG. Second, the expression of *CDKN1A*/p21 is predictive of synergy, with higher *CDKN1A*/p21 expression leading to decreased synergy. Importantly, both factors are associated with NE differentiation and MYC overexpression, with recalcitrant high-MYC, non-NE tumors displaying increased *ERCC5*/XPG and decreased *CDKN1A*/p21 leading to increased synergy of the combination.

SCLC may be particularly responsive to this combination due to the frequent mutations of *TP53* and thus decreased ability of cells to activate *CDKN1A*/p21 in response to DNA damage. Previous work has indicated that ATM inhibitors were required for ATR inhibitors to synergize effectively with lurbinectedin (Lima *et al*, 2016). We found that in the majority of SCLC cell lines, ATR and lurbinectedin were synergistic in the absence of ATM inhibitors, potentially due to the high frequency of loss-of-function *TP53* mutations. Furthermore, ATM inhibition was more impactful in the rare *TP53*-proficient cells as compared to *TP53*-mutant cells.

HR deficiency led to an increase in lurbinectedin efficacy. This observation is consistent with previous findings in breast cancer where HR-deficient breast cancers, particularly *BRCA2*-mutant tumors, were more likely to respond to lurbinectedin than *BRCA1*/2 wild-type tumors (Cruz *et al*, 2018). Classical HR deficiency due to

 © 2023 The Authors

HR pathway member mutation (i.e., *BRCA1/2*) is rare in SCLC (Sato *et al*, 2013; George *et al*, 2015; Farago *et al*, 2019; Simpson *et al*, 2020). However, based on our results in the BRCA2-KO DT40 cells where synergy was reduced but not abolished, we believe that the addition of berzosertib would still be meaningful in an HR-deficient setting. This is supported by our results in the SCLC cell line NCI-H1048, in which lurbinectedin and berzosertib were synergistic. This is noteworthy as NCI-H1048 cells were ~10-fold more sensitive to DNA-damaging agents (lurbinectedin and topotecan) as compared to other SCLC cell lines tested and displayed low *BRCA2* expression indicating this line may be HR deficient or have reduced HR competency.

Consistent with recently published work (Kundu *et al*, 2021), SLFN11 expression trended toward predicting lurbinectedin efficacy, and *SLFN11*-KO reduced lurbinectedin efficacy and increased lurbinectedin–berzosertib synergy. However, we found that SLFN11 expression across cell lines did not correlate with synergy and that there was still significant synergy *in vitro* and *in vivo* even in SCLC models with high expression of SLFN11. While cell lines/tumors with high SLFN11 are more sensitive to lurbinectedin alone, thus reducing the impact of adding an ATR inhibitor, lurbinectedin–berzosertib synergy is not mechanistically dependent on SLFN11 expression levels. This is supported by *ERCC5*/XPG-knockout cells which are also resistant to lurbinectedin, but unlike SLFN11-knockout cells, they display decreased synergy and lurbinectedin efficacy cannot be rescued with the addition of berzosertib. Based on these data and the high degree of heterogeneity in second-line SCLC, we are not currently stratifying patients based on SLFN11 or HR status although we will continue to observe these parameters.

In a previous clinical trial, lurbinectedin in combination with the topoisomerase II inhibitor doxorubicin failed to improve survival as compared to standard of care (Helwik, 2021). This may be due to similar mechanisms of action for both agents as both lurbinectedin and doxorubicin cause DNA damage through poisoning DNA–protein interactions (lurbinectedin binds DNA and XPG while doxorubicin binds DNA and topoisomerase II), likely leading to similar resistance and repair mechanisms (i.e., HR and SLFN11). We have demonstrated that lurbinectedin has much greater synergistic potential with ATR inhibitors, in particular berzosertib, than with doxorubicin as part of our initial screen. In light of the improved synergy of lurbinectedin and berzosertib as compared to doxorubicin, and the diverse mechanisms of action, we believe lurbinectedin and berzosertib may have improved efficacy in SCLC patients as compared to lurbinectedin and doxorubicin.

Berzosertib is currently being assessed in combination with topotecan for the treatment of SCLC and has so far shown promise leading to durable responses in several patients (Thomas *et al*, 2018, 2021). Both topotecan and lurbinectedin synergized highly with berzosertib based on our work, and although lurbinectedin was more potent in 7/9 SCLC cell lines tested, due to different dosing schemes and bioavailability whether this will impact efficacy in patients is unclear. The combination of berzosertib and topotecan has, however, shown the greatest response in patients with high-NE differentiation (Thomas *et al*, 2021), whereas our work has shown that the lurbinectedin–berzosertib combination is likely to have the greatest degree of improvement in non-NE patients. In particular, the increased *ERCC5*/XPG in non-NE tumors should only effect lurbinectedin–berzosertib combination efficacy, with little to no effect on topotecan–berzosertib combination efficacy. With continued assessment of biomarkers for both combinations, in future, second-line patients with NE SCLC could be treated with topotecan–berzosertib while patients with non-NE SCLC may be treated with lurbinectedin–berzosertib.

Based on these results, a phase I/II clinical trial with a combination of lurbinectedin and berzosertib was launched (NCT04802174) to determine antitumor efficacy in SCLC patients. In light of our findings described here, it will be important to assess whether NE differentiation along with *ERCC5*/XPG, *CDKN1A*/p21, and MYC family member expression are predictors for the efficacy of the combination treatment in patients.

Materials and Methods

Cell lines

NCI-H211, NCI-H524, DMS-114, NCI-H841, NCI-H1048, NCI-H1341, NCI-H446, NCI-H146, NCI-H889, and U2OS DRGFP (Nakanishi *et al*, 2011) cell lines were purchased from ATCC. NCI-H211, NCI-H889, NCI-H1048, NCI-H1341, and U2OS DRGFP cell lines are female and the rest are male, additional information for cell lines can be observed in Table 1. Cell lines were authenticated using short tandem repeat analysis, and were monthly tested for mycoplasma contamination. PDX-03 and PDX-06 were derived from a male and a female SCLC patient, respectively. Cell medium was RPMI-1640 supplemented with 10% FBS for all lines to maintain consistency. DT40 (chicken cell lines) were grown in Dulbecco's modified Eagle's medium (DMEM) supplemented with FBS 10% and chicken serum 5%. Cells were grown at 37°C and 5% CO_2.

Organoids

Human small-cell autopsies/biopsy specimens were obtained from the CCR-NCI biobank following patient consent and NIH institutional review board (IRB) and ethical approval. The pathological specimens were immediately stored in storage media (1× DMEM/F12, 1× Glutamax, and 10 mM HEPS buffer) on ice. The tissues were immediately subjected to enzymatic disassociation. Human small-cell organoids were cultured in minimal basal media (MBM) as described previously (Kim *et al*, 2019; Sedlack *et al*, 2022). Briefly, PDOs were cultured in drop of growth factor-reduced basement membrane extract (BME; Corning), and medium was refreshed every 4 days. The culture media contain DMEM/F12 (Gibco) with 50 ng/ml EGF (StemCell Technologies), 100 nM IGF-1 (StemCell Technologies), 1× N2 supplement (Thermo Fisher Scientific), 1× B27 (Themo Fisher Scientific), and 10 μM Y-27632 (StemCell Technologies). The organoids were passage through shear stress with cold 1 U/ml dispase/DMEM/F12 solution (StemCell Technologies) followed by trypsin–EDTA (Invitrogen). Organoids were grown at 37°C and 5% CO_2. PDX-456648 was derived from a male with small-cell cancer of the bladder and PDX-592484 was from a male with small-cell lung cancer.

Mouse tumor models

Eight-week-old male or female NSG mice (NOD.Cg-Prkdc scid Il2rg tm1Wjl/SzJ; # 005557, The Jackson Laboratory, Bar Harbor, ME)

© 2023 The Authors

were implanted subcutaneously with fresh patient-needle biopsy supported with Matrigel (Corning) to generate our PDX model. Mice were treated weekly with lurbinectedin at 0.18 mg/kg intravenously on day 1, and berzosertib at 20 mg/kg intraperitoneally on day 1/5. For our pharmacodynamic study, mice were harvested 24 h after the second dose of lurbinectedin and berzosertib. The Animal Study Protocol was approved and followed the Frederick National Laboratory Animal Care and Use Committee guidelines.

For the DMS 114 xenograft mouse model, 8- to 10-week-old female H2dRag2 mice (C;129P2-H2d-Rag2 < tm1Fwa IL2rgtm1; Taconic, Denmark) were used. Mice were treated with weekly cycles consisting of the following arms: vehicle, lurbinectedin alone, berzosertib alone, lurbinectedin day 1 berzosertib day 1, lurbinectedin day 1 berzosertib day 2, and lurbinectedin day 1 berzosertib days 1 and 2. Lurbinectedin was dosed at 0.18 mg/kg intravenously, and berzosertib at 50 mg/kg orally. The study design and animal usage were approved by local animal welfare authorities (Regierungspräsidium Darmstadt, Hesse, Germany, protocol registration number DA4/Anz.1040). All animal housing and husbandry were performed according to veterinary standards.

Screening and synergy

The screen analyzed in Fig 1A–C, and Tables EV1 and EV2 was performed in NCI-H446 SCLC cells as described previously (Thomas *et al*, 2021); the data for this screen (12831) are available at https://matrix.ncats.nih.gov/. For further synergy analysis, cells were seeded at 1,000 cells per well in 384-well plates, and collected using Cell Titer Glo (Promega, Madison, WI, USA) 72 h after drug treatment. Highest single-agent (HSA) synergy was determined by calculating the difference between the most effective single agent and the combination of agents. All matrix formats stated in this work will be presented as lurbinectedin x berzosertib with one control, that is, a 10 × 6 matrix would have 10 concentrations of lurbinectedin with 1 being 0 across 6 concentrations of berzosertib. For thymidine-based experiments, cells were plated at 1,000 cells per well in 384-well plates. On the next day, they were treated with thymidine 2 mM for 18 h followed by a 6–9 h release and then treated with thymidine at 2 mM ± 1 nM lurbinectedin ± 2 μM berzosertib for 72 h.

Comet assay

DMS 114 cells were plated at 500 K cells per well in a six-well plate, and cells were treated with ±1 nM lurbinectedin and ±2 μM berzosertib for 6 h and collected. Comet assays were performed using the Comet Assay Single Cell Gel Electrophoresis Assay (Trevigen, Gaithersburg, MD) according to manufacturer's instructions. Images were captured using BioSpa Live Cell Analysis System (Biotek) and comet tail length was calculated using OpenComet (https://cometbio.org/), a plugin for the image processing program ImageJ.

Immunoblotting

For immunoblotting experiments, we utilized the following antibodies: −γH2AX(S139) (Cell Signaling #80312), H2AX (Sigma-Aldrich # 07627), pRPA32(T21) (Abcam #ab109394), RPA32 (Cell Signaling #35869), −pATR(T1986) (Cell Signaling #58014 and #30632), ATR (Cell Signaling #13934), pCHK1(S345) (Cell Signaling #12302), CHK1 (Cell Signaling #2360), pATM(S1981) (Abcam #ab81292), ATM (Cell Signaling #2873), pCHK2(T68) (Cell Signaling #2197), CHK2 (Cell Signaling #6334), pDNA-PK(S2056) (Abcam# ab18192), DNA-PK (Abcam# ab32566), vinculin (Sigma-Aldrich # V9131), histone H3 (Sigma-Aldrich #07-690), and SLFN11 (Santa-Cruz #sc-374339). All antibodies were diluted at 1:1,000 except SLFN11 1:2,000, tubulin 1:4,000, and vinculin 1:4,000, and all secondary antibodies were diluted at 1:4,000.

Immunofluorescence

Cells were fixed with 2% paraformaldehyde/PBS for 20 min at room temperature, washed three times in PBS, cells were deposited on slide glass by cytospin, then put in prechilled 70% ethanol for 20 min, and blocked with 5% BSA/PBSTT (PBS-containing 0.5% Tween 20 and 0.1% Triton X-100) for 30 min. Slides were incubated with primary antibodies for 2 h and secondary antibodies for 1 h at room temperature. Images were captured with a Zeiss LSM 780 confocal microscope.

DNA combing

DMS 114 cells were treated with ±1 nM lurbinectedin and ±2 μM berzosertib for 6 h followed by treatment with CIDU for 30 min and then IDU for 30 min and collected. DNA combing assay was performed as described previously (Josse *et al*, 2014; Thomas *et al*, 2021).

Assessment of mitotic catastrophe by fluorescence microscopy

Using fluorescence microscopy, mitotic catastrophe was identified based on the characteristic morphologies of the DAPI-stained nuclei. Cells were seeded in 12-well plates on sterilized coverslips and treated the next day with vehicle, 1 nM lurbinectedin, 2 μM berzosertib, or combination of both for 6 h. Nocodazole was added into media for 3 h during the drug treatment (to enrich mitotic cells), then nocodazole and drugs were removed and fresh media were added for 45 min. After washing cells with cold PBS, fixation was done in 4% paraformaldehyde in PBS for 15 min at RT, followed by permeabilization with 0.2% Triton-X 100/PBS for 15 min. After washing with PBS, cells were mounted with DAPI (VECTASHIELD, Vector Laboratories). Images of nuclei were captured with Zeiss LSM 880 confocal microscope with 63× objective lens.

RNA analysis, NE calculation, and enrichment analysis

RNA data and neuroendocrine score calculation for cell lines are publicly available on CellMiner (Tlemsani *et al*, 2020). Patient tumor and CDX datasets of SCLC were obtained from publicly available datasets (integrated genomic and transcriptomic analysis of small-cell lung cancer reveals inter- and intratumoral heterogeneity and a novel chemotherapy refractory subtype; under revision, data available at dbGaP Study Accession: phs002541.v1.p1; Sato *et al*, 2013; George *et al*, 2015; Farago *et al*, 2019; Simpson *et al*, 2020). High MYC was determined in the patient and CDX datasets through assessing the expression of MYC, L-MYC, and N-MYC, z-scoring them, and selecting the max z-scored expression; high-MYC was defined as 1 SD above mean. ssGSEA hallmark and

© 2023 The Authors

The paper explained

Problem

Small-cell lung cancer (SCLC) is a recalcitrant disease with a 9% 5-year survival rate. This recalcitrance is driven in part by a high degree of intratumoral heterogeneity. SCLC tumors present with high neuroendocrine (NE) differentiation and evolve, driven by MYC, to a non-neuroendocrine (non-NE) state. DNA-damaging RNA Pol-II inhibitor lurbinectedin was approved for second-line therapy in 2020, however, only 35% of patients respond and the vast majority of patients still succumb to their disease. Combinatorial approaches are required to improve the efficacy of lurbinectedin.

Results

Here, we find that lurbinectedin shows high synergy with the ataxia telangiectasia–mutated and rad3-related (ATR) inhibitor berzosertib. Efficacy of the combination is dependent on expression of *ERCC5*/XPG and can be inhibited by *CDKN1A*/p21-dependent G1 arrest. Based on the high expression of XPG and decreased *CDKN1A* in MYC-high non-NE SCLC, we propose that non-NE SCLC is likely to be most susceptible to the combination of lurbinectedin and berzosertib.

Impact

ATR inhibition synergizes with lurbinectedin in SCLC models. *CDKN1A*/p21 and *ERCC5*/XPG expression are determinants of response. An ongoing clinical trial is examining the combination of lurbinectedin and berzosertib in relapsed SCLC (NCT04802174).

neuroendocrine enrichment scores were computed using the GSVA R/Bioconductor package (Hanzelmann *et al*, 2013; Liberzon *et al*, 2015; Zhang *et al*, 2018).

Metaphase spread

DMS 114 cells were plated at 1 million cells per 10 cm plate, and treated with ±1 nM lurbinectedin and ±2 μM berzosertib for 6 h and collected. Metaphase spread was performed as described https://ccr.cancer.gov/sites/default/files/metaphase_preparation_from_adherent_cells.pdf. Cells were imaged using a Leica Thunder Imager.

Flow cytometry

Cells were treated with ±1 nM lurbinectedin and ±2 μM berzosertib for 6 h, and ethynyl deoxyuridine (EDU) at 100 μM was added for the last hour prior to collection. EdU was detected using flow cytometry (Click-iT EdU Alexa Fluor 647 Flow Cytometry Assay, Invitrogen), DNA using DAPI, and γH2AX was assessed by staining cells with JBW301 (Millipore Sigma). Data were acquired using a BD LSRFortessa Flow Cytometer and analyzed using FlowJo.

Statistics

Statistical analysis was performed using Prism 9.3.1 (GraphPad). *P*-values < 0.05 were considered statistically significant. For animal experiments, mice were randomized in an unbiased fashion. Researchers were not blinded during mouse experiments. Samples sizes were selected to, based on estimated efficacy data, give a 90% chance of observing statistically significant deviations at $P < 0.05$ in efficacy between the combination and either individual treatment arm.

Patient data

NIH IRB, Office of Human Subjects Research Protections at NCI, approved the studies; all patients provided written informed consent for tumor sample sequencing. The experiments conformed to the principles set out in the WMA Declaration of Helsinki and the Department of Health and Human Services Belmont Report.

Data availability

This study includes no data deposited in external repositories.

Expanded View for this article is available online.

Acknowledgements

This work was supported in part by the intramural programs of the Center for Cancer Research, National Cancer Institute (ZIA BC 011793), and EMD Serono (CrossRef Funder ID: 10.13039/100004755). PDX 456648 and PDX 592484 were generated and graciously provided by the National Cancer Institute Patient Derived Models Repository. We would like to acknowledge and thank Dr. Thomas Ried for his support along with insightful comments and suggestions.

Author contributions

Christopher W Schultz: Conceptualization; data curation; formal analysis; validation; investigation; visualization; methodology; writing – original draft; project administration; writing – review and editing. **Yang Zhang:** Data curation; investigation; methodology; writing – review and editing. **Rajaa Elmeskini:** Data curation; formal analysis; investigation; methodology; writing – review and editing. **Astrid Zimmermann:** Formal analysis; investigation; methodology; writing – review and editing. **Haiqing Fu:** Investigation; methodology; writing – review and editing. **Yasuhisa Murai:** Investigation; methodology. **Darawalee Wangsa:** Investigation; methodology. **Suresh Kumar:** Resources; investigation. **Nobuyuki Takahashi:** Conceptualization; investigation; writing – review and editing. **Devon Atkinson:** Investigation; methodology; writing – review and editing. **Liton Kumar Saha:** Investigation; methodology. **Chien-Fei Lee:** Investigation; methodology. **Brian Elenbaas:** Investigation; methodology; writing – review and editing. **Parth Desai:** Investigation; methodology; writing – review and editing. **Robin Sebastian:** Resources; investigation; methodology.
Ajit Kumar Sharma: Methodology. **Melissa Abel:** Visualization.
Brett Schroeder: Visualization. **Manan Krishnamurthy:** Validation.
Rajesh Kumar: Data curation. **Nitin Roper:** Resources.
Mirit Aladjem: Resources; writing – review and editing. **Frank T Zenke:** Resources; visualization; writing – review and editing. **Zoe Weaver Ohler:** Resources; visualization; writing – review and editing. **Yves Pommier:** Resources; visualization; writing – review and editing. **Anish Thomas:** Conceptualization; resources; supervision; funding acquisition; visualization; methodology; writing – original draft; project administration; writing – review and editing.

Disclosure and competing interests statement

This work was supported by the intramural programs of the Center for Cancer Research, NCI (ZIA BC 011793). AZ and FTZ are employees of Merck KGaA, Darmstadt, Germany. BE and C-FL are employees of the EMD Serono Research

© 2023 The Authors

& Development Institute Inc., Billerica, MA, USA; a business of Merck KGaA, Darmstadt, Germany. AT and YP report research funding to the institution from the following entities: EMD Serono (CrossRef Funder ID: 10.13039/100004755), AstraZeneca, Tarveda Therapeutics, Immunomedics, and Prolynx Inc.

For more information

Screening data for this paper along with other screens is available at https://matrix.ncats.nih.gov/. CellminerCDB is a website which allows access to and compiles many different primarily cell-line based datasets, this useful website can be found at https://discover.nci.nih.gov/rsconnect/cellminercdb/.

References

Al-Haj L, Blackshear PJ, Khabar KS (2012) Regulation of p21/CIP1/WAF-1 mediated cell-cycle arrest by RNase L and tristetraprolin, and involvement of AU-rich elements. *Nucleic Acids Res* 40: 7739 – 7752

Alves Rde C, Meurer RT, Roehe AV (2014) MYC amplification is associated with poor survival in small cell lung cancer: a chromogenic in situ hybridization study. *J Cancer Res Clin Oncol* 140: 2021 – 2025

Balanis NG, Sheu KM, Esedebe FN, Patel SJ, Smith BA, Park JW, Alhani S, Gomperts BN, Huang J, Witte ON *et al* (2019) Pan-cancer convergence to a small-cell neuroendocrine phenotype that shares susceptibilities with hematological malignancies. *Cancer Cell* 36: 17 – 34

Berenbaum MC (1989) What is synergy? *Pharmacol Rev* 41: 93 – 141

Biedermann KA, Sun JR, Giaccia AJ, Tosto LM, Brown JM (1991) Scid mutation in mice confers hypersensitivity to ionizing radiation and a deficiency in DNA double-strand break repair. *Proc Natl Acad Sci USA* 88: 1394 – 1397

Blackford AN, Jackson SP (2017) ATM, ATR, and DNA-PK: the trinity at the heart of the DNA damage response. *Mol Cell* 66: 801 – 817

Ciccia A, Elledge SJ (2010) The DNA damage response: making it safe to play with knives. *Mol Cell* 40: 179 – 204

Cruz C, Llop-Guevara A, Garber JE, Arun BK, Perez Fidalgo JA, Lluch A, Telli ML, Fernandez C, Kahatt C, Galmarini CM *et al* (2018) Multicenter phase II study of Lurbinectedin in BRCA-mutated and unselected metastatic advanced breast cancer and biomarker assessment substudy. *J Clin Oncol* 36: 3134 – 3143

Farago AF, Yeap BY, Stanzione M, Hung YP, Heist RS, Marcoux JP, Zhong J, Rangachari D, Barbie DA, Phat S *et al* (2019) Combination Olaparib and Temozolomide in relapsed small-cell lung cancer. *Cancer Discov* 9: 1372 – 1387

Fiorentino FP, Tokgun E, Sole-Sanchez S, Giampaolo S, Tokgun O, Jauset T, Kohno T, Perucho M, Soucek L, Yokota J (2016) Growth suppression by MYC inhibition in small cell lung cancer cells with TP53 and RB1 inactivation. *Oncotarget* 7: 31014 – 31028

Fudio S, Tabernero J, Subbiah V, Chawla SP, Moreno V, Longo F, Lopez R, Anton A, Trigo JM, Shapiro G *et al* (2021) Effect of lurbinectedin on the QTc interval in patients with advanced solid tumors: an exposure-response analysis. *Cancer Chemother Pharmacol* 87: 113 – 124

Gay CM, Stewart CA, Park EM, Diao LX, Groves SM, Heeke S, Nabet BY, Fujimoto J, Solis LM, Lu W *et al* (2021) Patterns of transcription factor programs and immune pathway activation define four major subtypes of SCLC with distinct therapeutic vulnerabilities. *Cancer Cell* 39: 346 – 360

George J, Lim JS, Peifer M, Sage J, Thomas R (2015) Comprehensive genomic characterization of small cell lung cancer. *Cancer Res* 75: LB-168

Golding SE, Rosenberg E, Valerie N, Hussaini I, Frigerio M, Cockcroft XF, Chong WY, Hummersone M, Rigoreau L, Menear KA *et al* (2009) Improved ATM kinase inhibitor KU-60019 radiosensitizes glioma cells, compromises insulin, AKT and ERK prosurvival signaling, and inhibits migration and invasion. *Mol Cancer Ther* 8: 2894 – 2902

Hanzelmann S, Castelo R, Guinney J (2013) GSVA: gene set variation analysis for microarray and RNA-seq data. *BMC Bioinformatics* 14: 7

Hauge S, Macurek L, Syljuasen RG (2019) p21 limits S phase DNA damage caused by the Wee1 inhibitor MK1775. *Cell Cycle* 18: 834 – 847

Helwik C (2021) No overall survival benefit with Lurbinectedin/doxorubicin in small cell lung cancer, The ASCO Post. ASCO Post

Horita N, Yamamoto M, Sato T, Tsukahara T, Nagakura H, Tashiro K, Shibata Y, Watanabe H, Nagai K, Inoue M *et al* (2015) Topotecan for relapsed small-cell lung cancer: systematic review and meta-analysis of 1347 patients. *Sci Rep* 5: 15437

Housman G, Byler S, Heerboth S, Lapinska K, Longacre M, Snyder N, Sarkar S (2014) Drug resistance in cancer: an overview. *Cancers (Basel)* 6: 1769 – 1792

Ireland AS, Micinski AM, Kastner DW, Guo B, Wait SJ, Spainhower KB, Conley CC, Chen OS, Guthrie MR, Soltero D *et al* (2020) MYC drives temporal evolution of small cell lung cancer subtypes by reprogramming neuroendocrine fate. *Cancer Cell* 38: e12

Iyer DR, Rhind N (2017) Replication fork slowing and stalling are distinct, checkpoint-independent consequences of replicating damaged DNA. *PLoS Genet* 13: e1006958

Jo U, Murai Y, Chakka S, Chen L, Cheng K, Murai J, Saha LK, Miller Jenkins LM, Pommier Y (2021a) SLFN11 promotes CDT1 degradation by CUL4 in response to replicative DNA damage, while its absence leads to synthetic lethality with ATR/CHK1 inhibitors. *Proc Natl Acad Sci USA* 118: e2015654118

Jo U, Senatorov IS, Zimmermann A, Saha LK, Murai Y, Kim SH, Rajapakse VN, Elloumi F, Takahashi N, Schultz CW *et al* (2021b) Novel and highly potent ATR inhibitor M4344 kills cancer cells with replication stress, and enhances the chemotherapeutic activity of widely used DNA damaging agents. *Mol Cancer Ther* 20: 1431 – 1441

Josse R, Martin SE, Guha R, Ormanoglu P, Pfister TD, Reaper PM, Barnes CS, Jones J, Charlton P, Pollard JR *et al* (2014) ATR inhibitors VE-821 and VX-970 sensitize cancer cells to topoisomerase I inhibitors by disabling DNA replication initiation and fork elongation responses. *Cancer Res* 74: 6968 – 6979

Kim M, Mun H, Sung CO, Cho EJ, Jeon HJ, Chun SM, Jung DJ, Shin TH, Jeong GS, Kim DK *et al* (2019) Patient-derived lung cancer organoids as in vitro cancer models for therapeutic screening. *Nat Commun* 10: 3991

Kim ST, Smith SA, Mortimer P, Loembe AB, Cho H, Kim KM, Smith C, Willis S, Irurzun-Arana I, Berges A *et al* (2021) Phase I study of Ceralasertib (AZD6738), a novel DNA damage repair agent, in combination with weekly paclitaxel in refractory cancer. *Clin Cancer Res* 27: 4700 – 4709

Kundu K, Cardnell RJ, Zhang B, Shen L, Stewart CA, Ramkumar K, Cargill KR, Wang J, Gay CM, Byers LA (2021) SLFN11 biomarker status predicts response to lurbinectedin as a single agent and in combination with ATR inhibition in small cell lung cancer. *Transl Lung Cancer Res* 10: 4095 – 4105

Liberzon A, Birger C, Thorvaldsdottir H, Ghandi M, Mesirov JP, Tamayo P (2015) The molecular signatures database (MSigDB) hallmark gene set collection. *Cell Syst* 1: 417 – 425

Lima M, Bouzid H, Soares DG, Selle F, Morel C, Galmarini CM, Henriques JA, Larsen AK, Escargueil AE (2016) Dual inhibition of ATR and ATM potentiates the activity of trabectedin and lurbinectedin by perturbing the DNA damage response and homologous recombination repair. *Oncotarget* 7: 25885 – 25901

 © 2023 The Authors

Lopez-Chavez A, Thomas A, Rajan A, Raffeld M, Morrow B, Kelly R, Carter CA, Guha U, Killian K, Lau CC *et al* (2015) Molecular profiling and targeted therapy for advanced thoracic malignancies: a biomarker-derived, multiarm, multihistology phase II basket trial. *J Clin Oncol* 33: 1000 – 1007

Murai J, Feng Y, Yu GK, Ru Y, Tang SW, Shen Y, Pommier Y (2016) Resistance to PARP inhibitors by SLFN11 inactivation can be overcome by ATR inhibition. *Oncotarget* 7: 76534 – 76550

Nakanishi K, Cavallo F, Brunet E, Jasin M (2011) Homologous recombination assay for interstrand cross-link repair. *Methods Mol Biol* 745: 283 – 291

Paz-Ares L, Dvorkin M, Chen Y, Reinmuth N, Hotta K, Trukhin D, Statsenko G, Hochmair MJ, Ozguroglu M, Ji JH *et al* (2019) Durvalumab plus platinum-etoposide versus platinum-etoposide in first-line treatment of extensive-stage small-cell lung cancer (CASPIAN): a randomised, controlled, open-label, phase 3 trial. *Lancet* 394: 1929 – 1939

Pongor LS, Schultz CW, Rinaldi L, Wangsa D, Redon CE, Takahashi N, Fialkoff G, Desai P, Zhang Y, Burkett S *et al* (2023) Extrachromosomal DNA amplification contributes to small cell lung cancer heterogeneity and is associated with worse outcomes. *Cancer Discov* 13: 928 – 949

Rajeshkumar NV, De Oliveira E, Ottenhof N, Watters J, Brooks D, Demuth T, Shumway SD, Mizuarai S, Hirai H, Maitra A *et al* (2011) MK-1775, a potent Wee1 inhibitor, synergizes with gemcitabine to achieve tumor regressions, selectively in p53-deficient pancreatic cancer xenografts. *Clin Cancer Res* 17: 2799 – 2806

Riches LC, Trinidad AG, Hughes G, Jones GN, Hughes AM, Thomason AG, Gavine P, Cui A, Ling S, Stott J *et al* (2020) Pharmacology of the ATM inhibitor AZD0156: potentiation of irradiation and Olaparib responses Preclinically. *Mol Cancer Ther* 19: 13 – 25

Romano M, Frapolli R, Zangarini M, Bello E, Porcu L, Galmarini CM, Garcia-Fernandez LF, Cuevas C, Allavena P, Erba E *et al* (2013) Comparison of in vitro and in vivo biological effects of trabectedin, lurbinectedin (PM01183) and Zalypsis(R) (PM00104). *Int J Cancer* 133: 2024 – 2033

Roper N, Velez MJ, Chiappori A, Kim YS, Wei JS, Sindiri S, Takahashi N, Mulford D, Kumar S, Ylaya K *et al* (2021) Notch signaling and efficacy of PD-1/PD-L1 blockade in relapsed small cell lung cancer. *Nat Commun* 12: 3880

Rudin CM, Poirier JT, Byers LA, Dive C, Dowlati A, George J, Heymach JV, Johnson JE, Lehman JM, MacPherson D *et al* (2019) Molecular subtypes of small cell lung cancer: a synthesis of human and mouse model data. *Nat Rev Cancer* 19: 289 – 297

Saldivar JC, Hamperl S, Bocek MJ, Chung M, Bass TE, Cisneros-Soberanis F, Samejima K, Xie L, Paulson JR, Earnshaw WC *et al* (2018) An intrinsic S/G2 checkpoint enforced by ATR. *Science* 361: 806 – 810

Santamaria Nunez G, Robles CM, Giraudon C, Martinez-Leal JF, Compe E, Coin F, Aviles P, Galmarini CM, Egly JM (2016) Lurbinectedin specifically triggers the degradation of phosphorylated RNA polymerase II and the formation of DNA breaks in cancer cells. *Mol Cancer Ther* 15: 2399 – 2412

Sato T, Kaneda A, Tsuji S, Isagawa T, Yamamoto S, Fujita T, Yamanaka R, Tanaka Y, Nukiwa T, Marquez VE *et al* (2013) PRC2 overexpression and PRC2-target gene repression relating to poorer prognosis in small cell lung cancer. *Sci Rep* 3: 1911

Sedlack AJH, Saleh-Anaraki K, Kumar S, Ear PH, Lines KE, Roper N, Pacak K, Bergsland E, Quelle DE, Howe JR *et al* (2022) Preclinical models of neuroendocrine neoplasia. *Cancers (Basel)* 14: 5646

Simpson KL, Stoney R, Frese KK, Simms N, Rowe W, Pearce SP, Humphrey S, Booth L, Morgan D, Dynowski M *et al* (2020) A biobank of small cell lung cancer CDX models elucidates inter- and intratumoral phenotypic heterogeneity. *Nat Cancer* 1: 437 – 451

Smith HL, Southgate H, Tweddle DA, Curtin NJ (2020) DNA damage checkpoint kinases in cancer. *Expert Rev Mol Med* 22: e2

Takebayashi Y, Pourquier P, Zimonjic DB, Nakayama K, Emmert S, Ueda T, Urasaki Y, Kanzaki A, Akiyama SI, Popescu N *et al* (2001) Antiproliferative activity of ecteinascidin 743 is dependent upon transcription-coupled nucleotide-excision repair. *Nat Med* 7: 961 – 966

Thomas A, Redon CE, Sciuto L, Padiernos E, Ji J, Lee MJ, Yuno A, Lee S, Zhang Y, Tran L *et al* (2018) Phase I study of ATR inhibitor M6620 in combination with Topotecan in patients with advanced solid tumors. *J Clin Oncol* 36: 1594 – 1602

Thomas A, Takahashi N, Rajapakse VN, Zhang X, Sun Y, Ceribelli M, Wilson KM, Zhang Y, Beck E, Sciuto L *et al* (2021) Therapeutic targeting of ATR yields durable regressions in small cell lung cancers with high replication stress. *Cancer Cell* 39: 566 – 579

Tlemsani C, Pongor L, Elloumi F, Girard L, Huffman KE, Roper N, Varma S, Luna A, Rajapakse VN, Sebastian R *et al* (2020) SCLC-CellMiner: a resource for small cell lung cancer cell line genomics and pharmacology based on genomic signatures. *Cell Rep* 33: 108296

Trigo J, Subbiah V, Besse B, Moreno V, Lopez R, Sala MA, Peters S, Ponce S, Fernandez C, Alfaro V *et al* (2020) Lurbinectedin as second-line treatment for patients with small-cell lung cancer: a single-arm, open-label, phase 2 basket trial. *Lancet Oncol* 21: 645 – 654

Wagner AH, Devarakonda S, Skidmore ZL, Krysiak K, Ramu A, Trani L, Kunisaki J, Masood A, Waqar SN, Spies NC *et al* (2018) Recurrent WNT pathway alterations are frequent in relapsed small cell lung cancer. *Nat Commun* 9: 3787

Weinstock DM, Nakanishi K, Helgadottir HR, Jasin M (2006) Assaying double-strand break repair pathway choice in mammalian cells using a targeted endonuclease or the RAG recombinase. *Methods Enzymol* 409: 524 – 540

Yap TA, Tan DSP, Terbuch A, Caldwell R, Guo C, Goh BC, Heong V, Haris NRM, Bashir S, Drew Y *et al* (2020) First-in-human trial of the Oral ataxia telangiectasia and RAD3-related (ATR) inhibitor BAY 1895344 in patients with advanced solid tumors. *Cancer Discov* 11: 80 – 91

Zhang W, Girard L, Zhang YA, Haruki T, Papari-Zareei M, Stastny V, Ghayee HK, Pacak K, Oliver TG, Minna JD *et al* (2018) Small cell lung cancer tumors and preclinical models display heterogeneity of neuroendocrine phenotypes. *Transl Lung Cancer Res* 7: 32 – 49

License: This is an open access article under the terms of the Creative Commons Attribution License, which permits use, distribution and reproduction in any medium, provided the original work is properly cited.

Expanded View Figures

Figure EV1. Lurbinectedin causes γH2AX induction and reduced EDU incorporation and ATR inhibition can ameliorate these effects.

A The majority of SCLC cell lines were significantly more sensitive to lurbinectedin as compared to topotecan. IC_{50}s for topotecan and lurbinectedin were assessed in nine SCLC cell lines (biological replicates = 2, technical replicates = 3).

B Lurbinectedin induced the ATR (yellow/gold), ATM (green), and DNA-PK (blue) damage repair pathways as well as inducing γH2AX (DNA damage marker) in all three cell lines. DMS 114, NCI-H146, and NCI-H889 SCLC cell lines were treated with lurbinectedin at various concentrations for 2, 6, and 24 h. DMS 114 cells are lacking 24 h lurbinectedin concentrations of 10 and 100 nM due to excessive cell death under these conditions in the DMS 114 cell line.

C Lurbinectedin caused the greatest increase in γH2AX induction in S-phase cells in NCI-H1341 cells. NCI-H1341 cells (SCLC cell line) were treated with 1 nM lurbinectedin from 1 to 6 h and γH2AX induction was assessed using FACS, four technical replicates of 10,000 cells for each timepoint were assessed; biological replicates = 3. Cell cycle was assessed using propidium iodide.

D–G The average γH2AX signal increased with the addition of berzosertib to lurbinectedin (likely due to a small portion of cells as demonstrated in Fig 2B) while the median decreased indicating the majority of cells displayed decreased γH2AX signal. DMS 114 and NCI-H1341 cells were treated with ±1 nM lurbinectedin ±2 μM berzosertib for 6 h, for the last hour EDU was added. S-phase cells were selected using PI content in FlowJo. γH2AX signal of S-phase cells is represented with a line at the median. (F) Average and median of ~10,000 cells for all groups were assessed and compared to control for both NCI-H1341 and DMS 114, biological replicates = 3. (G) NCI-H1341 cells displayed decreased EDU signal with the addition of lurbinectedin, this was partially rescued with cotreatment with berzosertib. Comparisons were made using an unpaired two-tailed Student's *t*-test in PRISM; DMS 114 data are displayed in Fig 2B.

H, I DMS 114 cells were treated with siRNA against ATR or Control siRNA. Three days later, they were treated with ±1 nM lurbinectedin ±2 μM berzosertib for 6 h, for the last hour EDU was added. γH2AX and EdU signal of S-phase cells is represented with a line at the median. A total of 10,000 cells for each condition were assessed, biological replicates = 2.

Source data are available online for this figure.

 © 2023 The Authors

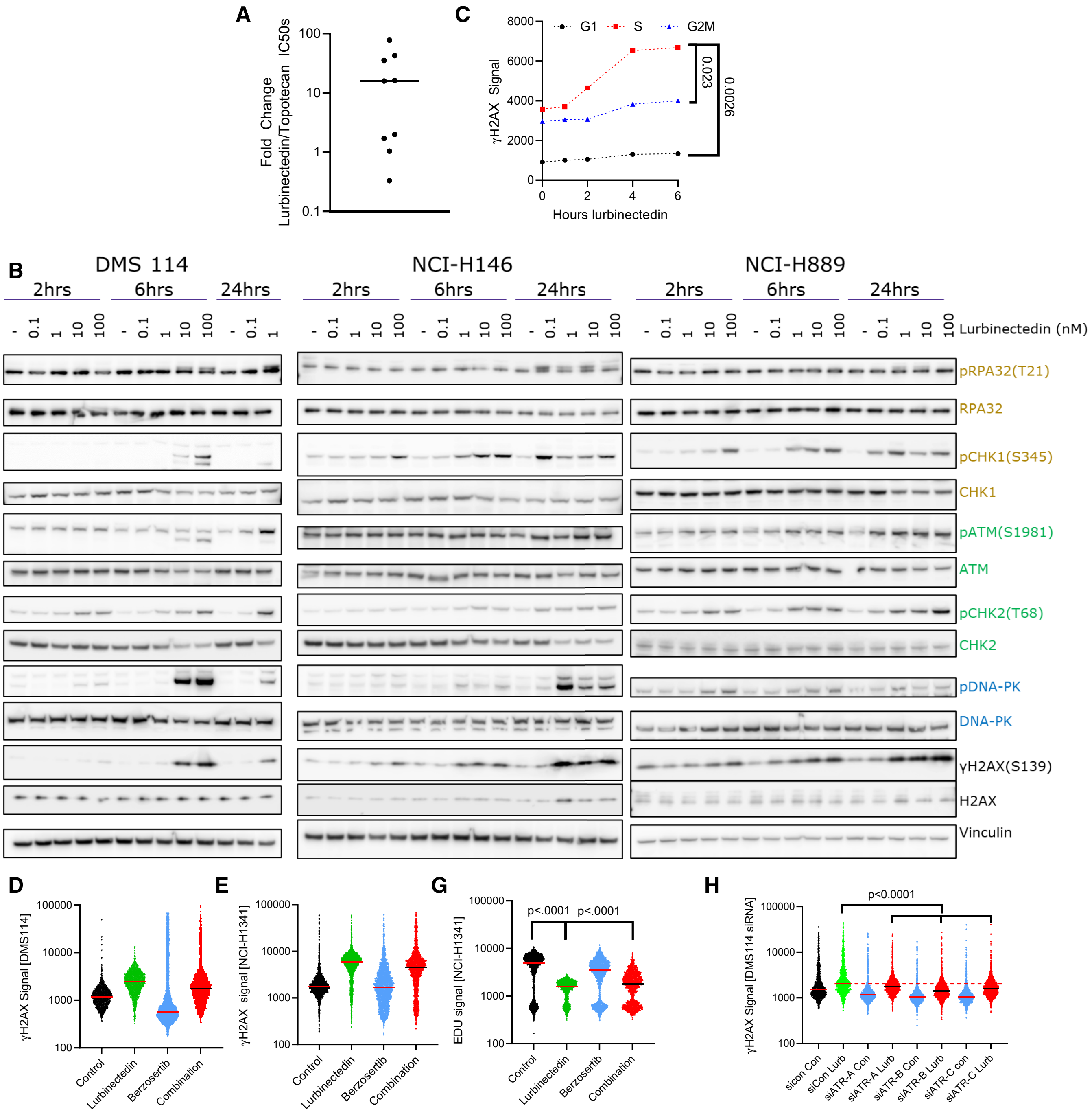

F

Cell Line	DMS 114	DMS 114	NCI-H1341	NCI-H1341
γH2AX Signal	Average	Median	Average	Median
Control	1.0	1.0	1.0	1.0
Lurbinectedin	2.1	2.1	2.6	3.3
Berzosertib	1.5	0.5	1.2	1.0
Combination	2.6	1.5	2.8	2.6

I

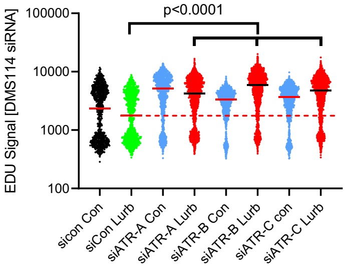

Figure EV1.

Figure EV2. XPG and XPA are required for combination efficacy, while ATR inhibition can rescue SLFN11-KO induced lurbinectedin resistance.

A, B BRCA2 KO led to increased lurbinectedin efficacy while XPG and XPA KO decreased efficacy, none of the knockouts displayed significantly different sensitivity to berzosertib. Lurbinectedin and berzosertib were treated in wild-type, BRCA2-KO, ERCC5-KO, and XPA-KO knockout DT40 cells at varying concentrations for 72 h, technical replicates = 3 and biological replicates = 3.

C, D Berzosertib inhibited HR competency at significantly lower concentrations (IC50 95%CI 0.2571 to 0.5847 μM) than required for berzosertib to inhibit viability (IC50 95%CI 2.285 to 3.016 μM). To assess HR competency, U2OS cells were stably transfected with a plasmid-expressing truncated GFP. Upon doxycycline treatment, this truncated version is cleaved, and cells which are HR competent are selectively able to repair the plasmid allowing for the expression of full-length GFP. Berzosertib impact on HR after accounting for changes to viability was plotted against berzosertib concentration. Each sample was assessed with > 10,000 cells, biological replicates = 3. IC50s were calculated utilizing the normalized variable slope model in PRISM.

E, F BRCA2-KO marginally reduced synergy of lurbinectedin and berzosertib while XPG-KO and XPA-KO reduced synergy to a greater extent. XPG-KO and XPA-KO cells maintain resistance to lurbinectedin even in the presence of berzosertib, indicating that berzosertib cannot rescue NER deficiency-induced resistance. The synergy of berzosertib and lurbinectedin across DT40 cells with wild-type, BRCA2-KO, ERCC5-KO, or XPA-KO was assessed after 72 h of treatment in a 10 × 10 matrix format; technical replicates = 3, biological replicates = 3. In (F), we demonstrate efficacy of lurbinectedin with 500 nM berzosertib (from the matrix data) as a marker of efficacy of the combination.

G–J We determined that SLFN11-KO in two DMS 114 models reduced lurbinectedin sensitivity (G) while not significantly effecting berzosertib sensitivity (H). SLFN11-KO led to increased synergy in both models (I). Accordingly, the addition of berzosertib rescued SLFN11-induced resistance in both models as lurbinectedin IC50s in all models in the presence of 500 nM berzosertib were similar (J). DMS 114 WT and two SLFN11 clones were plated in 10 × 10 matrix formats and lurbinectedin and berzosertib synergy was assessed, replicated = 3, *n* = 3.

 © 2023 The Authors

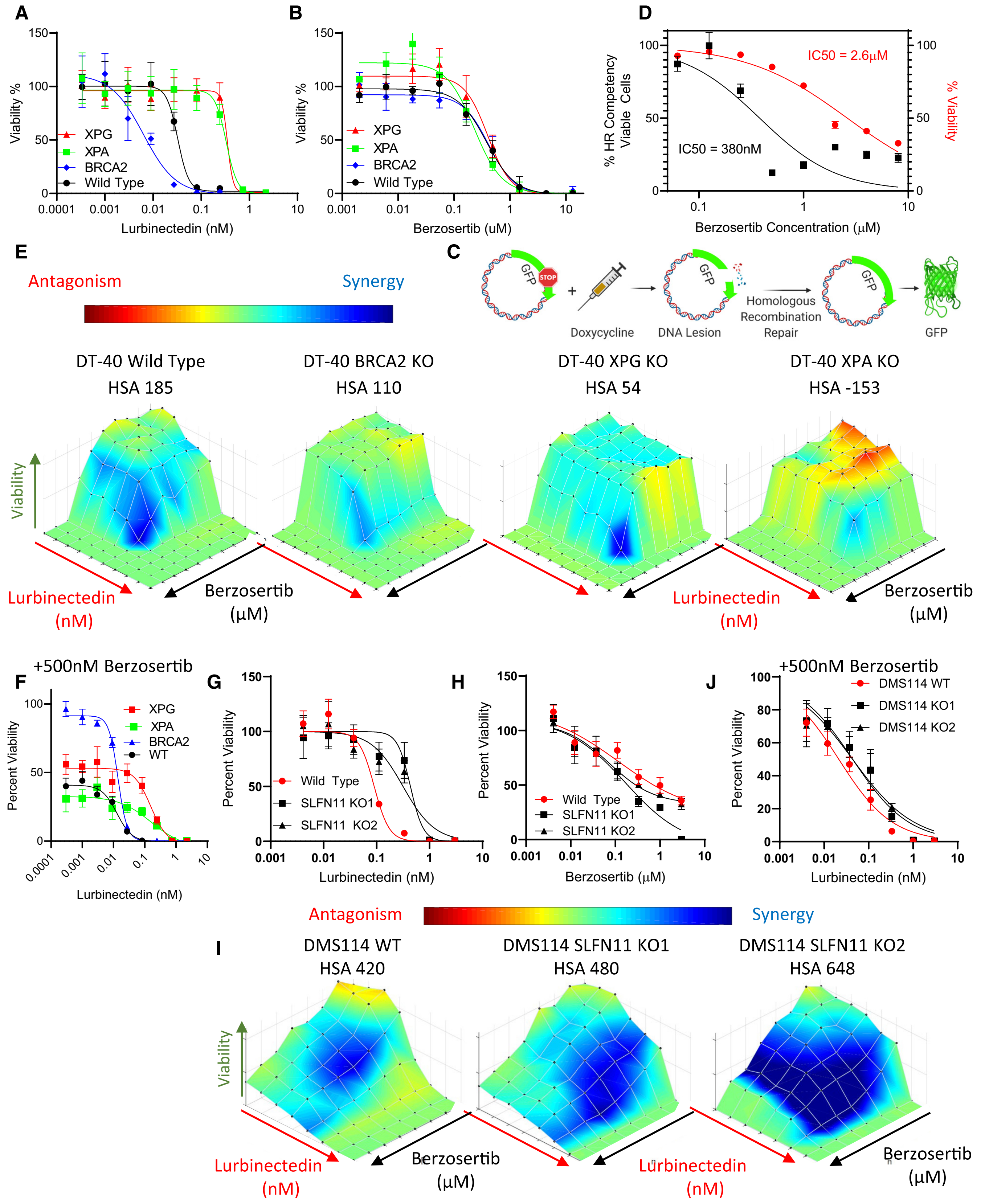
A
Viability %
XPG
XPA
BRCA2
Wild Type
Lurbinectedin (nM)
B
Berzosertib (uM)
D
% HR Competency Viable Cells
% Viability
IC50 = 2.6μM
IC50 = 380nM
Berzosertib Concentration (μM)
E
Antagonism
Synergy
C
GFP
STOP
Doxycycline
DNA Lesion
Homologous Recombination Repair
DT-40 Wild Type
HSA 185
DT-40 BRCA2 KO
HSA 110
DT-40 XPG KO
HSA 54
DT-40 XPA KO
HSA -153
Viability
Lurbinectedin (nM)
Berzosertib (μM)
F
+500nM Berzosertib
Percent Viability
WT
G
Wild Type
SLFN11 KO1
SLFN11 KO2
H
Berzosertib (μM)
J
DMS114 WT
DMS114 KO1
DMS114 KO2
I
DMS114 WT
HSA 420
DMS114 SLFN11 KO1
HSA 480
DMS114 SLFN11 KO2
HSA 648

Figure EV2.

Figure EV3. G1 arrest reduces combination synergy.

A The efficacy and synergy of lurbinectedin and berzosertib were assessed across nine cell lines in a 10 × 10 matrix format, replicates = 3, n = 1. We determined IC50s for lurbinectedin and berzosertib utilizing an unnormalized variable slope model in PRISM. RNA values for all genes investigated for these cell lines are publicly available at https://discover.nci.nih.gov/rsconnect/cellminercdb. These data represent the statistics for simple linear regression as assessed in PRISM when plotting variable 1 against variable 2. Overall, these correlations are nonsignificant, however, SLFN11 expression trended toward negatively associating with lurbinectedin IC_{50}s in the nine cell lines.

B Lurbinectedin efficacy (lurbinectedin IC50), synergy between lurbinectedin and berzosertib (HSA), and combination efficacy (lurbinectedin IC50 in the presence of 500 nm berzosertib) were assessed. Here, we demonstrate different conditions between cells that are resistant (red/alive) and sensitive (purple/dead) to either lurbinectedin alone or lurbinectedin–berzosertib in order to differentiate these terms. Importantly, the combination can be effective without synergy (i.e., the third line), meaning these terms cannot be utilized interchangeably.

C Synergistic cell lines (NCI-H1341, NCI-H841) as compared to less synergistic cell lines (NCI-H146, NCI-H899) displayed increased S-phase accumulation after lurbinectedin treatment, which could be ameliorated with berzosertib cotreatment. NCI-H1341, NCI-H841 NCI-H146, and NCI-H899 were treated with ±1 nM lurbinectedin ±2 μM berzosertib for 24 h and assessed using PI to determine cell cycle with flow cytometry analysis in FlowJo. Each treatment group for each cell line had 10,000 cells, technical replicates = 4, and biological replicates = 3.

D p21 protein normalized to control tubulin across two independent experiments with fresh samples correlated with log2 *CDKN1A* RNA expression. We assessed p21 protein expression by western blot in nine cell lines; error bars represent SD. Pearson correlation was assessed in PRISM.

E Cells that were halted in G1 using a double thymidine block led to a decrease in sensitivity to lurbinectedin, and to the combination of lurbinectedin and berzosertib. Here, cells treated with berzosertib, lurbinectedin, and the combination without thymidine are normalized to thymidine-free and untreated control, and cells treated with berzosertib, lurbinectedin, and the combination along with thymidine are normalized to thymidine-alone control cells. NCI-H841 (SCLC cell line chosen largely due to not being sensitive to loss of viability upon thymidine arrest) cells underwent a double-thymidine block-enforcing G1 arrest. Cells were treated with ±lurbinectedin 1 nM ± 2 μM berzosertib ± enforcement of thymidine arrest and collected after 72 h, replicates = 4, n = 3, P-values indicate unpaired two-tailed Student's t-test in PRISM, error bars are representative of SD.

F p21 overexpression in DMS 114 cells led to a decrease in lurbinectedin–berzosertib combination synergy; DMS 114 cells were treated with mock transfection or with overexpression of Flag-tagged wild-type p21 (Addgene Plasmid #16240). Cells were split at 1,000 cells/well into 384-well plates after 24 h and then treated the following day with lurbinectedin and berzosertib in a 10 × 6 matrix format, cells were collected after 72 h, and synergy was assessed; technical replicates = 4, biological replicates = 2.

 © 2023 The Authors

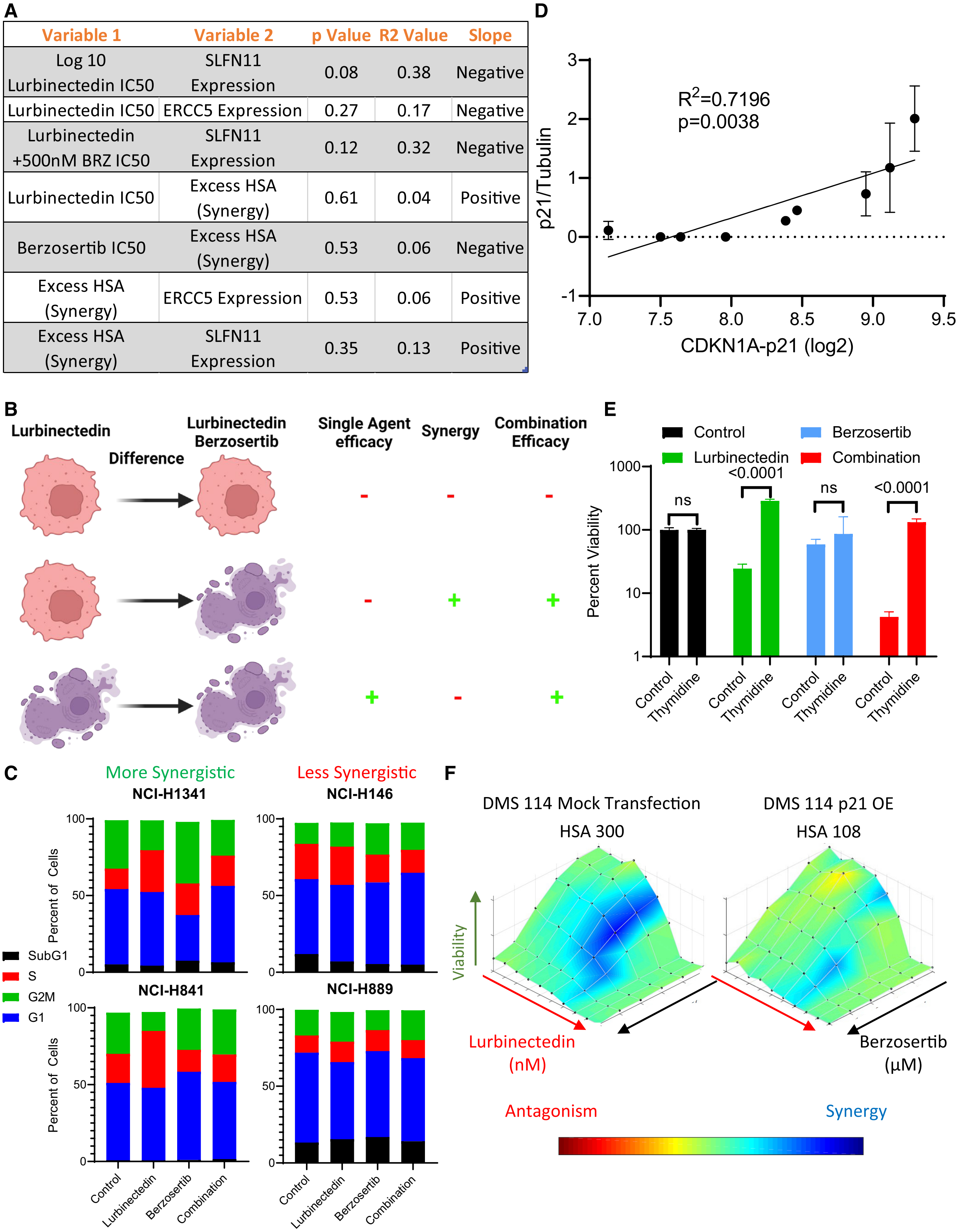

Variable 1	Variable 2	p Value	R2 Value	Slope
Log 10 Lurbinectedin IC50	SLFN11 Expression	0.08	0.38	Negative
Lurbinectedin IC50	ERCC5 Expression	0.27	0.17	Negative
Lurbinectedin +500nM BRZ IC50	SLFN11 Expression	0.12	0.32	Negative
Lurbinectedin IC50	Excess HSA (Synergy)	0.61	0.04	Positive
Berzosertib IC50	Excess HSA (Synergy)	0.53	0.06	Negative
Excess HSA (Synergy)	ERCC5 Expression	0.53	0.06	Positive
Excess HSA (Synergy)	SLFN11 Expression	0.35	0.13	Positive

Figure EV3.

Figure EV4. TP53 deficient cells may be more sensitive to lurbinectedin-berzosertib combination.

A Lurbinectedin treatment caused a decrease in p21 in NCI-H899 (p53 mutant) while it caused an increase in p21 in NCI-H146 (p53 wild-type) as assessed by immunoblotting. Cells were treated with ±1 nM lurbinectedin for 6 h and collected.

B, C The ATM inhibitor KU60019 increased synergy and increased combination efficacy of lurbinectedin and berzosertib in NCI-H146 cells (p53 wild = type) while not increasing synergy or combination efficacy to as great as an extent in NCI-H889 (p53 mutant). H889 and NCI-H146 cells were treated with a combination of lurbinectedin and berzosertib in an 8 × 8 matrix format ±2 μM KU60019 for 72 h, technical replicates = 4 and biological replicates = 2.

D, E PDX 592484 displayed greater synergy of lurbinectedin with berzosertib as compared to the PDX 456648 as displayed by the greater increase in lurbinectedin sensitivity with the addition of berzosertib. Patient-derived xenograft organoids were dissociated and plated in 384-well plates and treated with lurbinectedin ± 500 nM berzosertib for 72 h, technical replicates = 3, and biological replicates = 2.

F, G The more synergistic organoid model (PDX 592484) expressed less *CDKN1A* (p21) and *ERCC5* (XPG) potentially explaining the not only increased synergy but also decreased overall efficacy of lurbinectedin even in the presence of berzosertib as compared to PDX 456648. Patient-derived xenograft organoids samples were assessed using RNA seq, data are shown as TMM FPKM; PDX456648 technical replicates = 6 and PDX 592484 technical replicates = 4 for RNA seq; we utilized unpaired Student's *t*-tests in PRISM to compare groups.

H Neuroendocrine scores inversely associated with synergy in our nine cell lines, with NE cells displaying decreased synergy as compared to non-NE cells. Neuroendocrine scores and SCLC subtype clustering data (ASCL1, NEUROD1, POU2F3, and YAP1) are publicly available at https://discover.nci.nih.gov/rsconnect/cellminercdb, and HSA was determined as described previously. Pearson correlation was determined using PRISM.

© 2023 The Authors

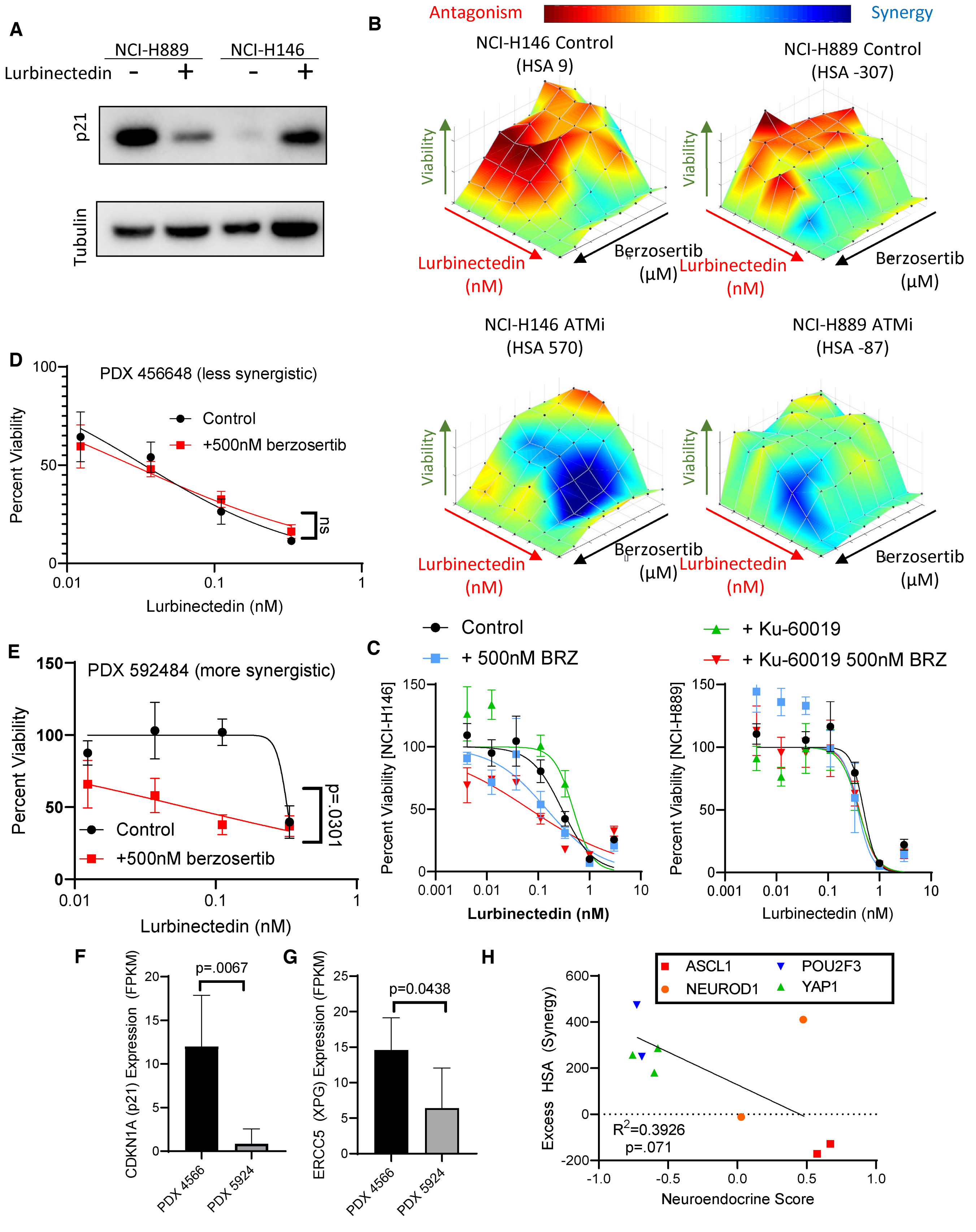
A
NCI-H889
NCI-H146
Lurbinectedin - + - +
p21
Tubulin
B
Antagonism
Synergy
NCI-H146 Control
(HSA 9)
NCI-H889 Control
(HSA -307)
NCI-H146 ATMi
(HSA 570)
NCI-H889 ATMi
(HSA -87)
Viability
Lurbinectedin
(nM)
Berzosertib
(μM)
C
Control
+ 500nM BRZ
+ Ku-60019
+ Ku-60019 500nM BRZ
Percent Viability [NCI-H146]
Percent Viability [NCI-H889]
Lurbinectedin (nM)
D
PDX 456648 (less synergistic)
Control
+500nM berzosertib
ns
Percent Viability
Lurbinectedin (nM)
E
PDX 592484 (more synergistic)
Control
+500nM berzosertib
p=.0301
Percent Viability
Lurbinectedin (nM)
F
CDKN1A (p21) Expression (FPKM)
p=.0067
PDX 4566
PDX 5924
G
ERCC5 (XPG) Expression (FPKM)
p=0.0438
PDX 4566
PDX 5924
H
ASCL1
NEUROD1
POU2F3
YAP1
Excess HSA (Synergy)
R2=0.3926
p=.071
Neuroendocrine Score

Figure EV4.

Figure EV5. Berzosertib and lurbinectedin efficacy and synergy *in vivo*.

A In the PDX-06 model, lurbinectedin appeared to induce p-CHK1 activation which was reduced with berzosertib cotreatment (quantified in Fig 4B). PDX-06 tumors were collected 24 h after being dosed with the indicated drugs. We assessed targets in the ATR (yellow/gold), ATM (green), and DNA-PK (blue) damage repair pathways as well as γH2AX (DNA damage marker).

B Lurbinectedin and berzosertib were less effective in the more aggressive mouse model of SCLC PDX-03 as compared to PDX-06 (Fig 4A). PDX-03 was treated the same as PDX-06 with lurbinectedin (0.18 mg/kg IV, with an increased volume of 200 μl vs. 100 μl) and berzosertib (20 mg/kg IP) in a format mirroring our clinical trial with topotecan/berzosertib in SCLC, lurbinectedin day 1, and berzosertib days 2 and 5 of a 7-day cycle. To approximate efficacy across an experiment with significant toxicity, we assessed growth rate as tumor size at animal death due to either toxicity or progression of tumor minus tumor volume at date of initiation, divided by days between initiation and final assessment of tumor for a growth rate.

C The PDX-03 model was more aggressive than the PDX-06 model as demonstrated by comparing rate of growth for the control arms for both experiments. PDX-06 mice = 10, PDX-03 mice = 11, and an unpaired two-tailed Student's *t*-test was used to compare rates.

D Lurbinectedin and the combination were less toxic in PDX-03 as compared to PDX-06, likely due to the increased volume for dosing of lurbinectedin, however, several mice still required sacrifice. Mouse body weights corresponding to Appendix Fig S7B, mice which required sacrifice due to toxicity (body weight loss or other) are quantified to the right in red.

E The combination of lurbinectedin and berzosertib overall appeared to decrease replication and increase markers of cell death. We assessed markers of replication (ki-67) and cell death (cleaved caspase-3 and necrosis) as assessed by IHC and H&E staining from tumors after collection from all mice possibly from our PDX-03 efficacy study (Appendix Fig S7B). Cleaved caspase and ki-67 are reported as percent positive of cells, while necrosis is reported as percent area. Lines represent median of groups as do numbers above.

F, G Similar to Fig 4D to determine differences in synergy with different scheduling regiments of lurbinectedin and berzosertib, we treated NCI-446 cells in a 10 × 10 matrix format; technical replicates = 3, biological replicates = 1. All groups were treated for 24 h with lurbinectedin, while group 1 was pretreated with berzosertib, group 2 was cotreated with berzosertib, group 3 was co- and posttreated with berzosertib, and group 4 was posttreated with berzosertib for 24 h. At the end of 4 days, cells were collected and synergy was assessed across the matrixes. We determined that group 3 (co- and posttreatment) displayed the greatest degree of synergy, mirroring our results with DMS 114 cells in Fig 4D. We also assessed synergy of DMS 114 cells and our PDX-06 cell line in a 12 × 3 matrix format, technical replicates = 4 and biological replicates = 2, and determined that PDX-06 cells appeared to have less synergy than DMS 114 cells (F) and were more sensitive to lurbinectedin (G).

H The most effective schedule in the DMS 114 xenograft model was treating with lurbinectedin and berzosertib cotreated and posttreated (lurbinectedin day 1, berzosertib days 1, 2) mirroring our *in vitro* results. The most effective scheduling model is shown in Fig 4F, error bars are representative of SEM, mice = 10 per group.

I In the DMS 114 mouse model of SCLC, lurbinectedin day 1 and berzosertib days 1 and 2 were nontoxic. Mouse body weights from the PDX experiment are demonstrated in Appendix Fig S1H. The number of mice sacrificed due to toxicity in each group is also displayed.

J, K Concentrations of berzosertib and lurbinectedin in mouse plasma were assessed in mice utilizing mass spectrometry after retro-orbital collection of blood at the indicated time points after dosing with lurbinectedin (0.18 mg/kg intravenous) and berzosertib (50 mg/kg oral), mice = 5 per group; error bars are representative of SD.

 © 2023 The Authors

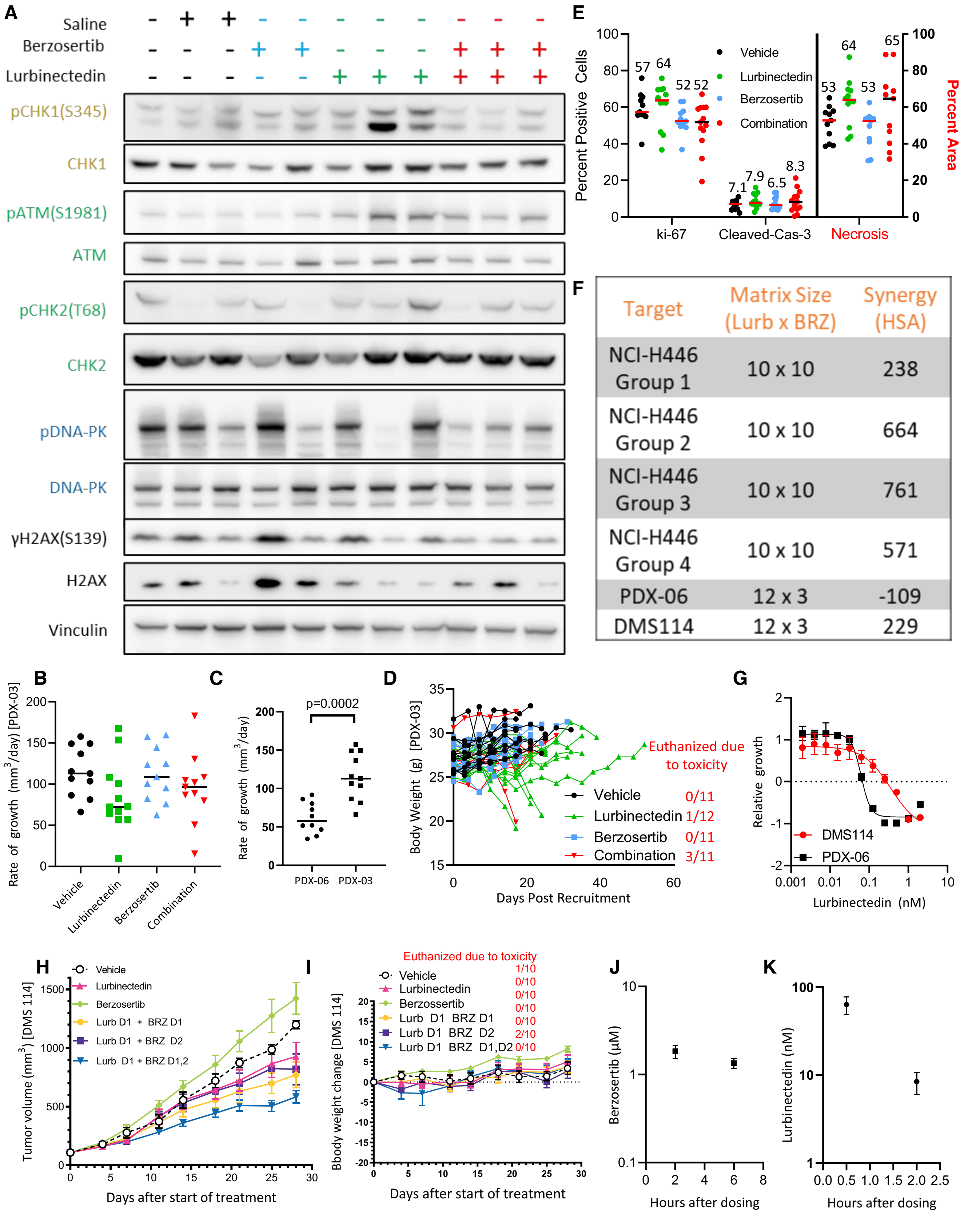
A
Saline
Berzosertib
Lurbinectedin
pCHK1(S345)
CHK1
pATM(S1981)
ATM
pCHK2(T68)
CHK2
pDNA-PK
DNA-PK
γH2AX(S139)
H2AX
Vinculin
E
Percent Positive Cells
Percent Area
Vehicle
Lurbinectedin
Berzosertib
Combination
ki-67
Cleaved-Cas-3
Necrosis
57 64 52 52
7.1 7.9 6.5 8.3
53 64 53 65
F
Target
Matrix Size (Lurb x BRZ)
Synergy (HSA)
NCI-H446 Group 1 10 x 10 238
NCI-H446 Group 2 10 x 10 664
NCI-H446 Group 3 10 x 10 761
NCI-H446 Group 4 10 x 10 571
PDX-06 12 x 3 -109
DMS114 12 x 3 229
B
Rate of growth (mm3/day) [PDX-03]
Vehicle
Lurbinectedin
Berzosertib
Combination
C
Rate of growth (mm3/day)
p=0.0002
PDX-06
PDX-03
D
Body Weight (g) [PDX-03]
Euthanized due to toxicity
Vehicle 0/11
Lurbinectedin 1/12
Berzosertib 0/11
Combination 3/11
Days Post Recruitment
G
Relative growth
DMS114
PDX-06
Lurbinectedin (nM)
H
Tumor volume (mm3) [DMS 114]
Vehicle
Lurbinectedin
Berzosertib
Lurb D1 + BRZ D1
Lurb D1 + BRZ D2
Lurb D1 + BRZ D1,2
Days after start of treatment
I
Bbody weight change [DMS 114]
Euthanized due to toxicity
Vehicle 1/10
Lurbinectedin 0/10
Berzossertib 0/10
Lurb D1 BRZ D1 0/10
Lurb D1 BRZ D2 0/10
Lurb D1 BRZ D1,D2 2/10
0/10
Days after start of treatment
J
Berzosertib (μM)
Hours after dosing
K
Lurbinectedin (nM)
Hours after dosing

Figure EV5.

ATR inhibition augments the efficacy of lurbinectedin in small cell lung cancer

Christopher W. Schultz *et al* 2023

APPENDIX

Table of Contents:

Appendix Figure S1 Legend: 2

Appendix Figure S1: 3

Appendix Figure S2 Legend: 4

Appendix Figure S2: 5

Appendix Fig. S1:

A-C) In two SCLC cell lines lurbinectedin treatment increased γH2AX induction which was reduced with berzosertib co-treatment. γH2AX induction was assessed using immunofluorescence in DMS 114 (A) and NCI-H446 (B) cells treated +/- 1 nM lurbinectedin +/- 2µM berzosertib for 6 hours. Quantification for NCI-H446 is displayed in (C) and quantification for DMS 114 is in Figure 2C. Quantification is from 100-150 cells per treatment, with error bars indicating SEM, comparisons were made using an unpaired two-tailed student's t test in PRISM, biological replicates = 3.

D) Lurbinectedin treatment caused an increase in γH2AX levels in 2/4 cell lines, while berzosertib treatment either alone or in combination with lurbinectedin causes a decrease in γH2AX. Cell lines were treated +/- 1 nM lurbinectedin +/- 2µM berzosertib for 6 hours. Quantification is indicative of γH2AX normalized to H3 levels vs control levels for each cell line. The green cell lines here were later determined to have greater synergy, while the red cell lines indicate cell lines later determined to be less synergistic.

E) Berzosertib caused an abnormal division phenotype, while only the combination of lurbinectedin and berzosertib caused a significant number of chromosomal breaks. DMS 114 cells were treated +/- 1 nM lurbinectedin +/- 2 µM berzosertib for 6 hours followed by metaphase spread analysis assessing chromosome breaks and abnormal metaphase spread. Data are indicative of three separate experiments with 150-200 metaphases assessed per experiment, error bars = SD, p values indicate unpaired two-tailed student's t tests in PRISM.

F) Representative images of normal (left) and abnormal chromosomal spread (right) as caused by berzosertib in representative images from Fig. S2D.

G) Barasertib re-capitulated the abnormal division phenotype caused by berzosertib in DMS 114 cells. DMS 114 cells were treated with 300nM barasertib for 6 hours with the final two 2 hours of treatment also including nocodazole. Cell chromosomal structure and integrity was then assessed using mitotic spread.

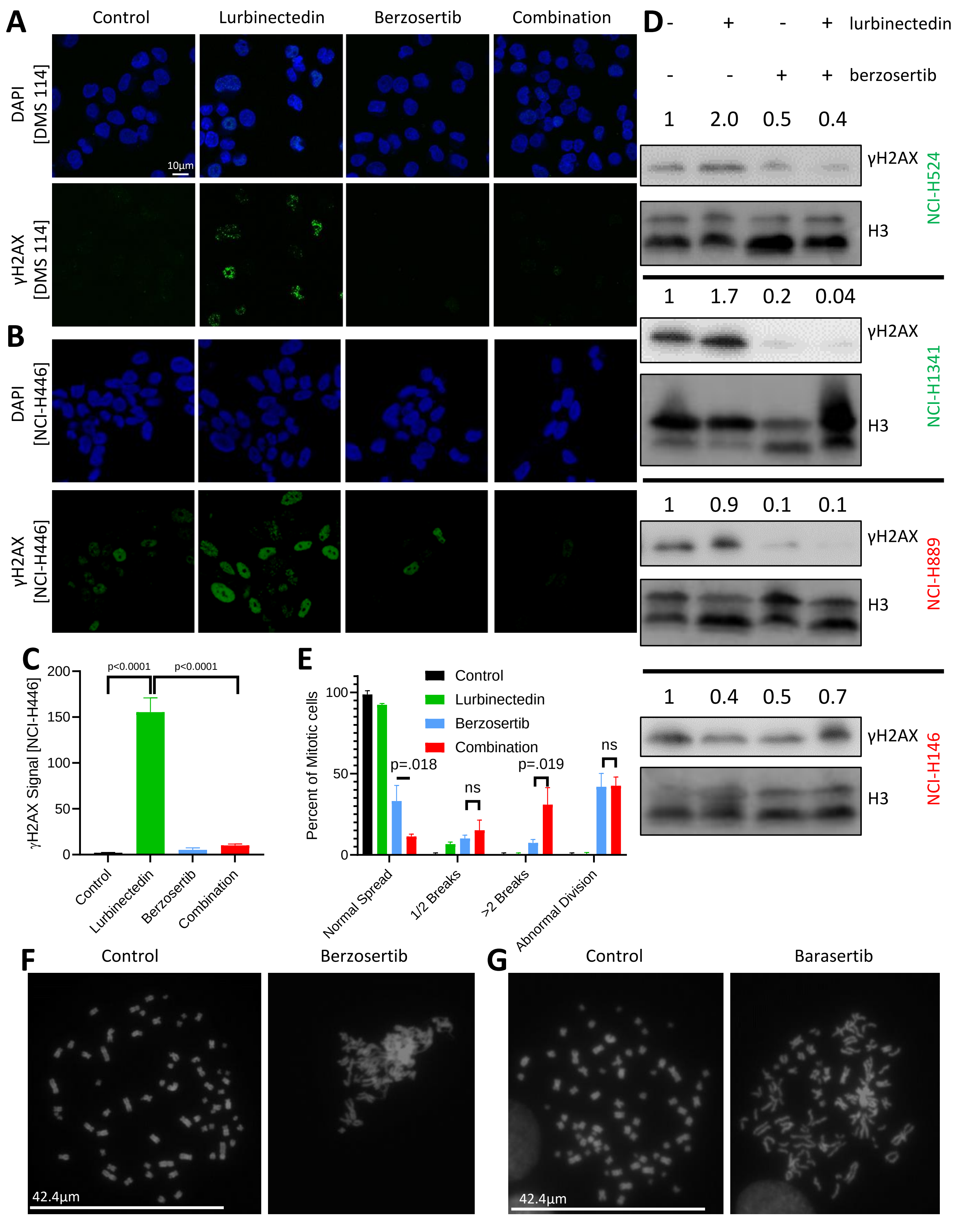
A
Control
Lurbinectedin
Berzosertib
Combination
DAPI [DMS 114]
γH2AX [DMS 114]
10µm
B
DAPI [NCI-H446]
γH2AX [NCI-H446]
C
γH2AX Signal [NCI-H446]
p<0.0001
p<0.0001
Control
Lurbinectedin
Berzosertib
Combination
D
lurbinectedin
berzosertib
- + - +
- - + +
1 2.0 0.5 0.4
γH2AX
H3
NCI-H524
1 1.7 0.2 0.04
γH2AX
H3
NCI-H1341
1 0.9 0.1 0.1
γH2AX
H3
NCI-H889
1 0.4 0.5 0.7
γH2AX
H3
NCI-H146
E
Percent of Mitotic cells
Control
Lurbinectedin
Berzosertib
Combination
p=.018
ns
p=.019
ns
Normal Spread
1/2 Breaks
>2 Breaks
Abnormal Division
F
Control
Berzosertib
42.4µm
G
Control
Barasertib
42.4µm

Appendix Fig. S2:

A) *ASCL1* expression positively correlated with NE score in patient tumor samples (positive control) while *MYC* expression negatively correlated with NE score in the same dataset. In the NCI dataset of 100 SCLC patients NE score was analyzed using SSGSEA. Pearson correlation was assessed in PRISM.

B) Similar to figure 3H, high MYC family member expression negatively associated with *CDKN1A* (p21) expression in a CDX SCLC datasets, and trended towards negative association in a smaller CDX dataset indicating that Non-NE differentiation may reduce *CDKN1A* (p21) expression. *MYC*, *MYCL* and *MYCN* expression were z scored (within each database) and the max MYC family member z score expression was determined for each sample. Those samples which were greater than one standard deviation above average were considered to be high MYC family member expressing. Comparisons between groups were made using unpaired two-tailed student's t tests in PRISM.

C,D) *MYC* expression was assessed against *CDKN1A* and NE differentiation score in a panel of 67 SCLC cell lines and they were found to be inversely correlated. Pearson correlation was assessed in PRISM.

E) Out of 50 hallmark pathway genesets, MYC Targets V1 was the most predictive of resistance to first line platinum-based chemotherapy followed by DNA repair and MYC Targets V2. We assessed enrichment of Hallmark pathways in 100 SCLC patient samples using SSGSEA, platinum resistance was defined as a response to carboplatin etoposide treatment of <90 days.

F,G) MYC high patients were more likely to be platinum resistant. 100 patient samples/records from the NCI SCLC dataset were assessed. Platinum sensitivity was defined as recurrence or relapse < 90 days after completion of first line platinum-based chemotherapy. Patients were stratified based on median (F) and quartile (G) MYC targets V1 enrichment. We utilized Fisher's exact t tests to determine significance for these samples in PRISM. Hazard ratios were calculated using odd's ratio in PRISM for F and G were 0.2130 and 0.1830 for low MYC vs high MYC respectively indicating a ~5 fold decreased risk for resistance to platinum based therapy in low MYC patients.

H) In our panel of 9 cell lines the combination efficacy (IC_{50} of lurbinectedin + 500 nM BRZ) negatively correlated with MYC Targets V2 enrichment. MYC Targets V2 was calculated using SSGSEA, Pearson correlation was assessed in PRISM.

I,J) In both the NCI dataset of SCLC 100 (I) and the George patient Dataset (J) NE score negatively correlated with ERCC5 expression. Pearson correlation was assessed in PRISM.

K) Both lurbinectedin and combination treatment were toxic in a large proportion of mice. Mouse body weights corresponding to figure 4A, mice which required sacrifice due to toxicity (body weight loss or other) are quantified in the top right in red.

L) We compared variables assessed with RNA-sequencing data of tumor models PDX-06 vs PDX-03 as well as aggressiveness as approximated by the time to reach $1000mm^3$ for initially transplanted fresh tumor samples from patients (P0). These data are part of initial screening data for our PDX models generated and represent 1 RNA seq sample from 1 passage 1 tumor from each model. NE score was calculated using SSGSEA.

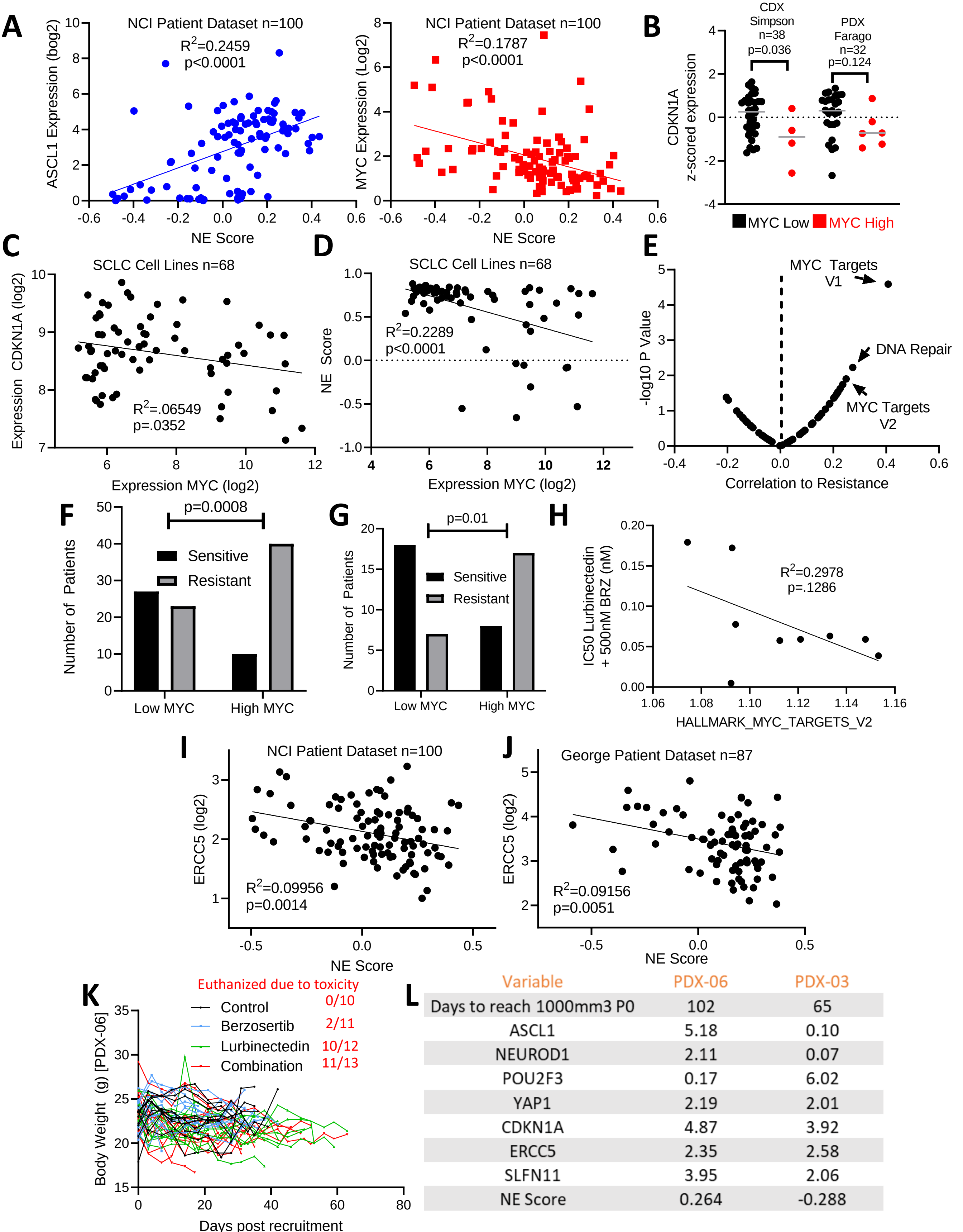

Variable	PDX-06	PDX-03
Days to reach 1000mm3 P0	102	65
ASCL1	5.18	0.10
NEUROD1	2.11	0.07
POU2F3	0.17	6.02
YAP1	2.19	2.01
CDKN1A	4.87	3.92
ERCC5	2.35	2.58
SLFN11	3.95	2.06
NE Score	0.264	-0.288
