# Supplementary material for: ATR inhibition augments the efficacy of lurbinectedin in small‐cell lung cancer
Source: EMBO Mol Med. 2023 Jul 25;15(8):e17313. doi: 10.15252/emmm.202217313 (PMC10405061; doi:10.15252/emmm.202217313)
Supplement: Supplementary file 8 — Source Data for Figure 1 [file EMMM-15-e17313-s004.zip › Figure 1/C/1_C_README.docx]

All combination screening data is publicly available at https://matrix.ncats.nih.gov/. Each unique screen is independently searchable, and a ‘help’ tab provides an overview of methods and a tutorial to aid users as they search this database. These data represent curated data from screen 12831 exploring synergy of lurbinectedin and other agents. We have expanded upon drug targets and added a general class of targets as compared to the publicly available data. This screen was performed in the SCLC cell line NCI-H446. Notably in our work to improve reader clarity positive HSA denotes synergy whereas negative denotes antagonism whereas in the original screen these signs are reversed.

We have included the publicly available data, the help page available at this repository. This data was then curated to make figure C, we have also included Supplemental Tables 1 and 2 here as this is another format of curation for the data represented in C.
