# Supplementary figures and images for: ATR inhibition augments the efficacy of lurbinectedin in small‐cell lung cancer (part 1 of 2)
Source: EMBO Mol Med. 2023 Jul 25;15(8):e17313. doi: 10.15252/emmm.202217313 (PMC10405061; doi:10.15252/emmm.202217313)

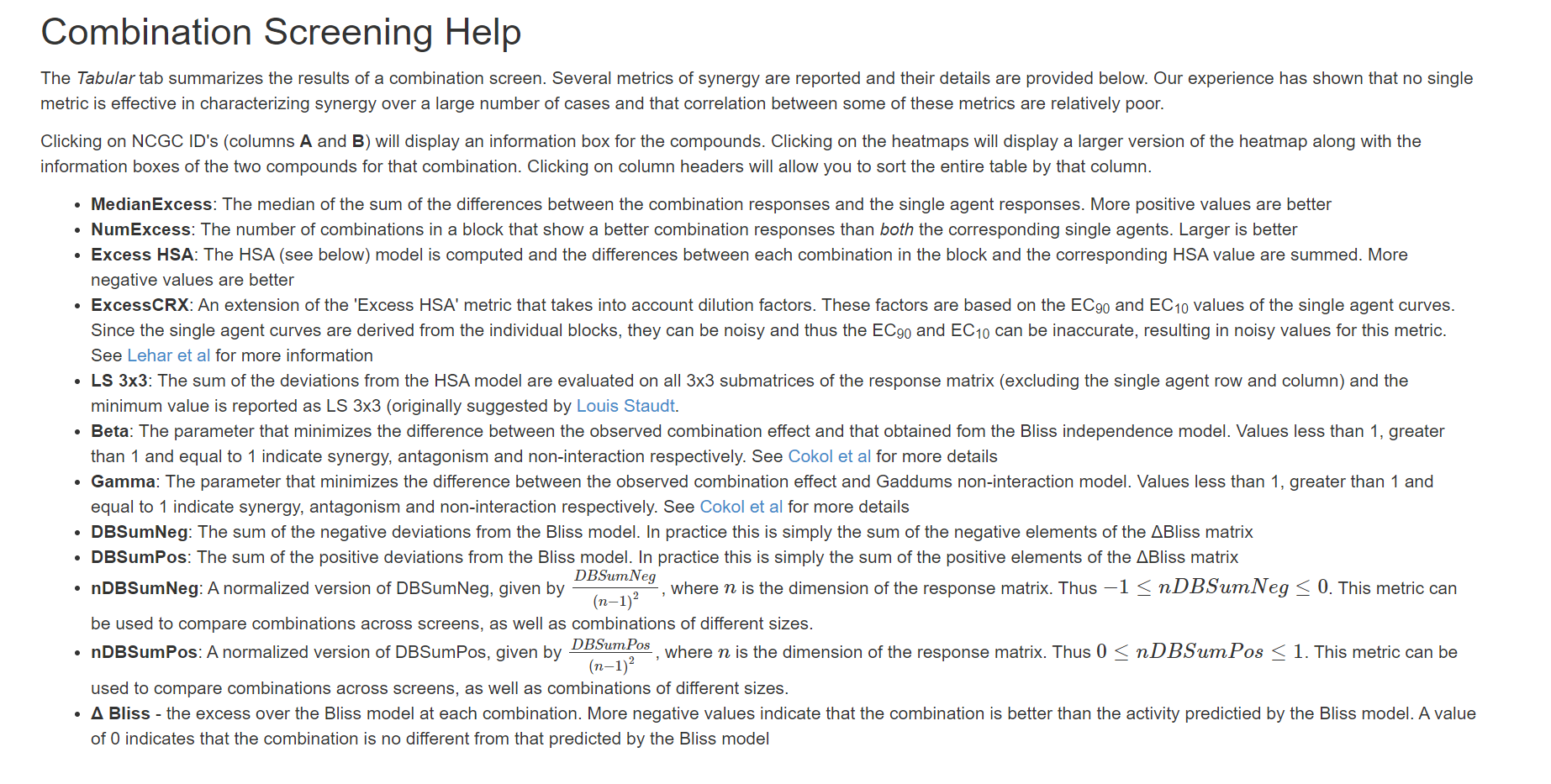

Supplement: Supplementary file 8 — Source Data for Figure 1 [file EMMM-15-e17313-s004.zip › Figure 1/B/3_29_23_Help_file_NCATS.docx]

# Synergy mapped to D-R (HSA)

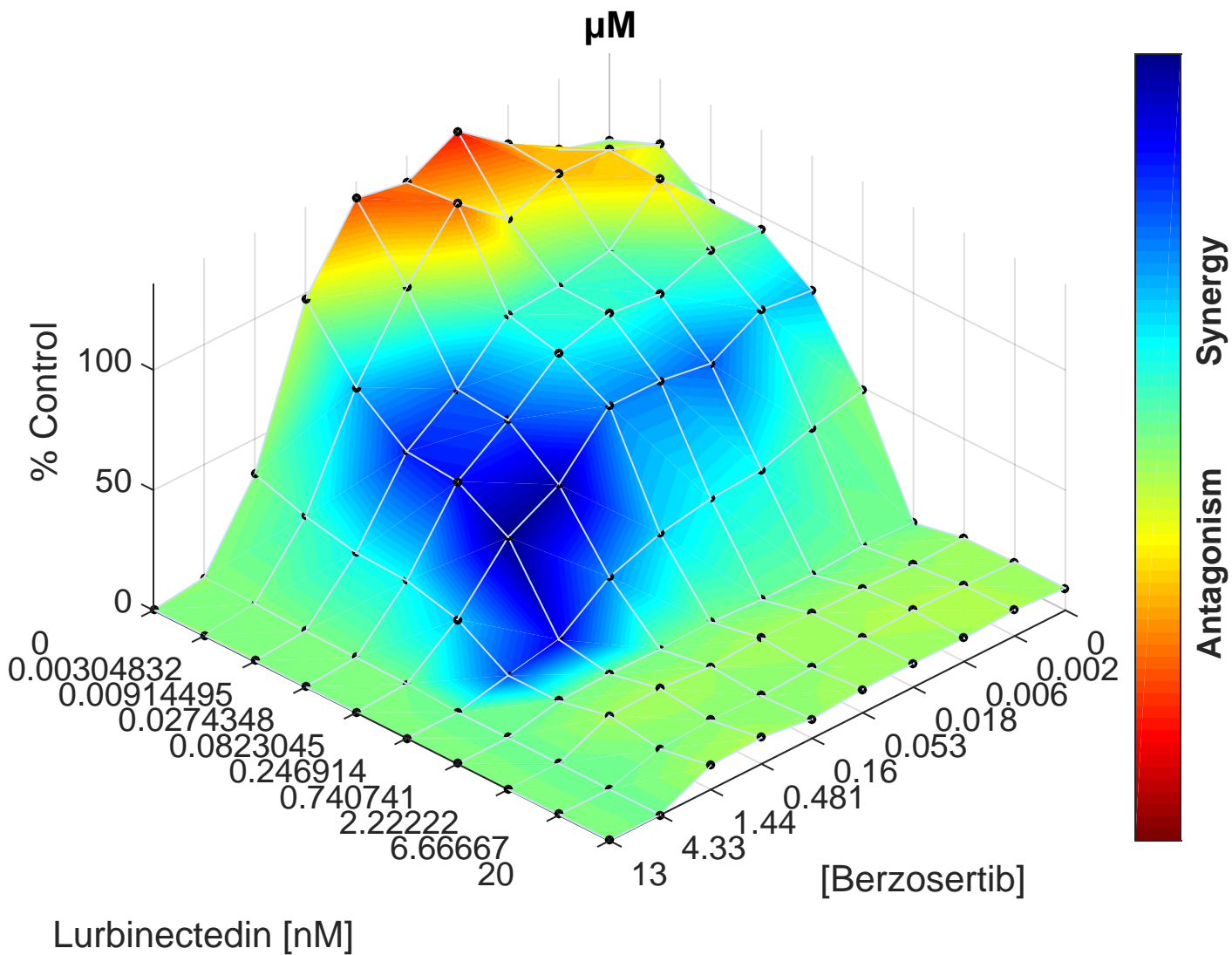

Supplement: Supplementary file 8 — Source Data for Figure 1 [file EMMM-15-e17313-s004.zip › Figure 1/D/Mapped_Surface_HSA_SYN_ANT_μM.pdf]

# Synergy mapped to D-R (HSA)

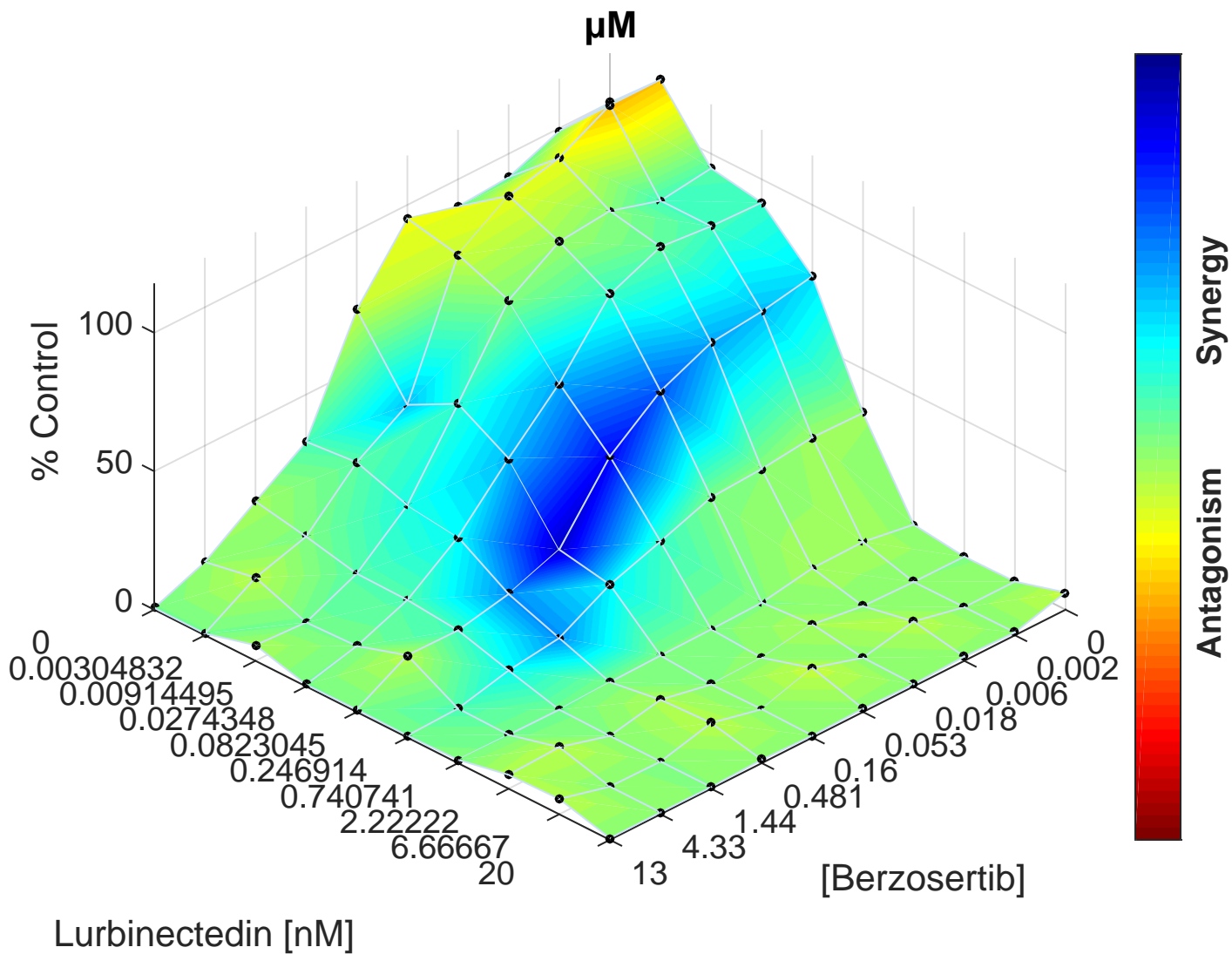

Supplement: Supplementary file 8 — Source Data for Figure 1 [file EMMM-15-e17313-s004.zip › Figure 1/E/Mapped_Surface_HSA_SYN_ANT_μM.pdf]

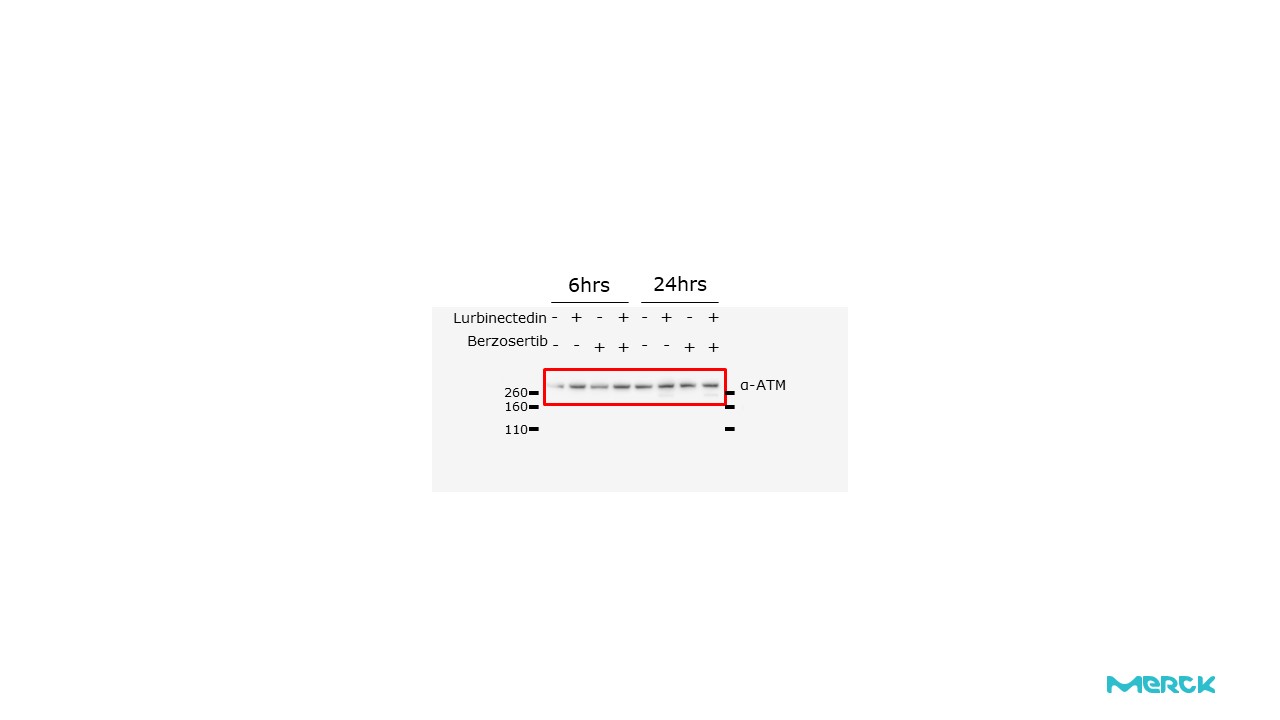

Supplement: Supplementary file 8 — Source Data for Figure 1 [file EMMM-15-e17313-s004.zip › Figure 1/F/Images/ATM.JPG]

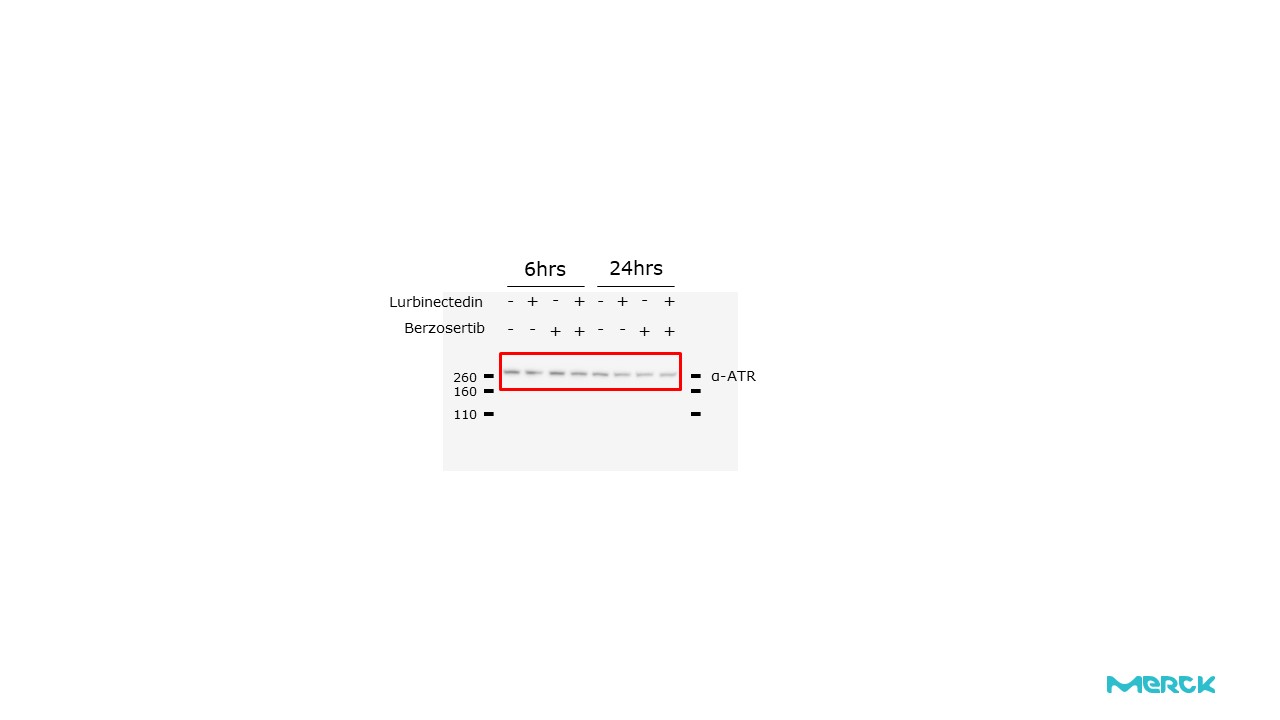

Supplement: Supplementary file 8 — Source Data for Figure 1 [file EMMM-15-e17313-s004.zip › Figure 1/F/Images/ATR.JPG]

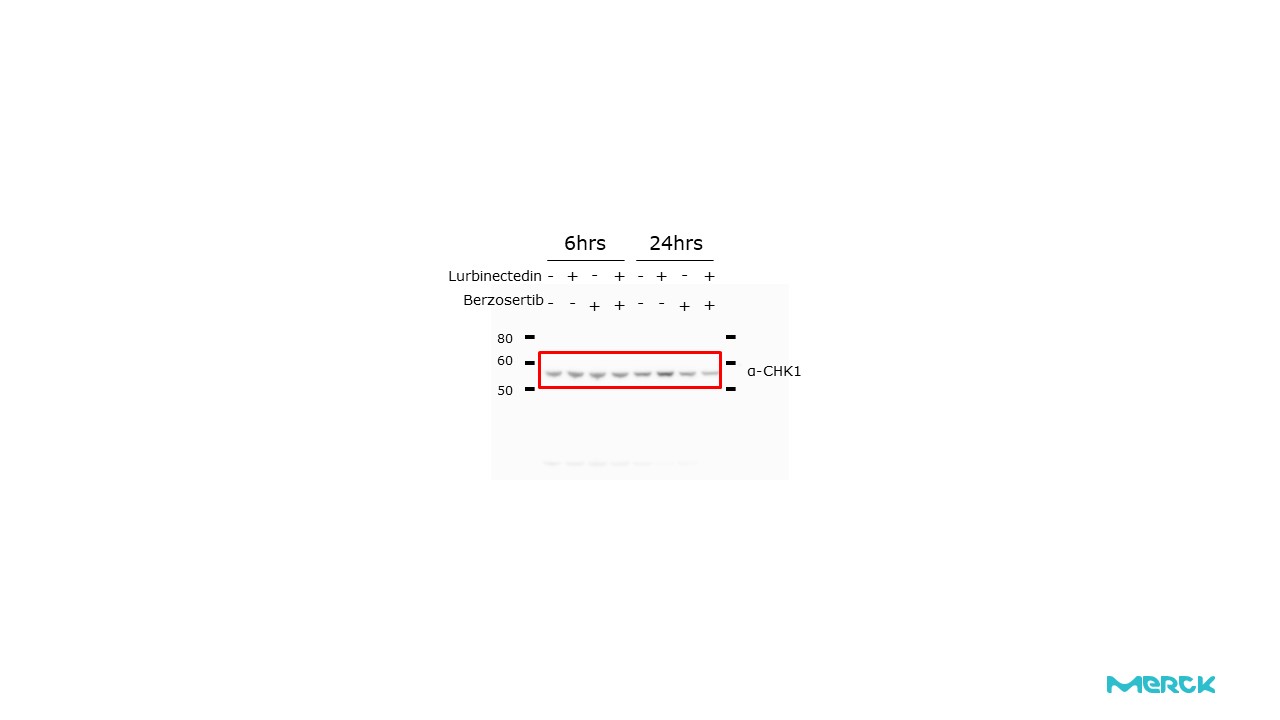

Supplement: Supplementary file 8 — Source Data for Figure 1 [file EMMM-15-e17313-s004.zip › Figure 1/F/Images/CHK1.JPG]

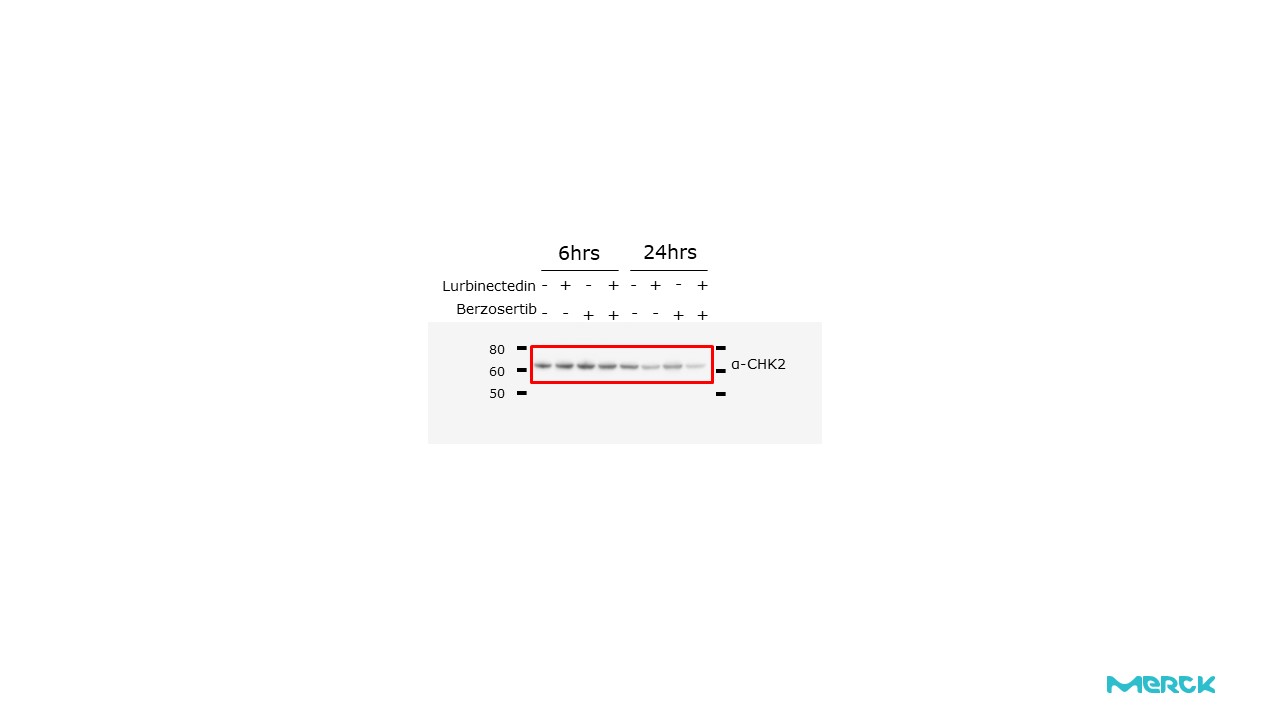

Supplement: Supplementary file 8 — Source Data for Figure 1 [file EMMM-15-e17313-s004.zip › Figure 1/F/Images/CHK2.JPG]

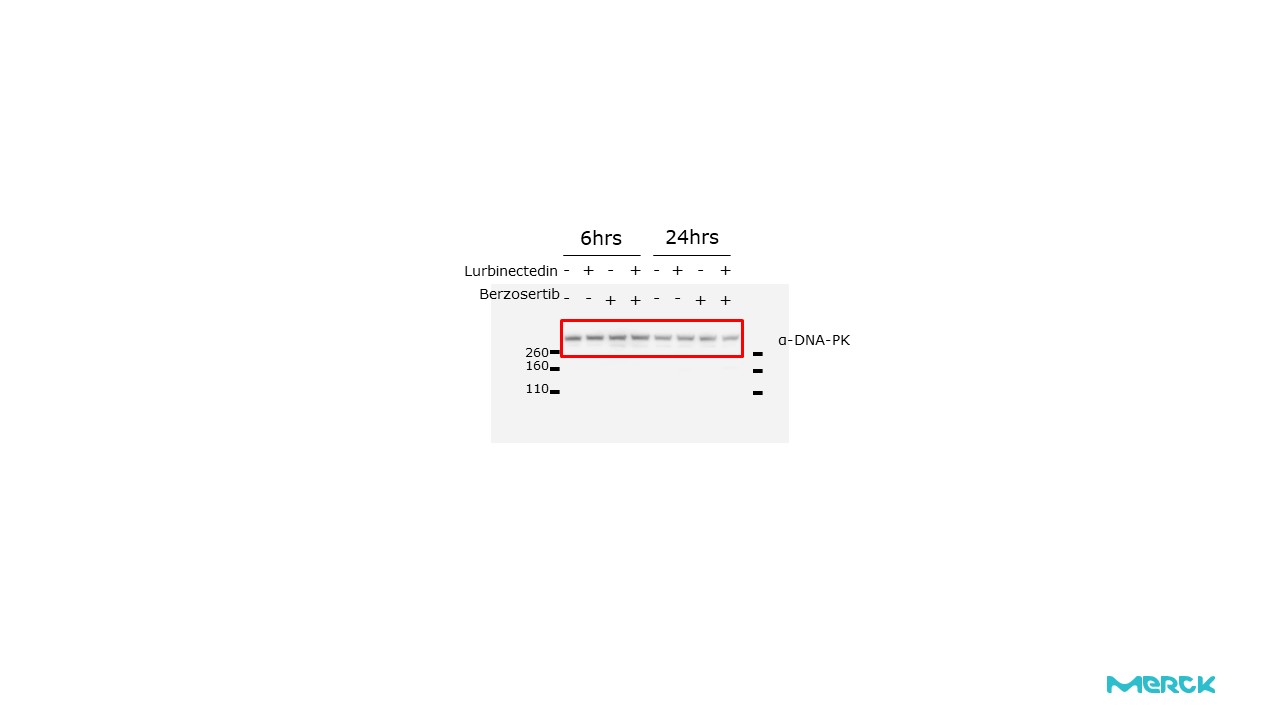

Supplement: Supplementary file 8 — Source Data for Figure 1 [file EMMM-15-e17313-s004.zip › Figure 1/F/Images/DNA-PK.JPG]

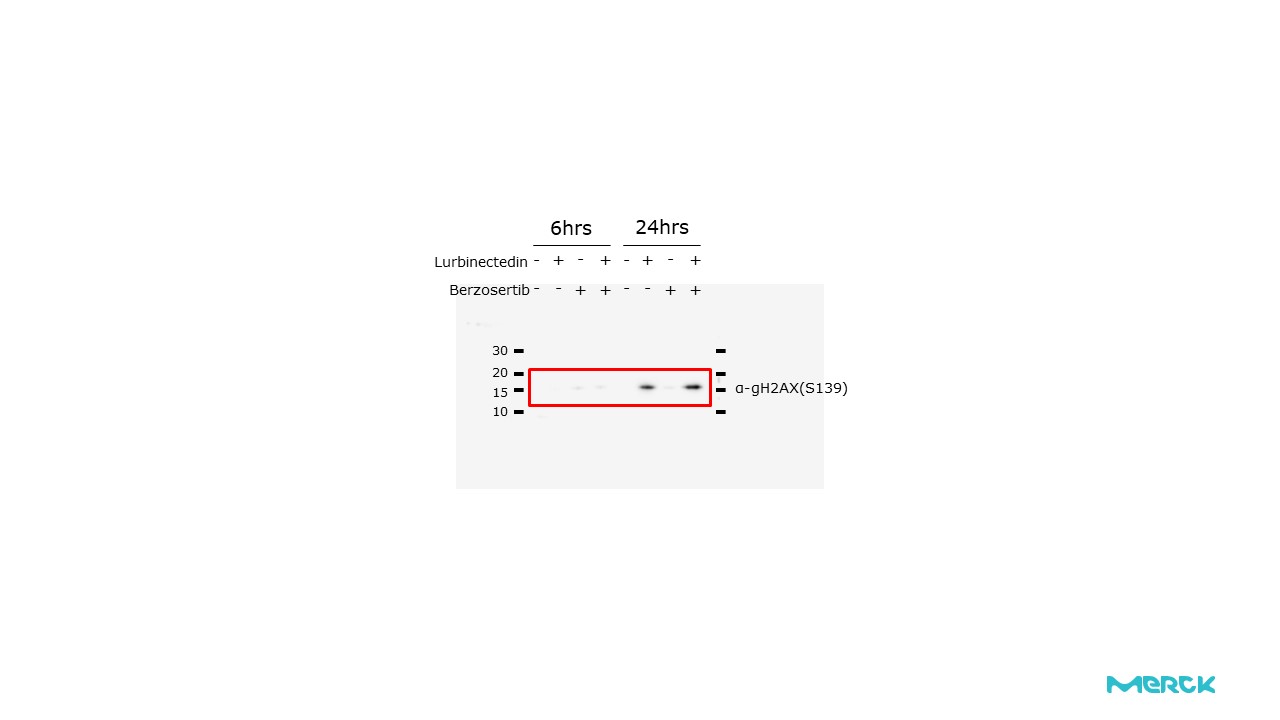

Supplement: Supplementary file 8 — Source Data for Figure 1 [file EMMM-15-e17313-s004.zip › Figure 1/F/Images/gH2AX(S139).JPG]

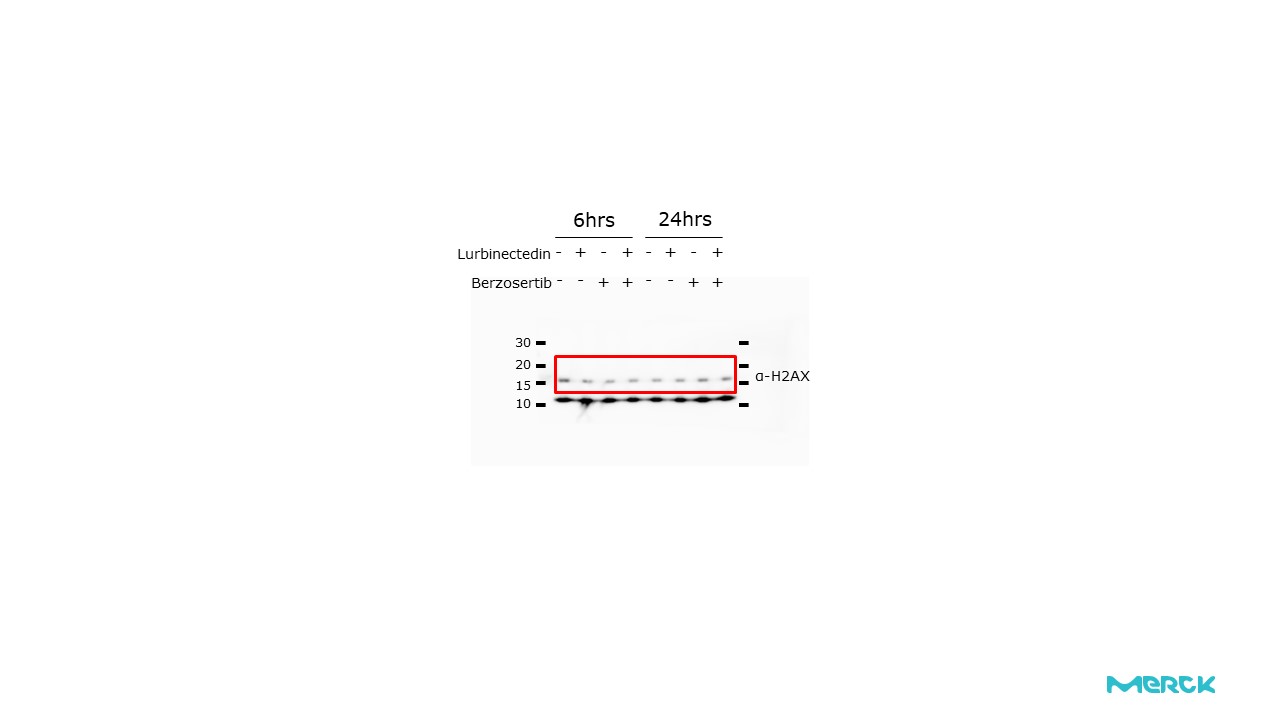

Supplement: Supplementary file 8 — Source Data for Figure 1 [file EMMM-15-e17313-s004.zip › Figure 1/F/Images/H2AX.JPG]

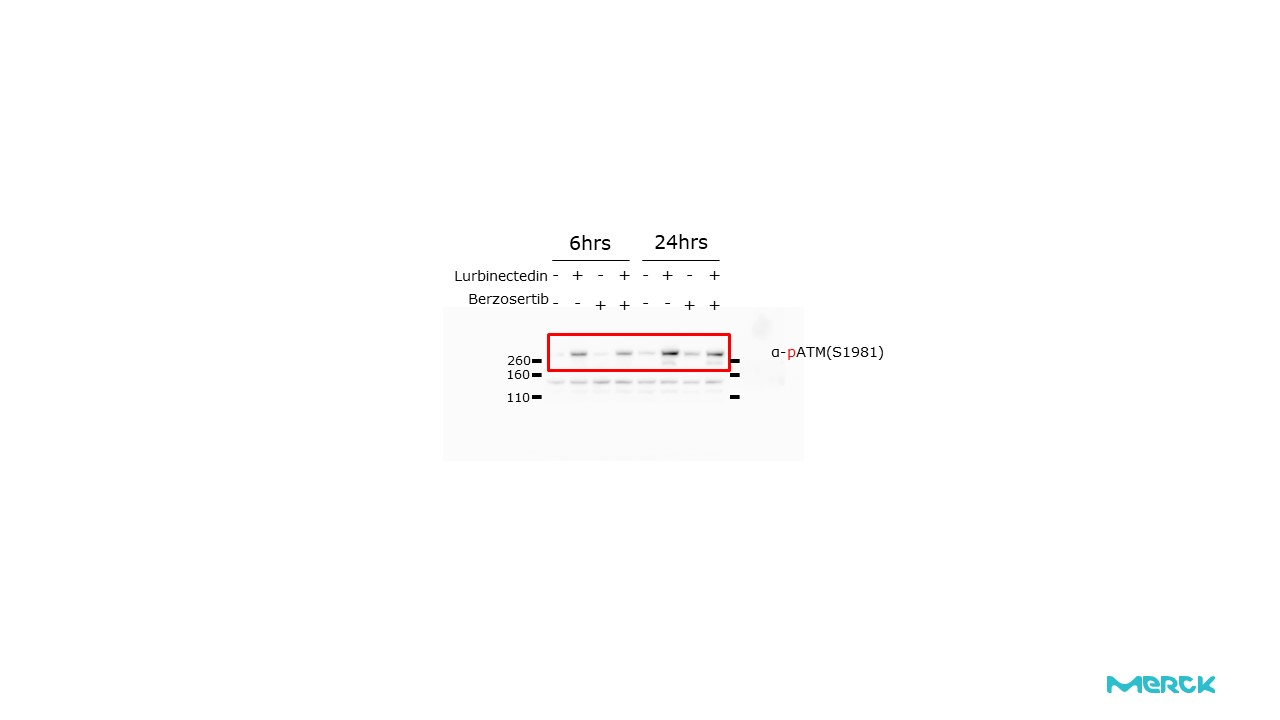

Supplement: Supplementary file 8 — Source Data for Figure 1 [file EMMM-15-e17313-s004.zip › Figure 1/F/Images/pATM(S1981).JPG]

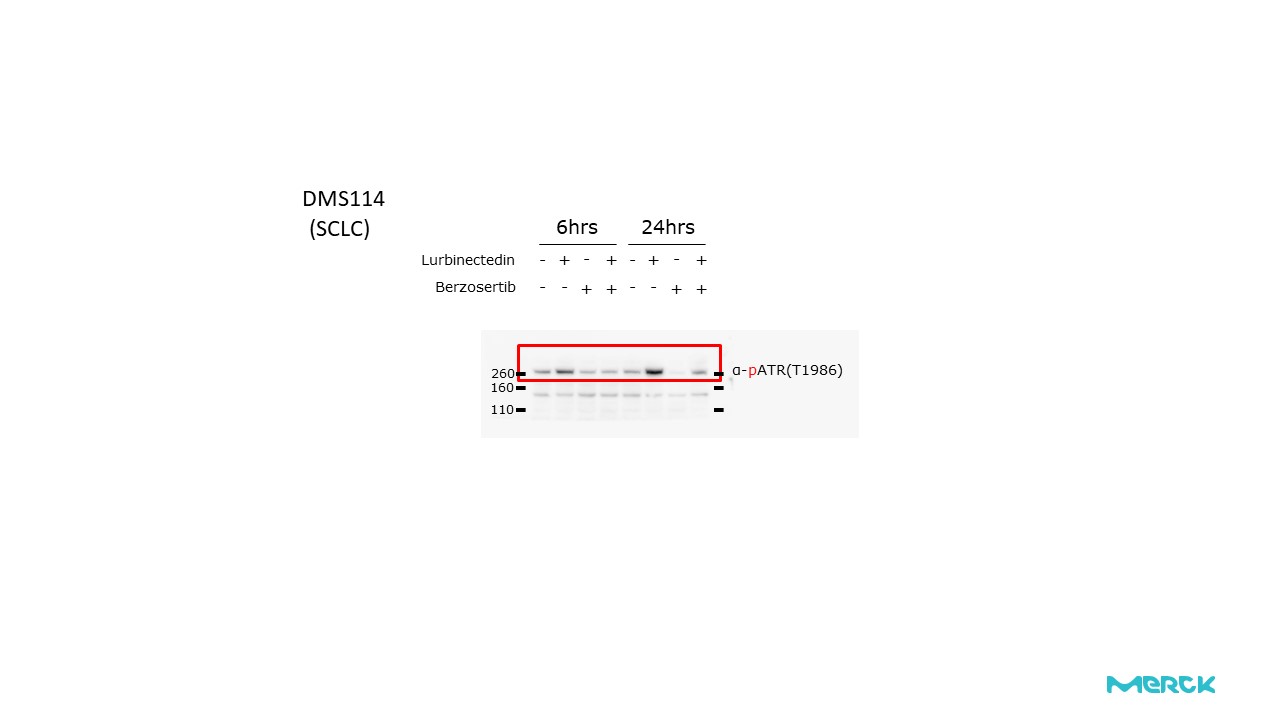

Supplement: Supplementary file 8 — Source Data for Figure 1 [file EMMM-15-e17313-s004.zip › Figure 1/F/Images/pATR(T1986).JPG]

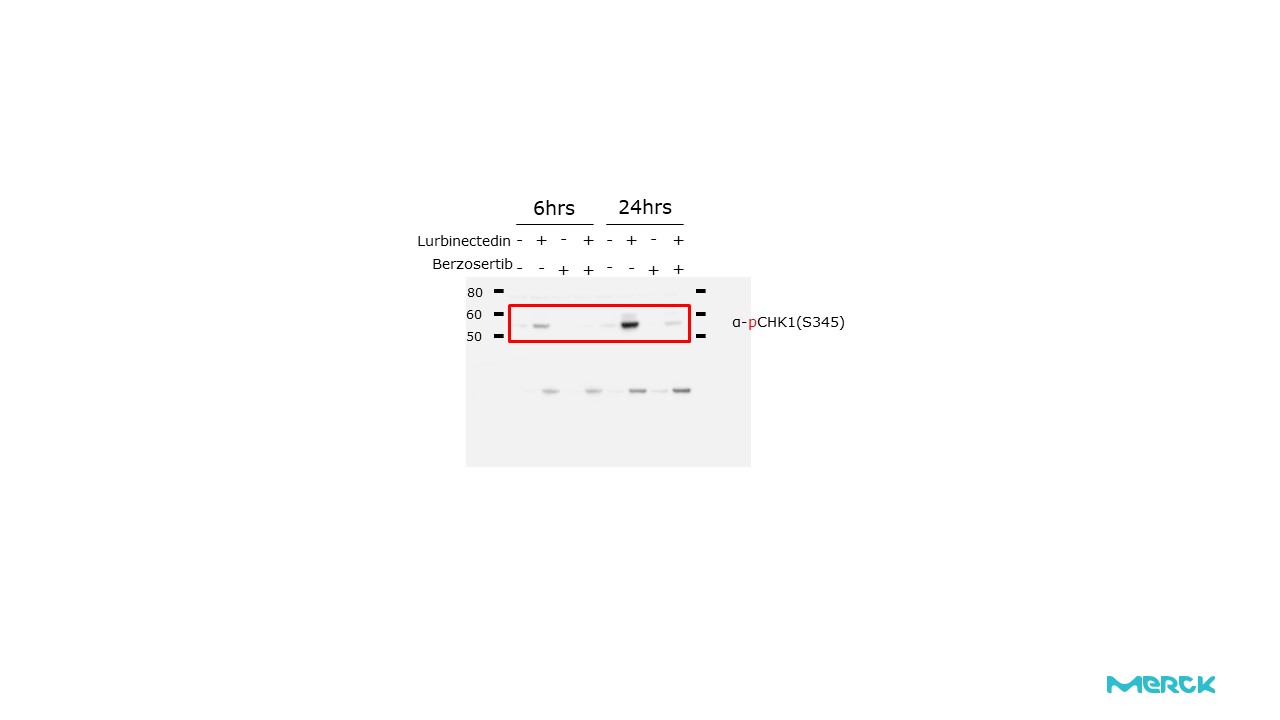

Supplement: Supplementary file 8 — Source Data for Figure 1 [file EMMM-15-e17313-s004.zip › Figure 1/F/Images/pCHK1(S345).JPG]

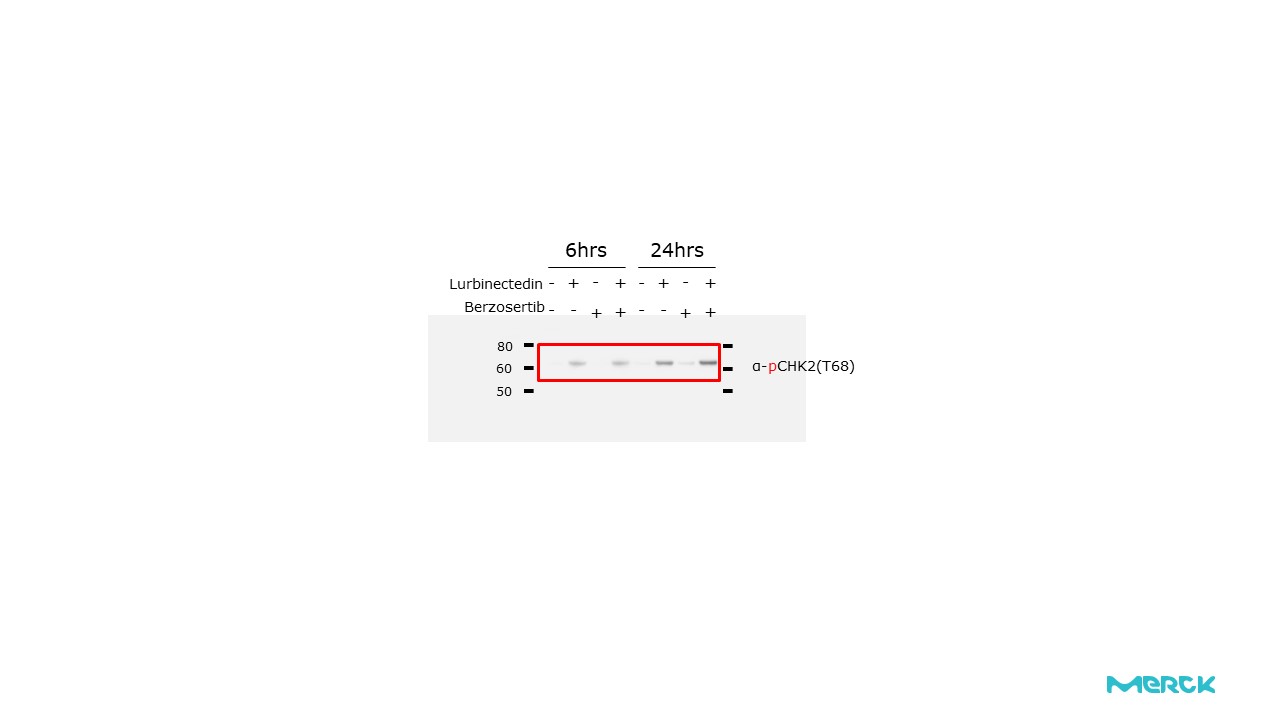

Supplement: Supplementary file 8 — Source Data for Figure 1 [file EMMM-15-e17313-s004.zip › Figure 1/F/Images/pCHK2(T68).JPG]

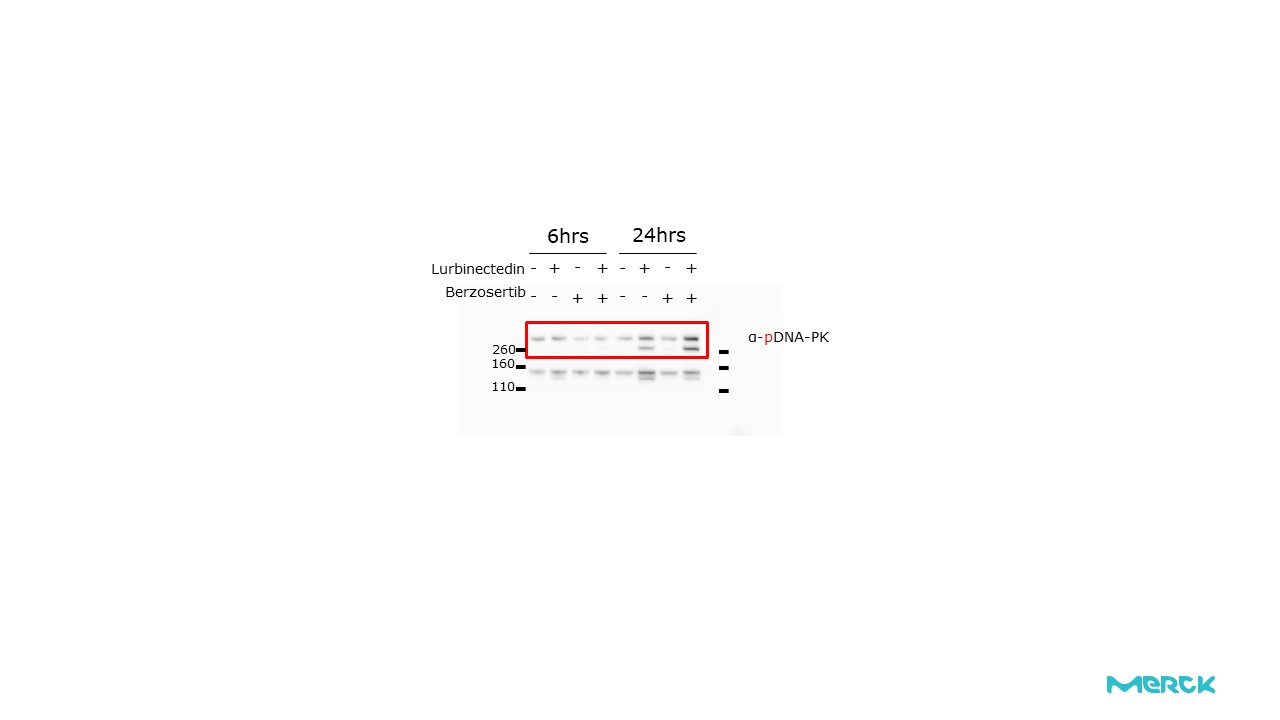

Supplement: Supplementary file 8 — Source Data for Figure 1 [file EMMM-15-e17313-s004.zip › Figure 1/F/Images/pDNA-PK.JPG]

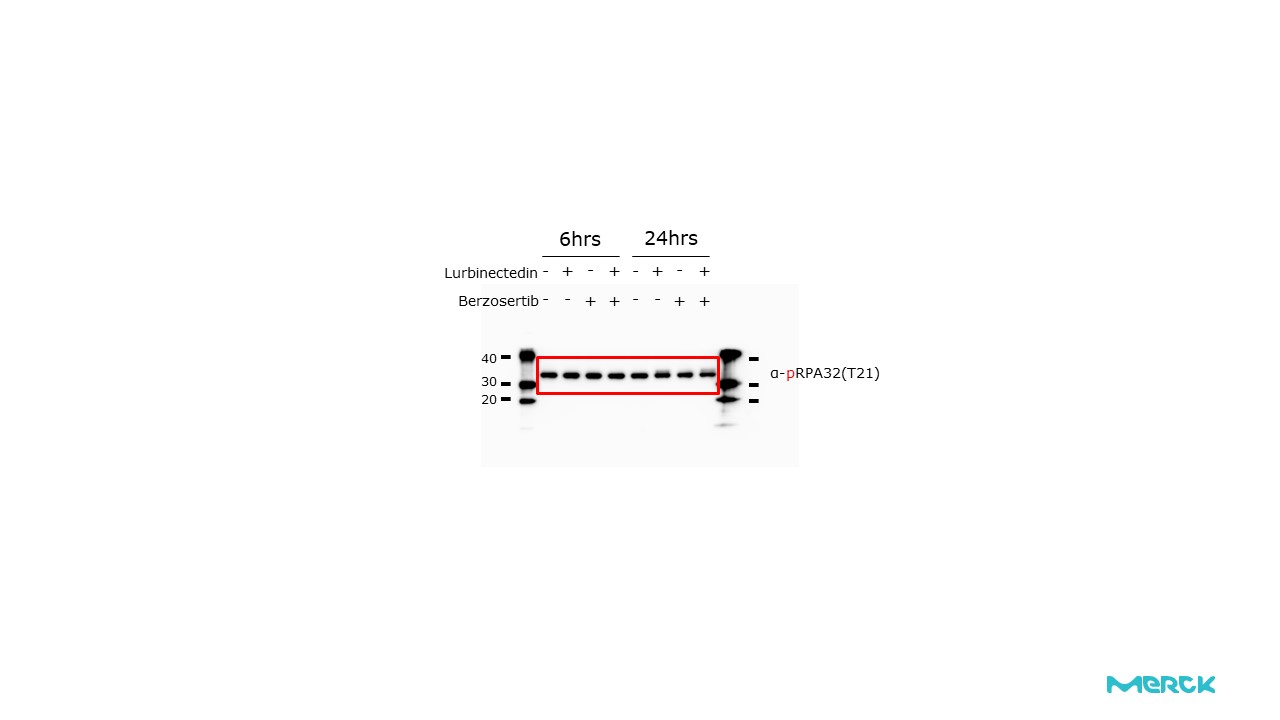

Supplement: Supplementary file 8 — Source Data for Figure 1 [file EMMM-15-e17313-s004.zip › Figure 1/F/Images/pRPA32(T21).JPG]

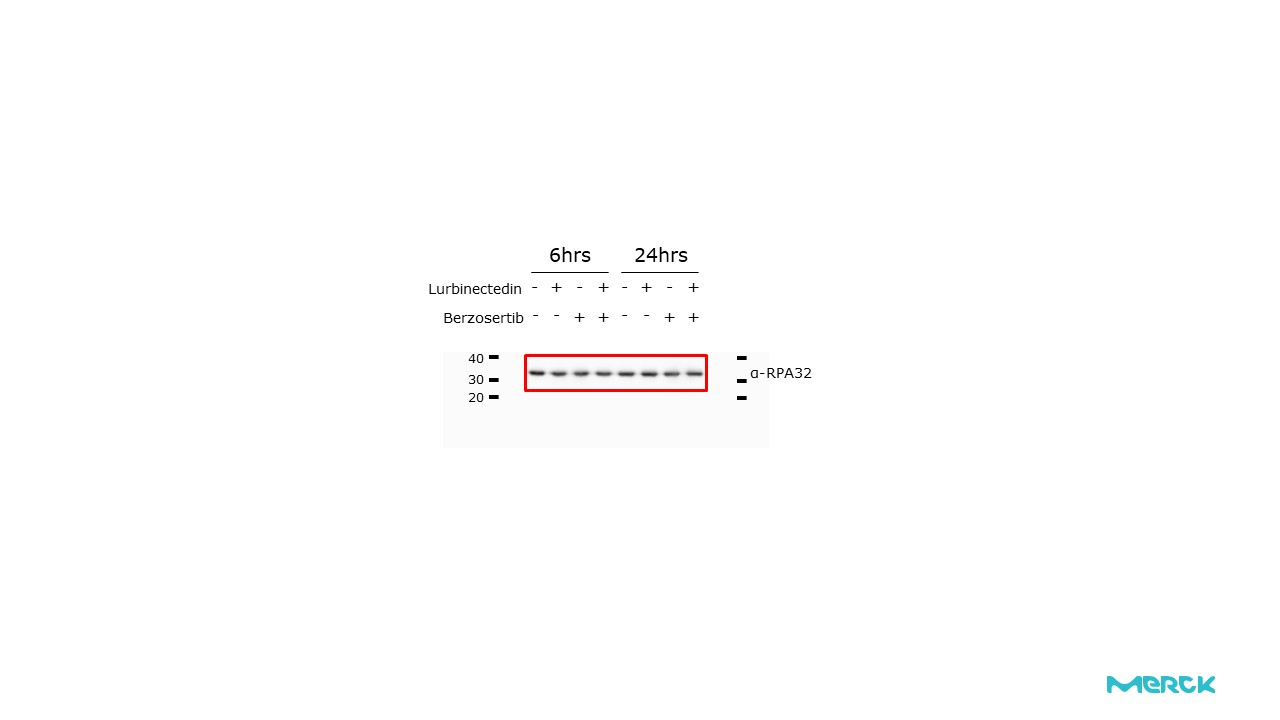

Supplement: Supplementary file 8 — Source Data for Figure 1 [file EMMM-15-e17313-s004.zip › Figure 1/F/Images/RPA32.JPG]

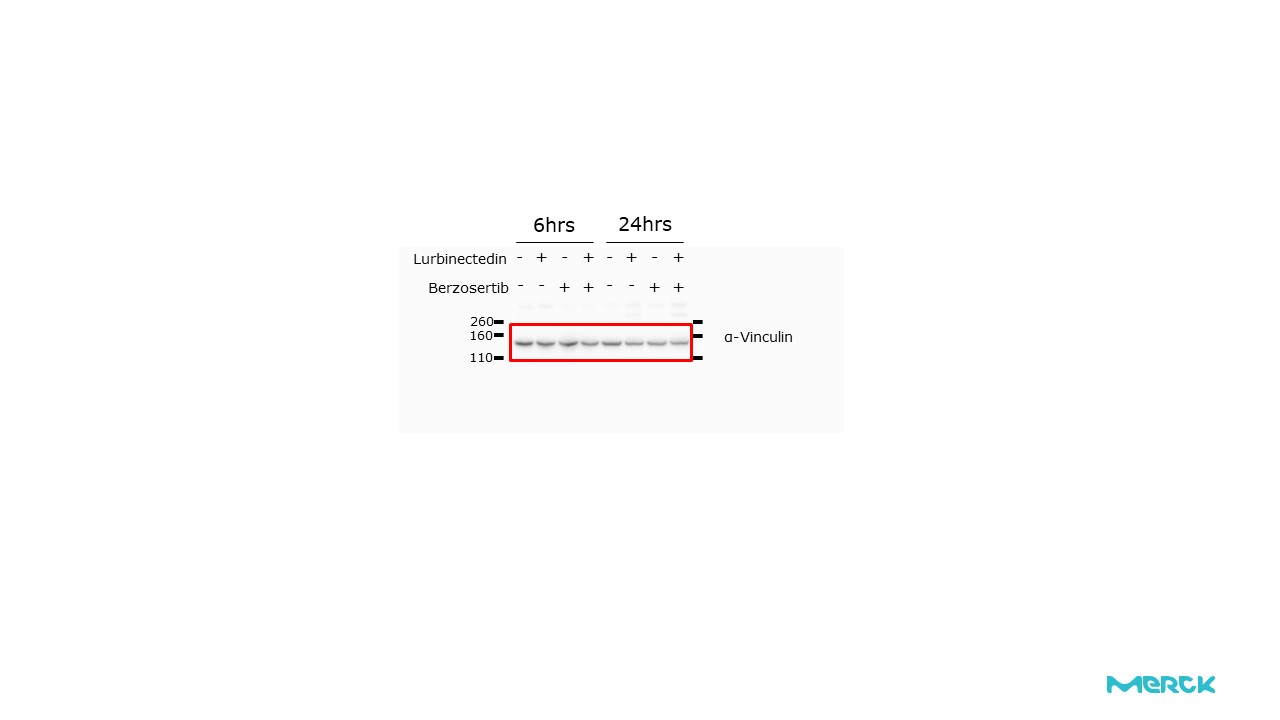

Supplement: Supplementary file 8 — Source Data for Figure 1 [file EMMM-15-e17313-s004.zip › Figure 1/F/Images/Vinculin.JPG]

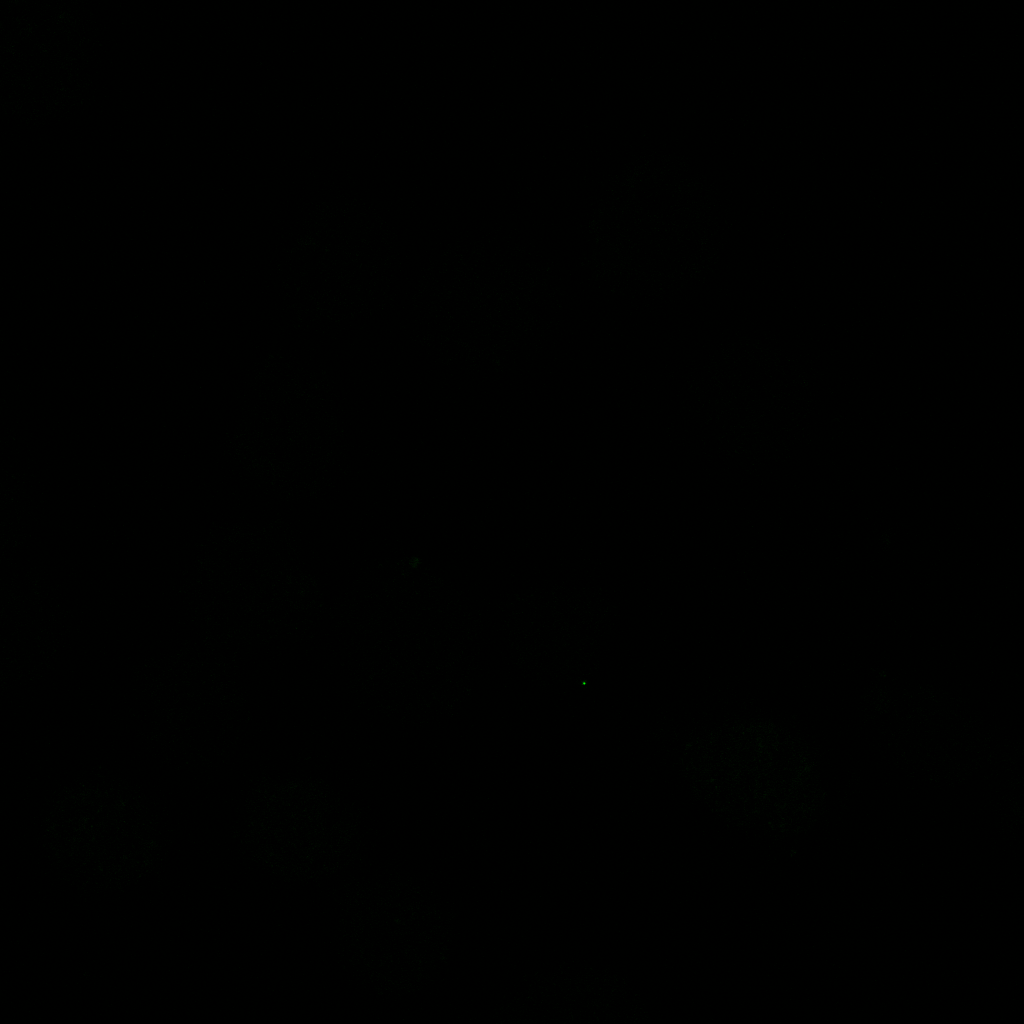

Supplement: Supplementary file 9 — Source Data for Figure 2 [file EMMM-15-e17313-s002.zip › Figure 2/C/Images/DMS114-a-Image Export-01_c1.tif]

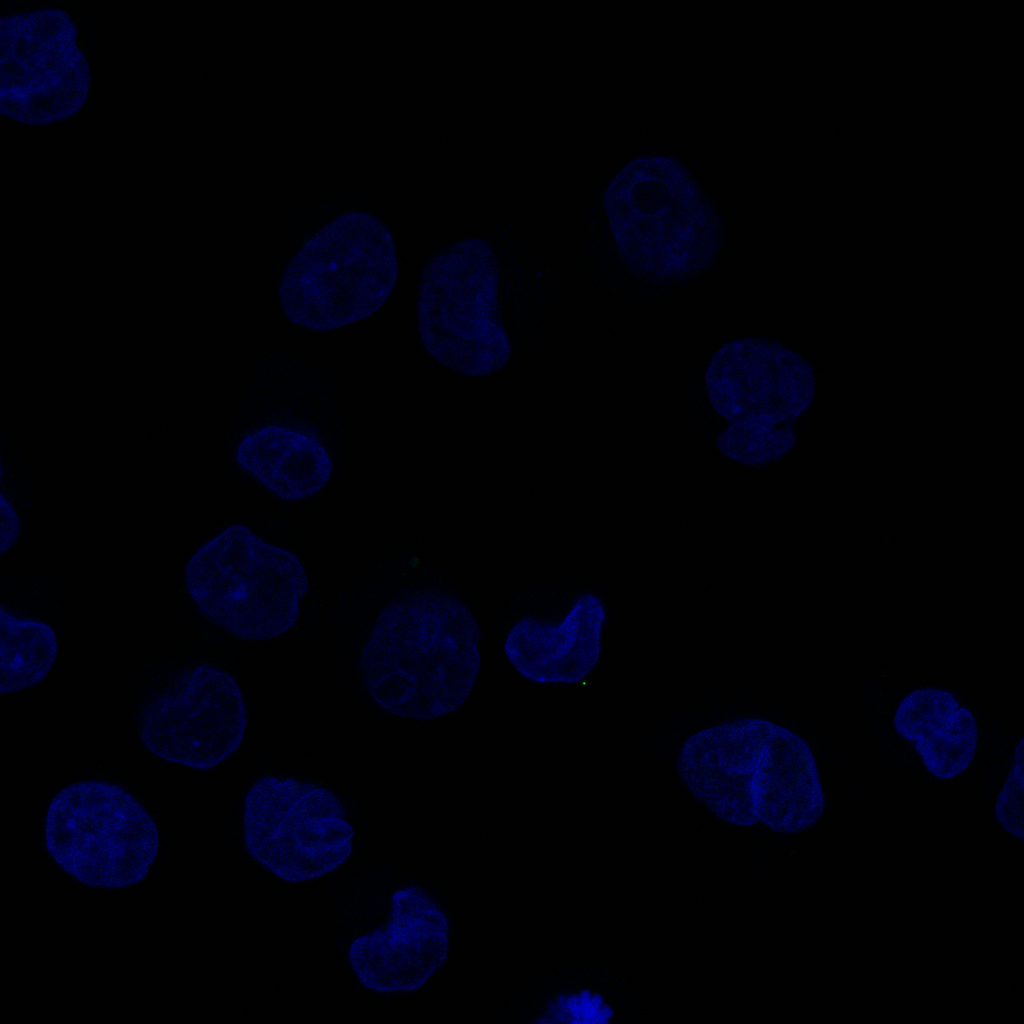

Supplement: Supplementary file 9 — Source Data for Figure 2 [file EMMM-15-e17313-s002.zip › Figure 2/C/Images/DMS114-a-Image Export-01_c1+2.tif]

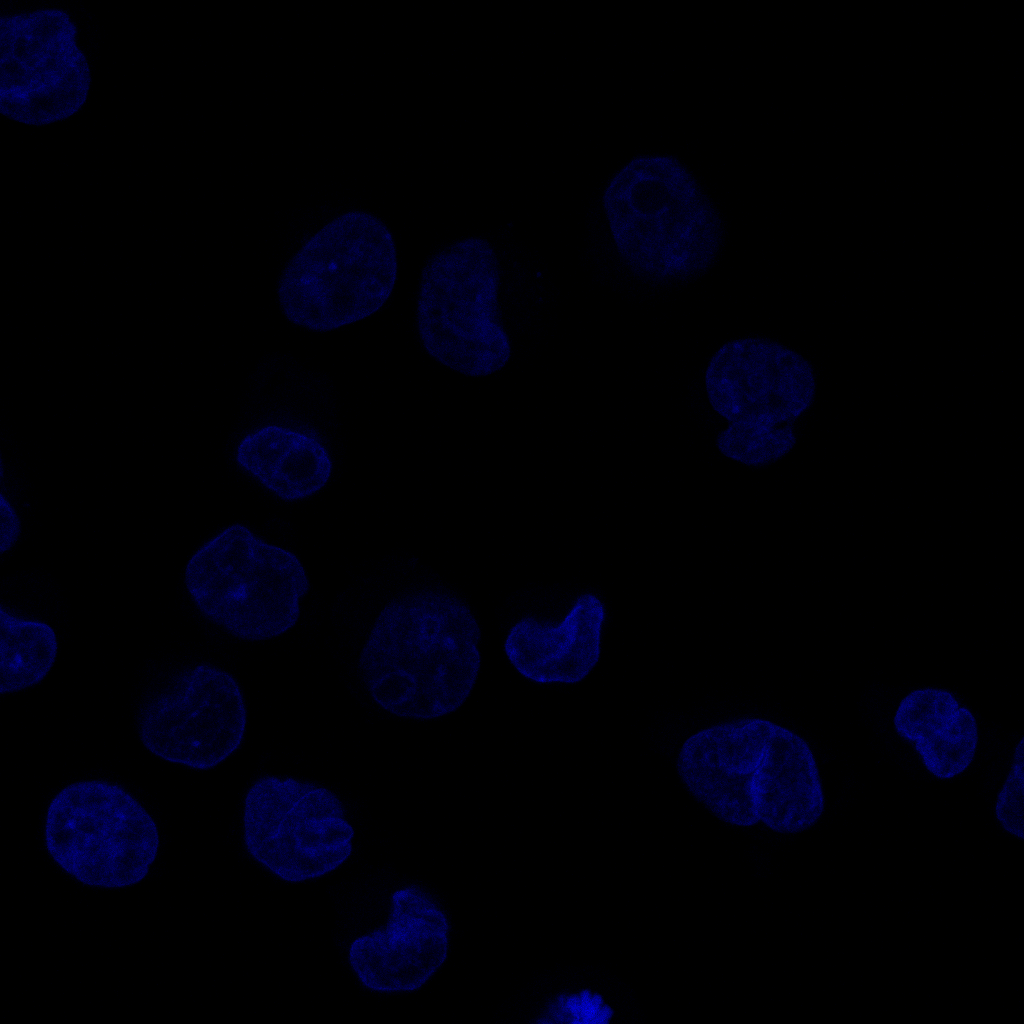

Supplement: Supplementary file 9 — Source Data for Figure 2 [file EMMM-15-e17313-s002.zip › Figure 2/C/Images/DMS114-a-Image Export-01_c2.tif]

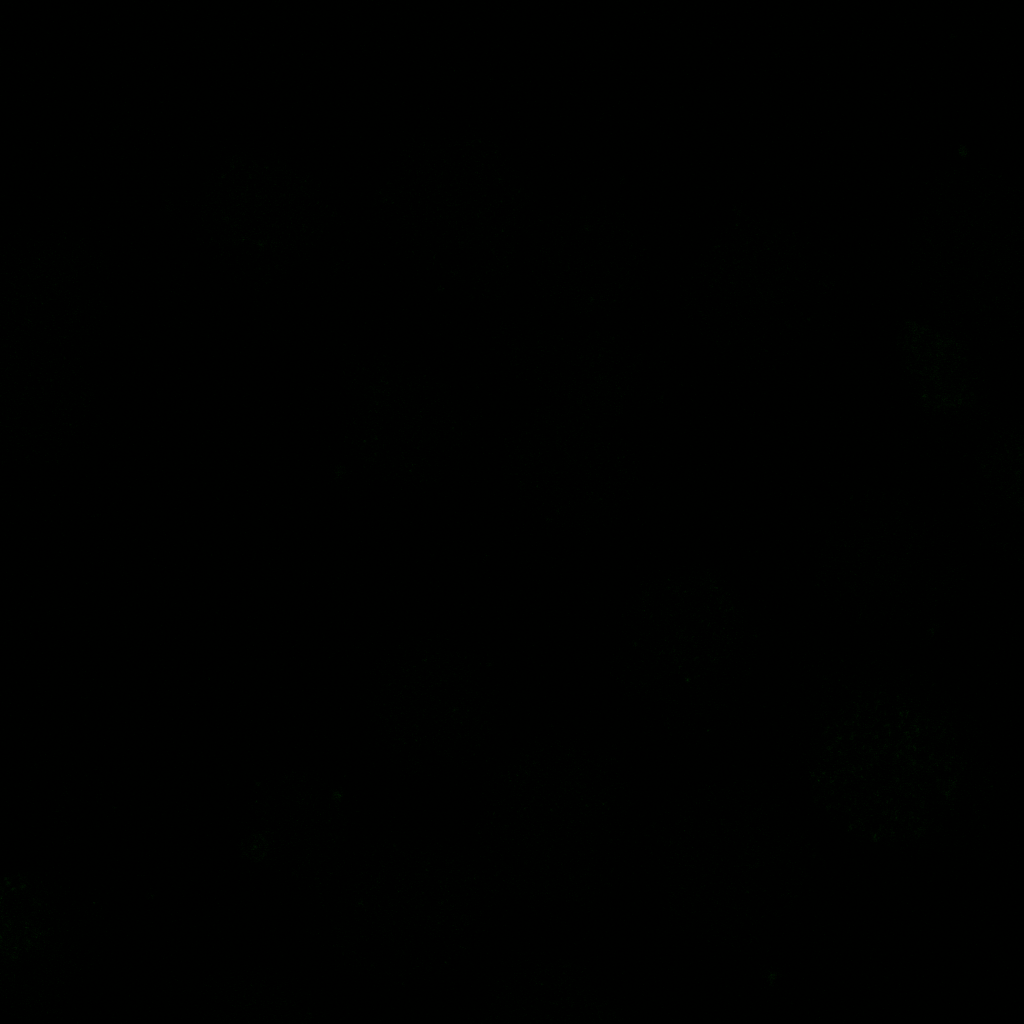

Supplement: Supplementary file 9 — Source Data for Figure 2 [file EMMM-15-e17313-s002.zip › Figure 2/C/Images/DMS114-ATR+Lurb-a-Image Export-02_c1.tif]

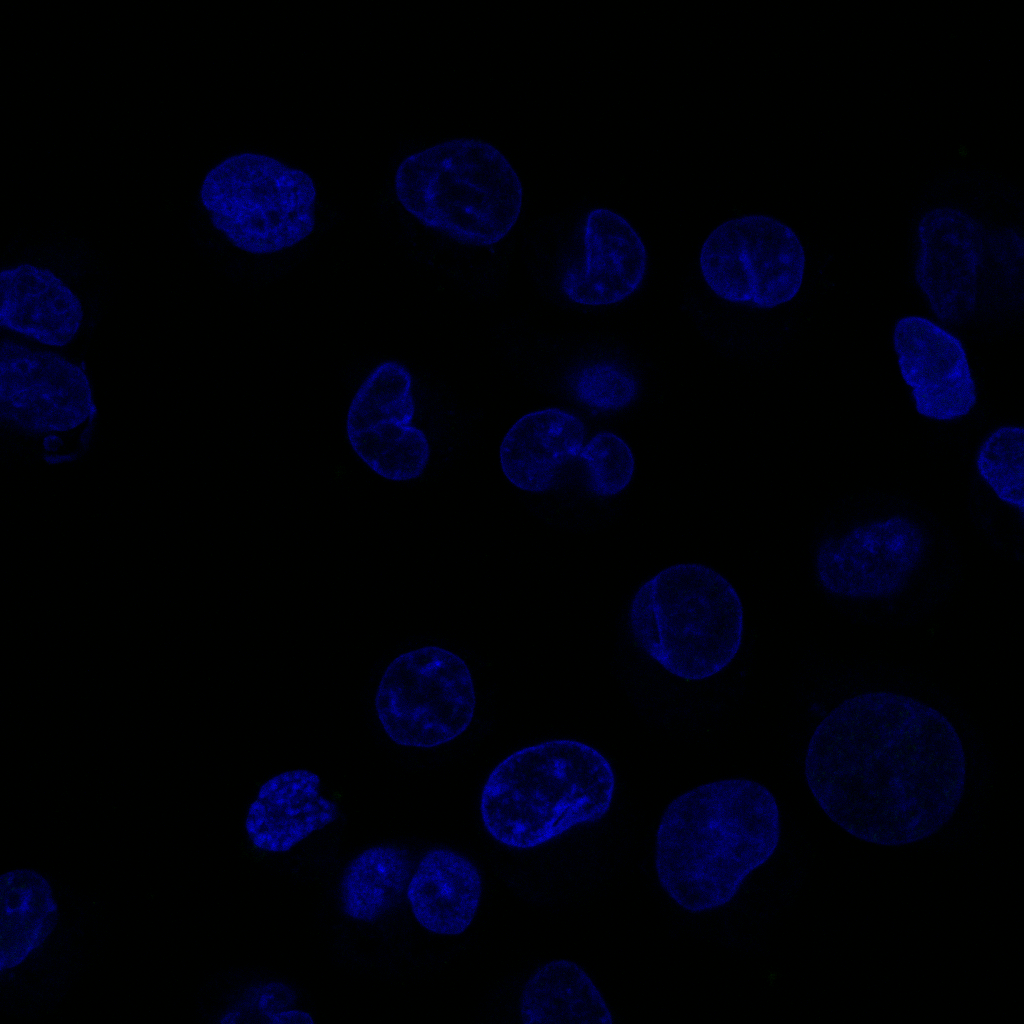

Supplement: Supplementary file 9 — Source Data for Figure 2 [file EMMM-15-e17313-s002.zip › Figure 2/C/Images/DMS114-ATR+Lurb-a-Image Export-02_c1+2.tif]

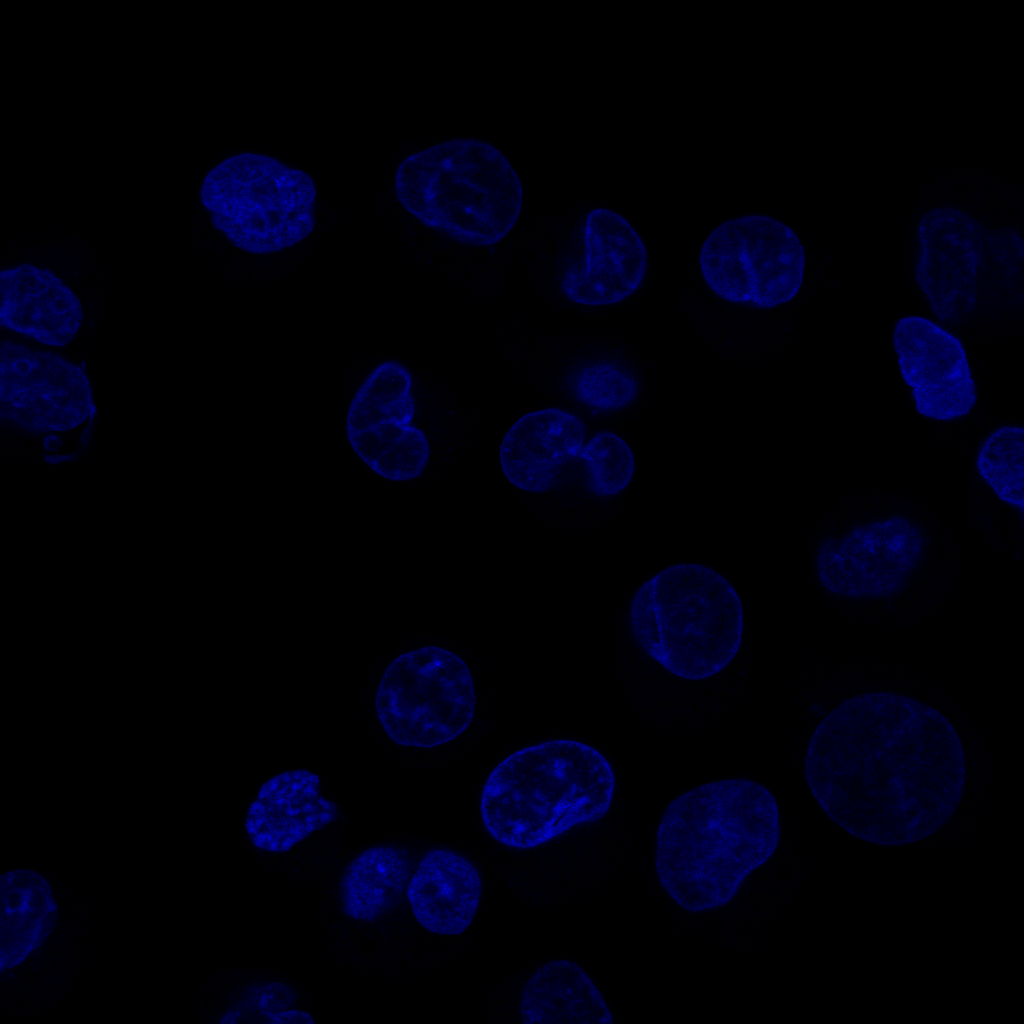

Supplement: Supplementary file 9 — Source Data for Figure 2 [file EMMM-15-e17313-s002.zip › Figure 2/C/Images/DMS114-ATR+Lurb-a-Image Export-02_c2.tif]

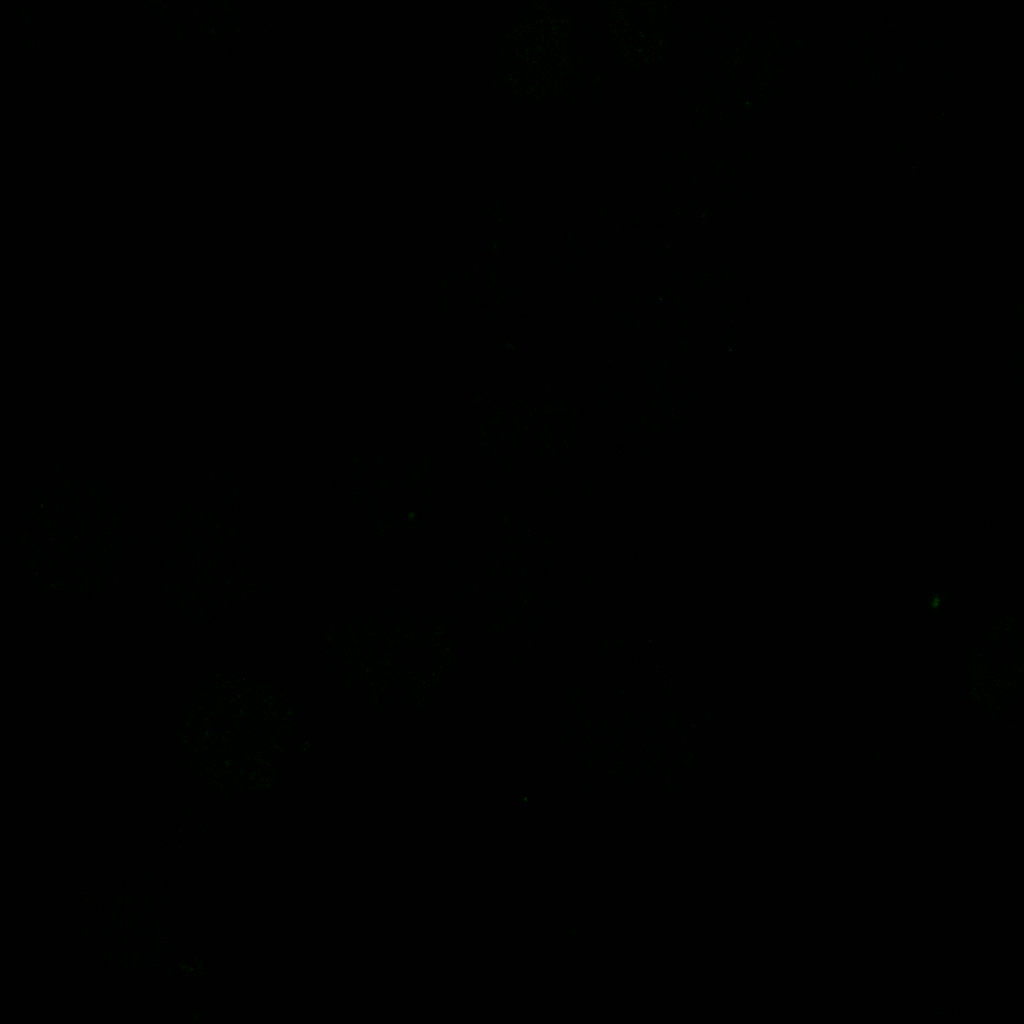

Supplement: Supplementary file 9 — Source Data for Figure 2 [file EMMM-15-e17313-s002.zip › Figure 2/C/Images/DMS114-ATR+Lurb-b-Image Export-03_c1.tif]

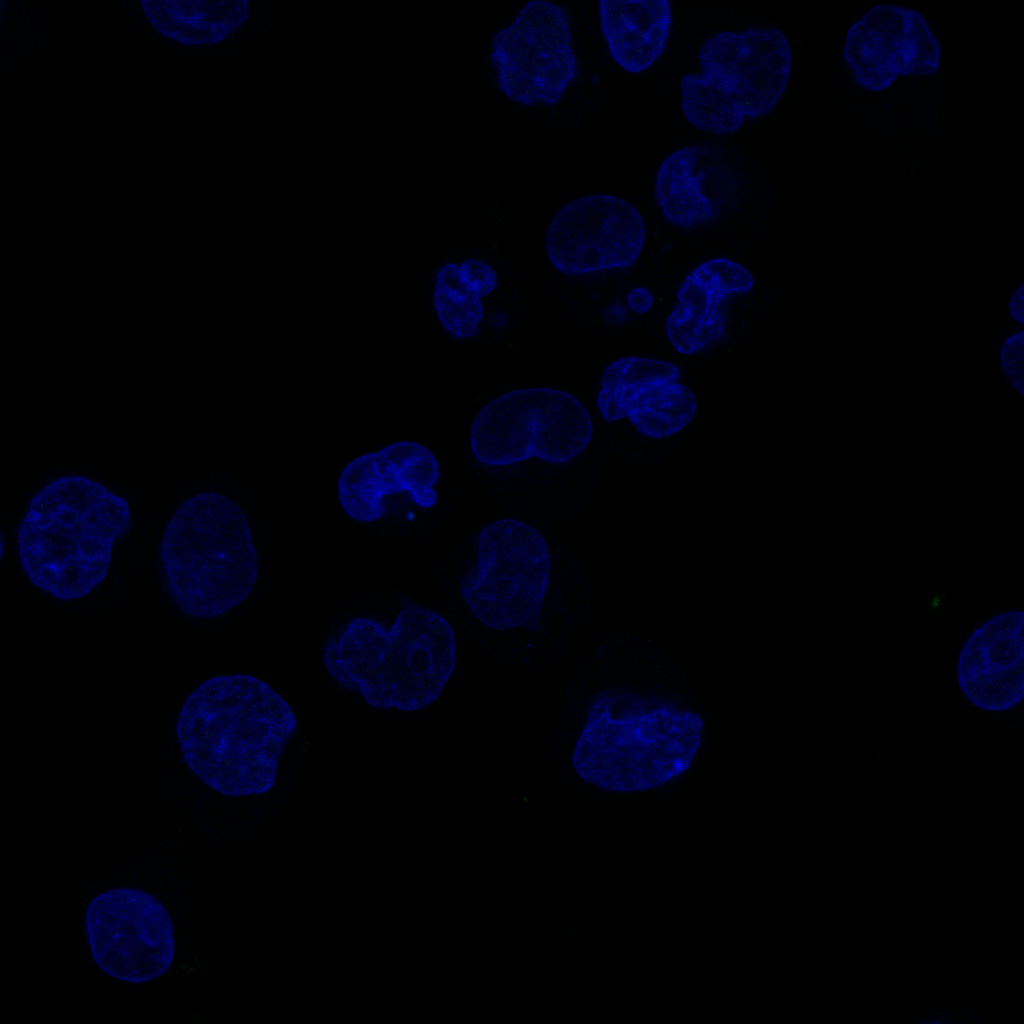

Supplement: Supplementary file 9 — Source Data for Figure 2 [file EMMM-15-e17313-s002.zip › Figure 2/C/Images/DMS114-ATR+Lurb-b-Image Export-03_c1+2.tif]

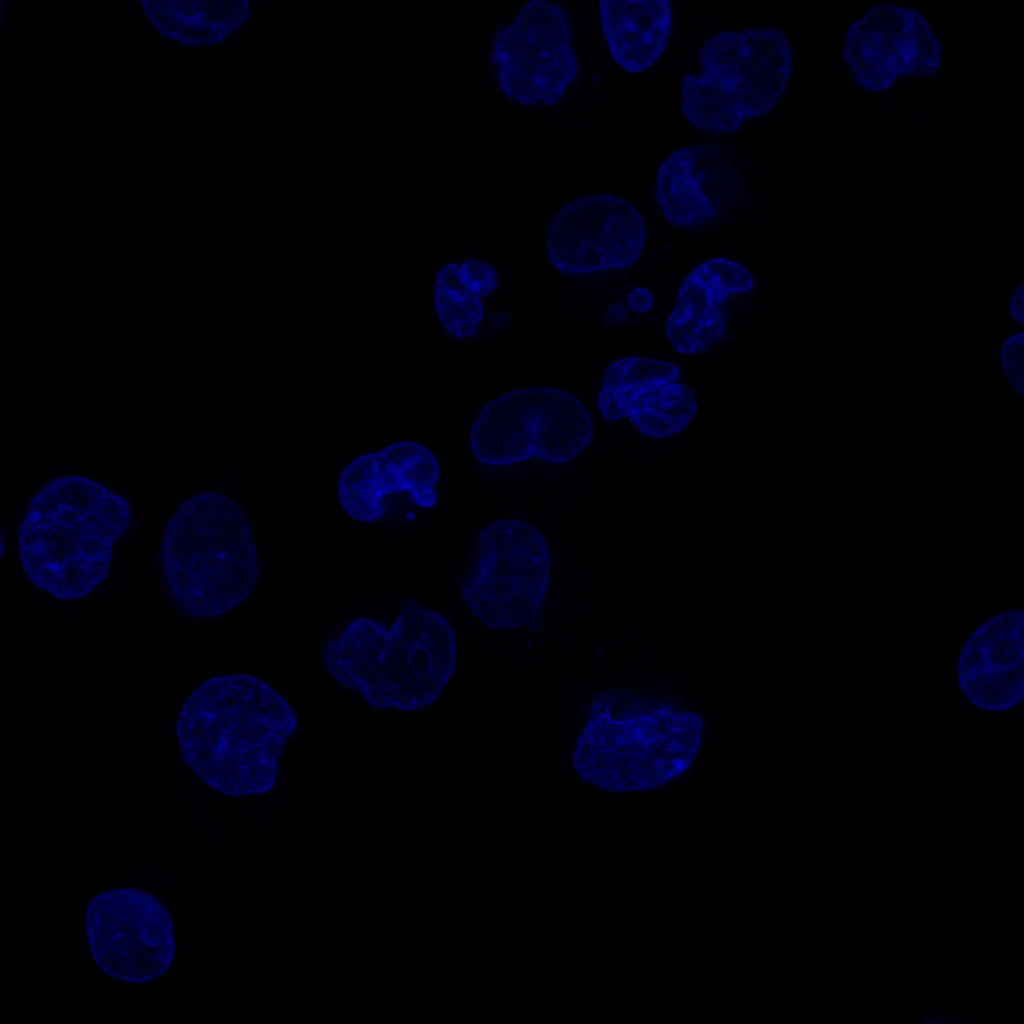

Supplement: Supplementary file 9 — Source Data for Figure 2 [file EMMM-15-e17313-s002.zip › Figure 2/C/Images/DMS114-ATR+Lurb-b-Image Export-03_c2.tif]

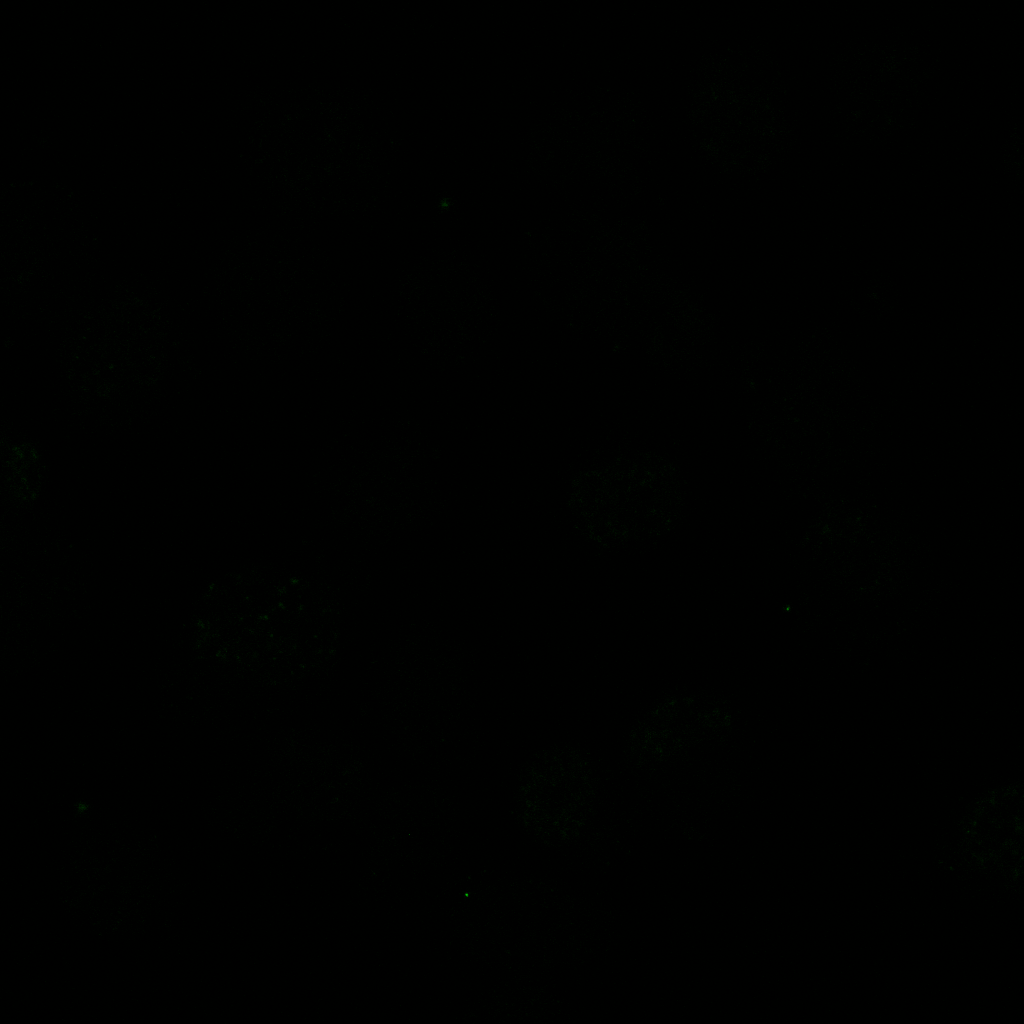

Supplement: Supplementary file 9 — Source Data for Figure 2 [file EMMM-15-e17313-s002.zip › Figure 2/C/Images/DMS114-ATR+Lurb-c-Image Export-04_c1.tif]

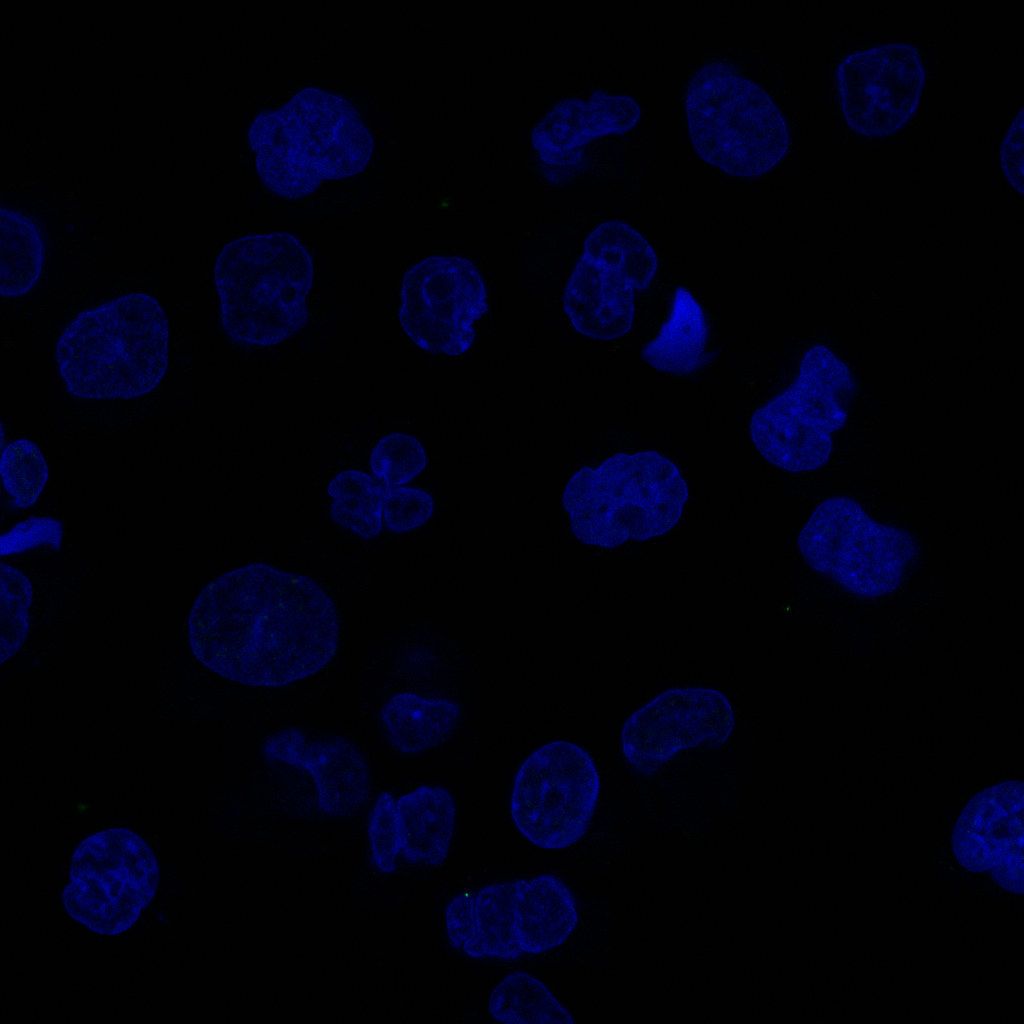

Supplement: Supplementary file 9 — Source Data for Figure 2 [file EMMM-15-e17313-s002.zip › Figure 2/C/Images/DMS114-ATR+Lurb-c-Image Export-04_c1+2.tif]

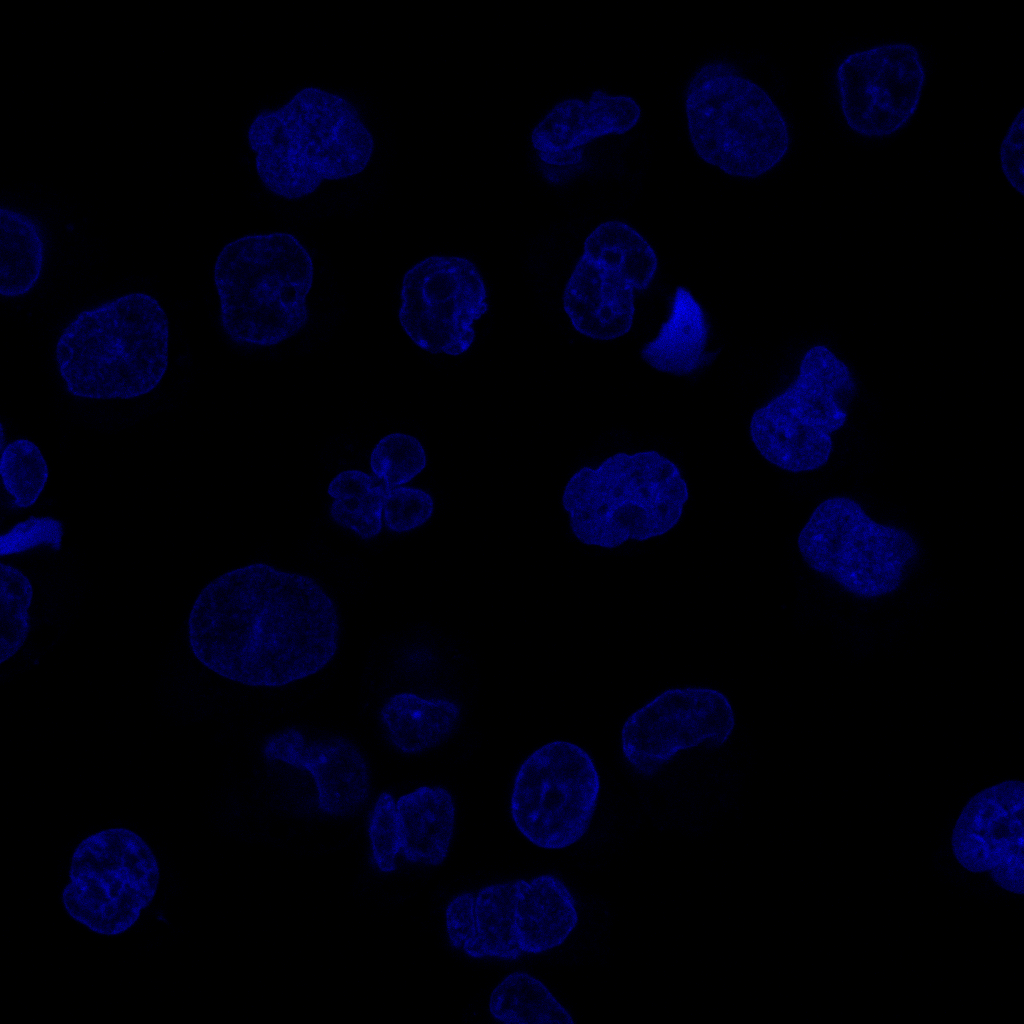

Supplement: Supplementary file 9 — Source Data for Figure 2 [file EMMM-15-e17313-s002.zip › Figure 2/C/Images/DMS114-ATR+Lurb-c-Image Export-04_c2.tif]

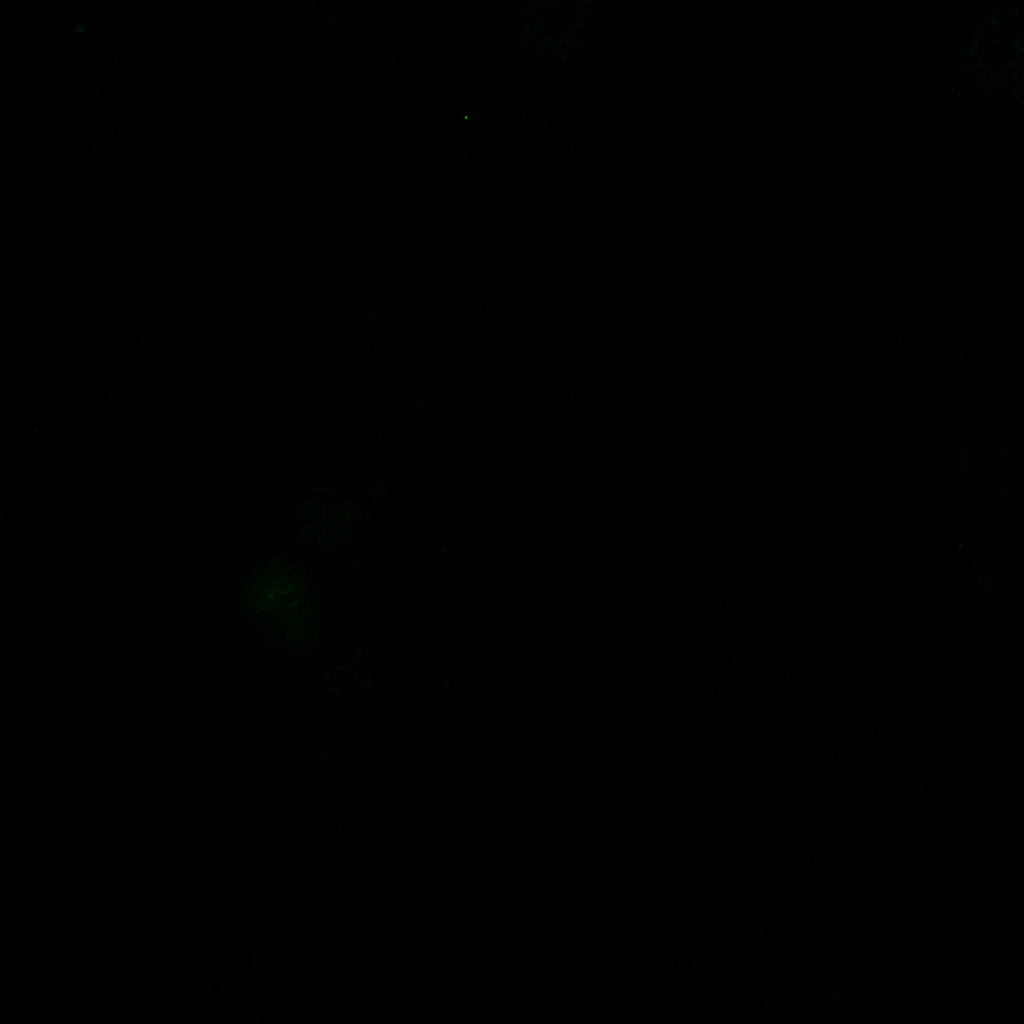

Supplement: Supplementary file 9 — Source Data for Figure 2 [file EMMM-15-e17313-s002.zip › Figure 2/C/Images/DMS114-ATR+Lurb-d-Image Export-05_c1.tif]

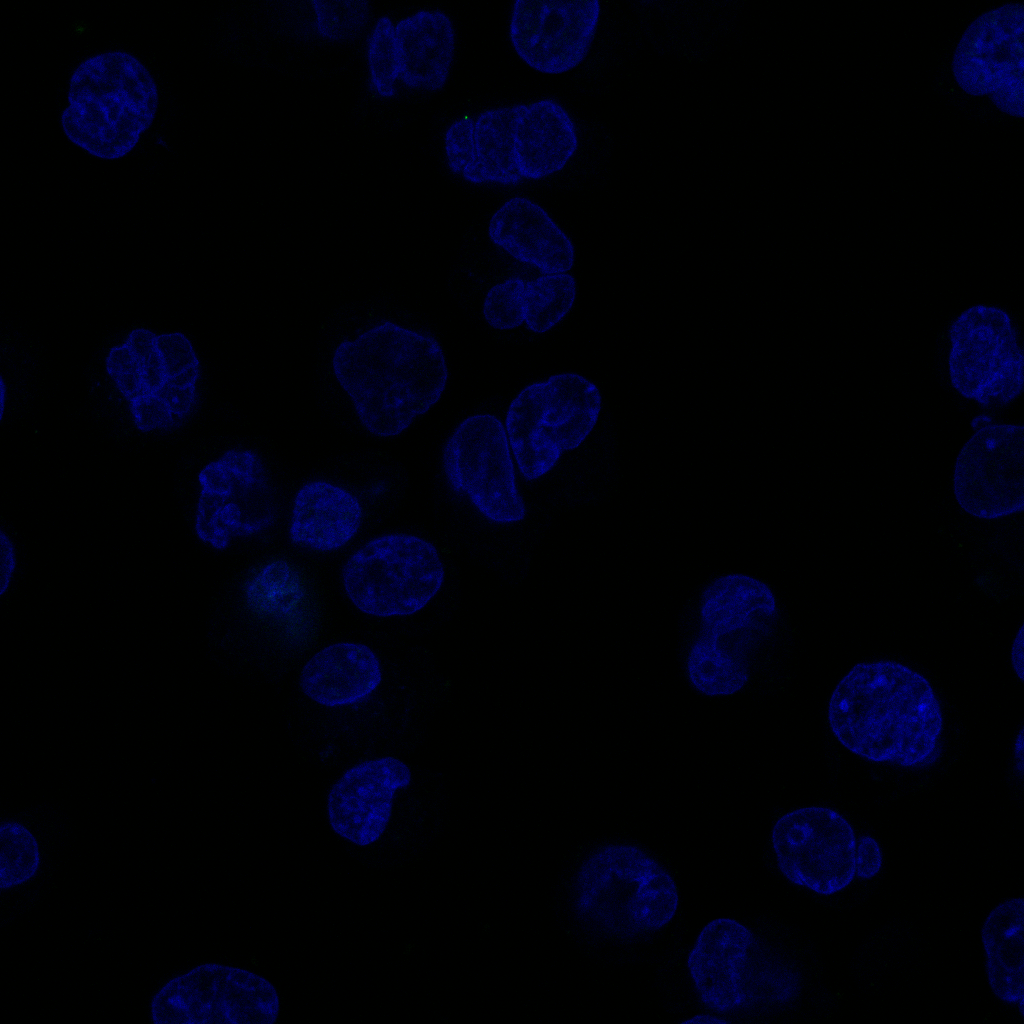

Supplement: Supplementary file 9 — Source Data for Figure 2 [file EMMM-15-e17313-s002.zip › Figure 2/C/Images/DMS114-ATR+Lurb-d-Image Export-05_c1+2.tif]

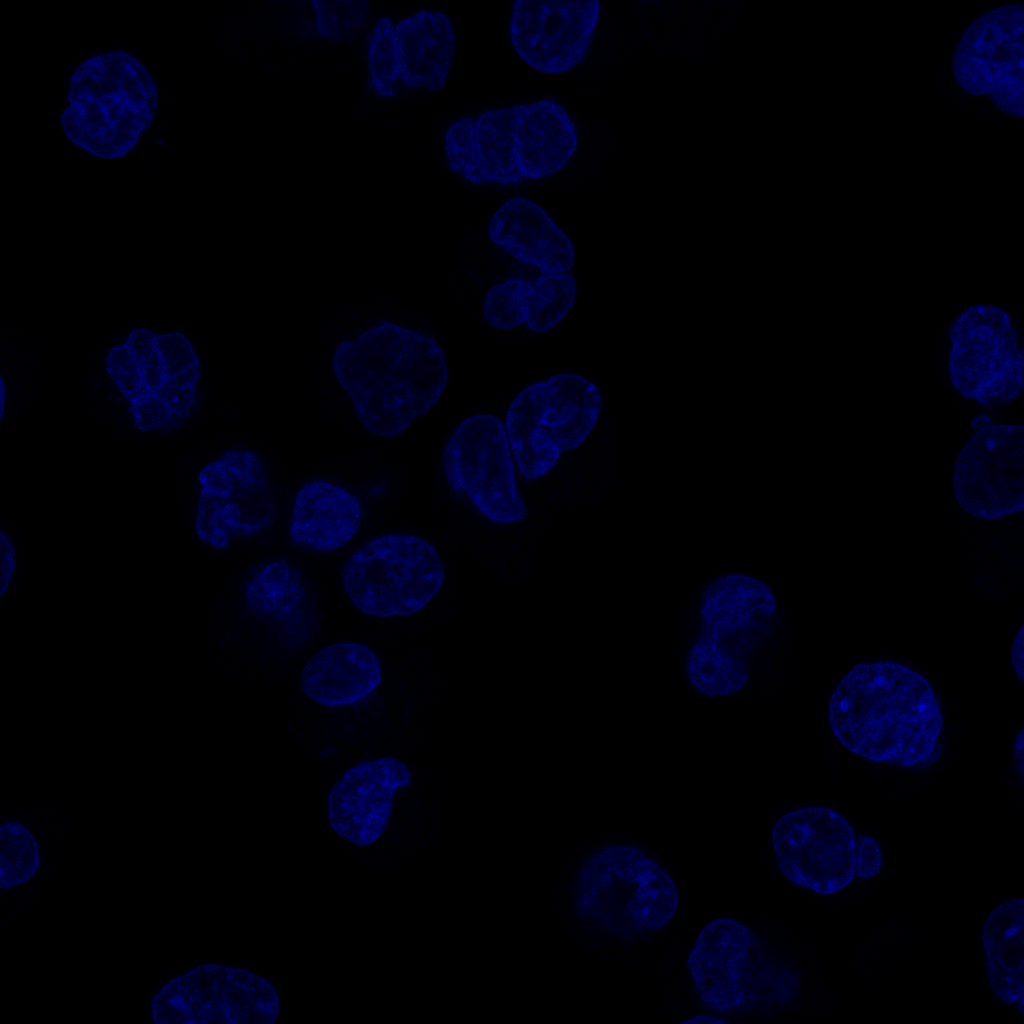

Supplement: Supplementary file 9 — Source Data for Figure 2 [file EMMM-15-e17313-s002.zip › Figure 2/C/Images/DMS114-ATR+Lurb-d-Image Export-05_c2.tif]

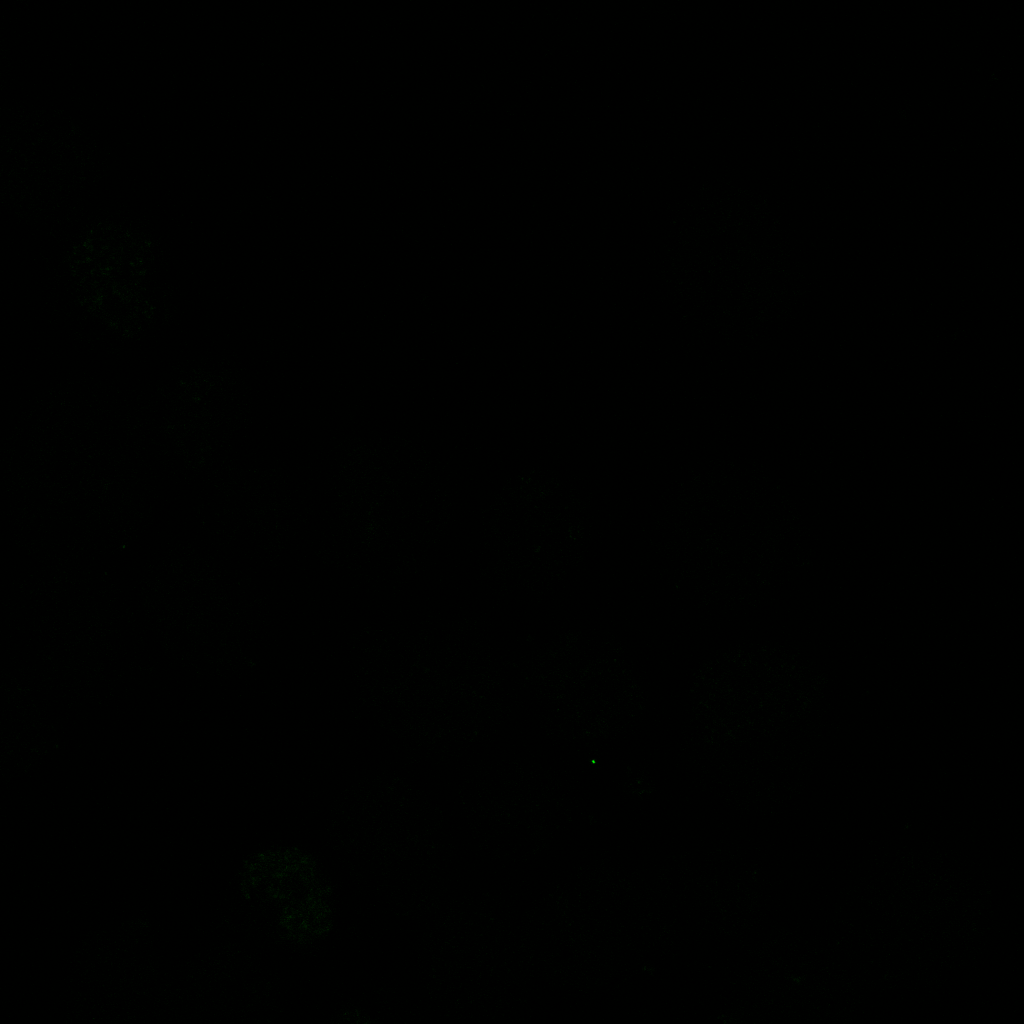

Supplement: Supplementary file 9 — Source Data for Figure 2 [file EMMM-15-e17313-s002.zip › Figure 2/C/Images/DMS114-ATR+Lurb-e-Image Export-06_c1.tif]

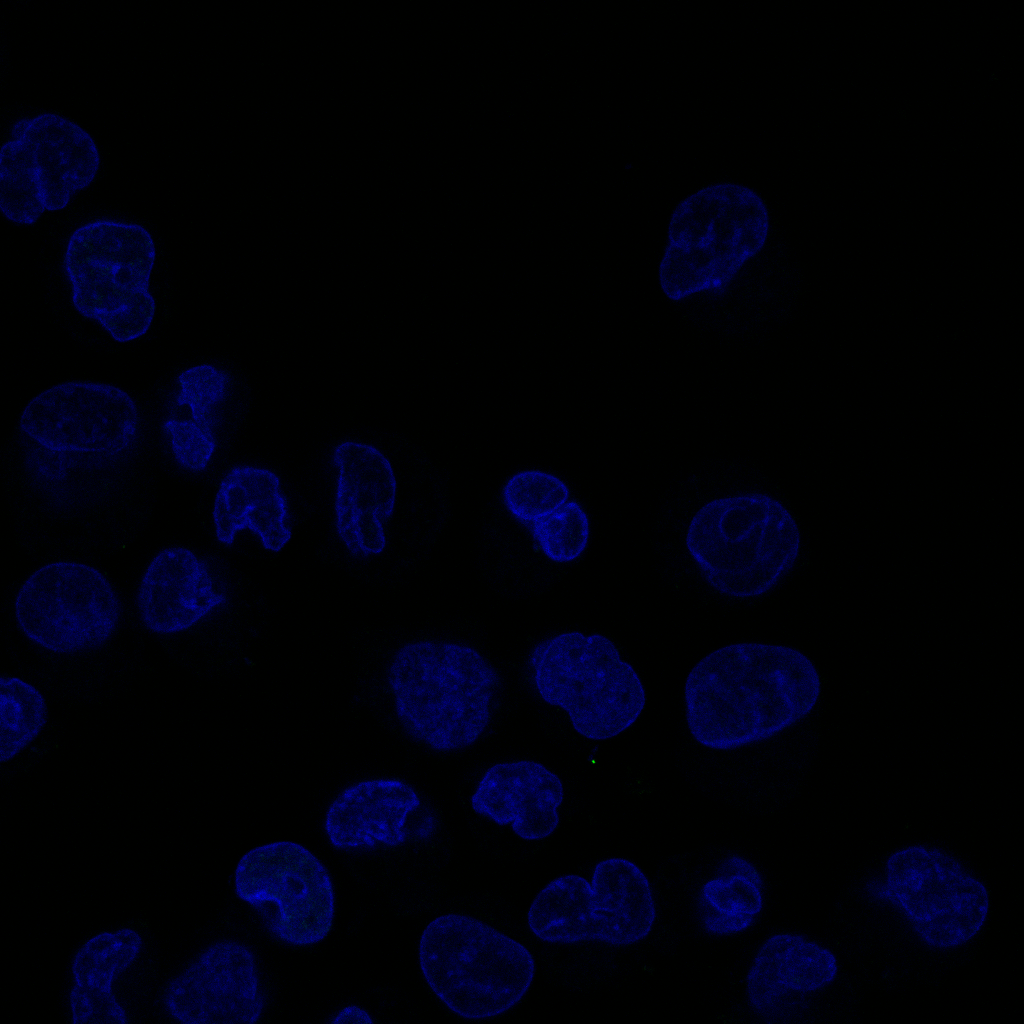

Supplement: Supplementary file 9 — Source Data for Figure 2 [file EMMM-15-e17313-s002.zip › Figure 2/C/Images/DMS114-ATR+Lurb-e-Image Export-06_c1+2.tif]

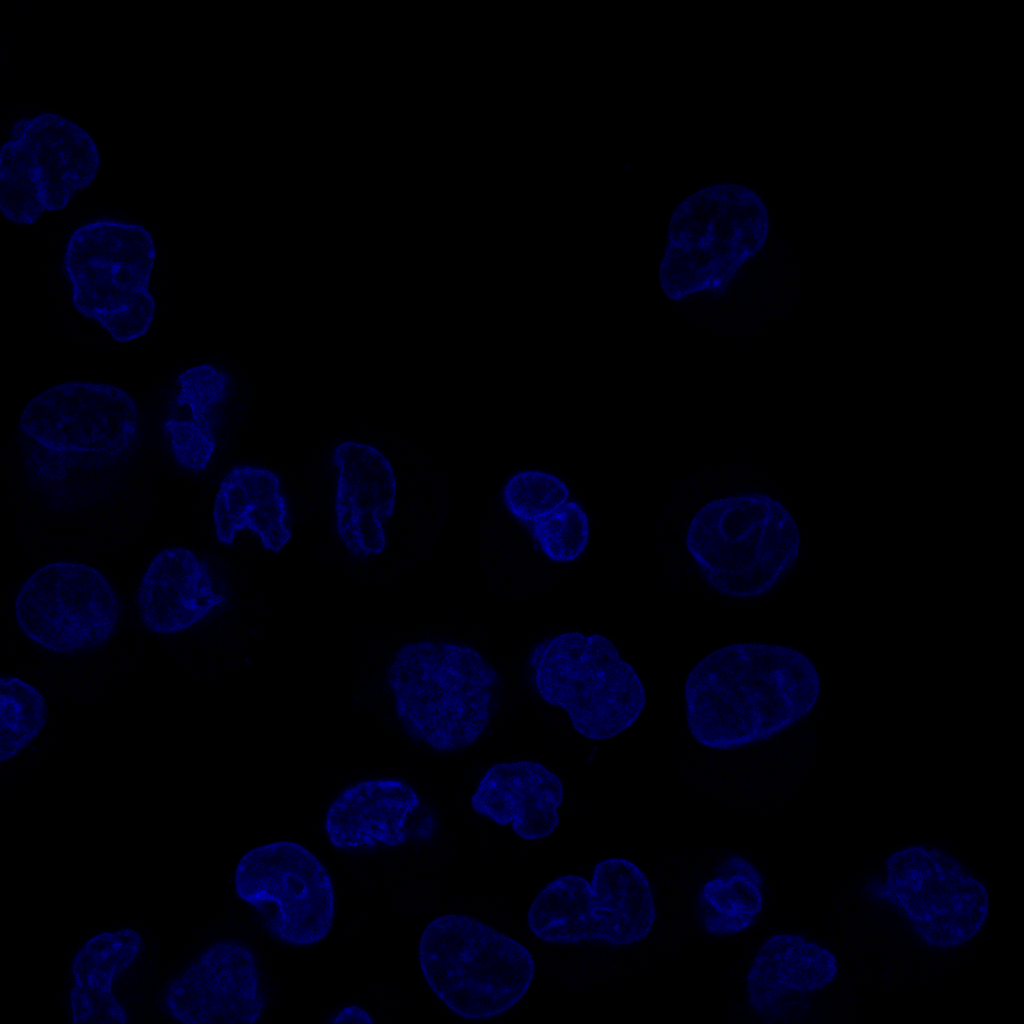

Supplement: Supplementary file 9 — Source Data for Figure 2 [file EMMM-15-e17313-s002.zip › Figure 2/C/Images/DMS114-ATR+Lurb-e-Image Export-06_c2.tif]

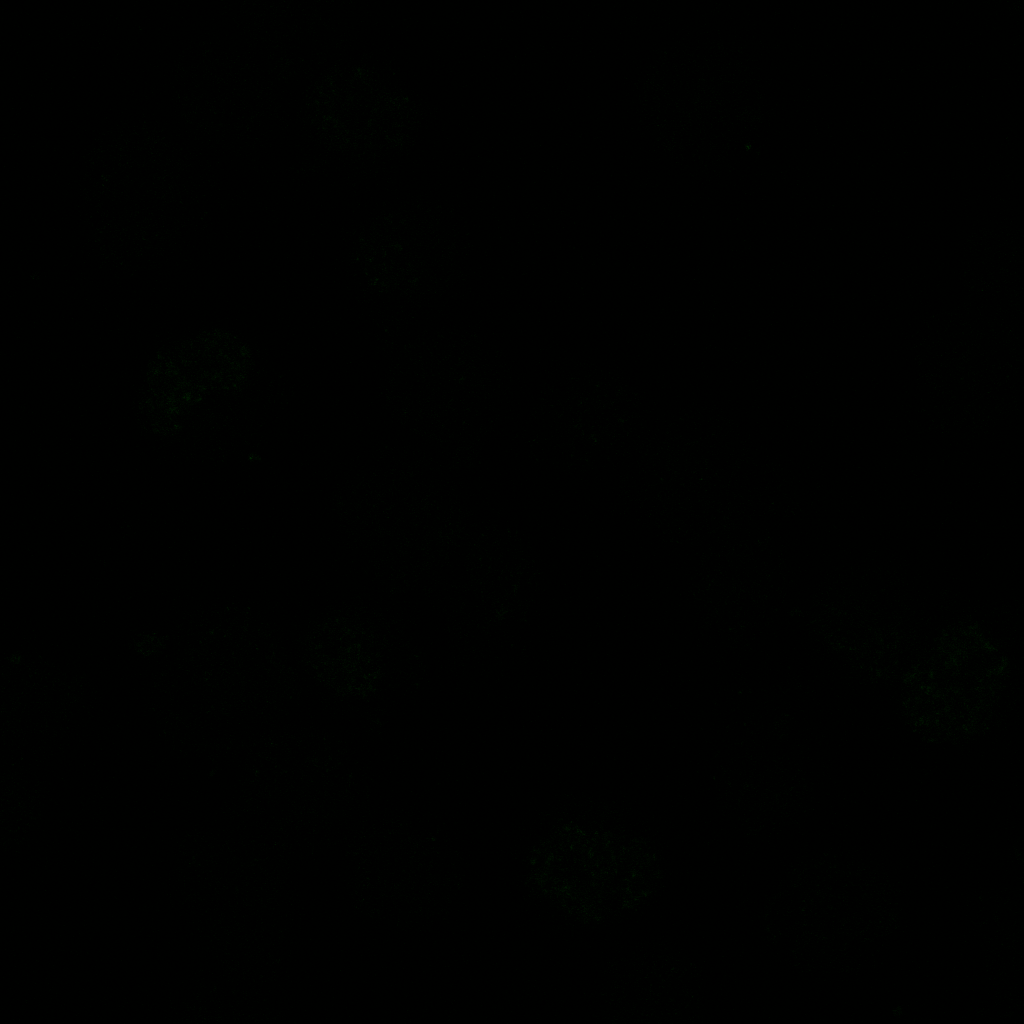

Supplement: Supplementary file 9 — Source Data for Figure 2 [file EMMM-15-e17313-s002.zip › Figure 2/C/Images/DMS114-ATR+Lurb-f-Image Export-07_c1.tif]

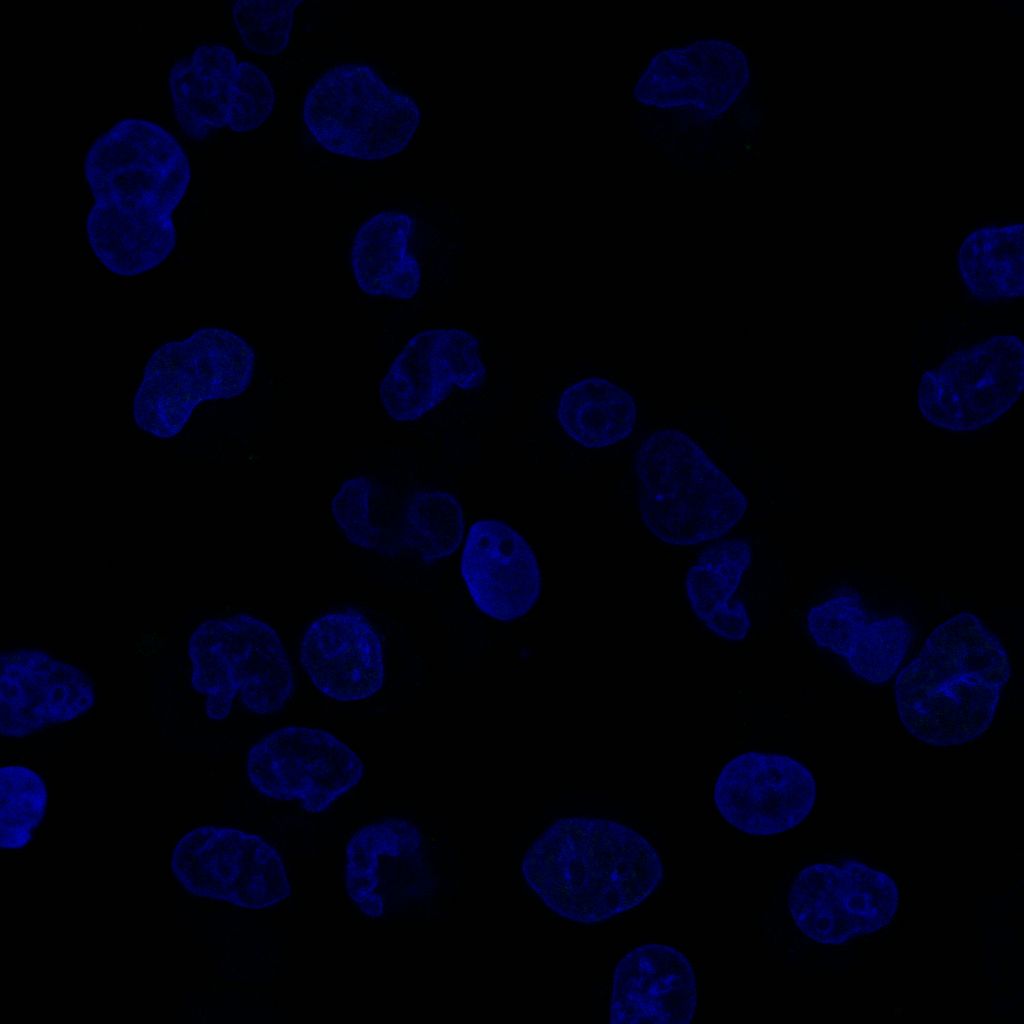

Supplement: Supplementary file 9 — Source Data for Figure 2 [file EMMM-15-e17313-s002.zip › Figure 2/C/Images/DMS114-ATR+Lurb-f-Image Export-07_c1+2.tif]

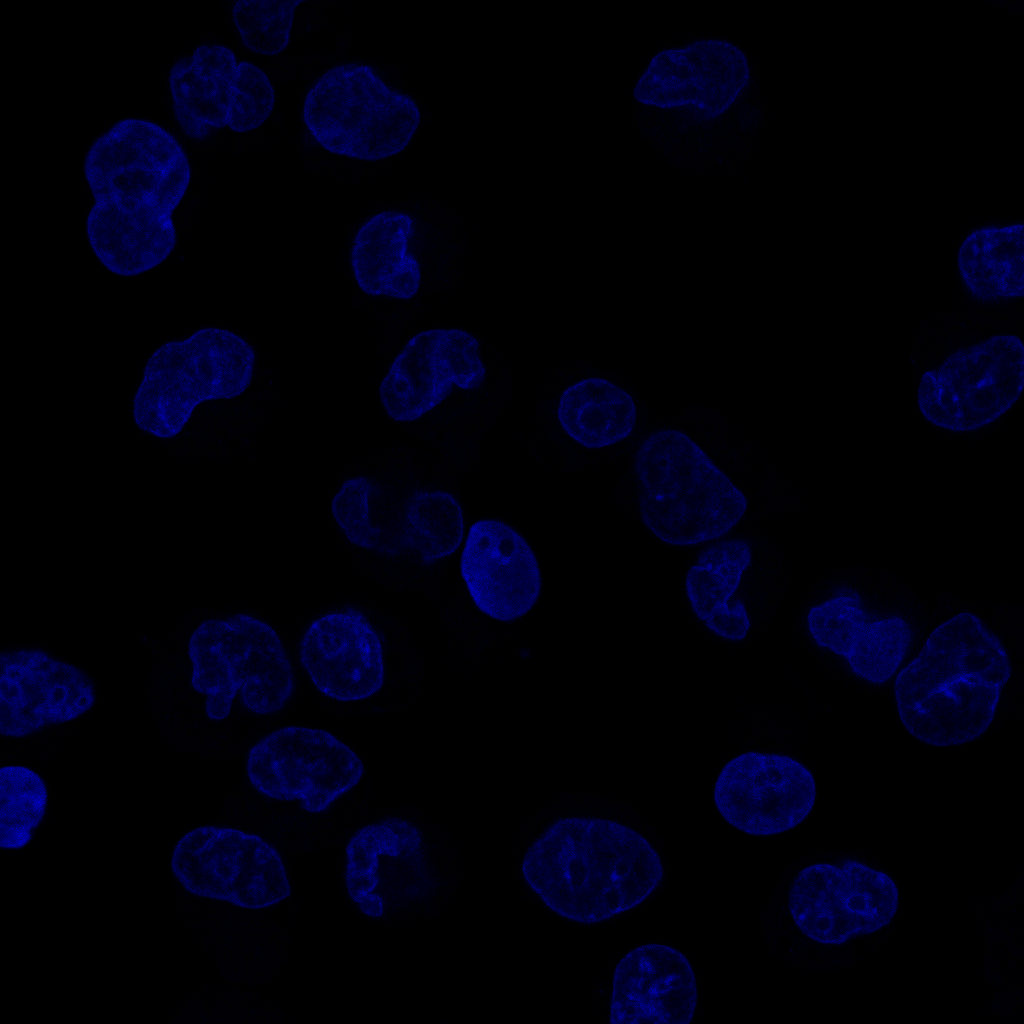

Supplement: Supplementary file 9 — Source Data for Figure 2 [file EMMM-15-e17313-s002.zip › Figure 2/C/Images/DMS114-ATR+Lurb-f-Image Export-07_c2.tif]

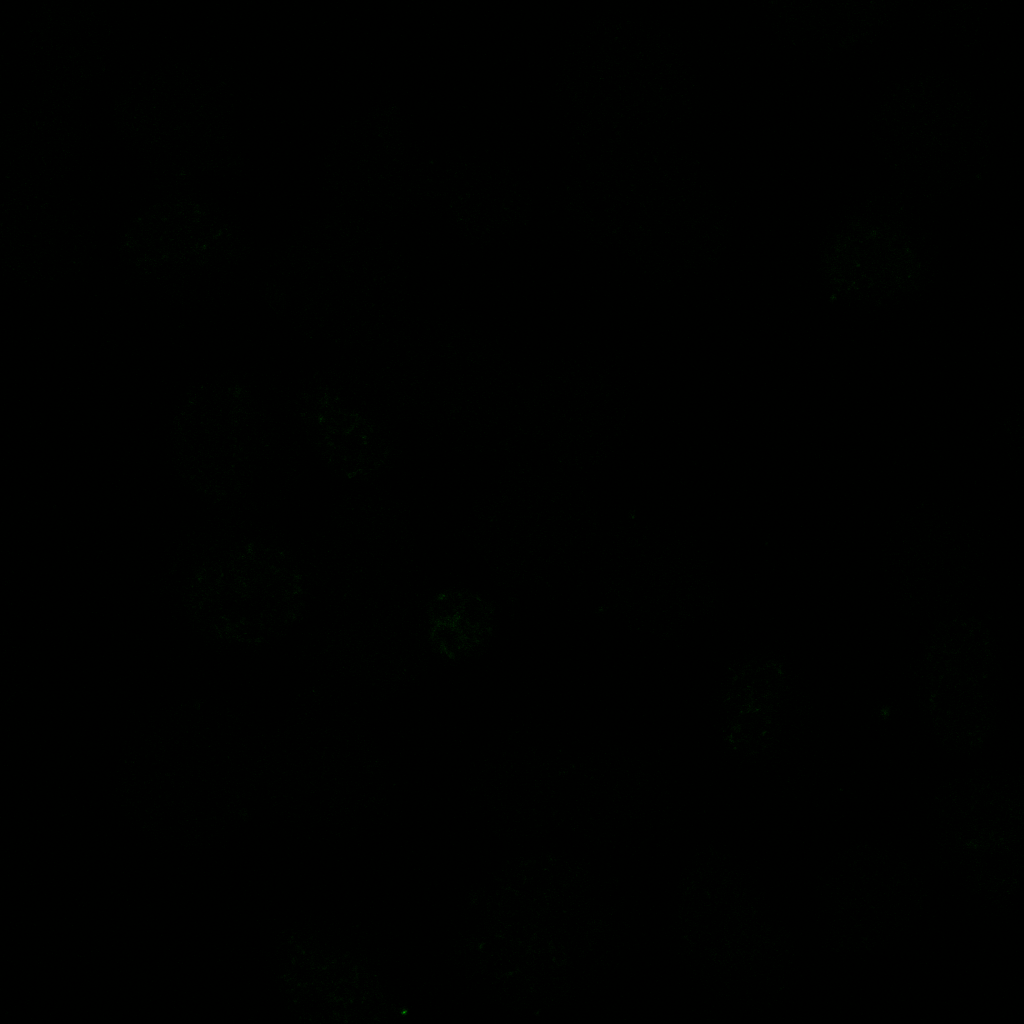

Supplement: Supplementary file 9 — Source Data for Figure 2 [file EMMM-15-e17313-s002.zip › Figure 2/C/Images/DMS114-ATR+Lurb-g-Image Export-08_c1.tif]

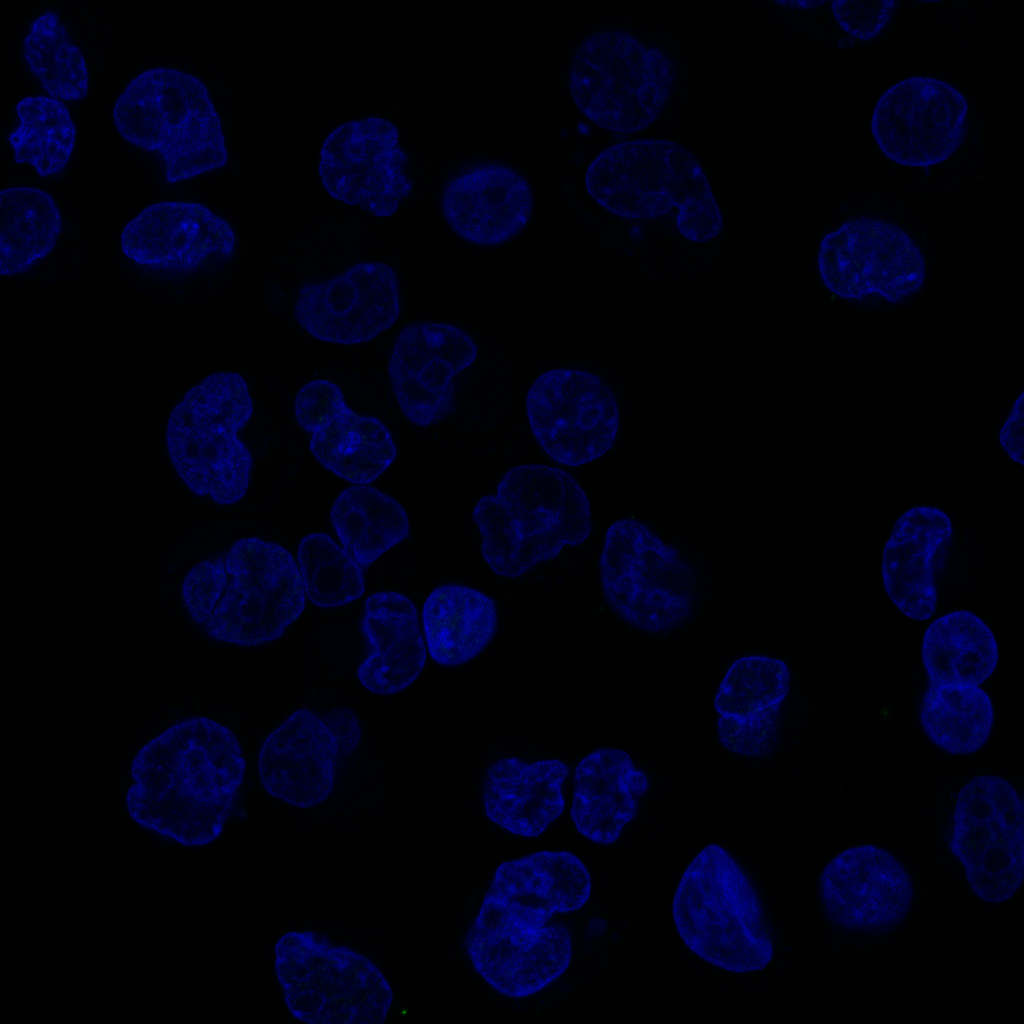

Supplement: Supplementary file 9 — Source Data for Figure 2 [file EMMM-15-e17313-s002.zip › Figure 2/C/Images/DMS114-ATR+Lurb-g-Image Export-08_c1+2.tif]

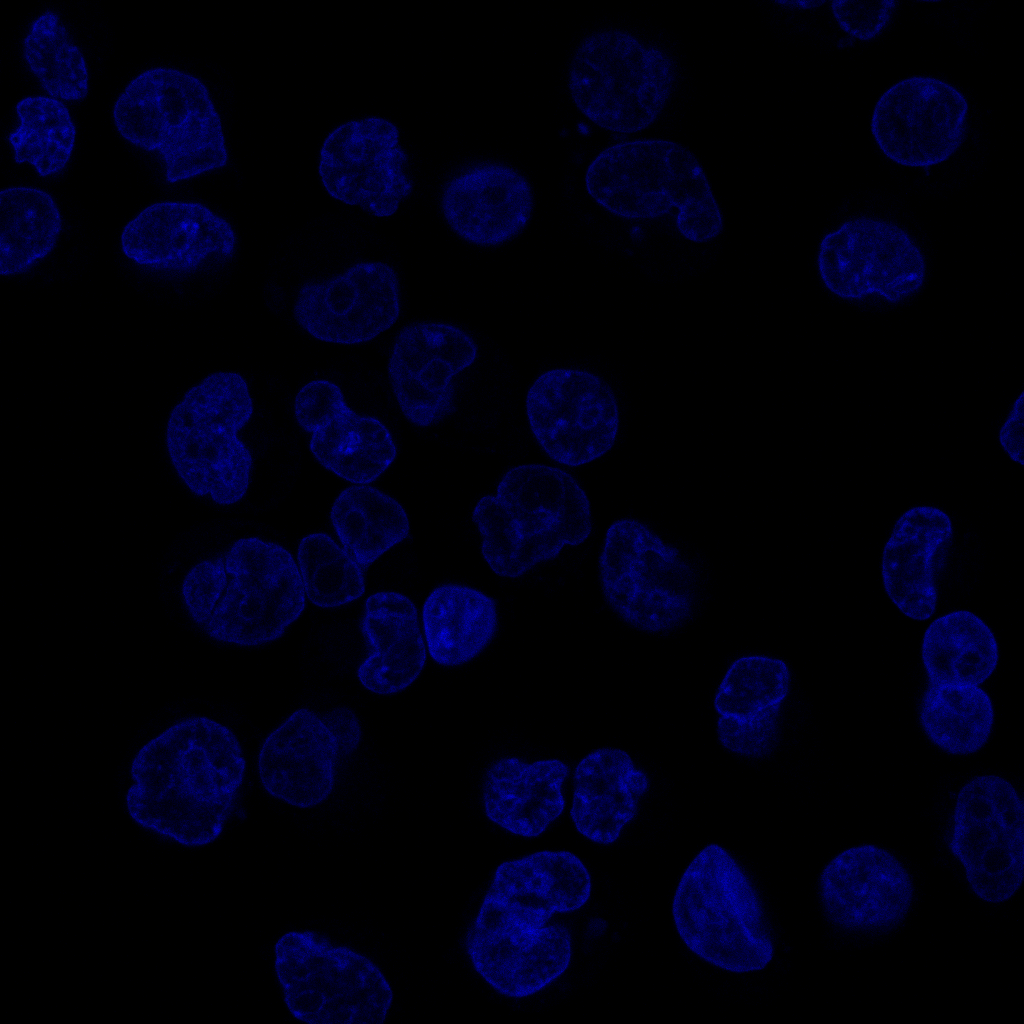

Supplement: Supplementary file 9 — Source Data for Figure 2 [file EMMM-15-e17313-s002.zip › Figure 2/C/Images/DMS114-ATR+Lurb-g-Image Export-08_c2.tif]

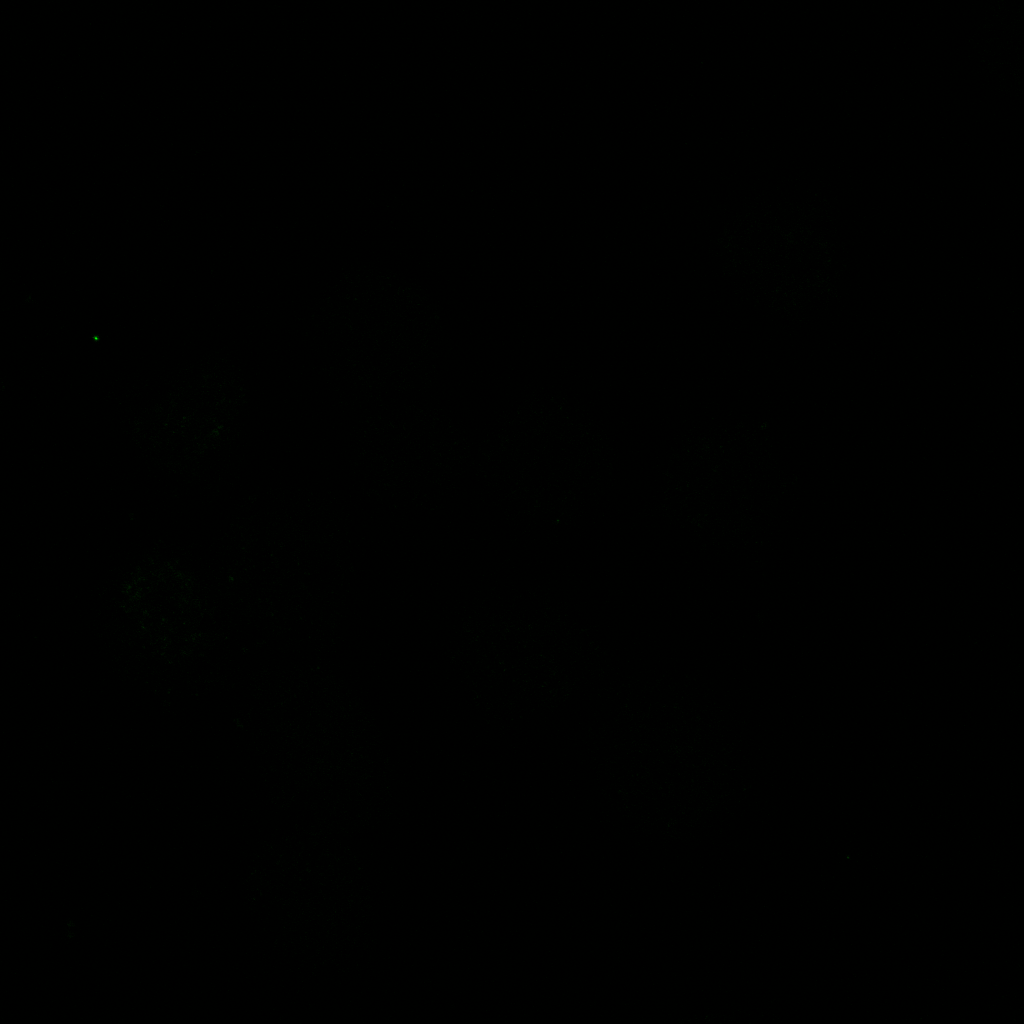

Supplement: Supplementary file 9 — Source Data for Figure 2 [file EMMM-15-e17313-s002.zip › Figure 2/C/Images/DMS114-ATR-a-Image Export-09_c1.tif]

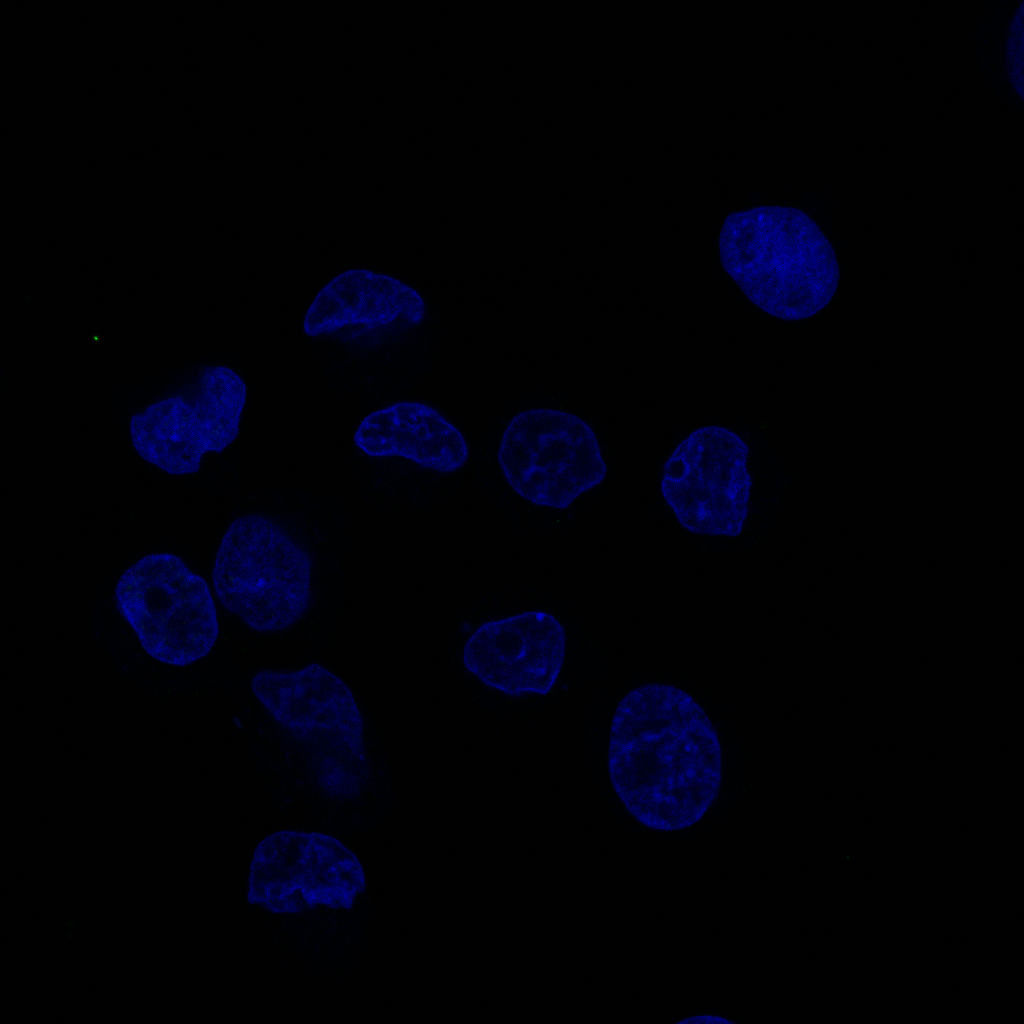

Supplement: Supplementary file 9 — Source Data for Figure 2 [file EMMM-15-e17313-s002.zip › Figure 2/C/Images/DMS114-ATR-a-Image Export-09_c1+2.tif]

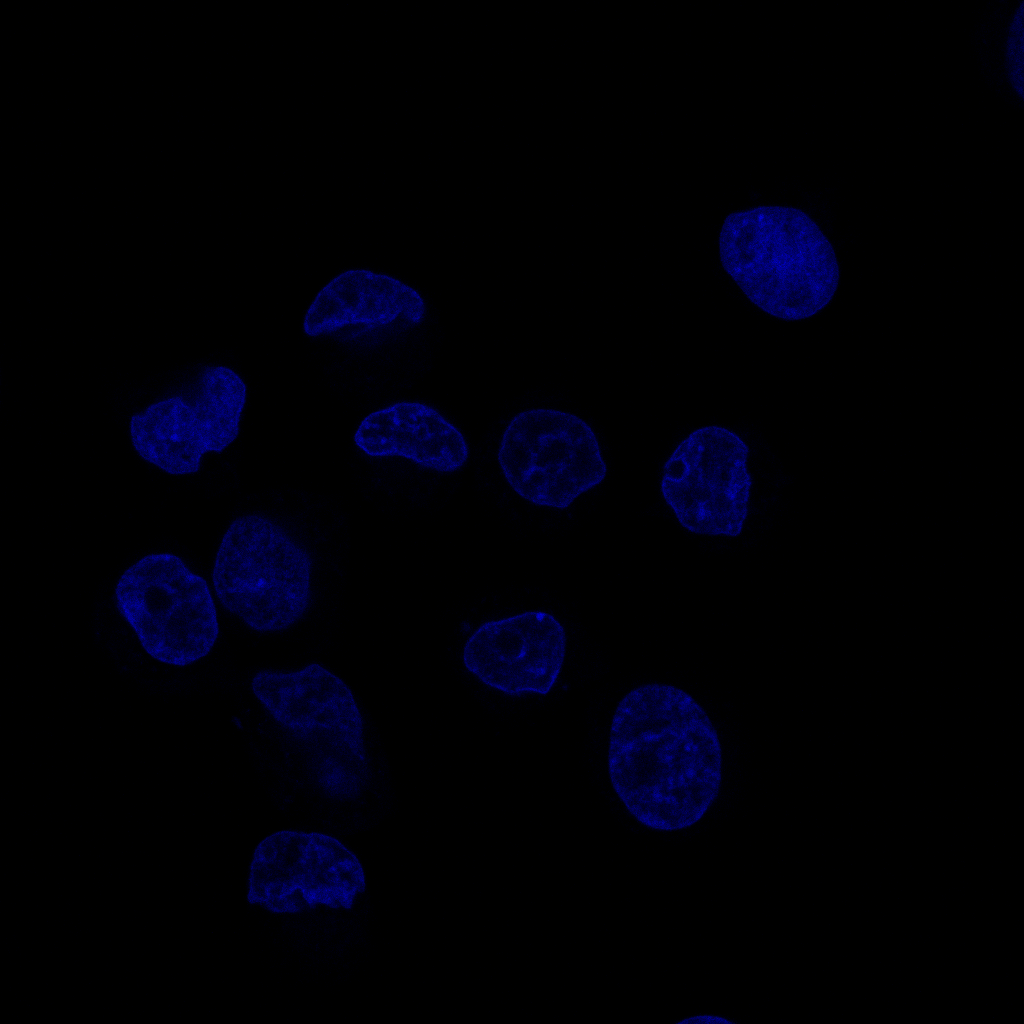

Supplement: Supplementary file 9 — Source Data for Figure 2 [file EMMM-15-e17313-s002.zip › Figure 2/C/Images/DMS114-ATR-a-Image Export-09_c2.tif]

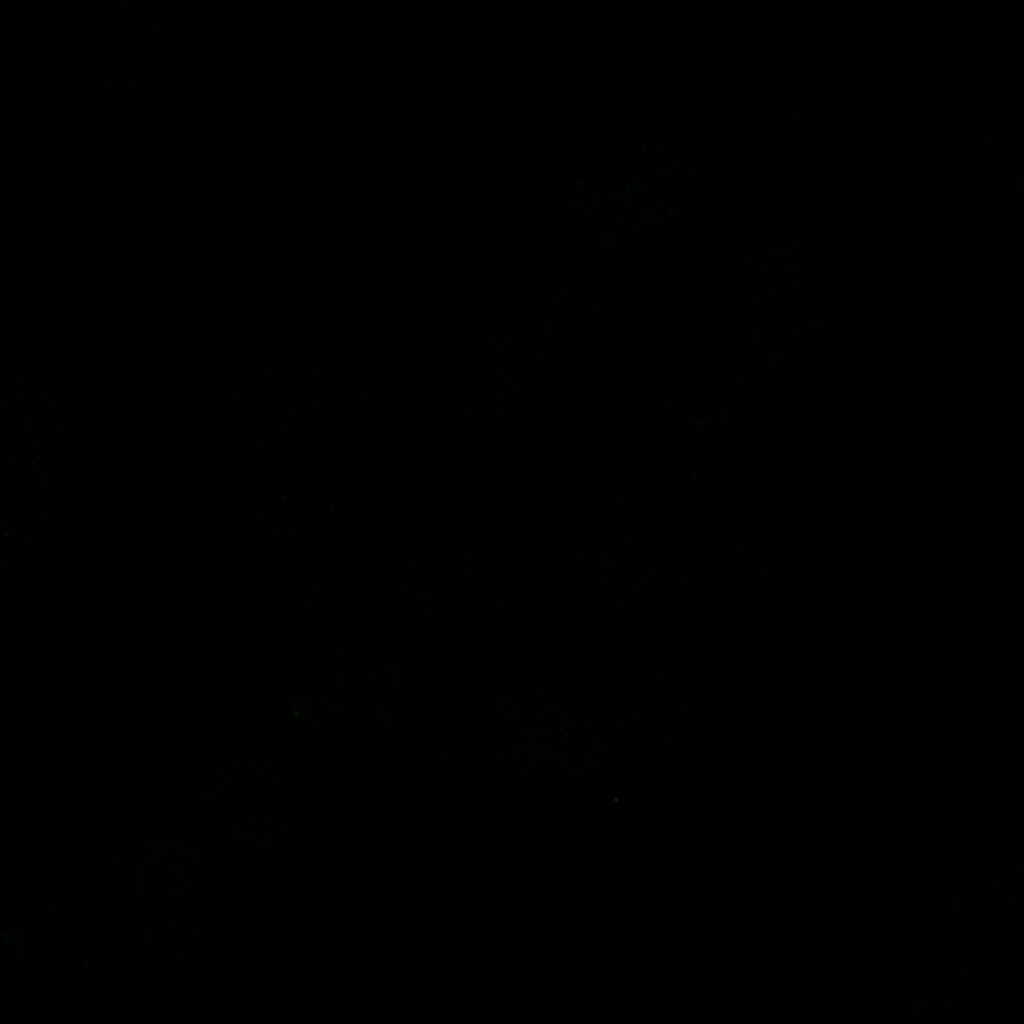

Supplement: Supplementary file 9 — Source Data for Figure 2 [file EMMM-15-e17313-s002.zip › Figure 2/C/Images/DMS114-ATR-b-Image Export-10_c1.tif]

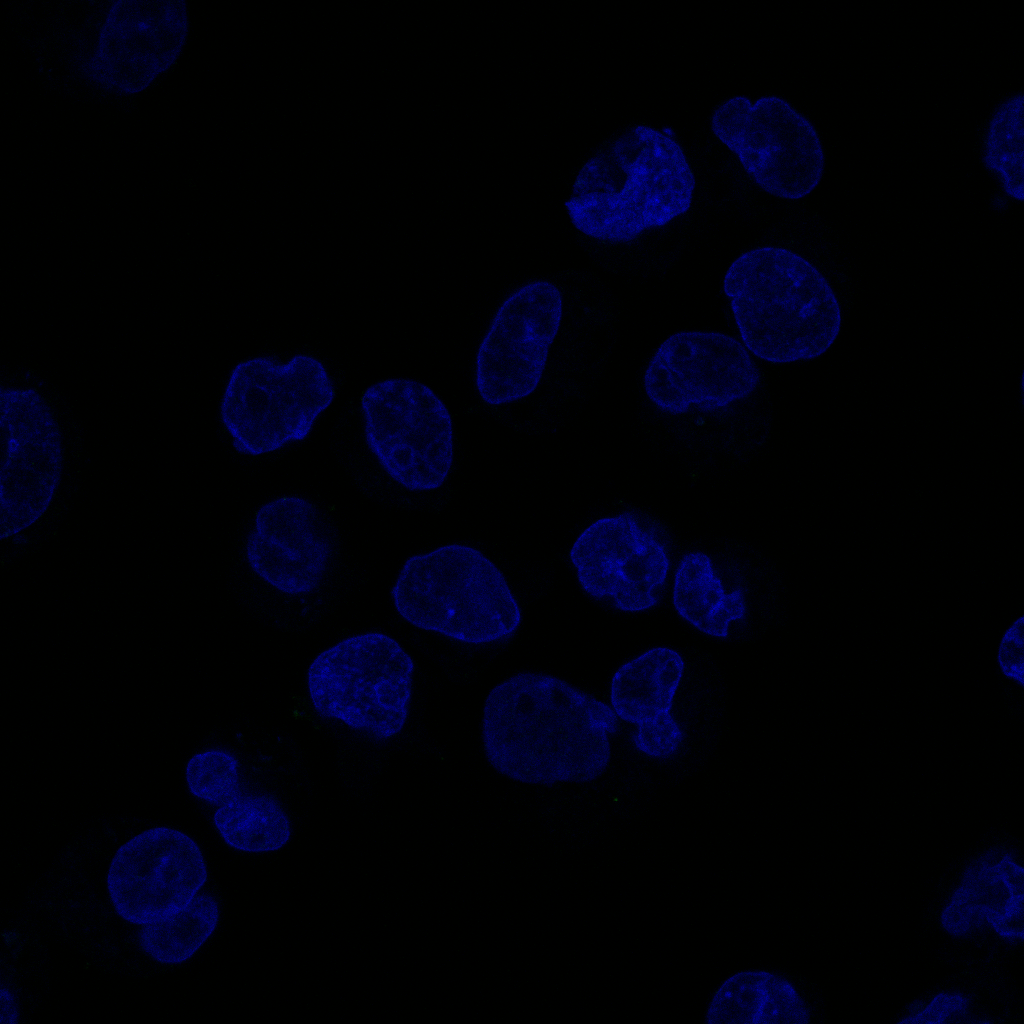

Supplement: Supplementary file 9 — Source Data for Figure 2 [file EMMM-15-e17313-s002.zip › Figure 2/C/Images/DMS114-ATR-b-Image Export-10_c1+2.tif]

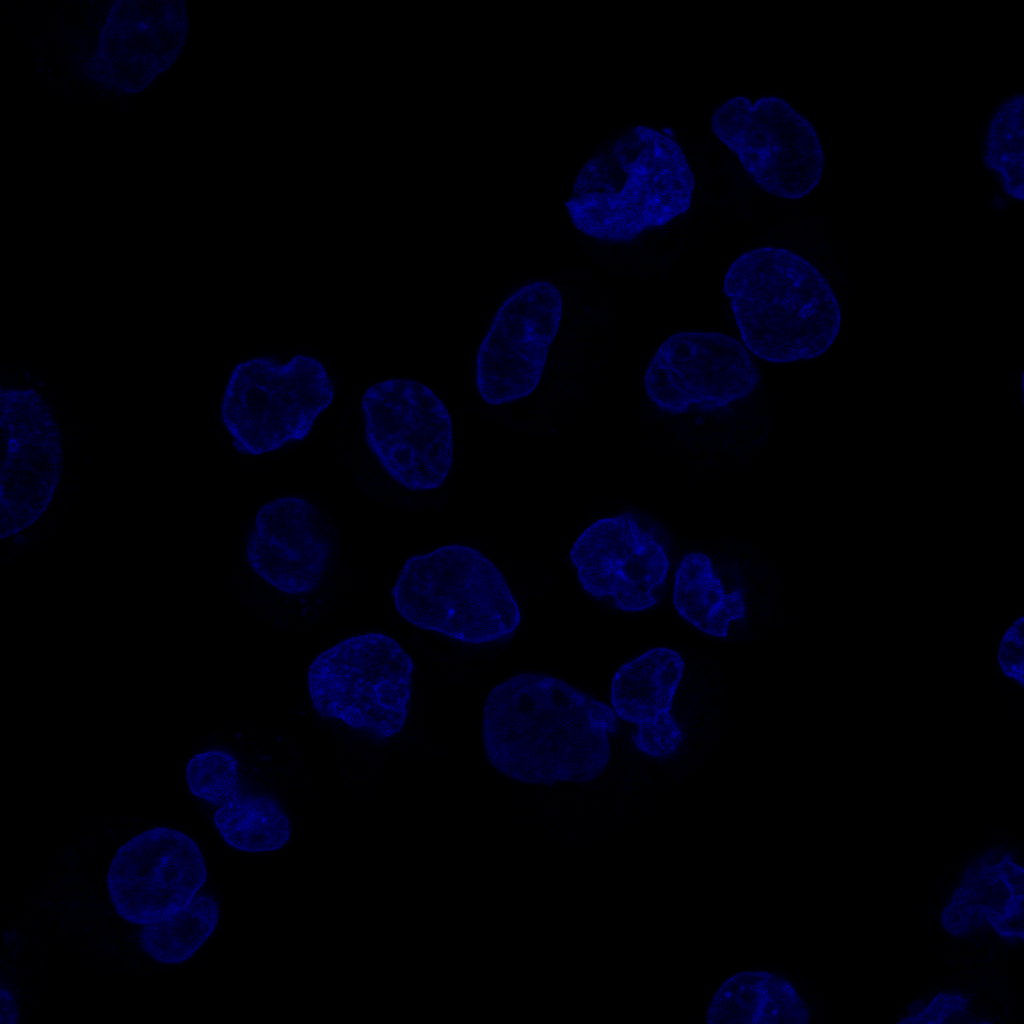

Supplement: Supplementary file 9 — Source Data for Figure 2 [file EMMM-15-e17313-s002.zip › Figure 2/C/Images/DMS114-ATR-b-Image Export-10_c2.tif]

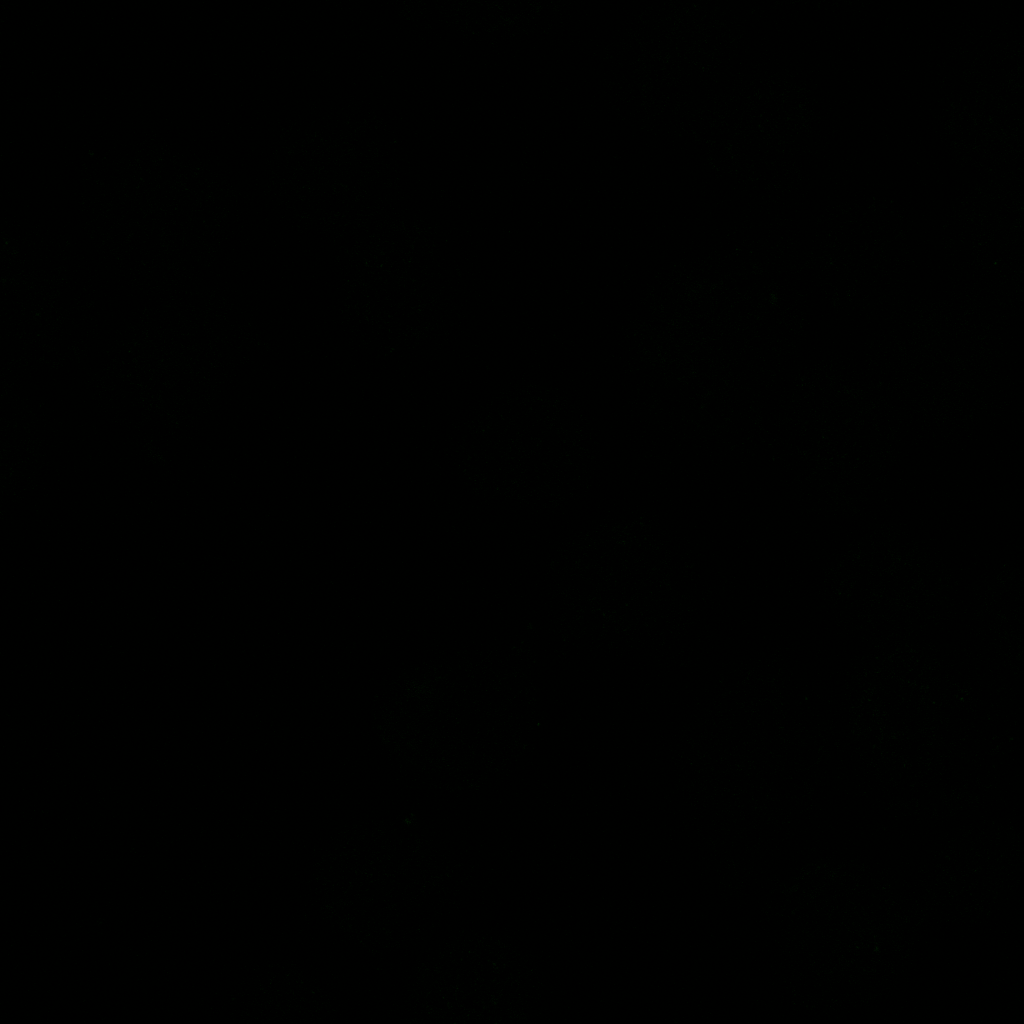

Supplement: Supplementary file 9 — Source Data for Figure 2 [file EMMM-15-e17313-s002.zip › Figure 2/C/Images/DMS114-ATR-c-Image Export-11_c1.tif]

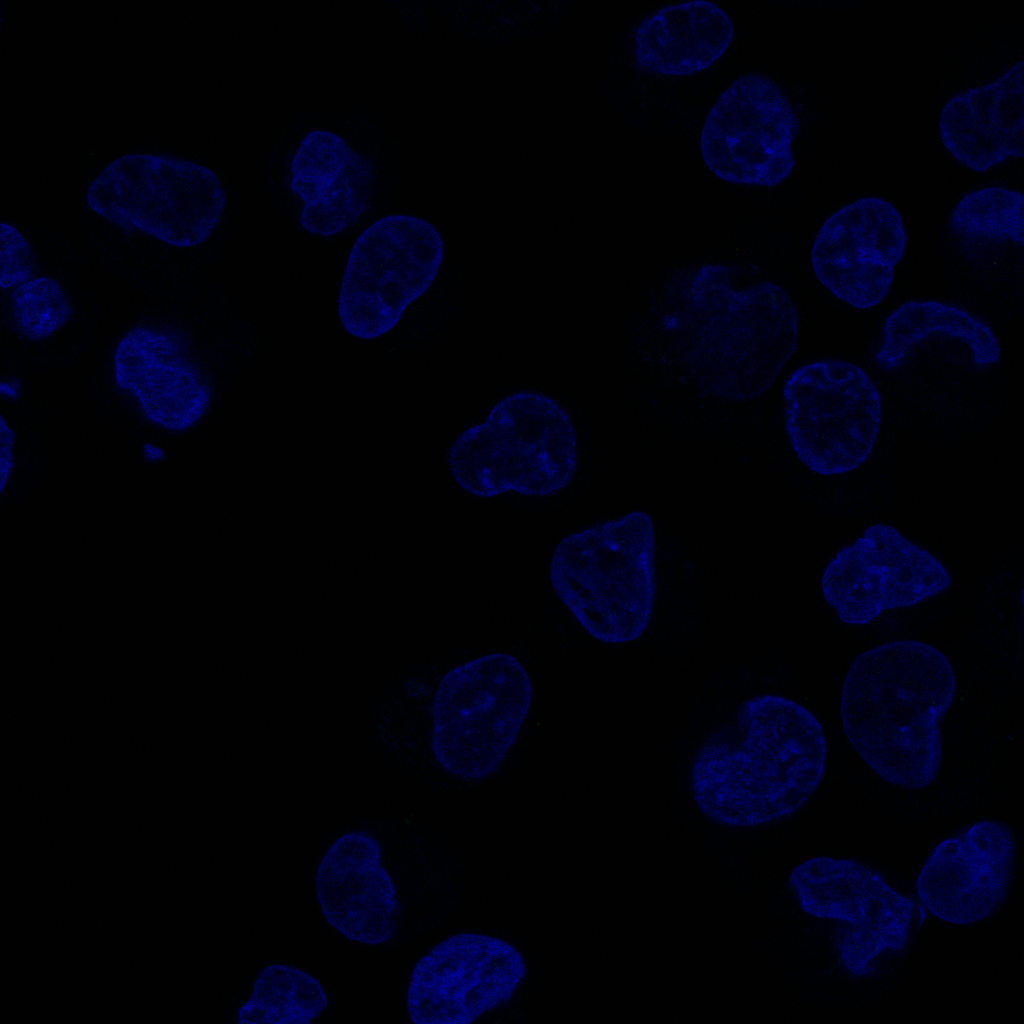

Supplement: Supplementary file 9 — Source Data for Figure 2 [file EMMM-15-e17313-s002.zip › Figure 2/C/Images/DMS114-ATR-c-Image Export-11_c1+2.tif]

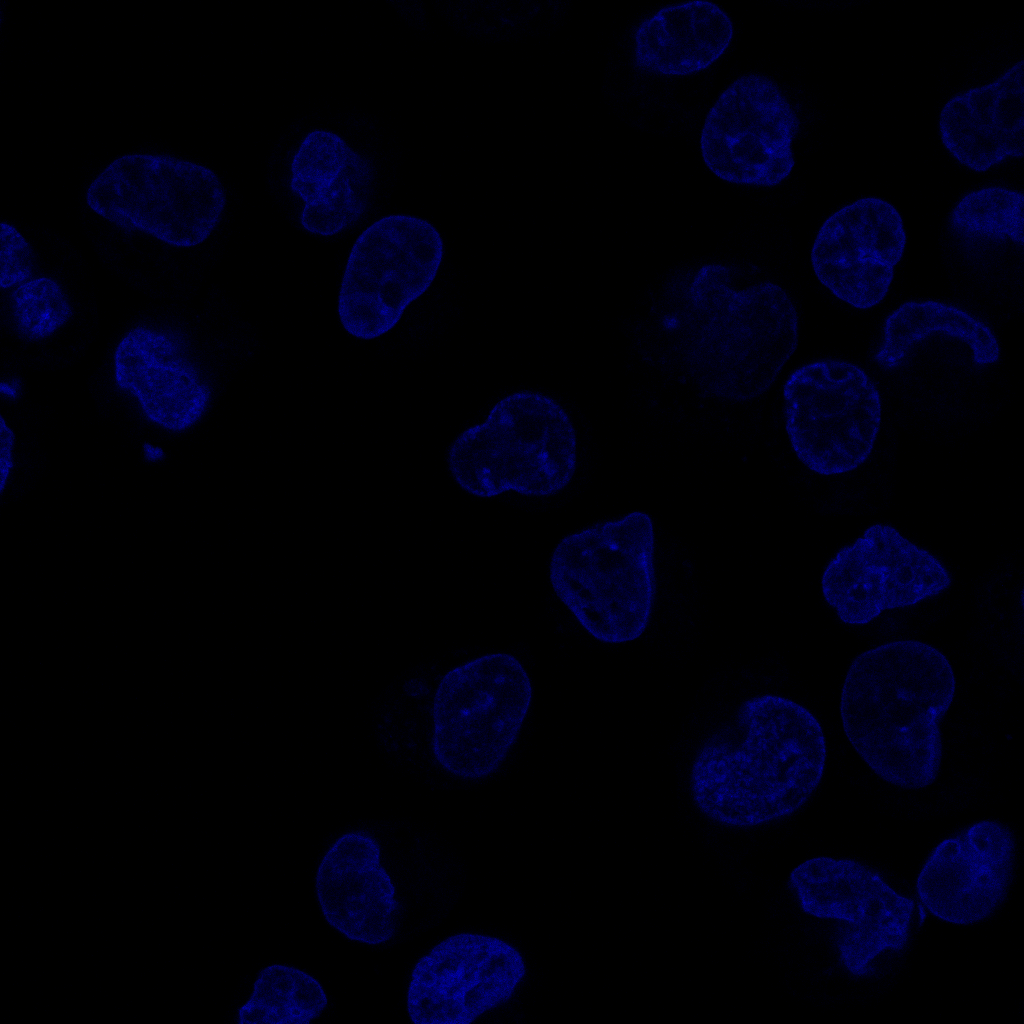

Supplement: Supplementary file 9 — Source Data for Figure 2 [file EMMM-15-e17313-s002.zip › Figure 2/C/Images/DMS114-ATR-c-Image Export-11_c2.tif]

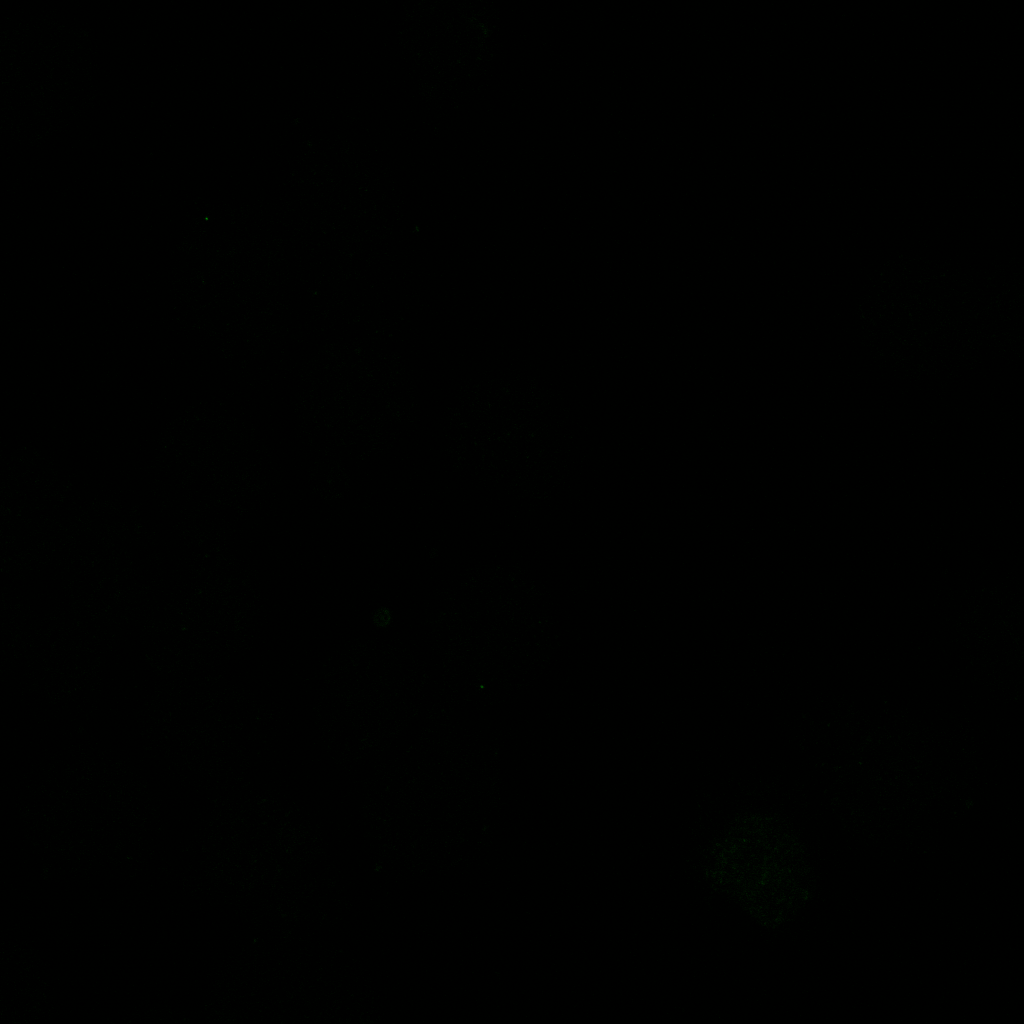

Supplement: Supplementary file 9 — Source Data for Figure 2 [file EMMM-15-e17313-s002.zip › Figure 2/C/Images/DMS114-ATR-d-Image Export-12_c1.tif]

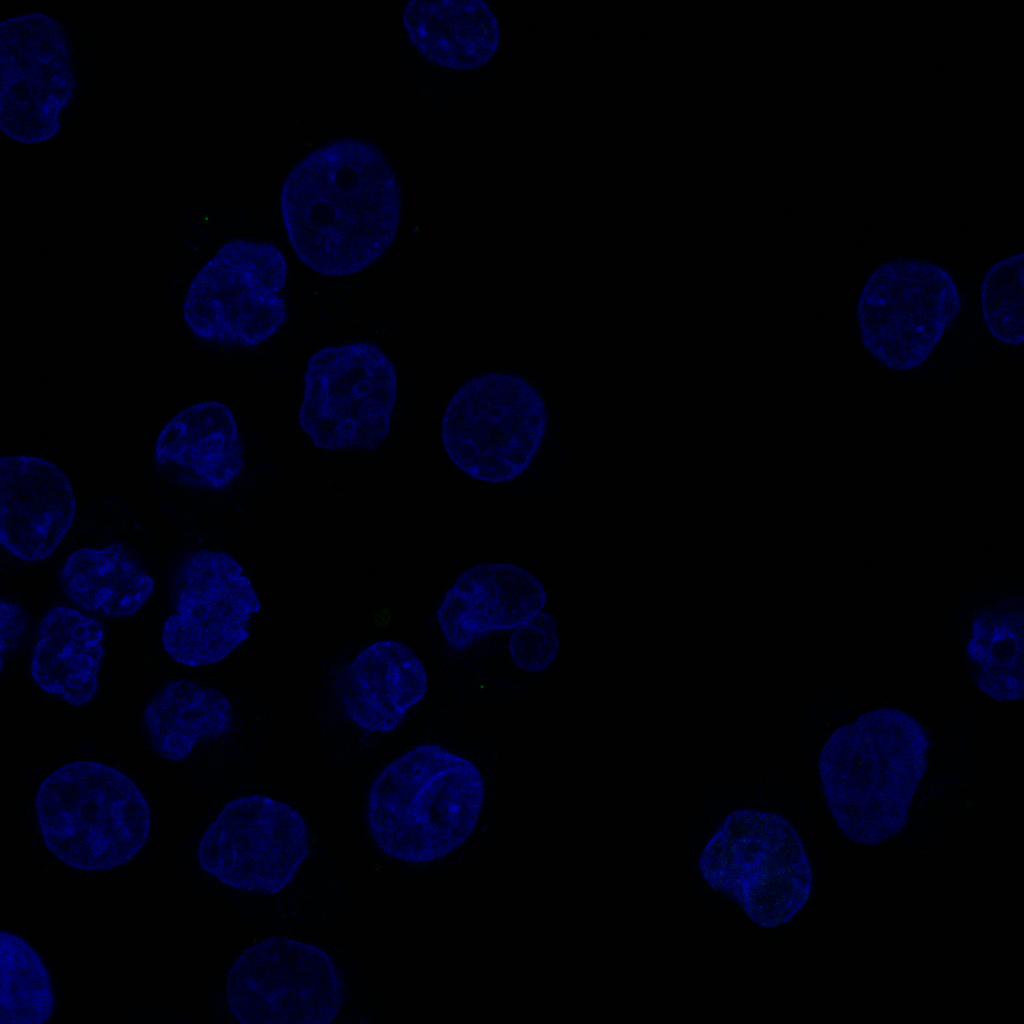

Supplement: Supplementary file 9 — Source Data for Figure 2 [file EMMM-15-e17313-s002.zip › Figure 2/C/Images/DMS114-ATR-d-Image Export-12_c1+2.tif]

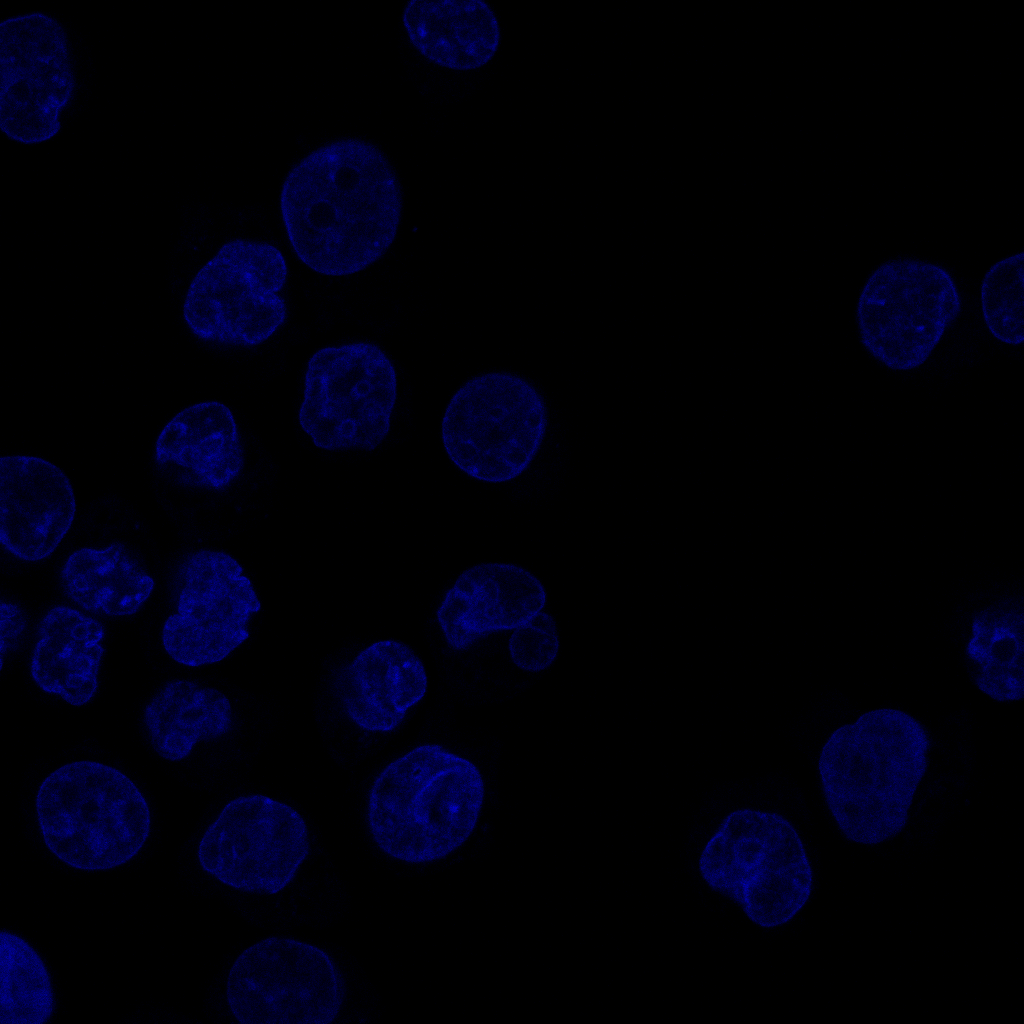

Supplement: Supplementary file 9 — Source Data for Figure 2 [file EMMM-15-e17313-s002.zip › Figure 2/C/Images/DMS114-ATR-d-Image Export-12_c2.tif]

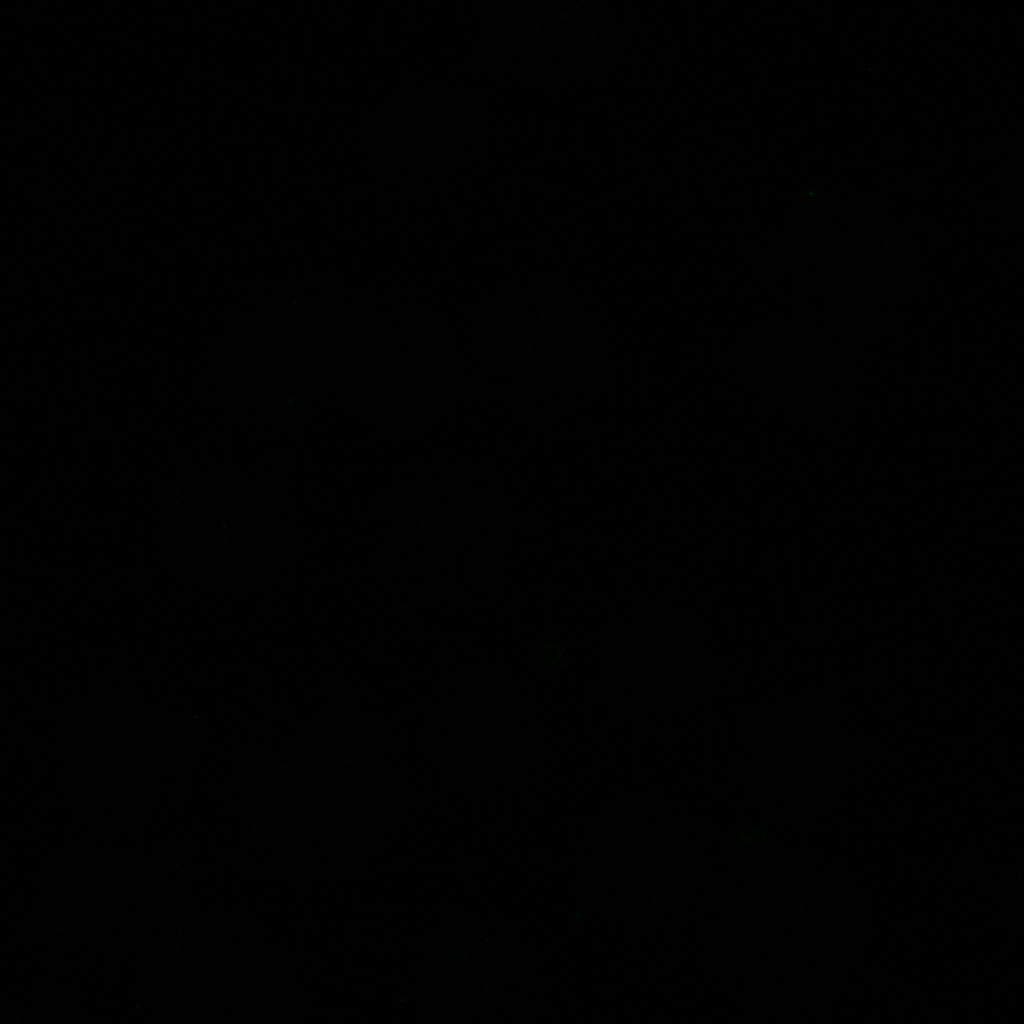

Supplement: Supplementary file 9 — Source Data for Figure 2 [file EMMM-15-e17313-s002.zip › Figure 2/C/Images/DMS114-ATR-e-Image Export-13_c1.tif]

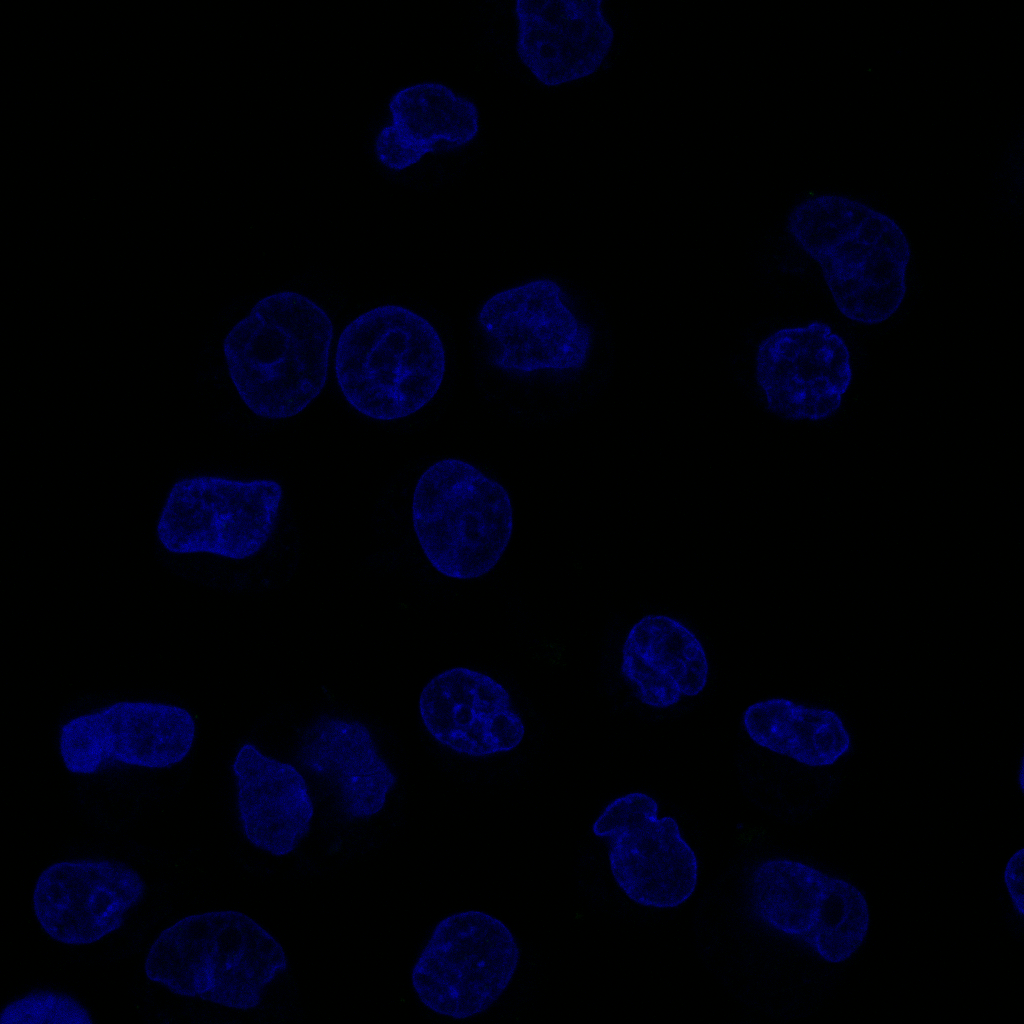

Supplement: Supplementary file 9 — Source Data for Figure 2 [file EMMM-15-e17313-s002.zip › Figure 2/C/Images/DMS114-ATR-e-Image Export-13_c1+2.tif]

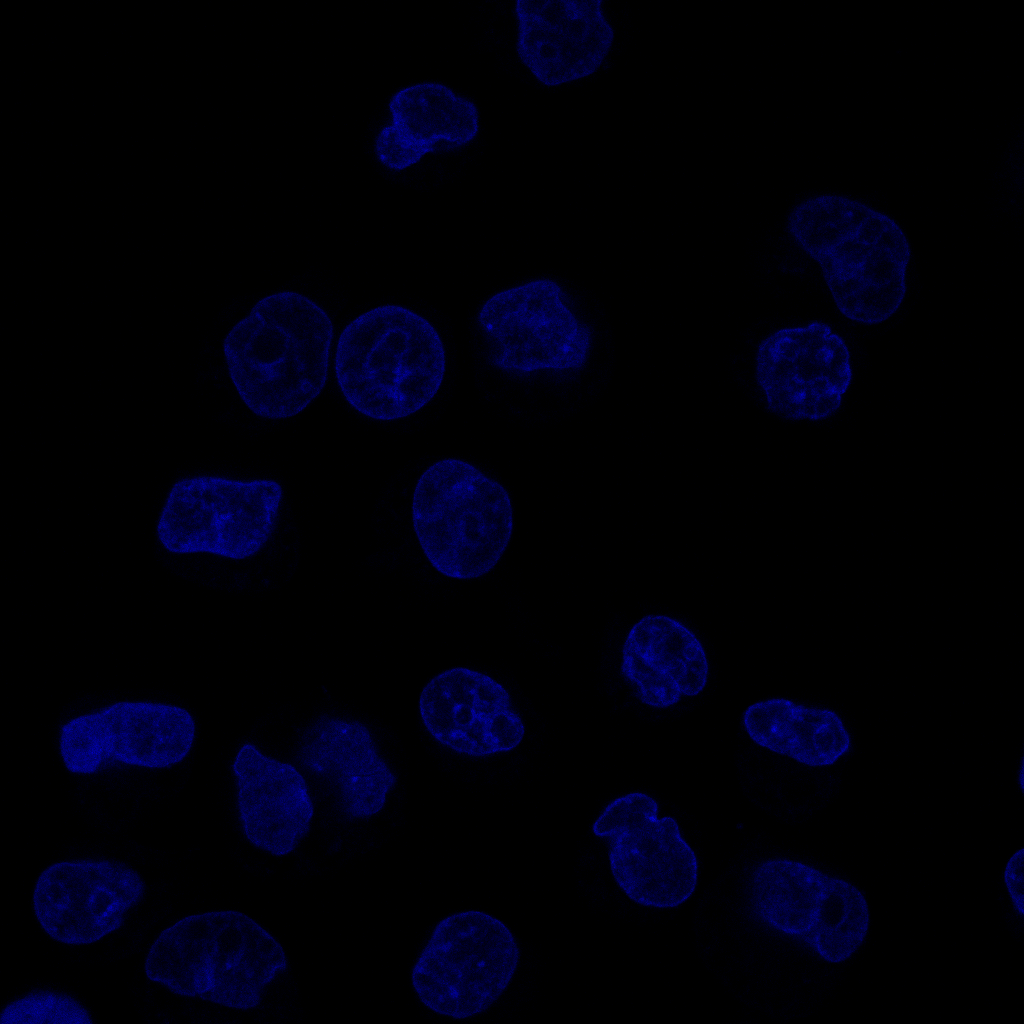

Supplement: Supplementary file 9 — Source Data for Figure 2 [file EMMM-15-e17313-s002.zip › Figure 2/C/Images/DMS114-ATR-e-Image Export-13_c2.tif]

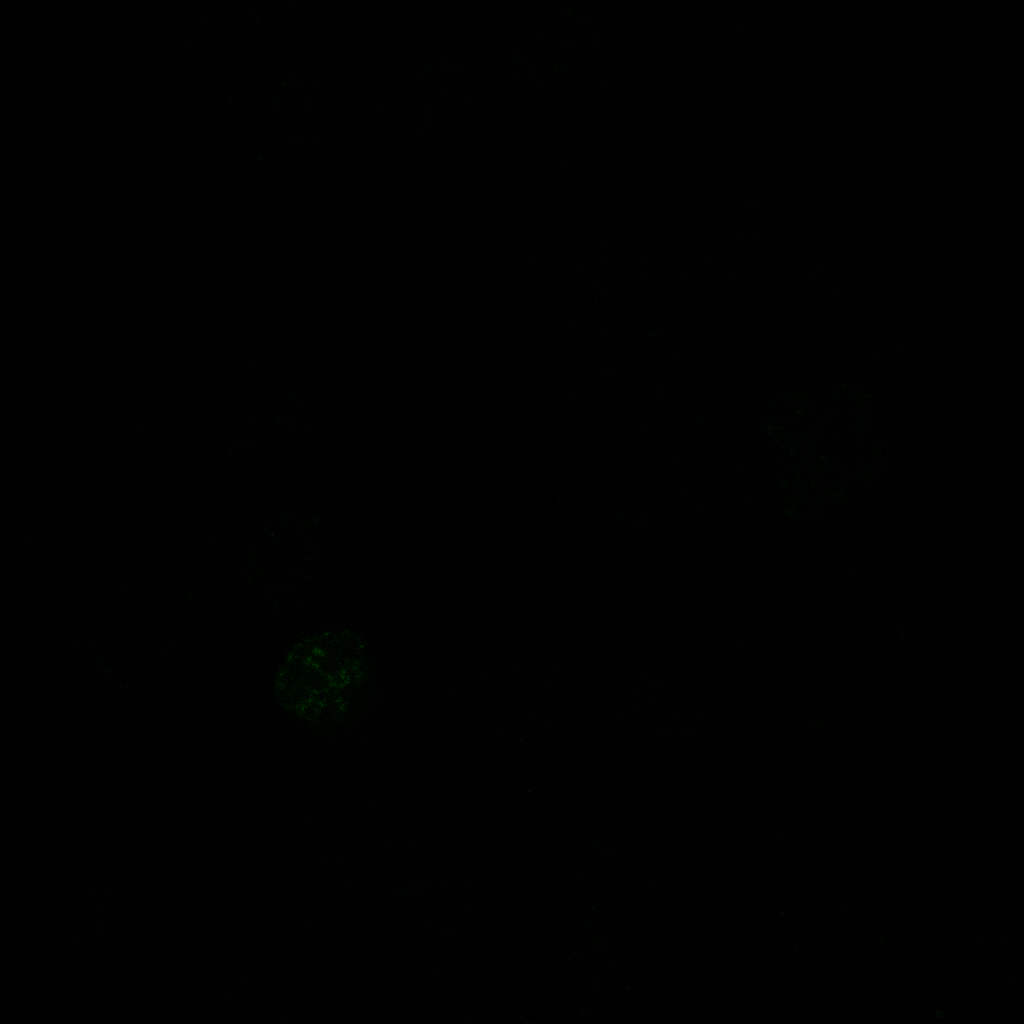

Supplement: Supplementary file 9 — Source Data for Figure 2 [file EMMM-15-e17313-s002.zip › Figure 2/C/Images/DMS114-ATR-f-Image Export-14_c1.tif]

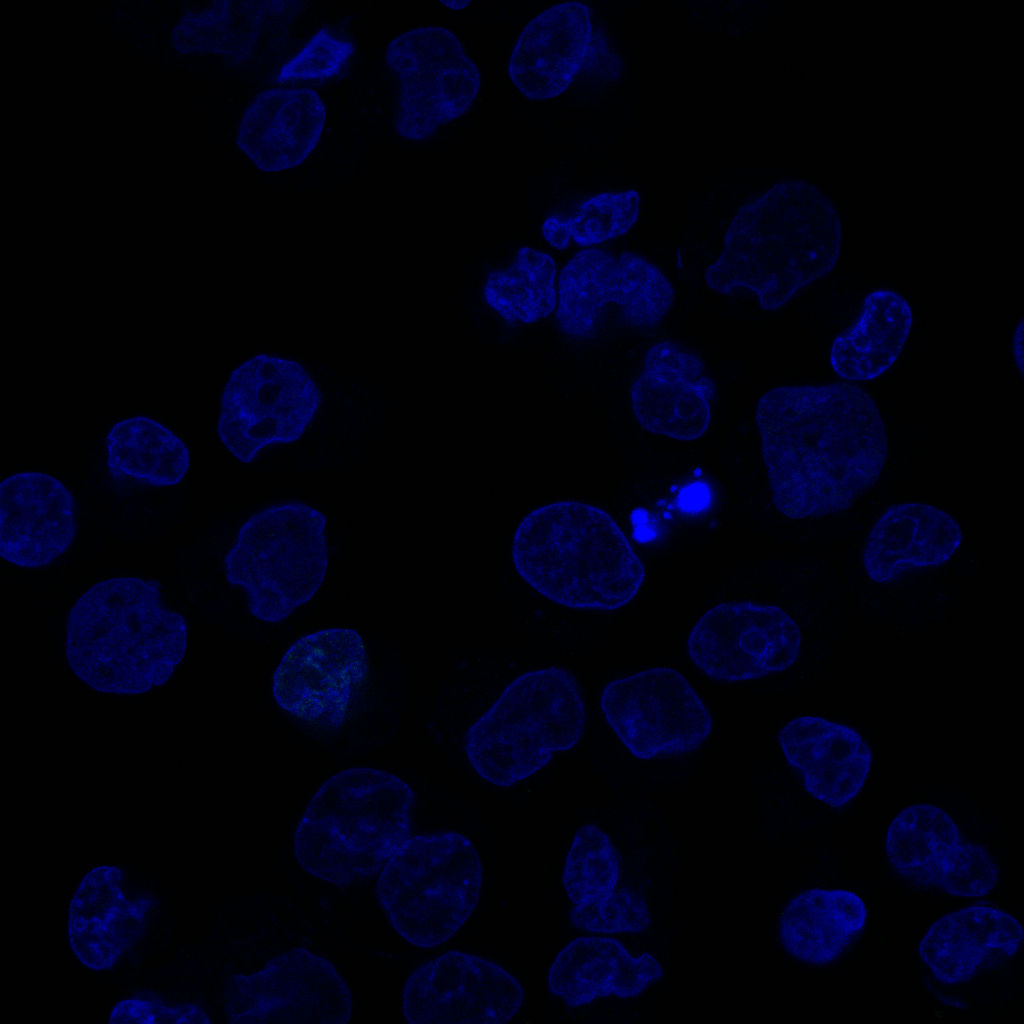

Supplement: Supplementary file 9 — Source Data for Figure 2 [file EMMM-15-e17313-s002.zip › Figure 2/C/Images/DMS114-ATR-f-Image Export-14_c1+2.tif]

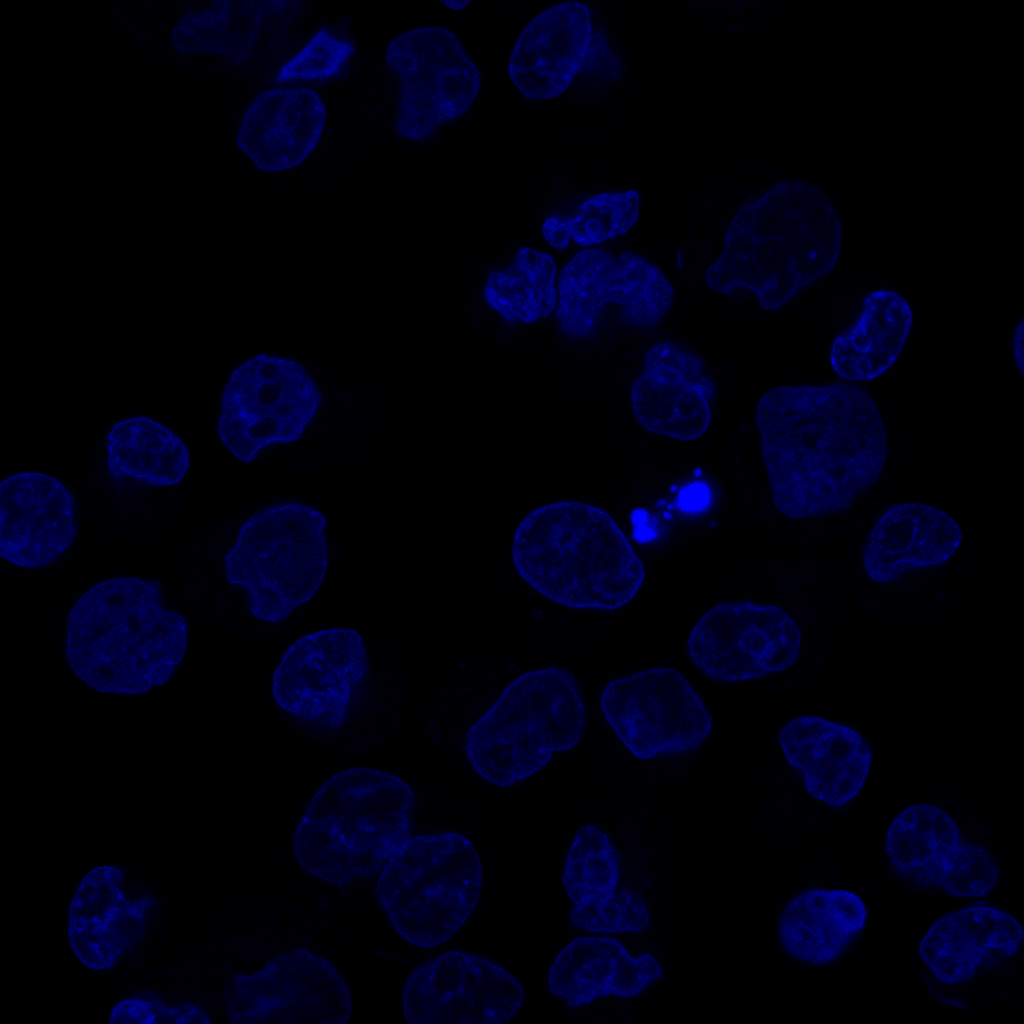

Supplement: Supplementary file 9 — Source Data for Figure 2 [file EMMM-15-e17313-s002.zip › Figure 2/C/Images/DMS114-ATR-f-Image Export-14_c2.tif]

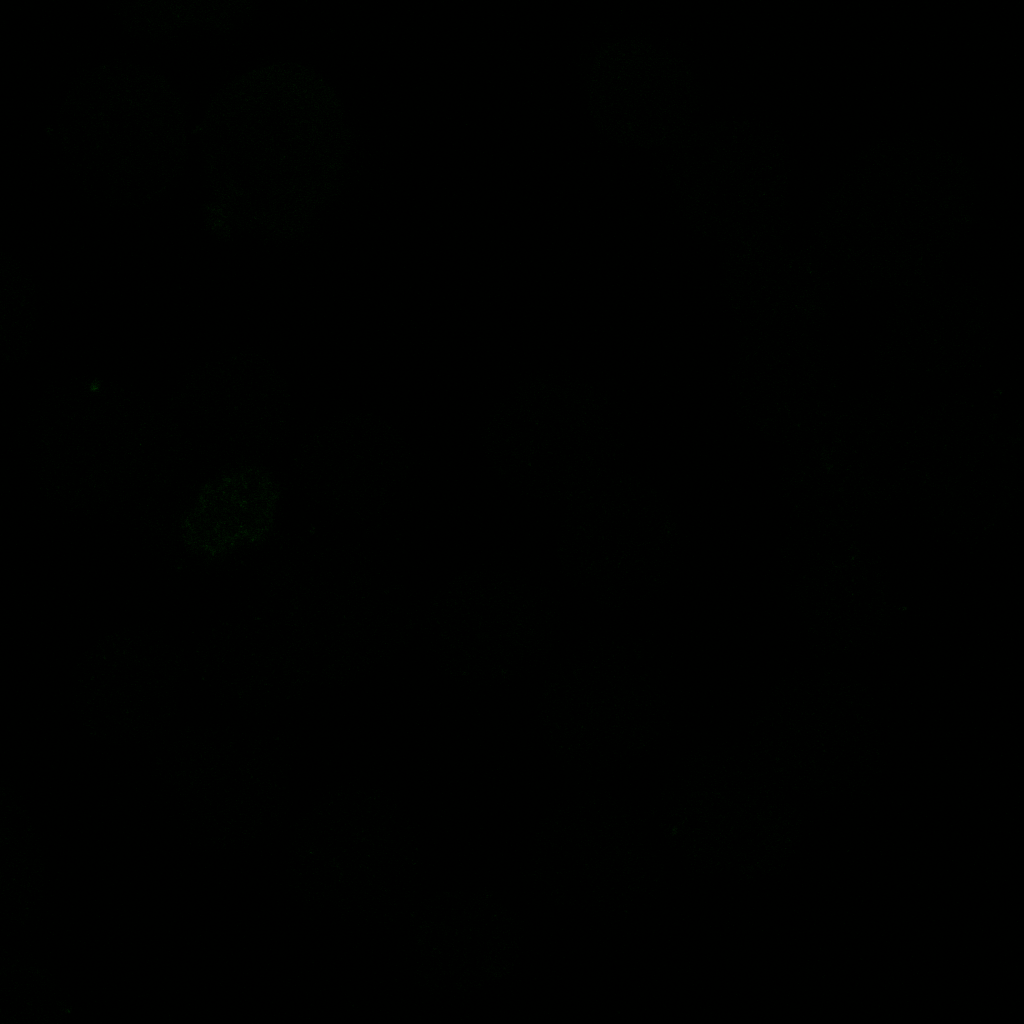

Supplement: Supplementary file 9 — Source Data for Figure 2 [file EMMM-15-e17313-s002.zip › Figure 2/C/Images/DMS114-ATR-g-Image Export-15_c1.tif]

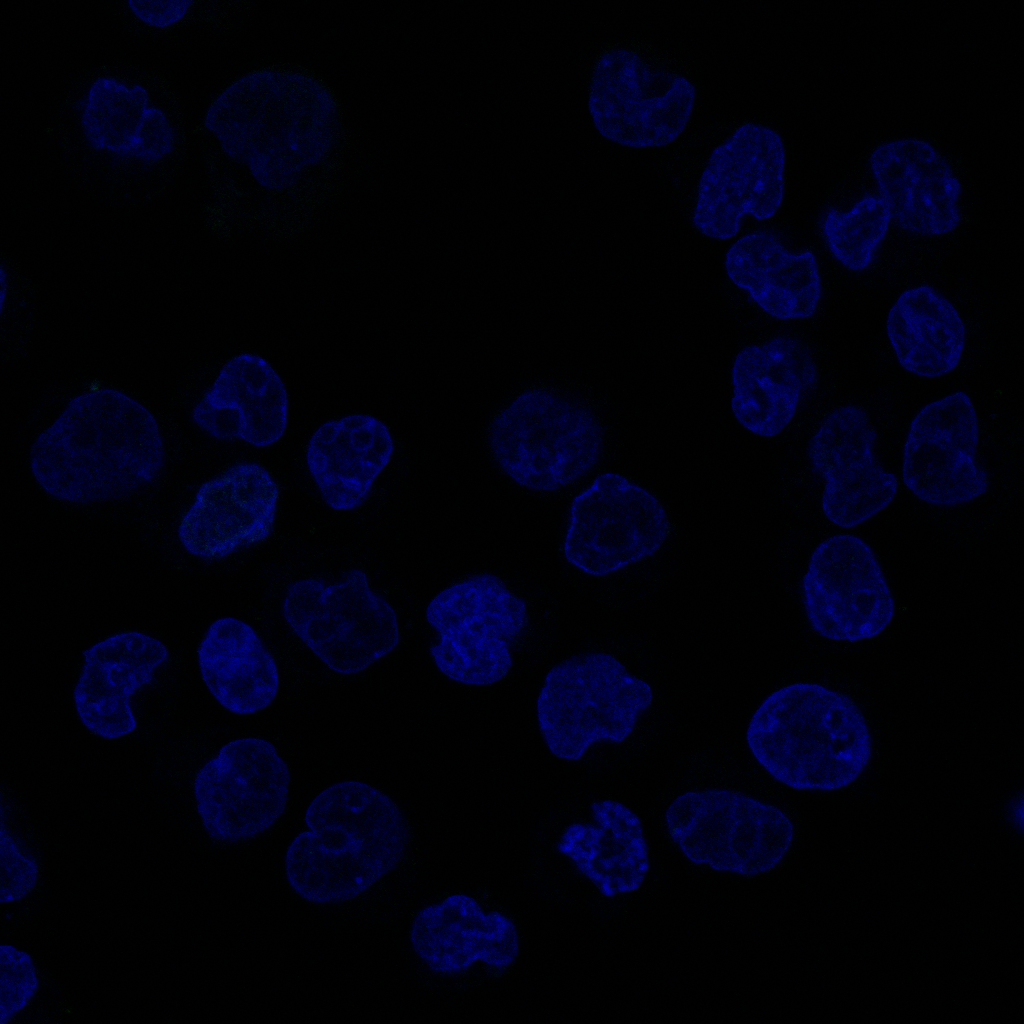

Supplement: Supplementary file 9 — Source Data for Figure 2 [file EMMM-15-e17313-s002.zip › Figure 2/C/Images/DMS114-ATR-g-Image Export-15_c1+2.tif]

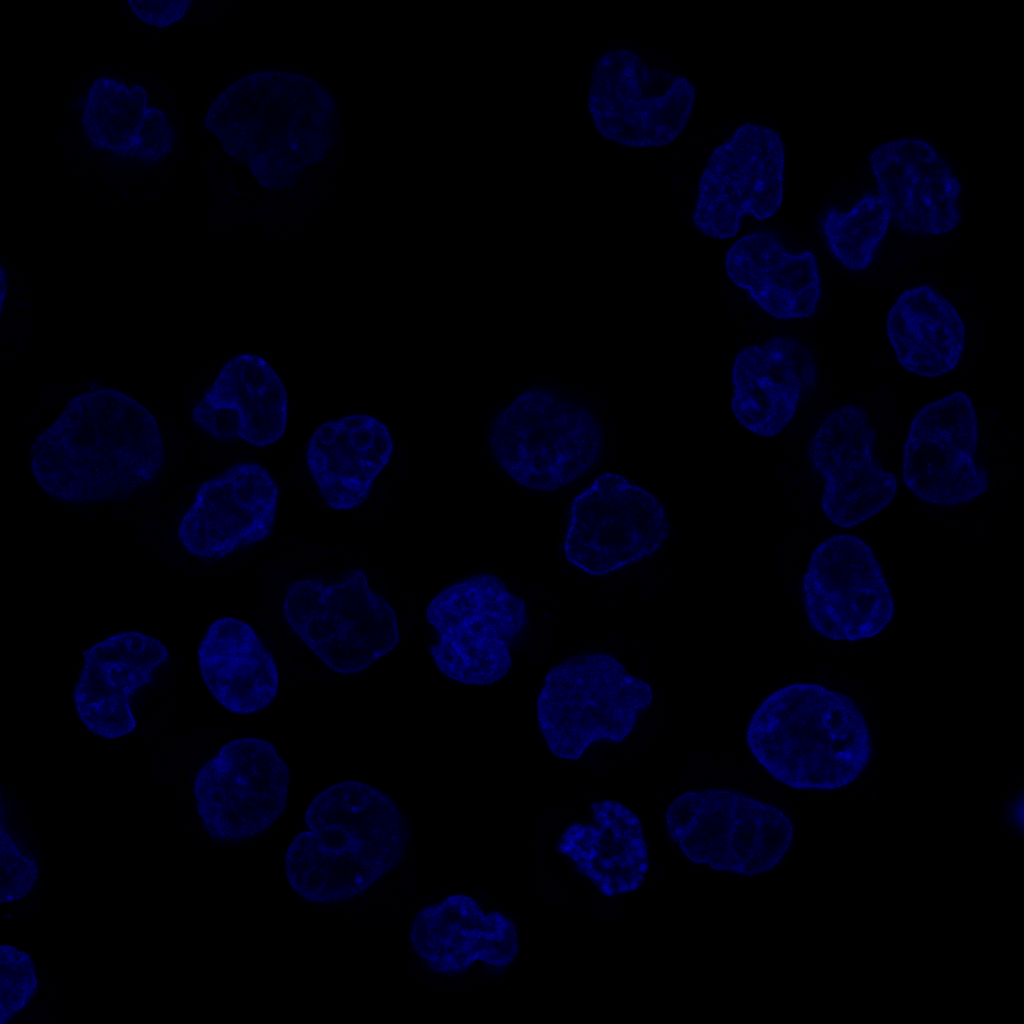

Supplement: Supplementary file 9 — Source Data for Figure 2 [file EMMM-15-e17313-s002.zip › Figure 2/C/Images/DMS114-ATR-g-Image Export-15_c2.tif]

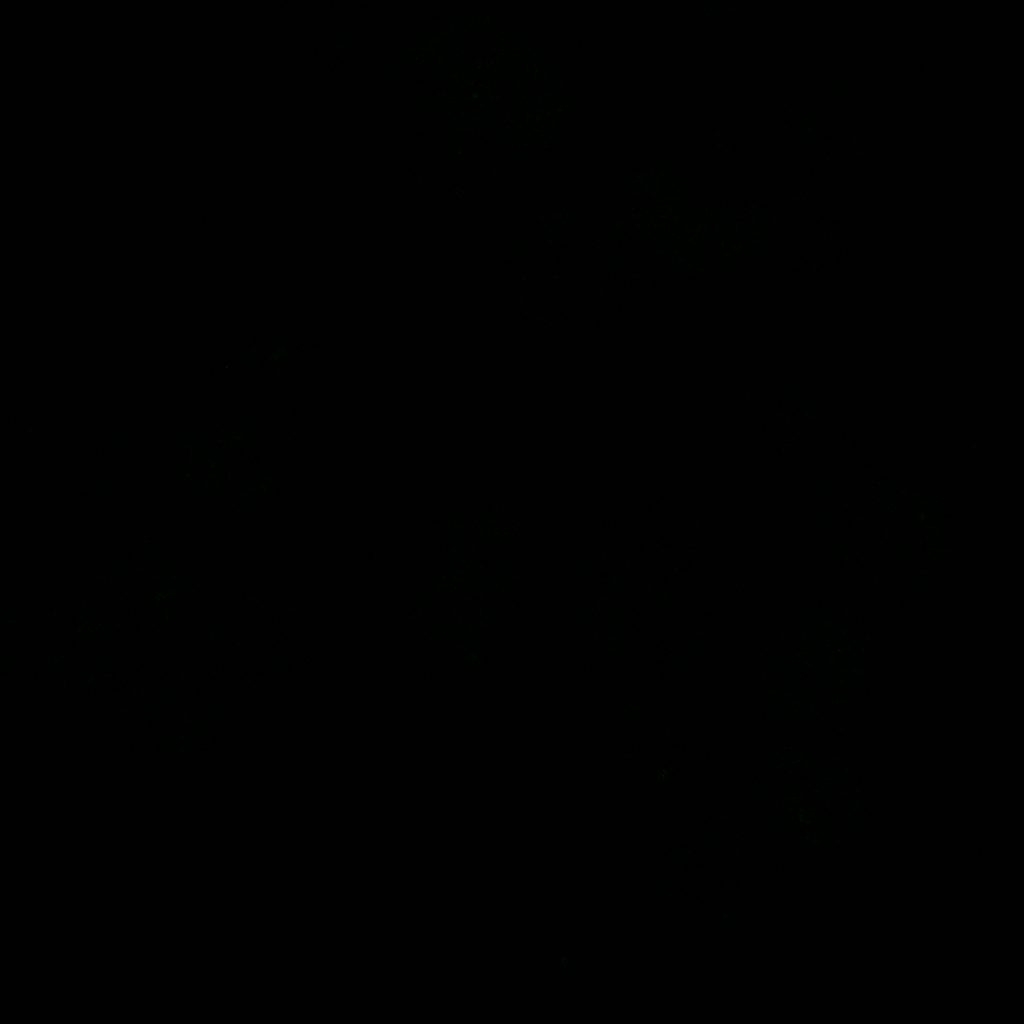

Supplement: Supplementary file 9 — Source Data for Figure 2 [file EMMM-15-e17313-s002.zip › Figure 2/C/Images/DMS114-b-Image Export-16_c1.tif]

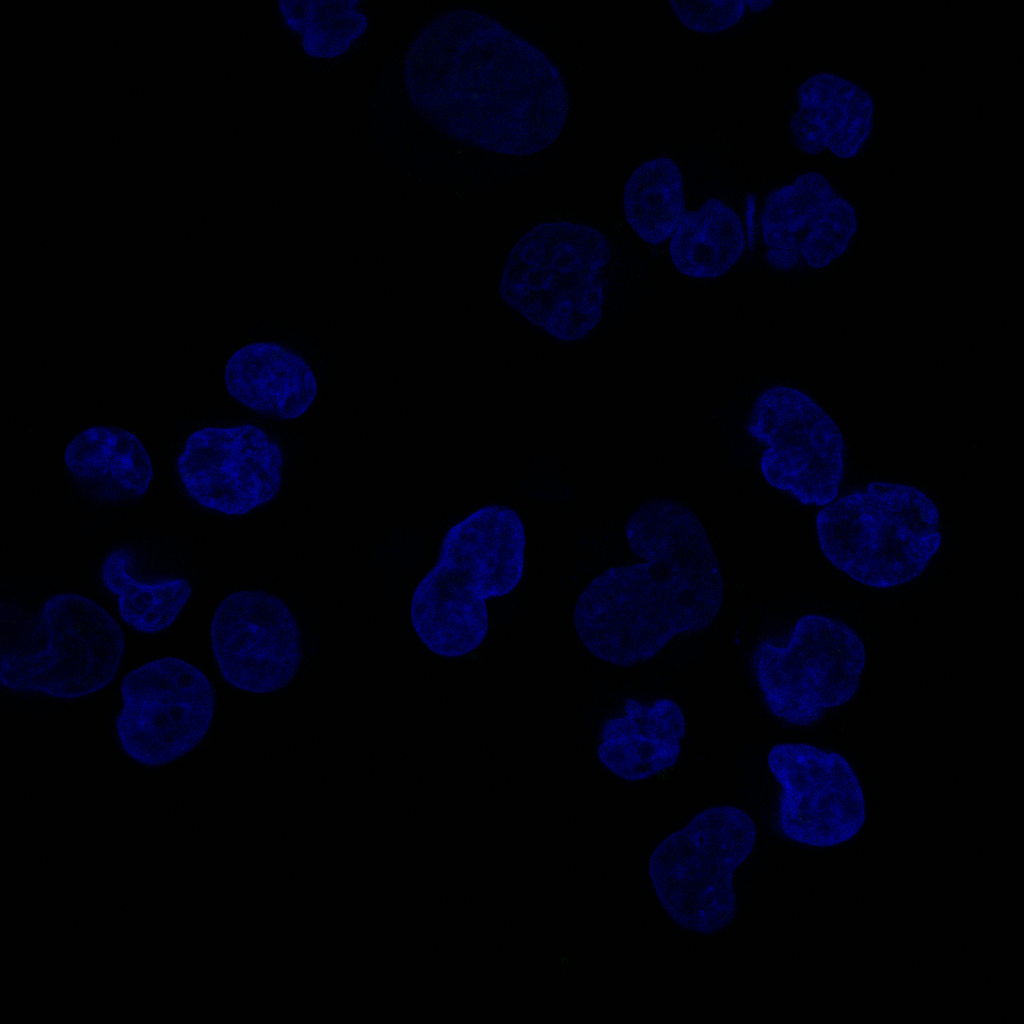

Supplement: Supplementary file 9 — Source Data for Figure 2 [file EMMM-15-e17313-s002.zip › Figure 2/C/Images/DMS114-b-Image Export-16_c1+2.tif]

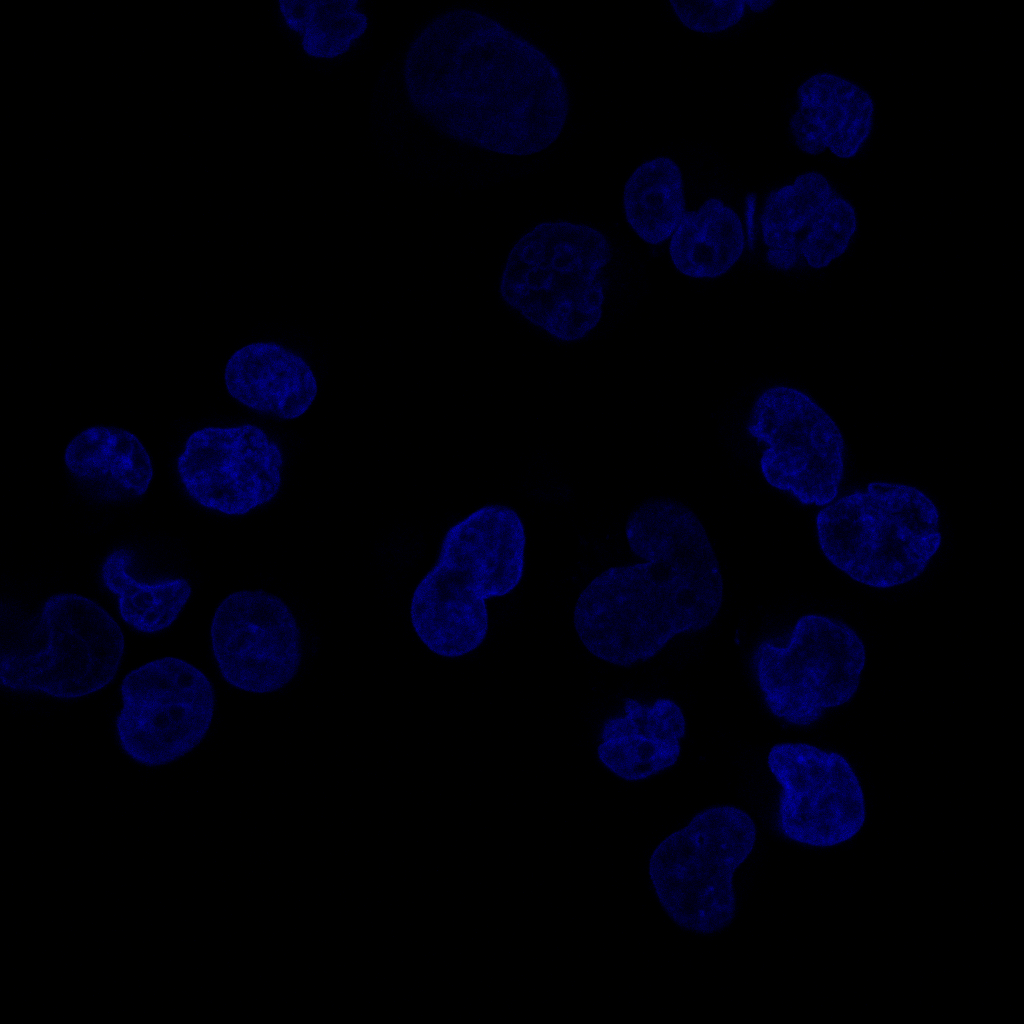

Supplement: Supplementary file 9 — Source Data for Figure 2 [file EMMM-15-e17313-s002.zip › Figure 2/C/Images/DMS114-b-Image Export-16_c2.tif]

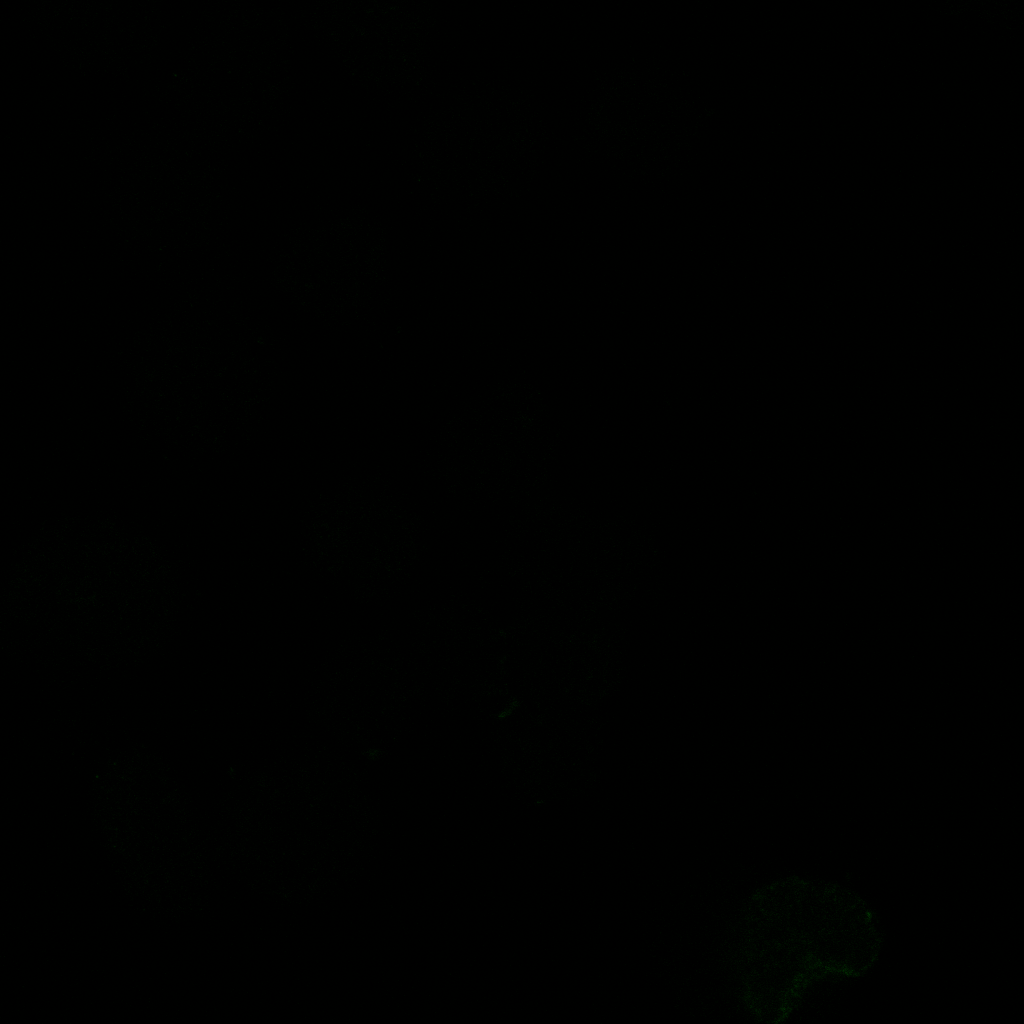

Supplement: Supplementary file 9 — Source Data for Figure 2 [file EMMM-15-e17313-s002.zip › Figure 2/C/Images/DMS114-c-Image Export-17_c1.tif]

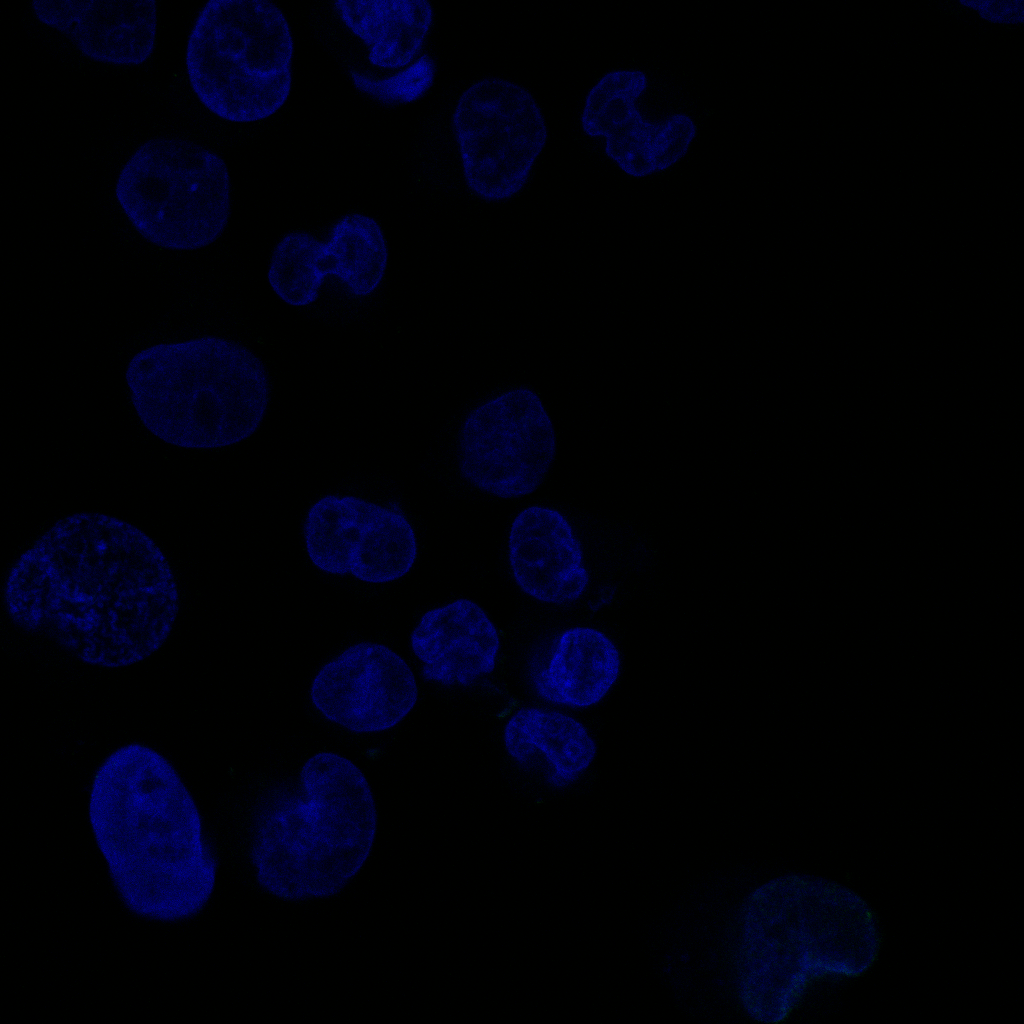

Supplement: Supplementary file 9 — Source Data for Figure 2 [file EMMM-15-e17313-s002.zip › Figure 2/C/Images/DMS114-c-Image Export-17_c1+2.tif]

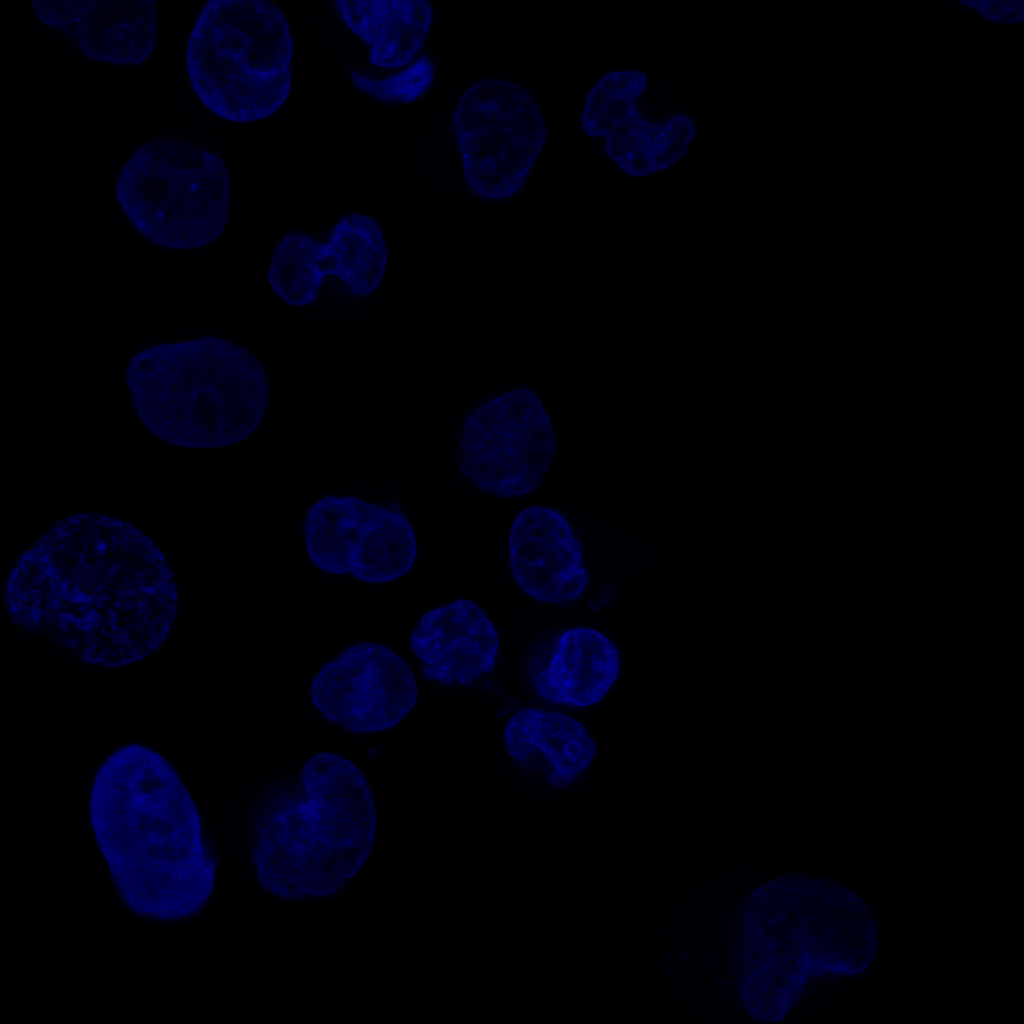

Supplement: Supplementary file 9 — Source Data for Figure 2 [file EMMM-15-e17313-s002.zip › Figure 2/C/Images/DMS114-c-Image Export-17_c2.tif]

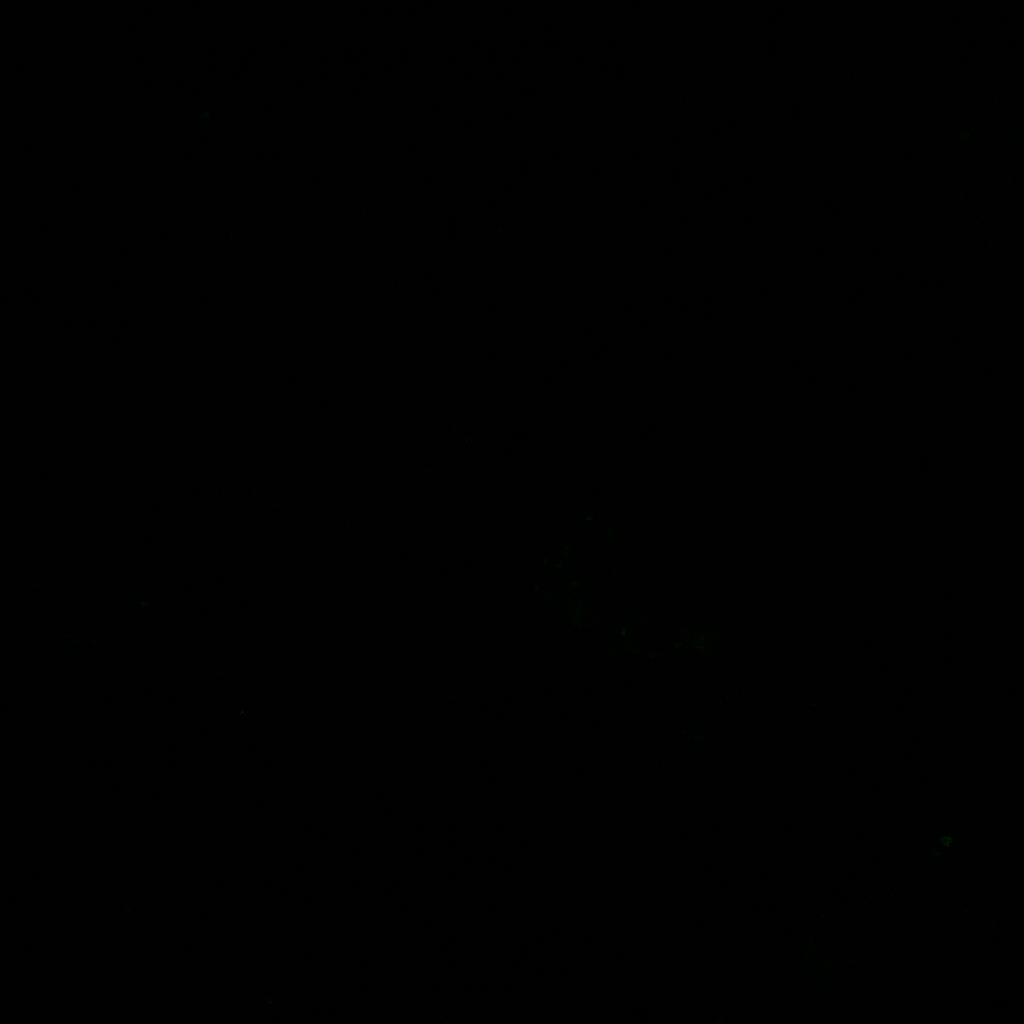

Supplement: Supplementary file 9 — Source Data for Figure 2 [file EMMM-15-e17313-s002.zip › Figure 2/C/Images/DMS114-d-Image Export-18_c1.tif]

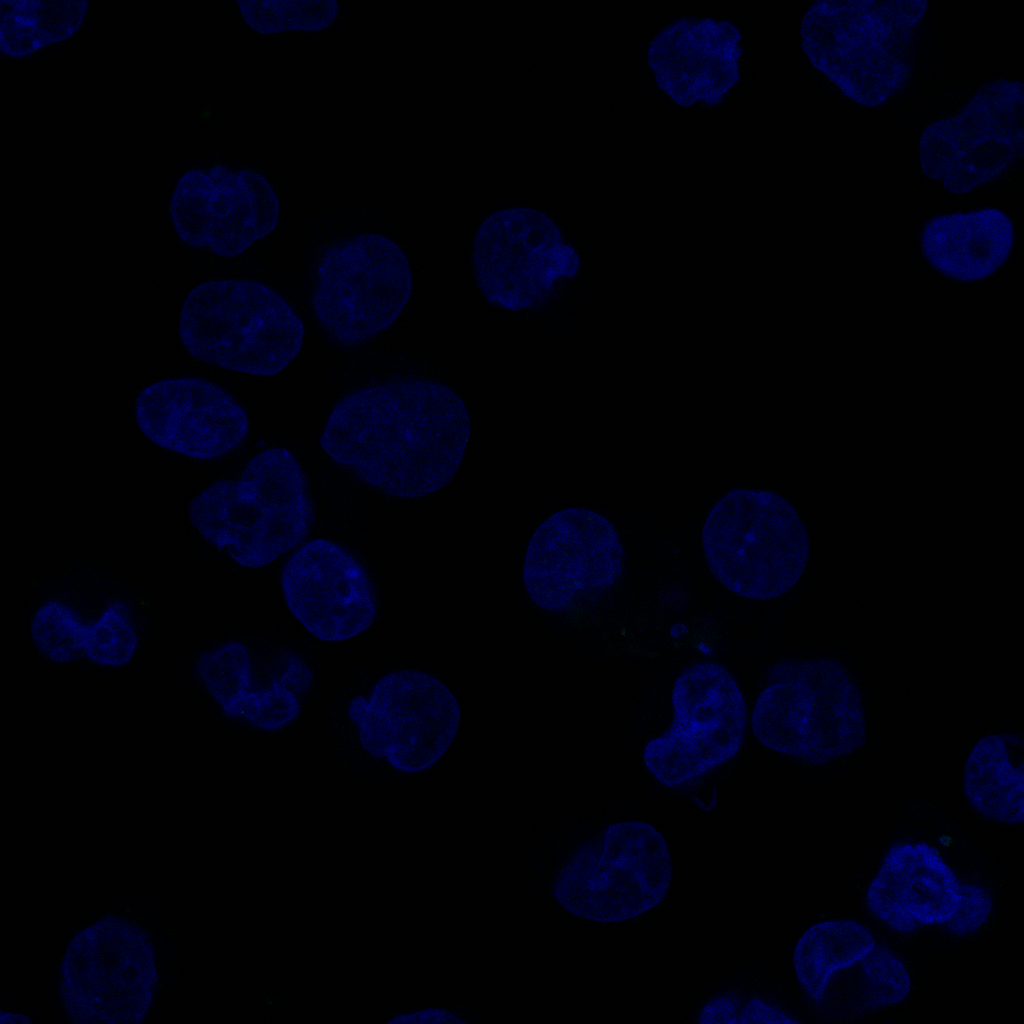

Supplement: Supplementary file 9 — Source Data for Figure 2 [file EMMM-15-e17313-s002.zip › Figure 2/C/Images/DMS114-d-Image Export-18_c1+2.tif]

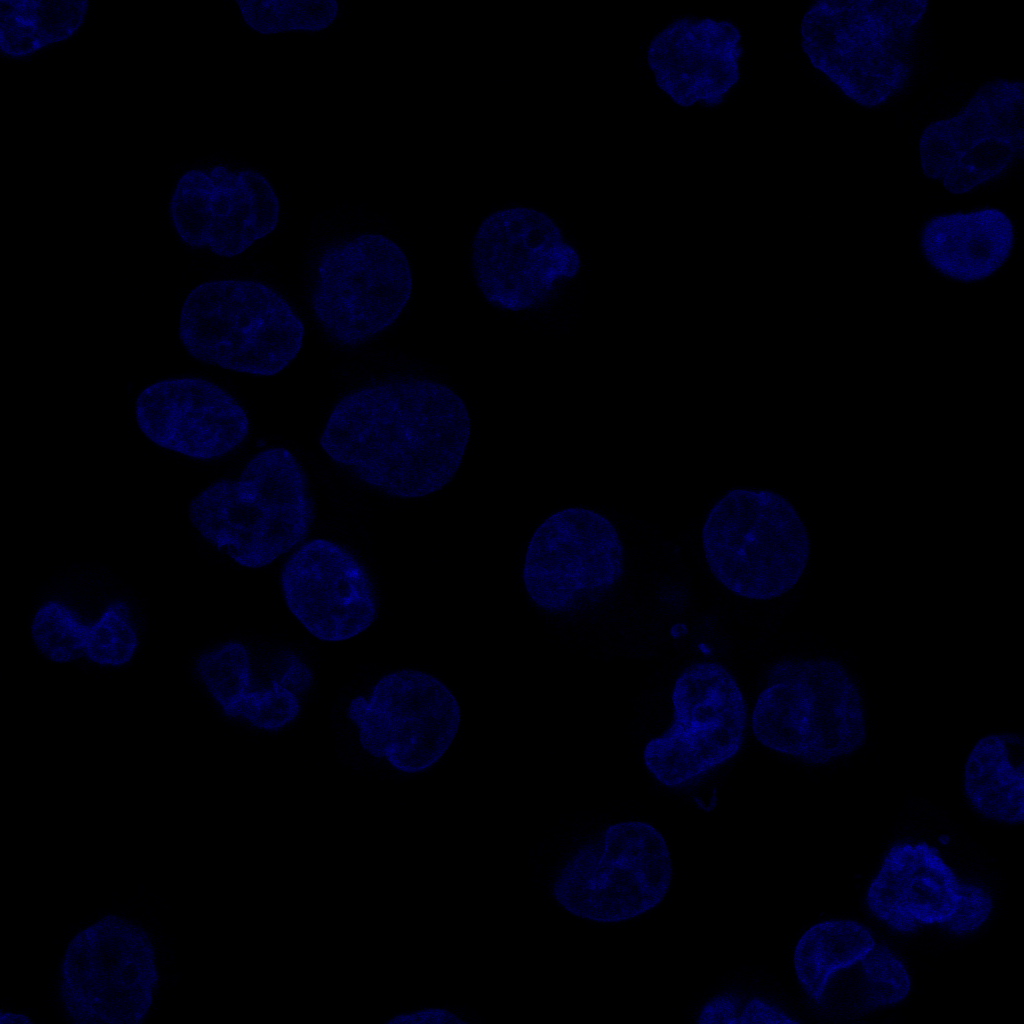

Supplement: Supplementary file 9 — Source Data for Figure 2 [file EMMM-15-e17313-s002.zip › Figure 2/C/Images/DMS114-d-Image Export-18_c2.tif]

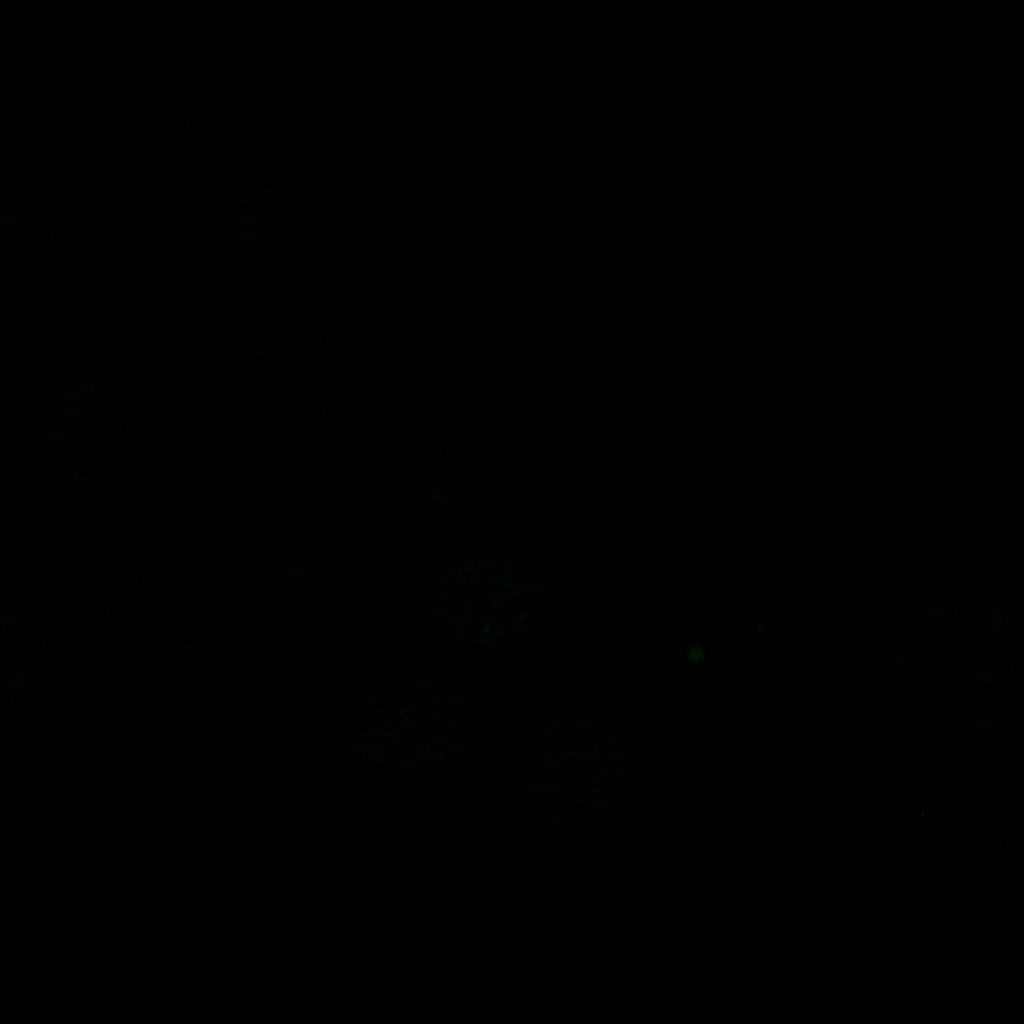

Supplement: Supplementary file 9 — Source Data for Figure 2 [file EMMM-15-e17313-s002.zip › Figure 2/C/Images/DMS114-e-Image Export-19_c1.tif]

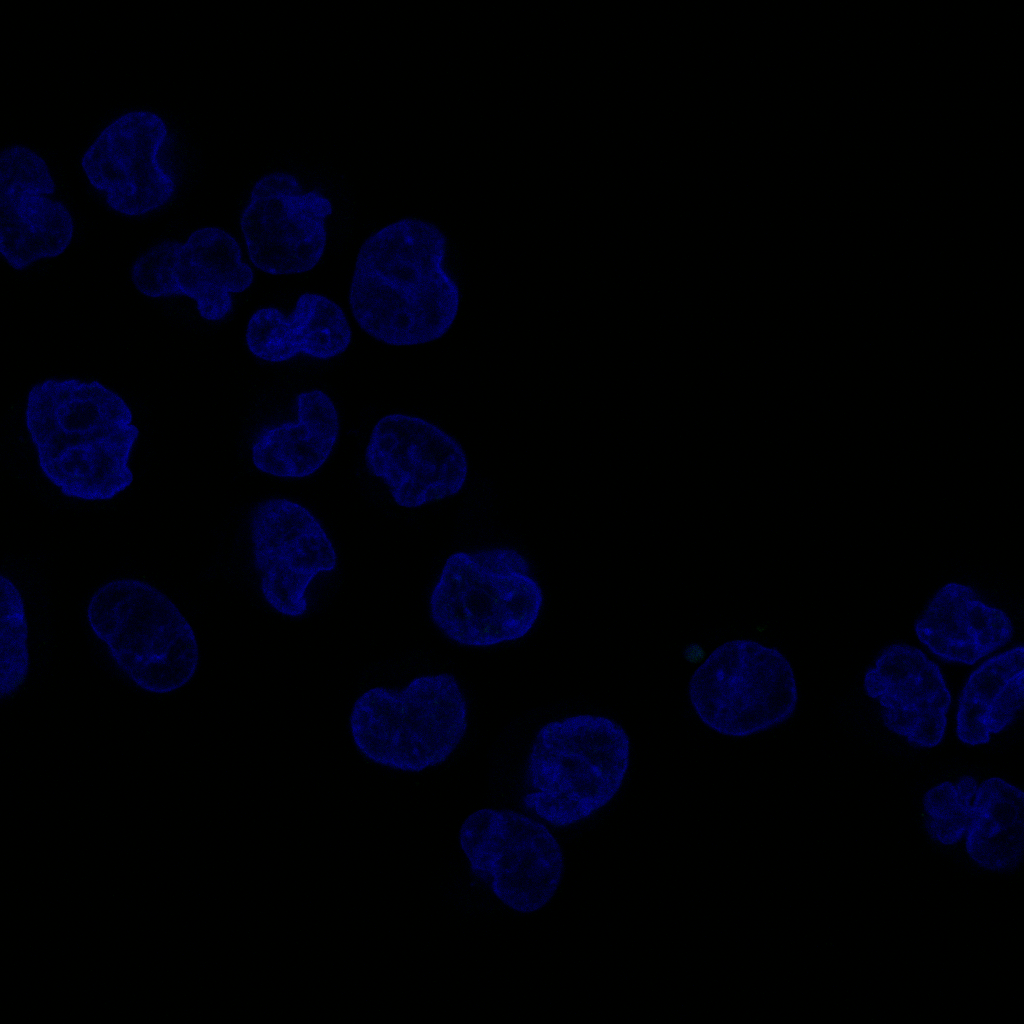

Supplement: Supplementary file 9 — Source Data for Figure 2 [file EMMM-15-e17313-s002.zip › Figure 2/C/Images/DMS114-e-Image Export-19_c1+2.tif]

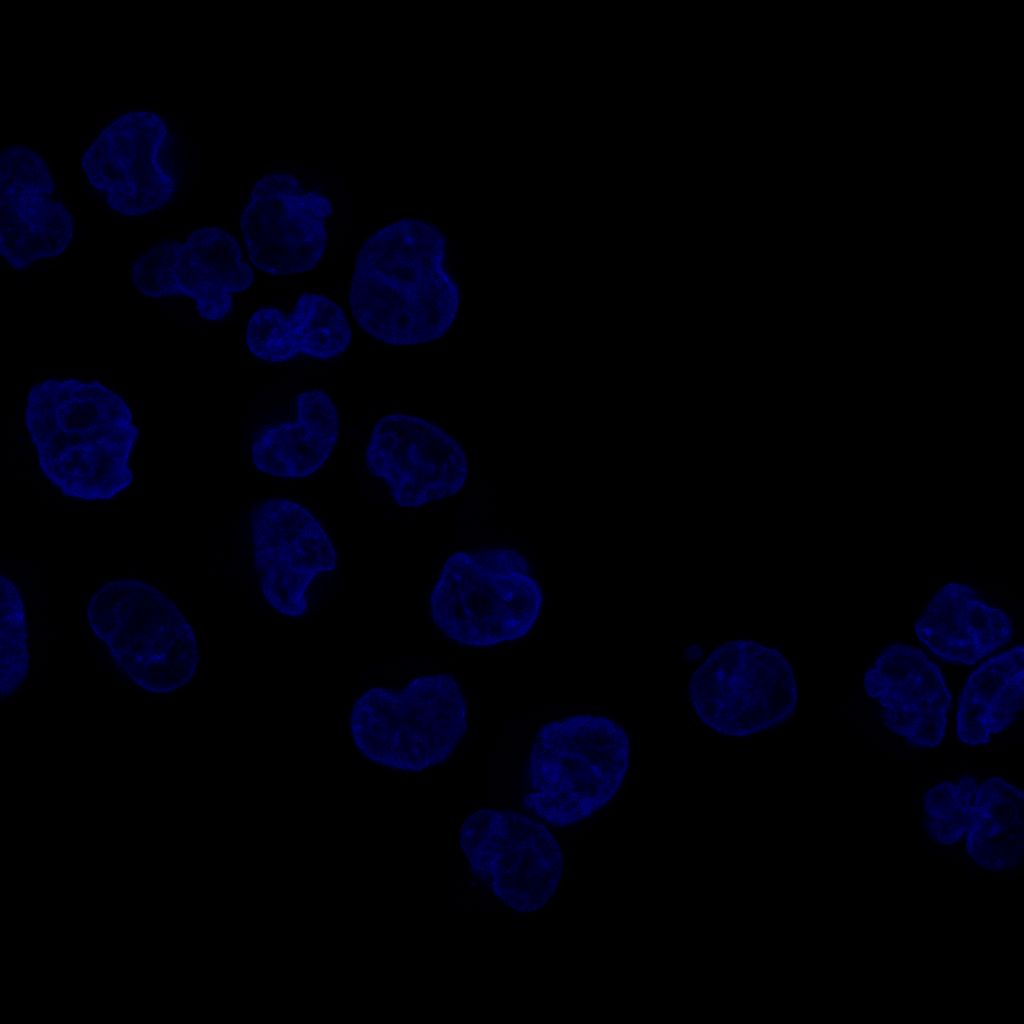

Supplement: Supplementary file 9 — Source Data for Figure 2 [file EMMM-15-e17313-s002.zip › Figure 2/C/Images/DMS114-e-Image Export-19_c2.tif]

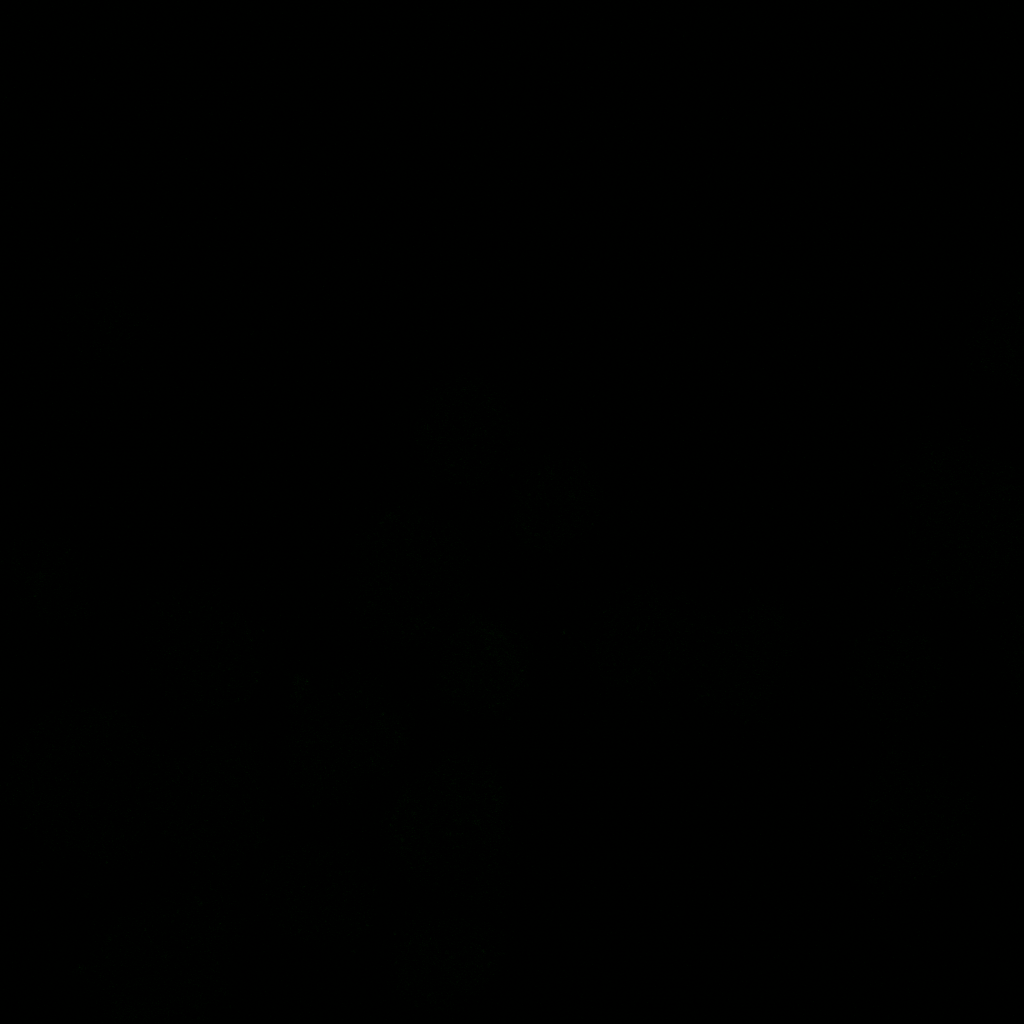

Supplement: Supplementary file 9 — Source Data for Figure 2 [file EMMM-15-e17313-s002.zip › Figure 2/C/Images/DMS114-f-Image Export-20_c1.tif]

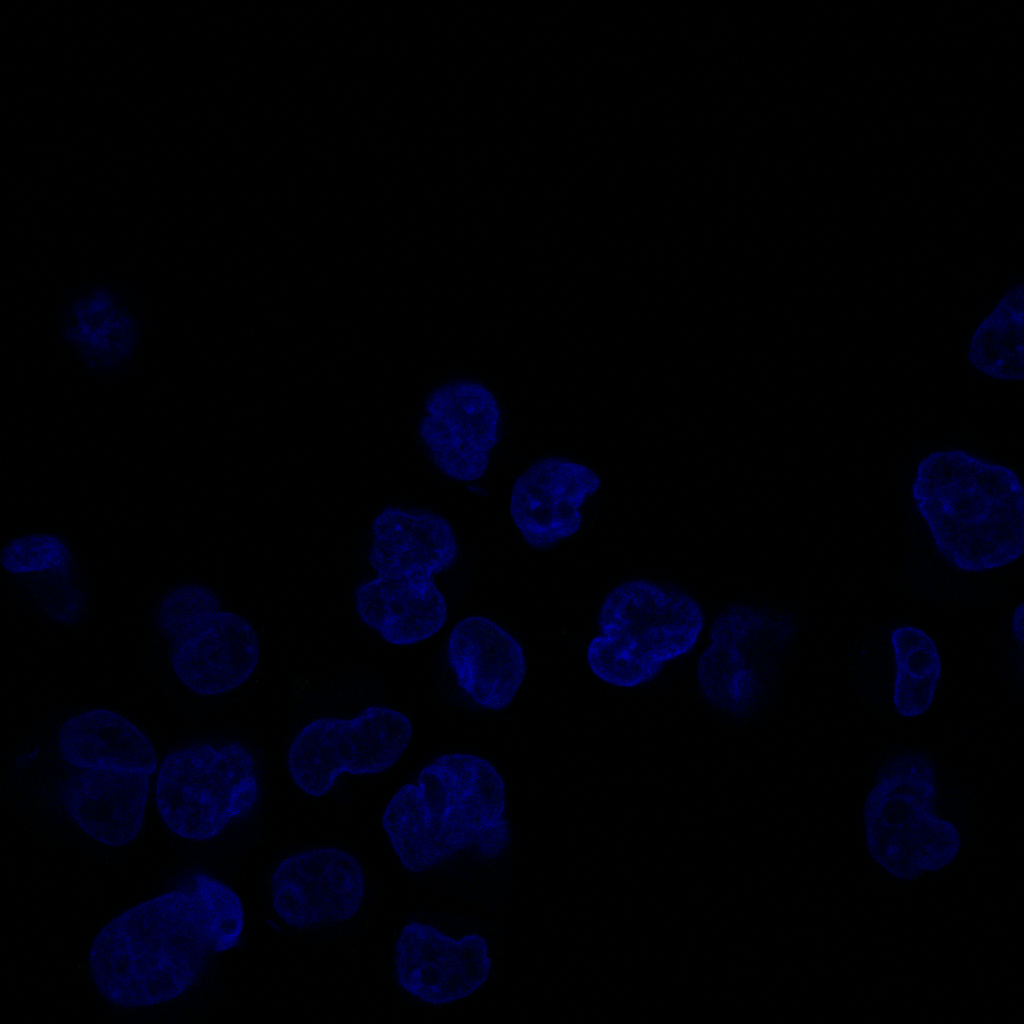

Supplement: Supplementary file 9 — Source Data for Figure 2 [file EMMM-15-e17313-s002.zip › Figure 2/C/Images/DMS114-f-Image Export-20_c1+2.tif]

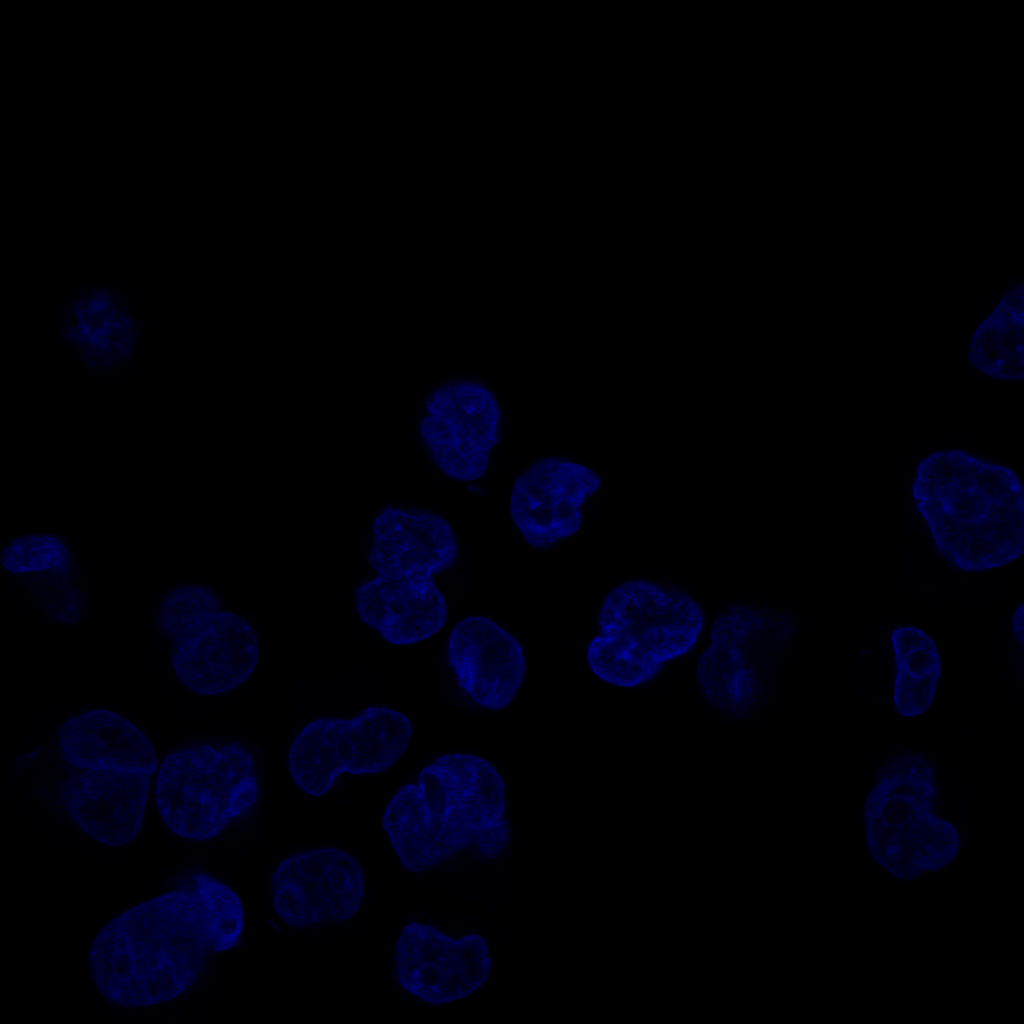

Supplement: Supplementary file 9 — Source Data for Figure 2 [file EMMM-15-e17313-s002.zip › Figure 2/C/Images/DMS114-f-Image Export-20_c2.tif]

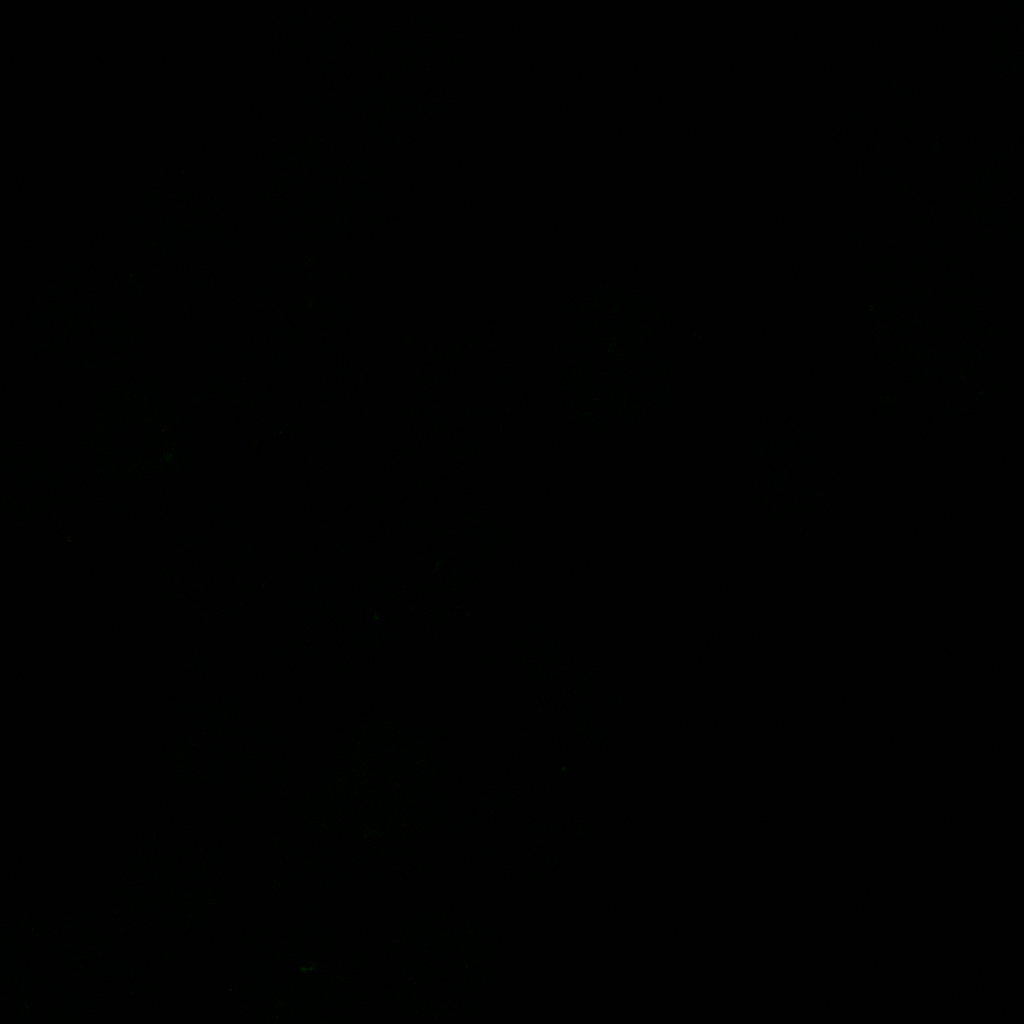

Supplement: Supplementary file 9 — Source Data for Figure 2 [file EMMM-15-e17313-s002.zip › Figure 2/C/Images/DMS114-g-Image Export-21_c1.tif]

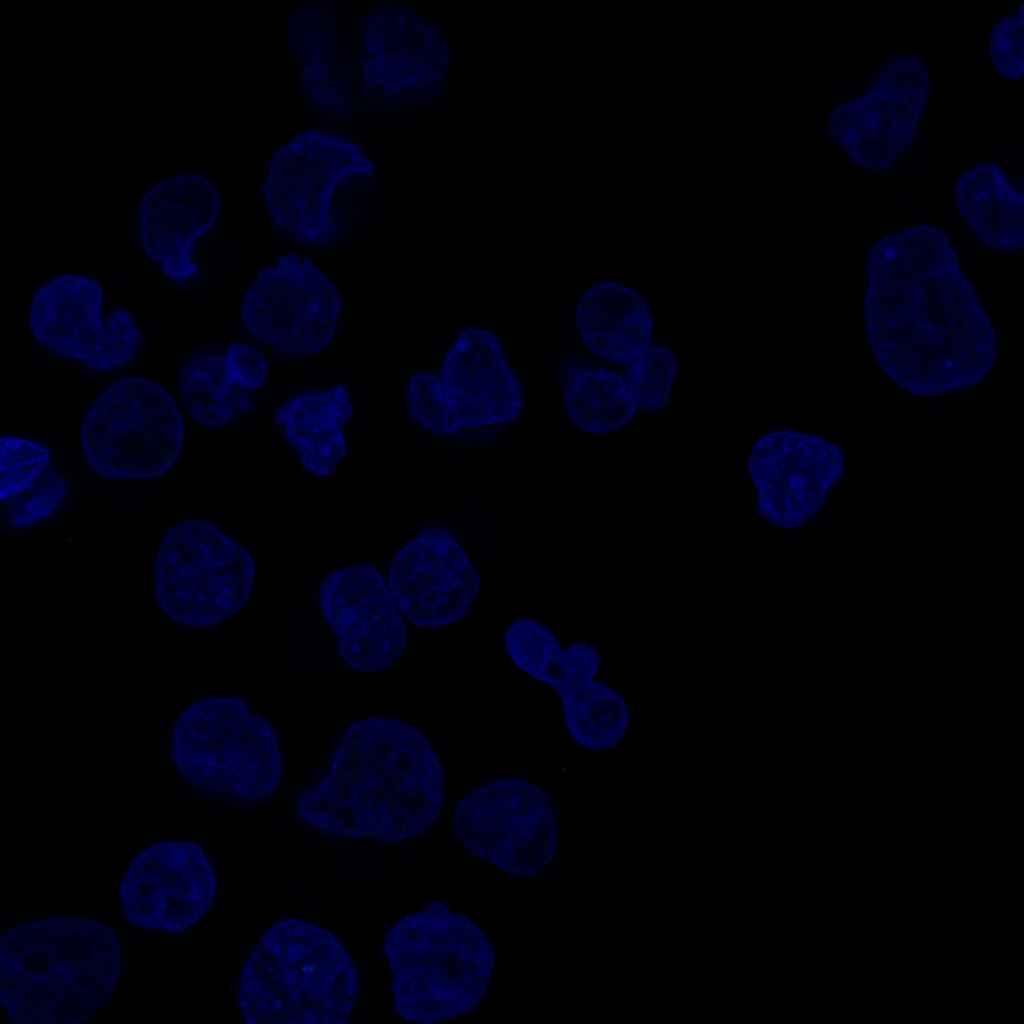

Supplement: Supplementary file 9 — Source Data for Figure 2 [file EMMM-15-e17313-s002.zip › Figure 2/C/Images/DMS114-g-Image Export-21_c1+2.tif]

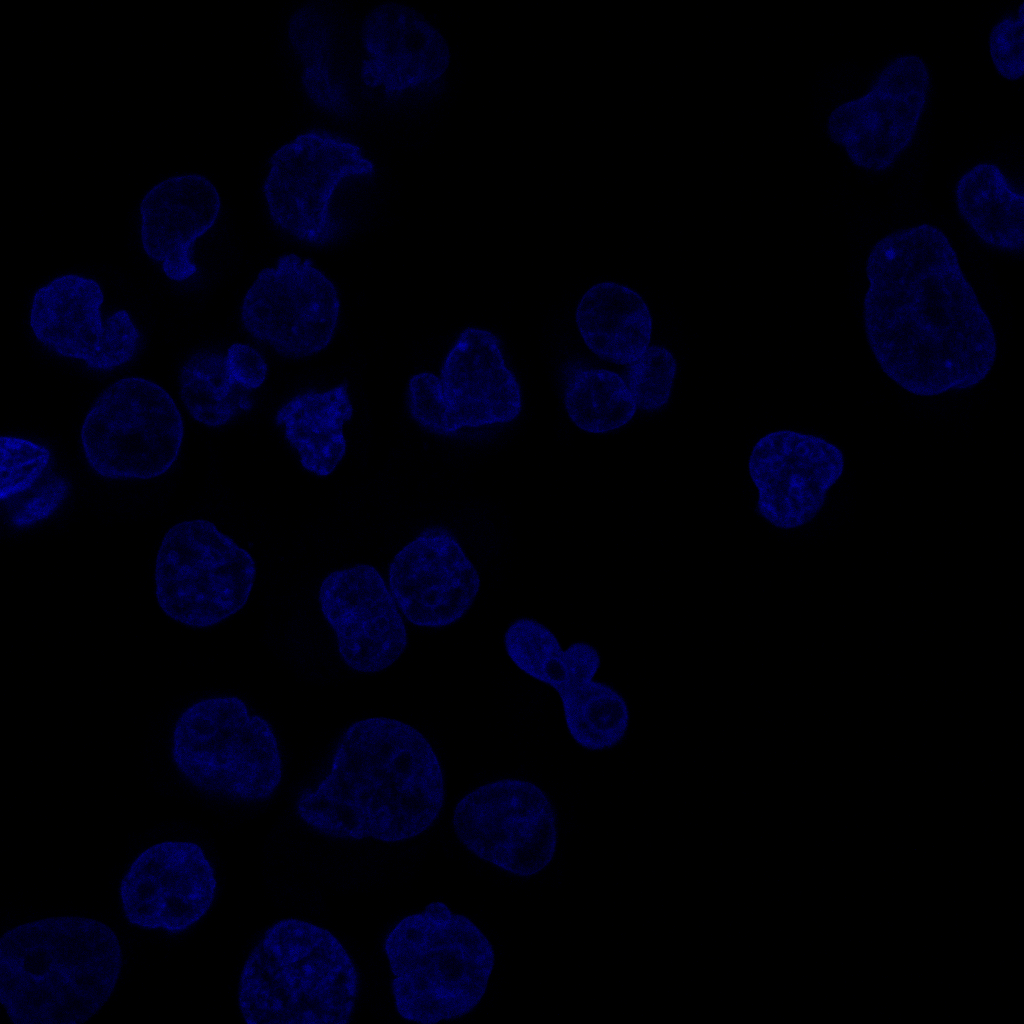

Supplement: Supplementary file 9 — Source Data for Figure 2 [file EMMM-15-e17313-s002.zip › Figure 2/C/Images/DMS114-g-Image Export-21_c2.tif]

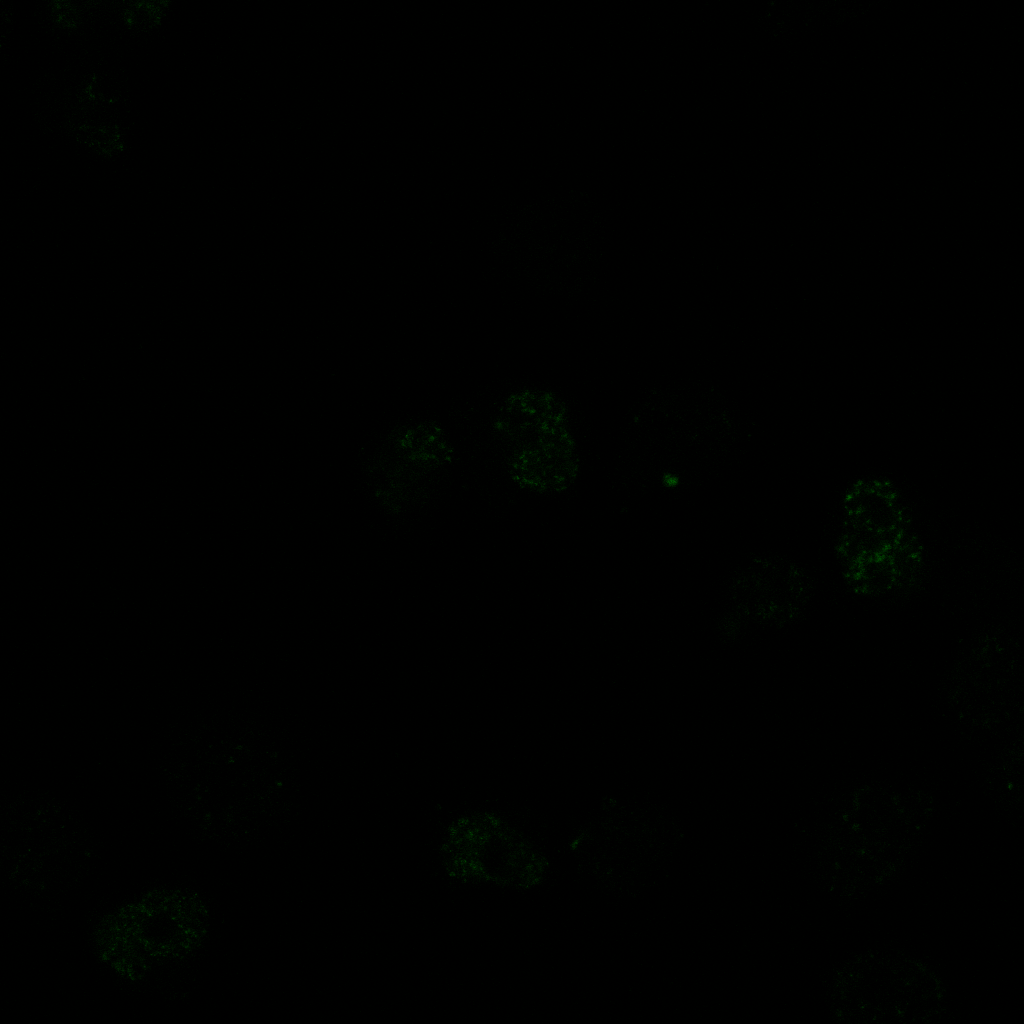

Supplement: Supplementary file 9 — Source Data for Figure 2 [file EMMM-15-e17313-s002.zip › Figure 2/C/Images/DMS114-Lurb-a-Image Export-22_c1.tif]

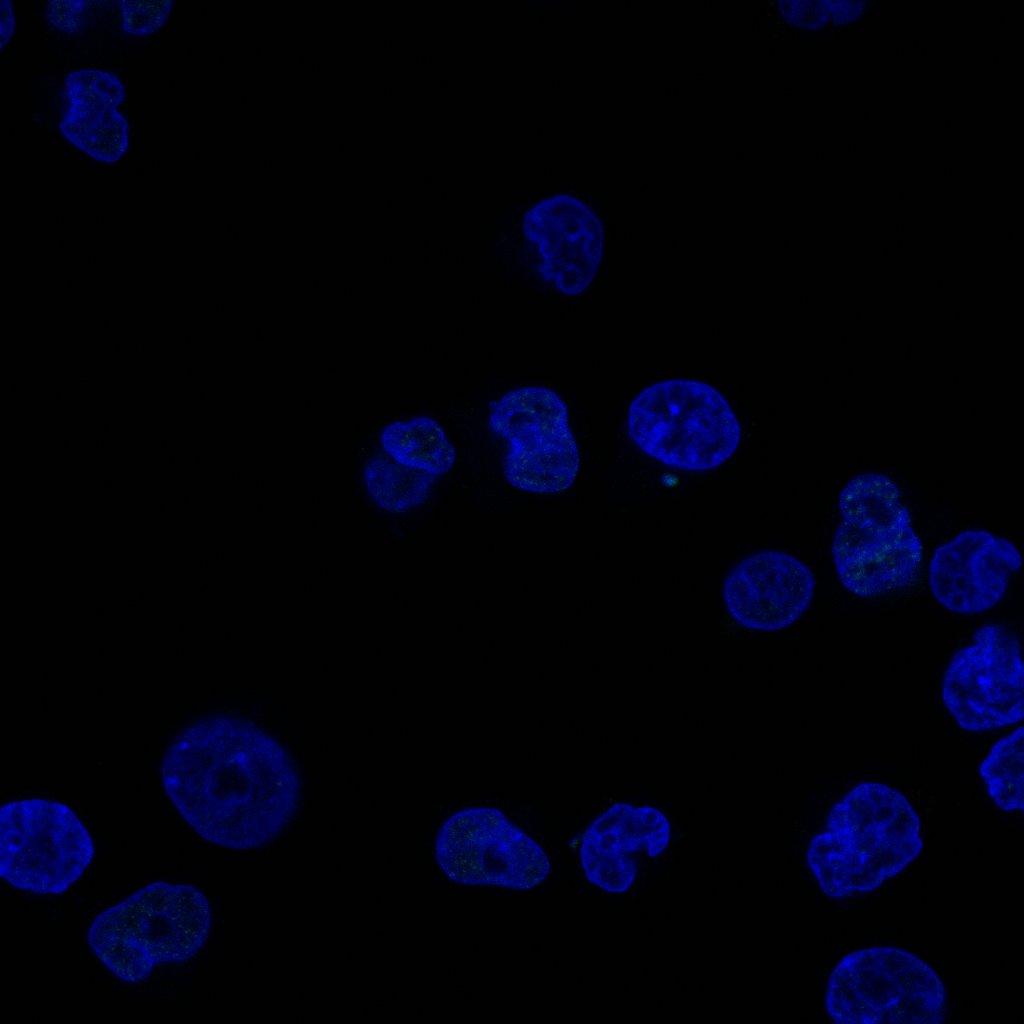

Supplement: Supplementary file 9 — Source Data for Figure 2 [file EMMM-15-e17313-s002.zip › Figure 2/C/Images/DMS114-Lurb-a-Image Export-22_c1+2.tif]

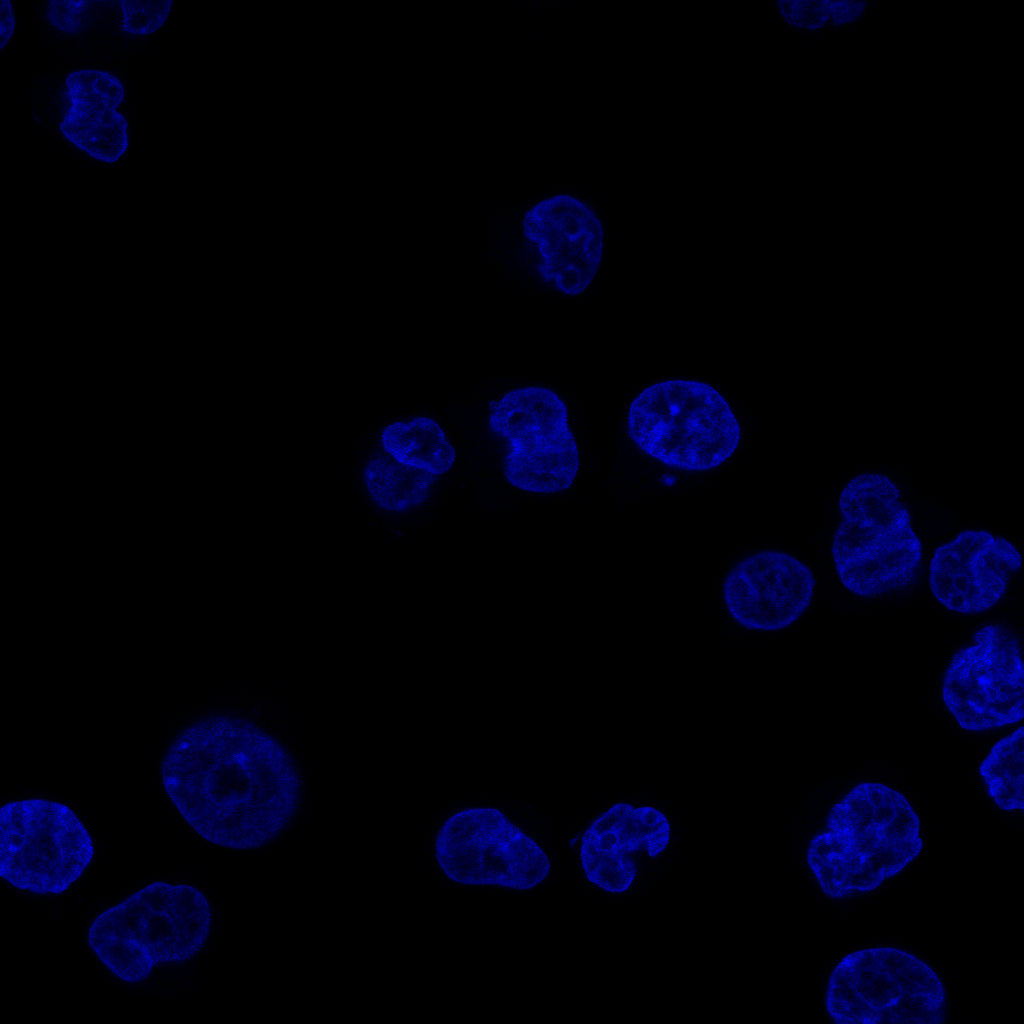

Supplement: Supplementary file 9 — Source Data for Figure 2 [file EMMM-15-e17313-s002.zip › Figure 2/C/Images/DMS114-Lurb-a-Image Export-22_c2.tif]

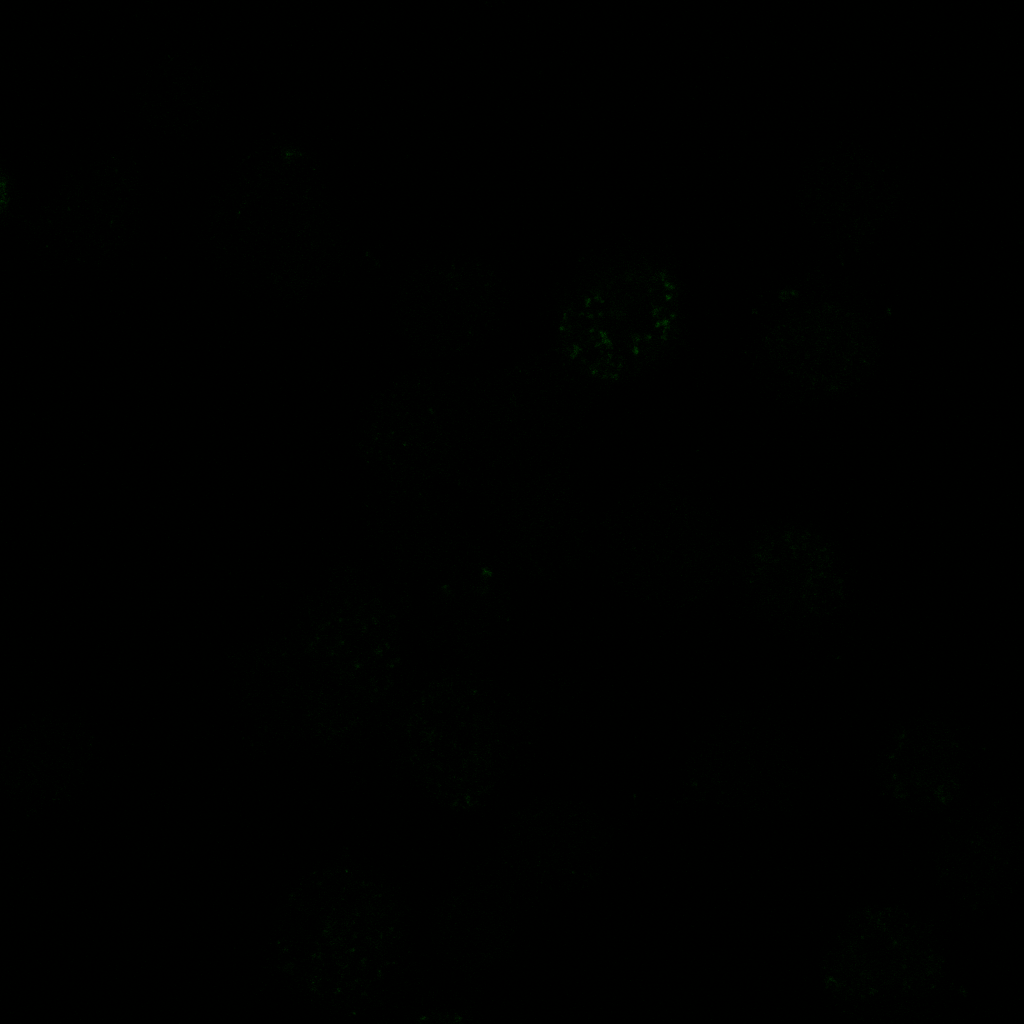

Supplement: Supplementary file 9 — Source Data for Figure 2 [file EMMM-15-e17313-s002.zip › Figure 2/C/Images/DMS114-Lurb-b-Image Export-23_c1.tif]

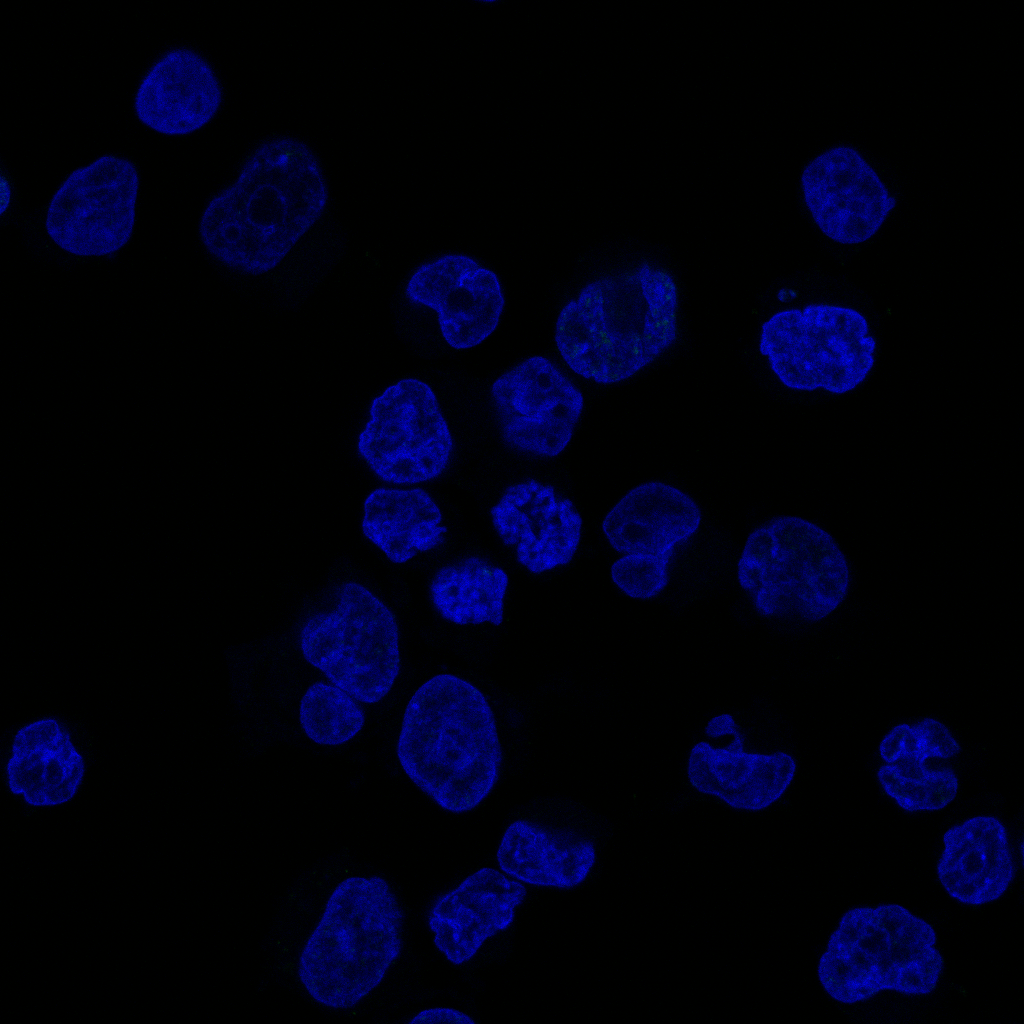

Supplement: Supplementary file 9 — Source Data for Figure 2 [file EMMM-15-e17313-s002.zip › Figure 2/C/Images/DMS114-Lurb-b-Image Export-23_c1+2.tif]

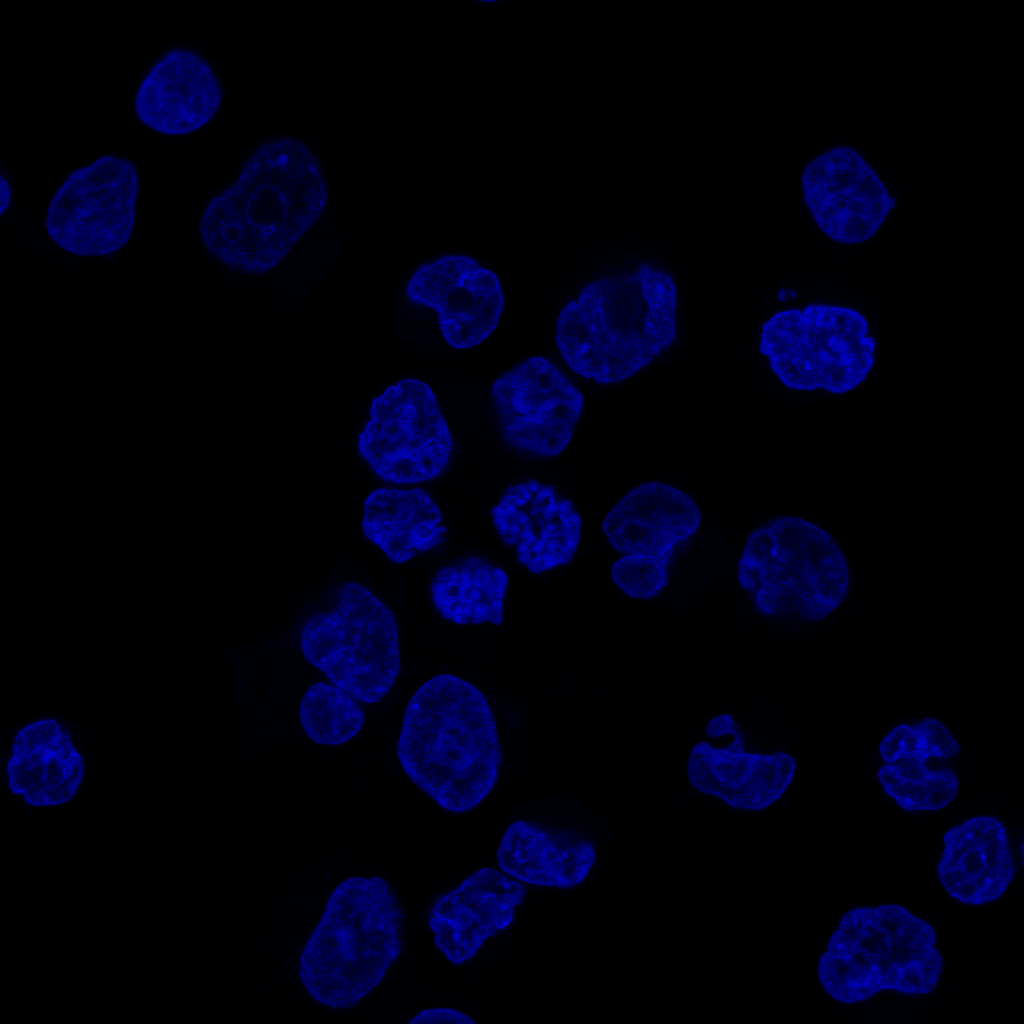

Supplement: Supplementary file 9 — Source Data for Figure 2 [file EMMM-15-e17313-s002.zip › Figure 2/C/Images/DMS114-Lurb-b-Image Export-23_c2.tif]

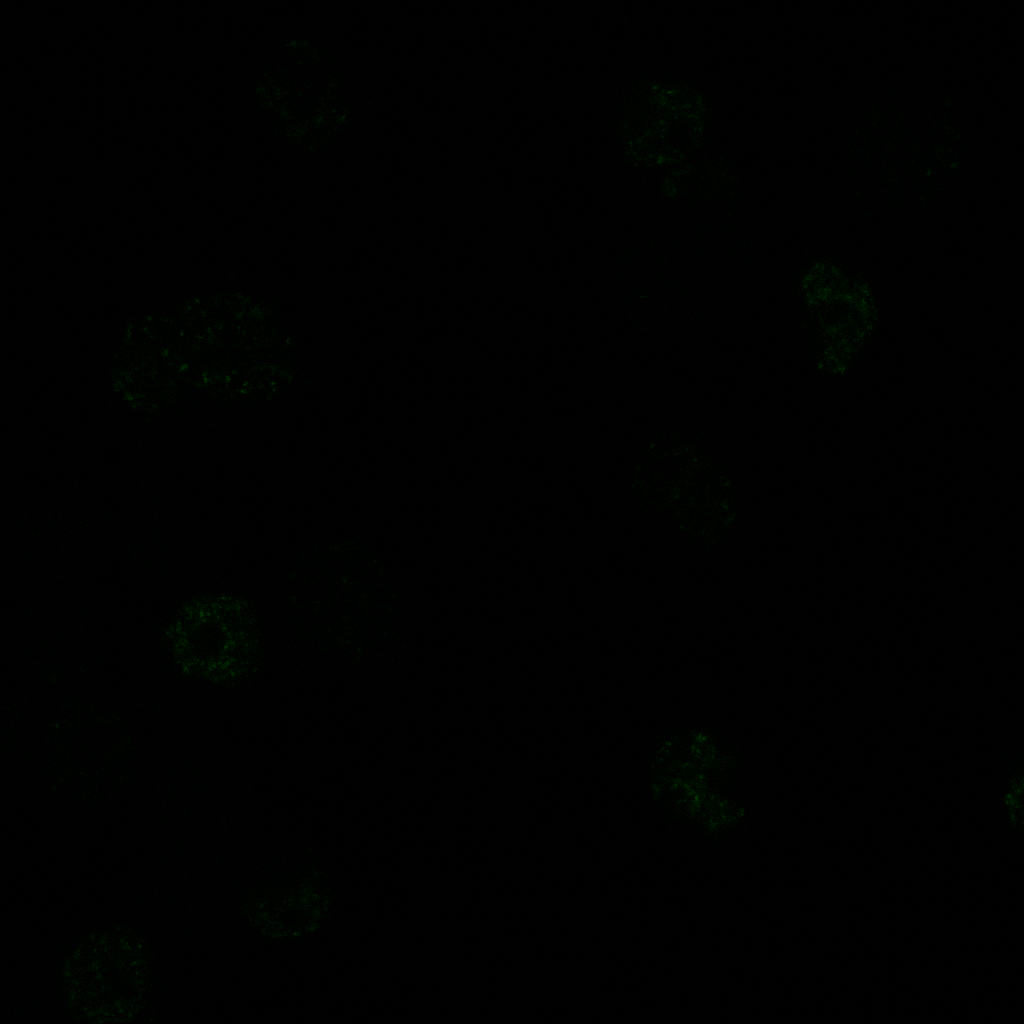

Supplement: Supplementary file 9 — Source Data for Figure 2 [file EMMM-15-e17313-s002.zip › Figure 2/C/Images/DMS114-Lurb-c-Image Export-24_c1.tif]

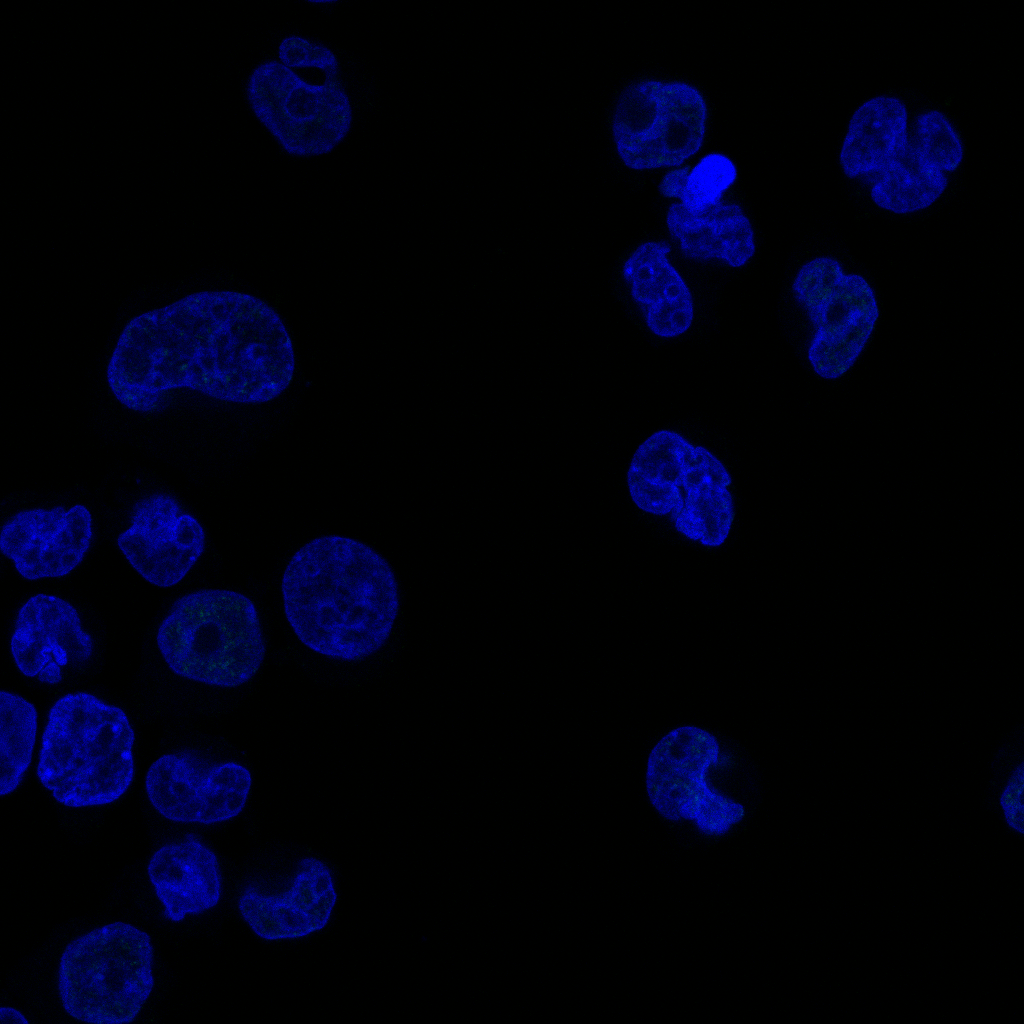

Supplement: Supplementary file 9 — Source Data for Figure 2 [file EMMM-15-e17313-s002.zip › Figure 2/C/Images/DMS114-Lurb-c-Image Export-24_c1+2.tif]

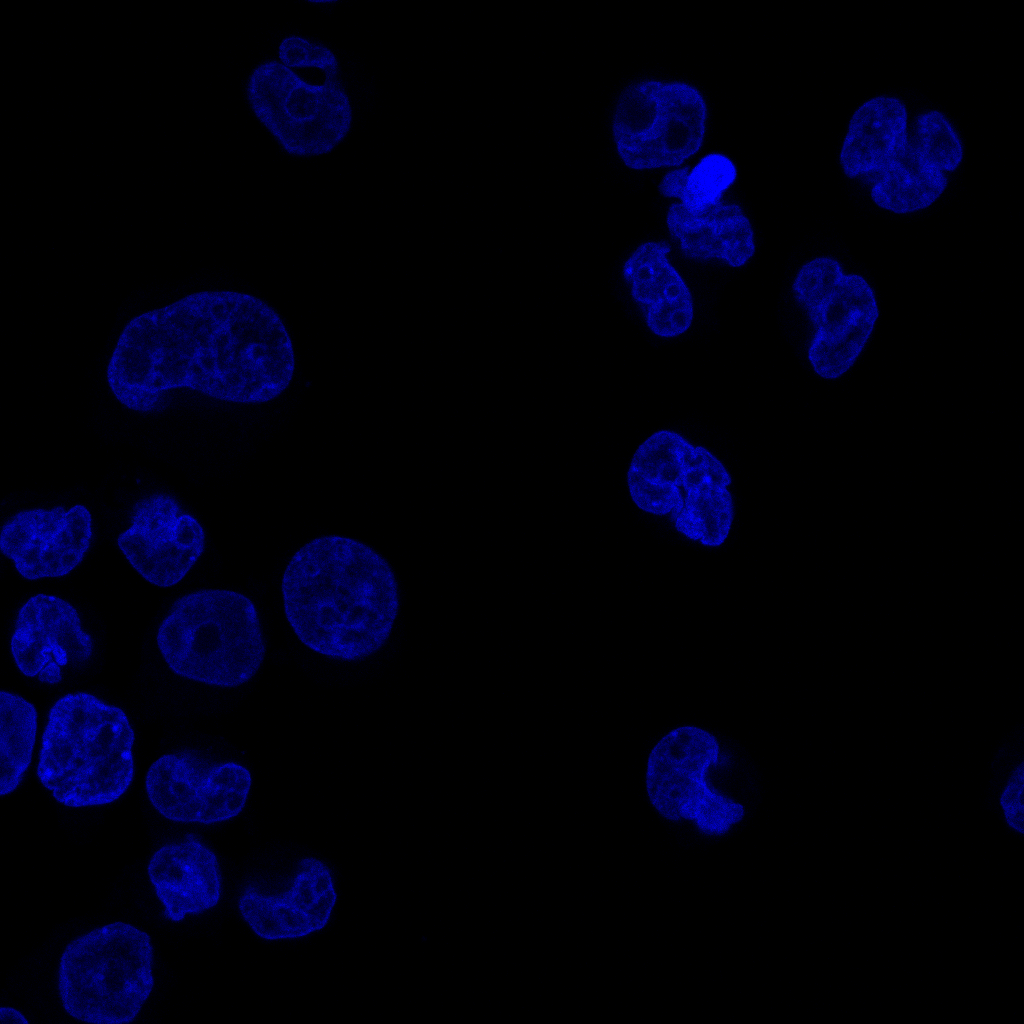

Supplement: Supplementary file 9 — Source Data for Figure 2 [file EMMM-15-e17313-s002.zip › Figure 2/C/Images/DMS114-Lurb-c-Image Export-24_c2.tif]

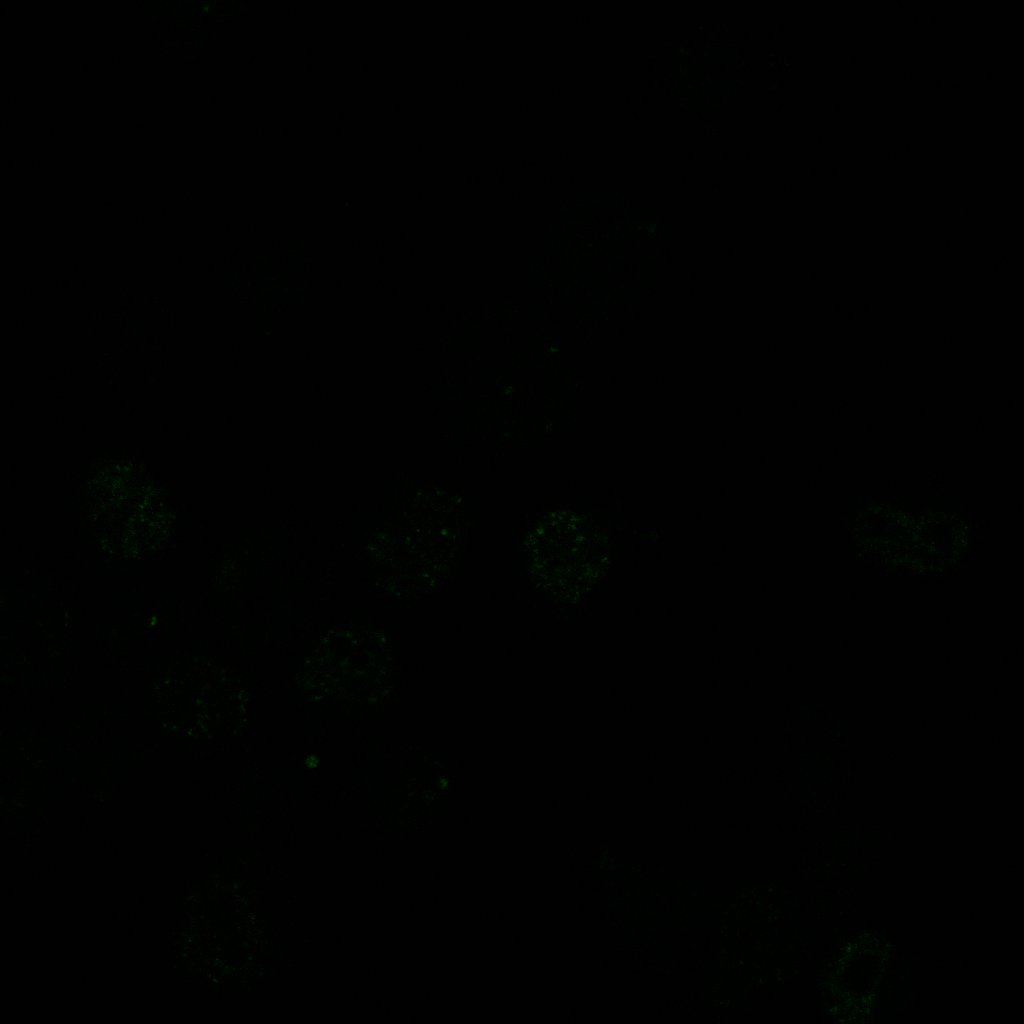

Supplement: Supplementary file 9 — Source Data for Figure 2 [file EMMM-15-e17313-s002.zip › Figure 2/C/Images/DMS114-Lurb-d-Image Export-25_c1.tif]

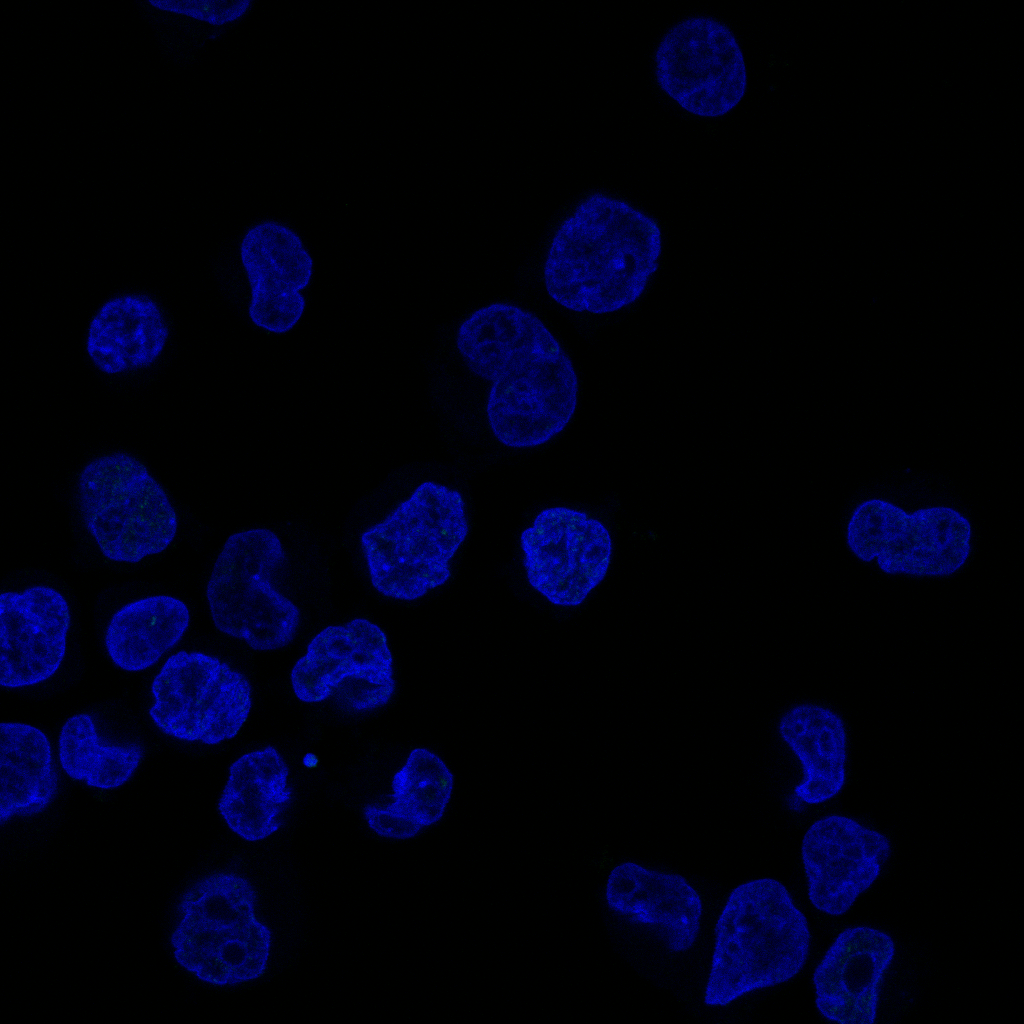

Supplement: Supplementary file 9 — Source Data for Figure 2 [file EMMM-15-e17313-s002.zip › Figure 2/C/Images/DMS114-Lurb-d-Image Export-25_c1+2.tif]

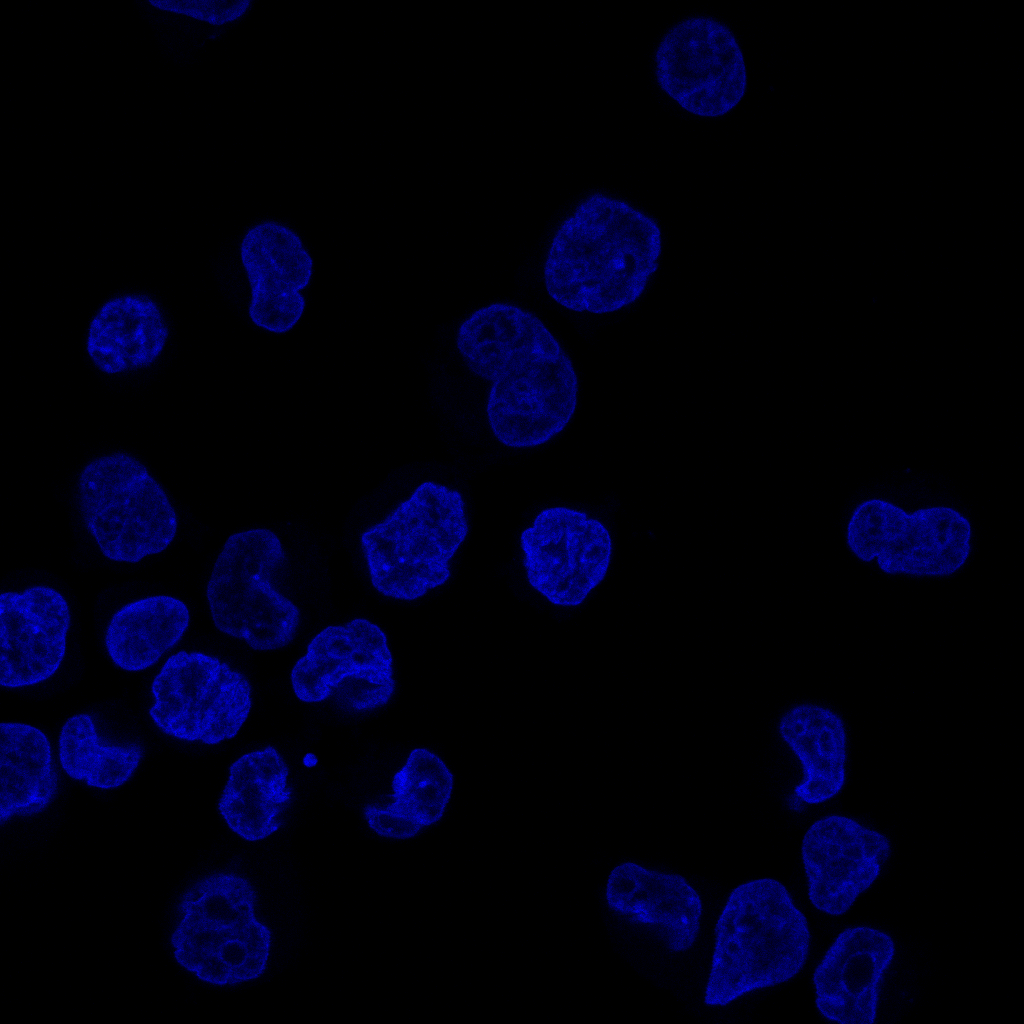

Supplement: Supplementary file 9 — Source Data for Figure 2 [file EMMM-15-e17313-s002.zip › Figure 2/C/Images/DMS114-Lurb-d-Image Export-25_c2.tif]

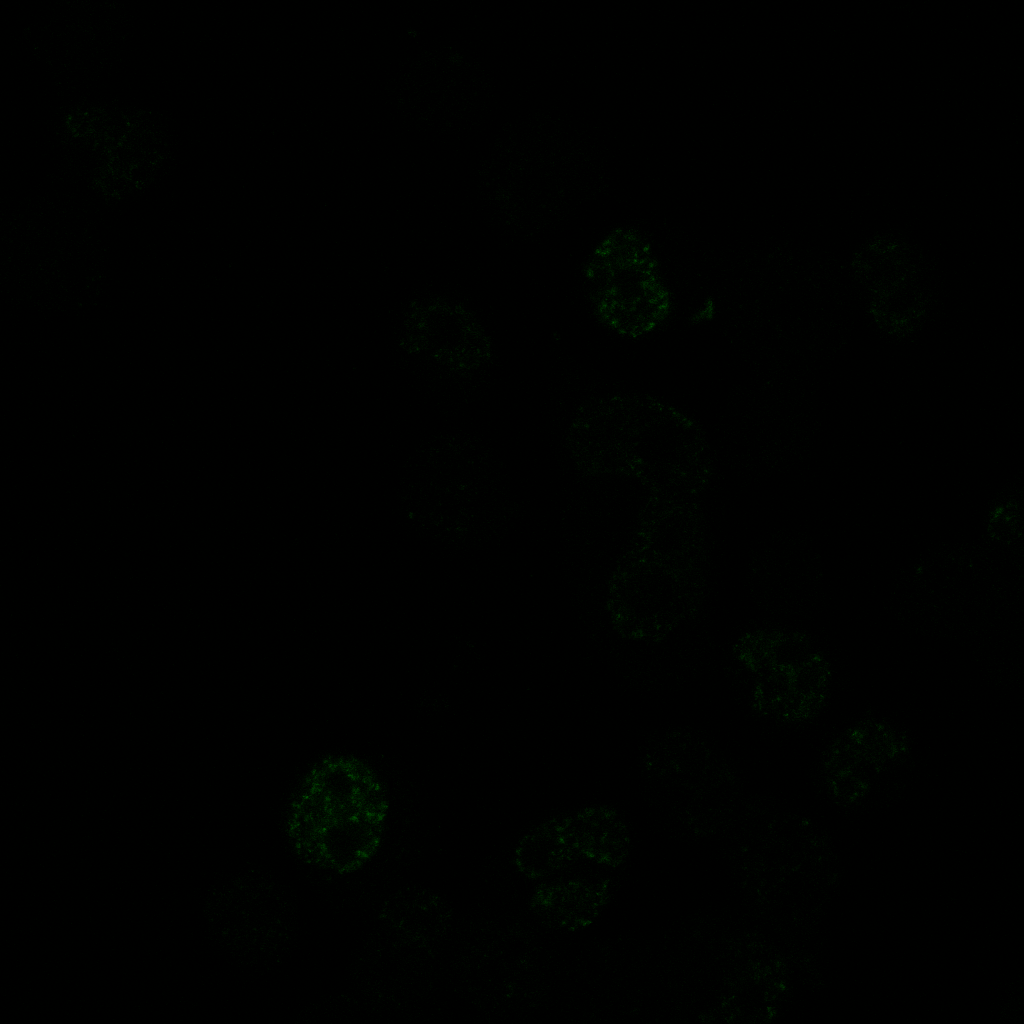

Supplement: Supplementary file 9 — Source Data for Figure 2 [file EMMM-15-e17313-s002.zip › Figure 2/C/Images/DMS114-Lurb-e-Image Export-26_c1.tif]

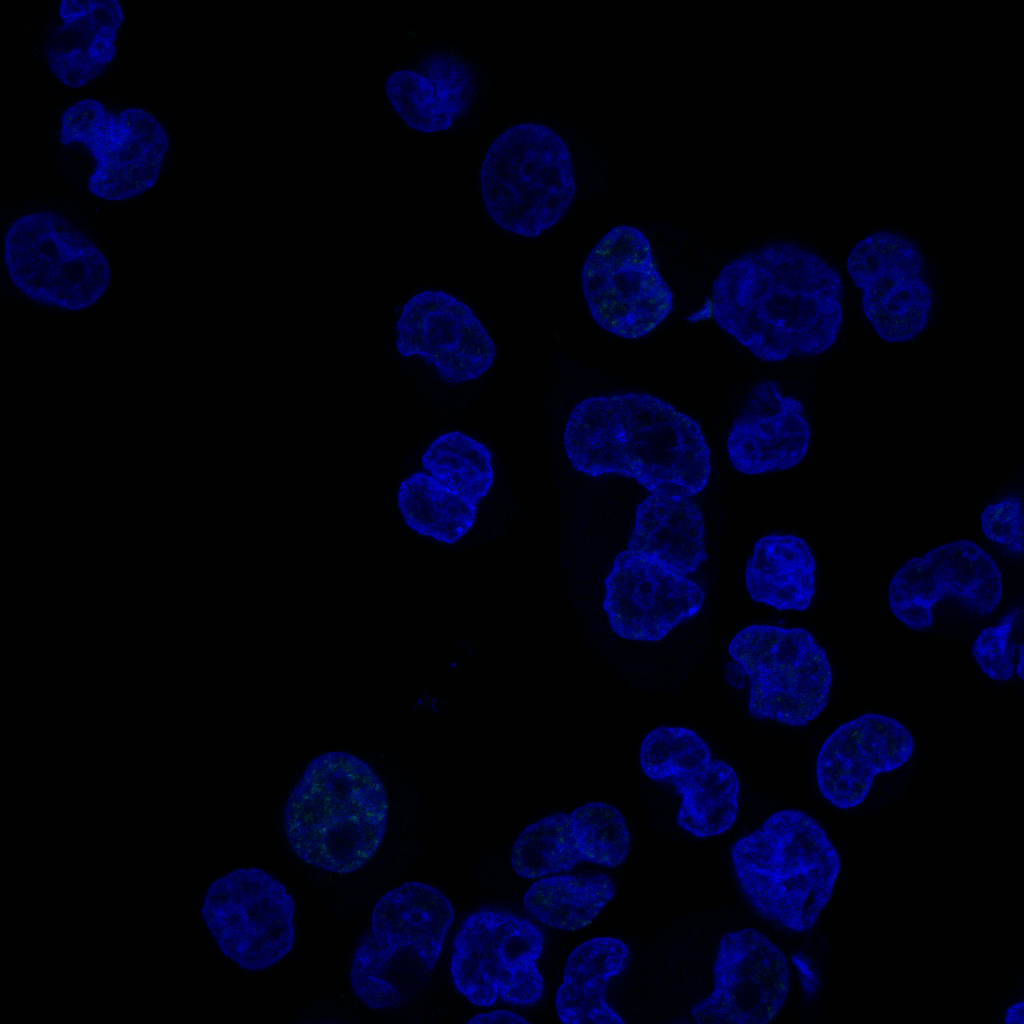

Supplement: Supplementary file 9 — Source Data for Figure 2 [file EMMM-15-e17313-s002.zip › Figure 2/C/Images/DMS114-Lurb-e-Image Export-26_c1+2.tif]

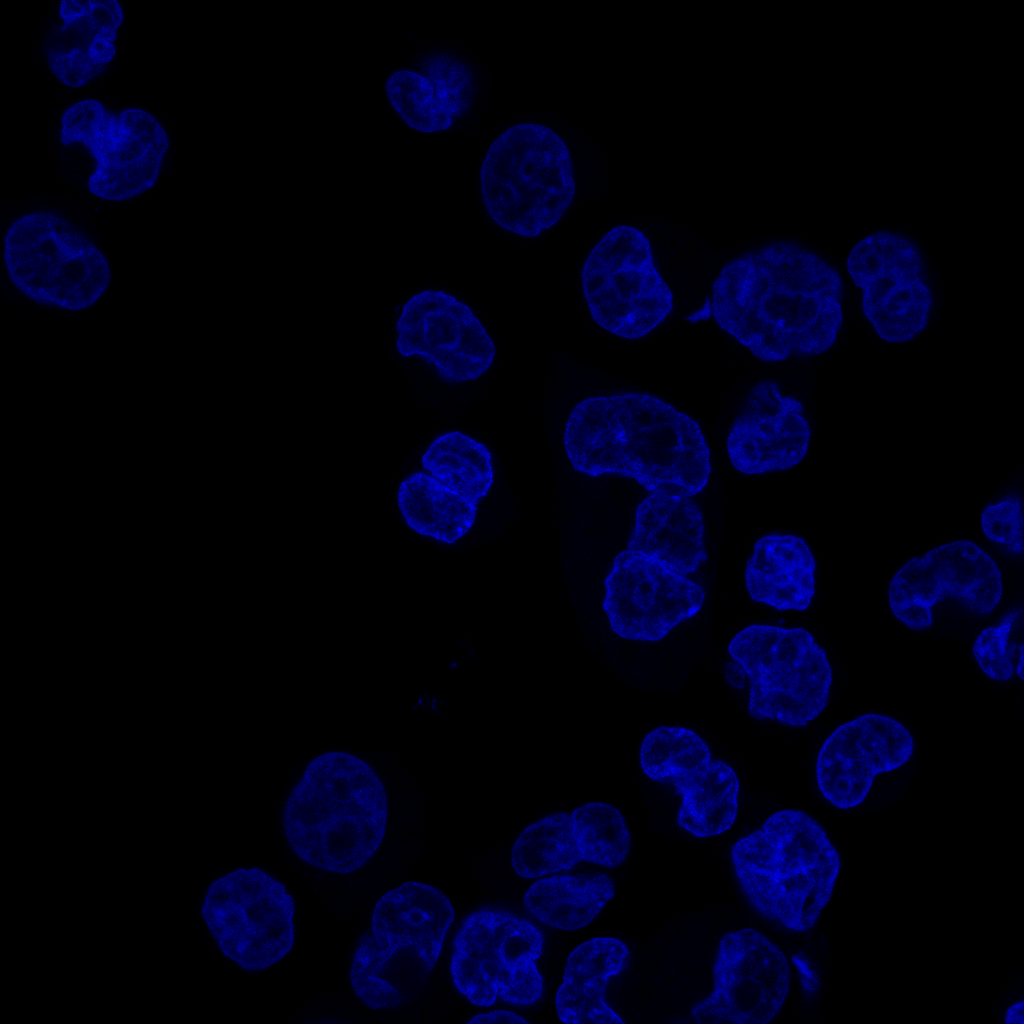

Supplement: Supplementary file 9 — Source Data for Figure 2 [file EMMM-15-e17313-s002.zip › Figure 2/C/Images/DMS114-Lurb-e-Image Export-26_c2.tif]

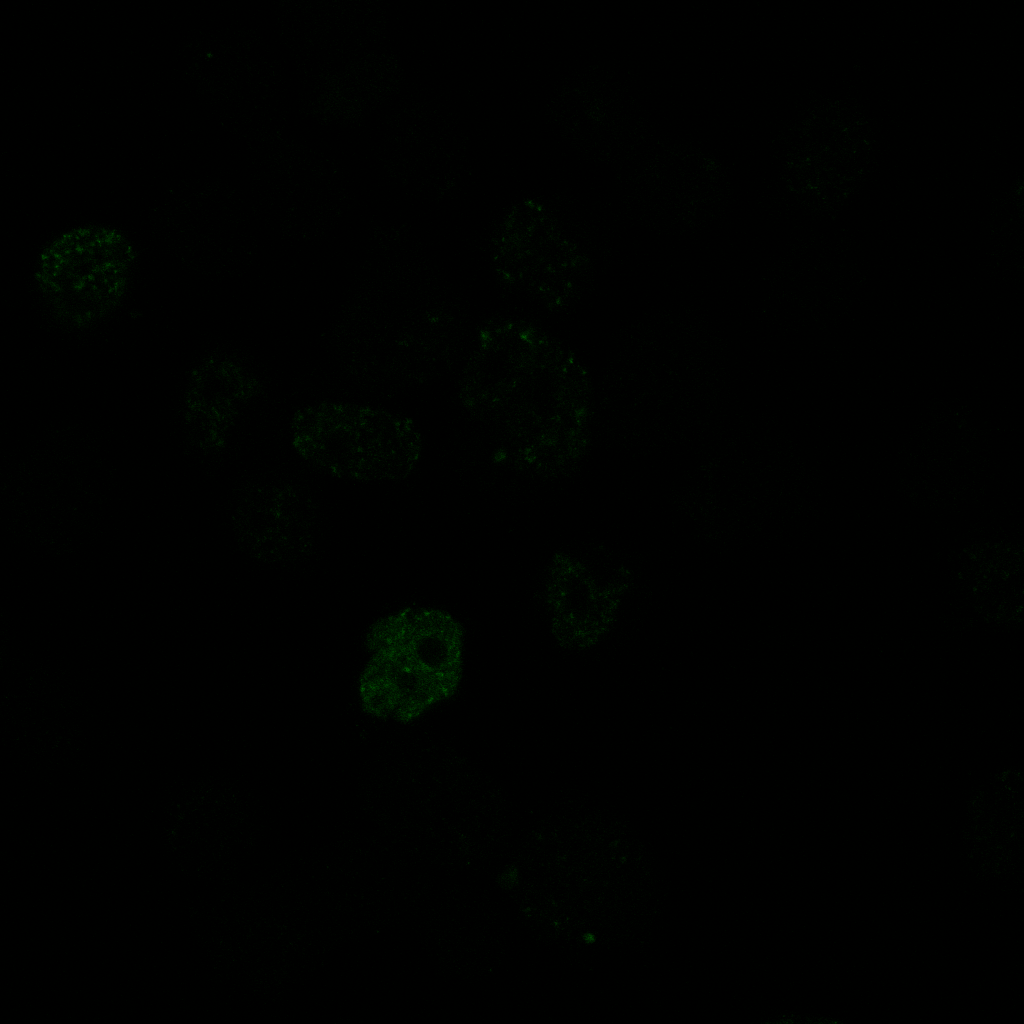

Supplement: Supplementary file 9 — Source Data for Figure 2 [file EMMM-15-e17313-s002.zip › Figure 2/C/Images/DMS114-Lurb-f-Image Export-27_c1.tif]

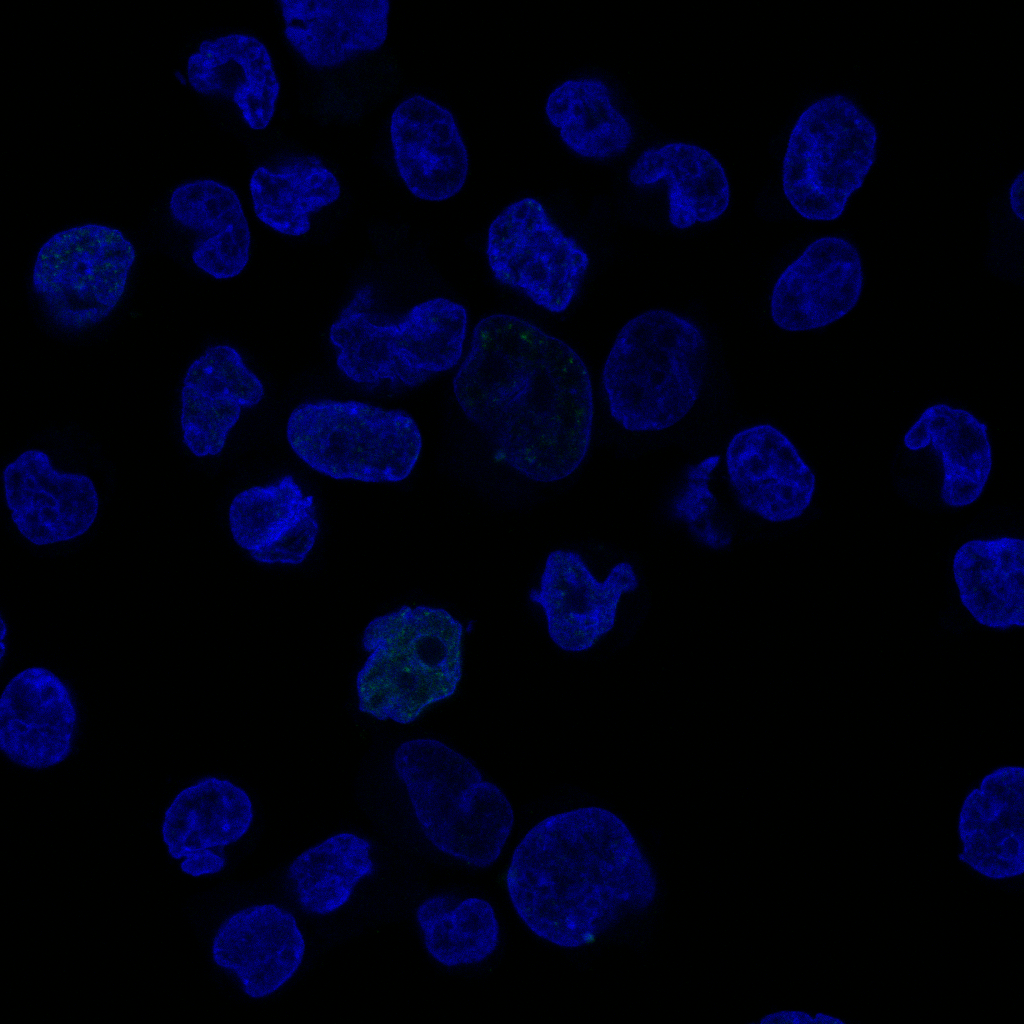

Supplement: Supplementary file 9 — Source Data for Figure 2 [file EMMM-15-e17313-s002.zip › Figure 2/C/Images/DMS114-Lurb-f-Image Export-27_c1+2.tif]

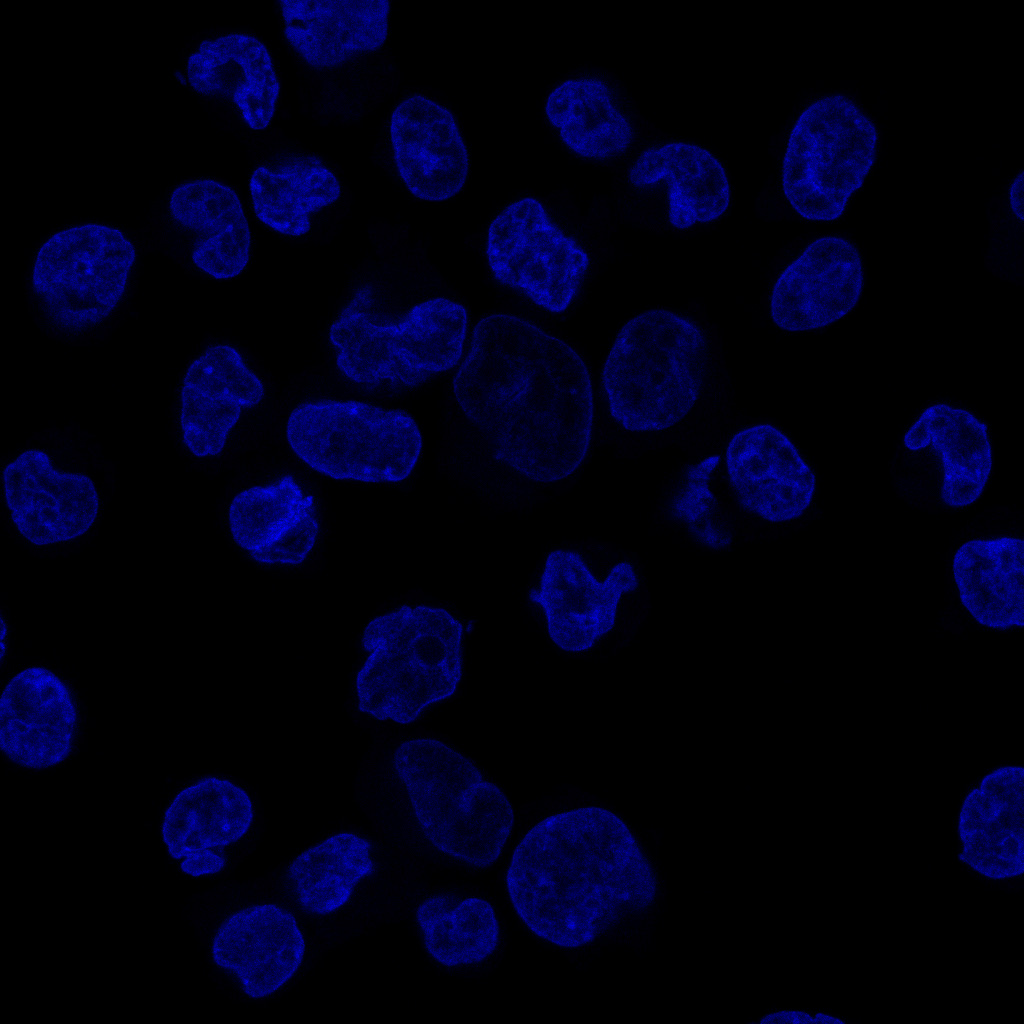

Supplement: Supplementary file 9 — Source Data for Figure 2 [file EMMM-15-e17313-s002.zip › Figure 2/C/Images/DMS114-Lurb-f-Image Export-27_c2.tif]

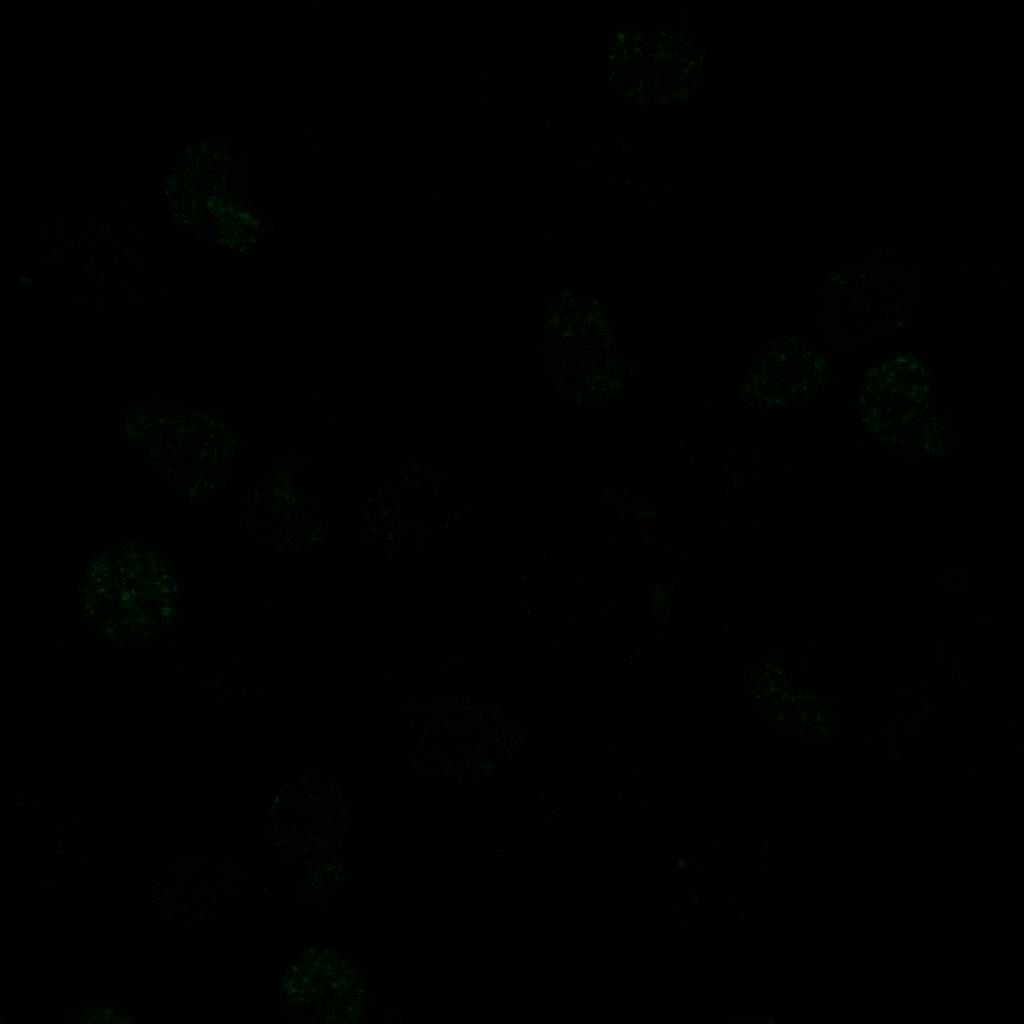

Supplement: Supplementary file 9 — Source Data for Figure 2 [file EMMM-15-e17313-s002.zip › Figure 2/C/Images/DMS114-Lurb-g-Image Export-28_c1.tif]
