# Supplementary figures and images for: ATR inhibition augments the efficacy of lurbinectedin in small‐cell lung cancer (part 2 of 2)
Source: EMBO Mol Med. 2023 Jul 25;15(8):e17313. doi: 10.15252/emmm.202217313 (PMC10405061; doi:10.15252/emmm.202217313)

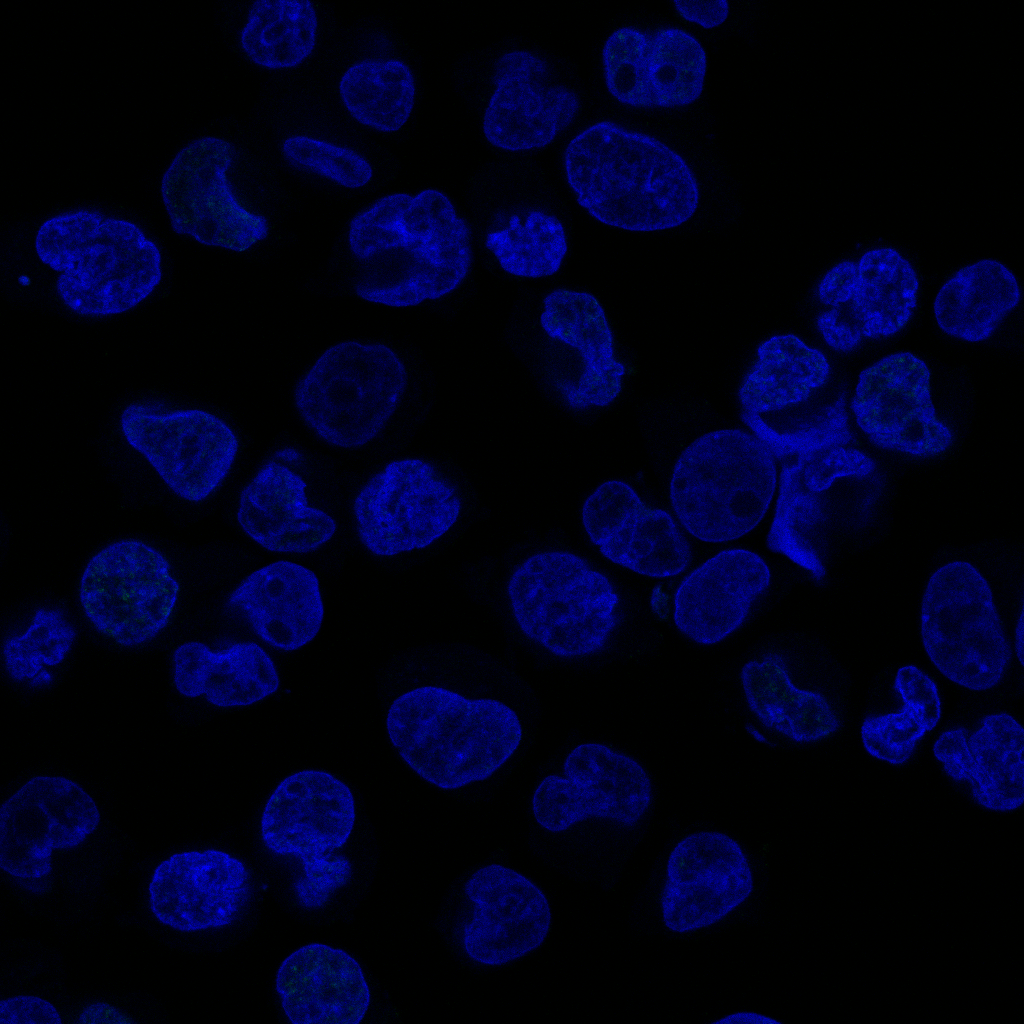

Supplement: Supplementary file 9 — Source Data for Figure 2 [file EMMM-15-e17313-s002.zip › Figure 2/C/Images/DMS114-Lurb-g-Image Export-28_c1+2.tif]

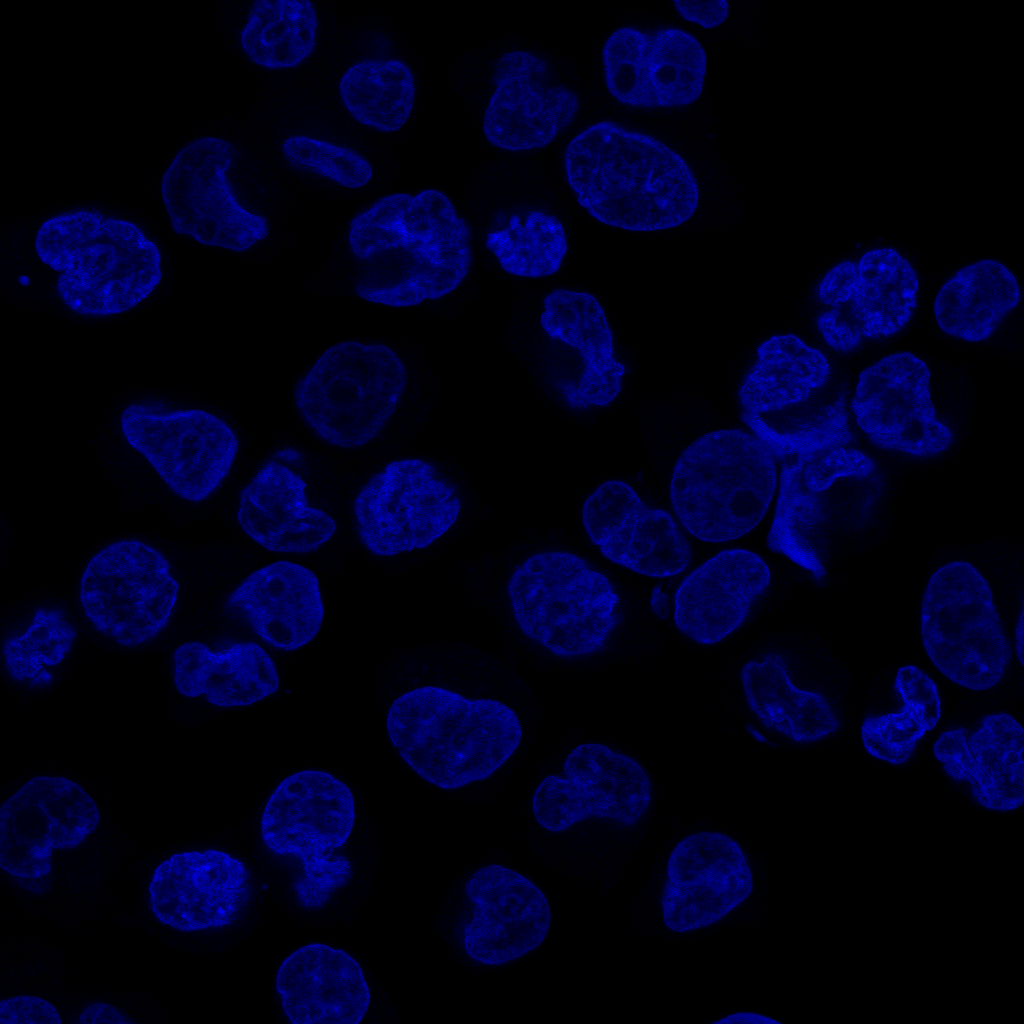

Supplement: Supplementary file 9 — Source Data for Figure 2 [file EMMM-15-e17313-s002.zip › Figure 2/C/Images/DMS114-Lurb-g-Image Export-28_c2.tif]

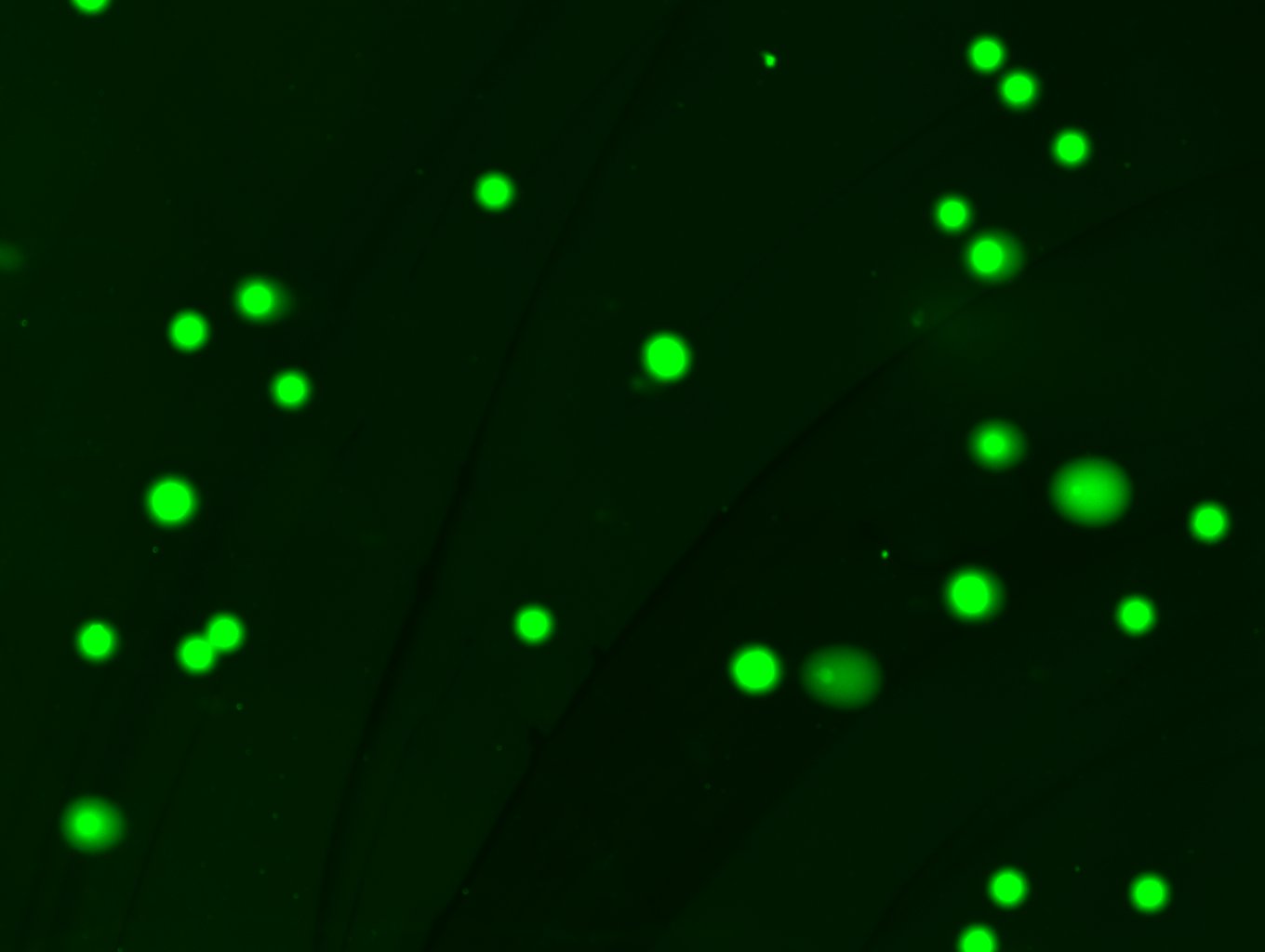

Supplement: Supplementary file 9 — Source Data for Figure 2 [file EMMM-15-e17313-s002.zip › Figure 2/D/Representative Images/Berzosertib9.jpg]

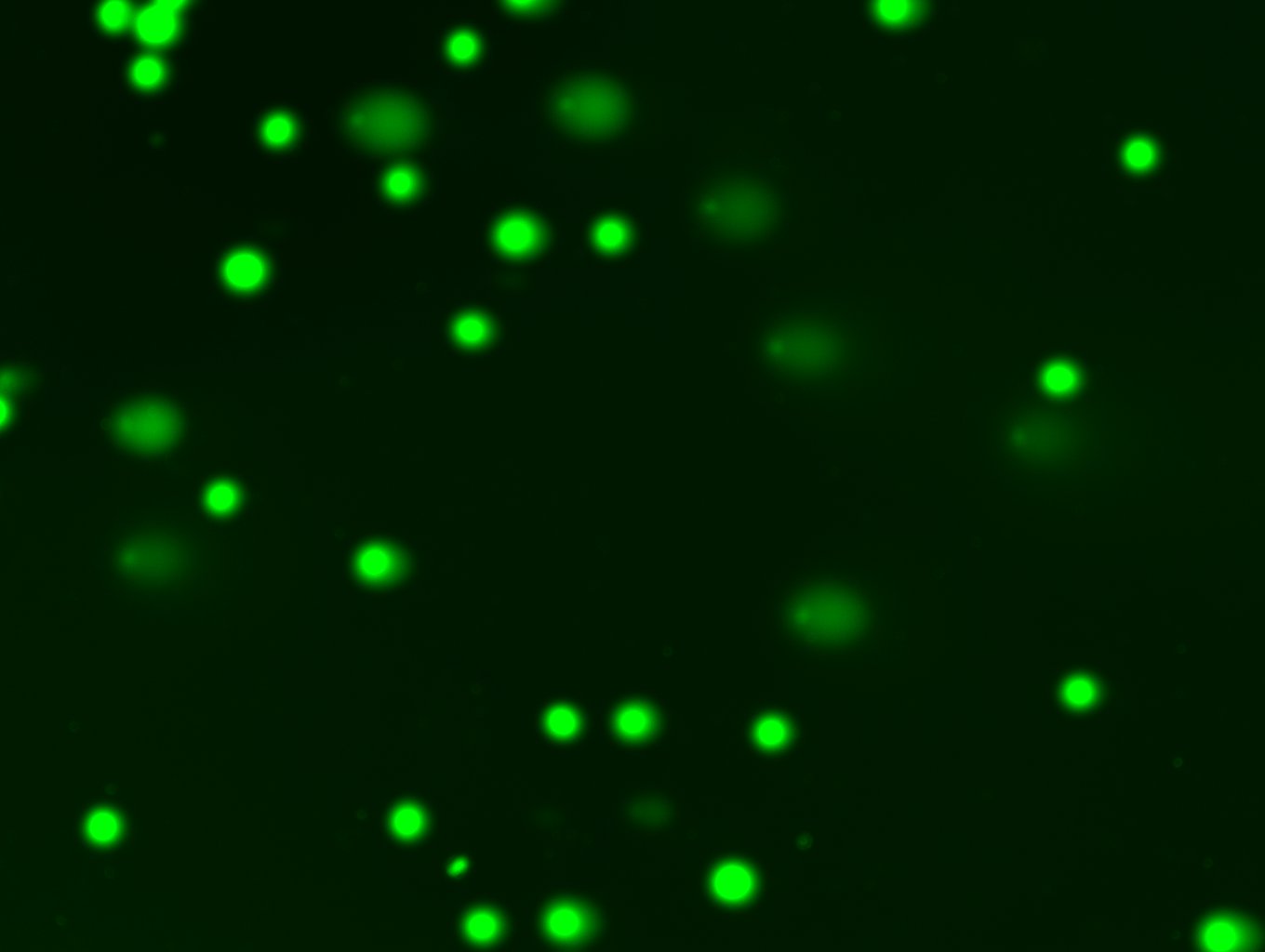

Supplement: Supplementary file 9 — Source Data for Figure 2 [file EMMM-15-e17313-s002.zip › Figure 2/D/Representative Images/combi7.jpg]

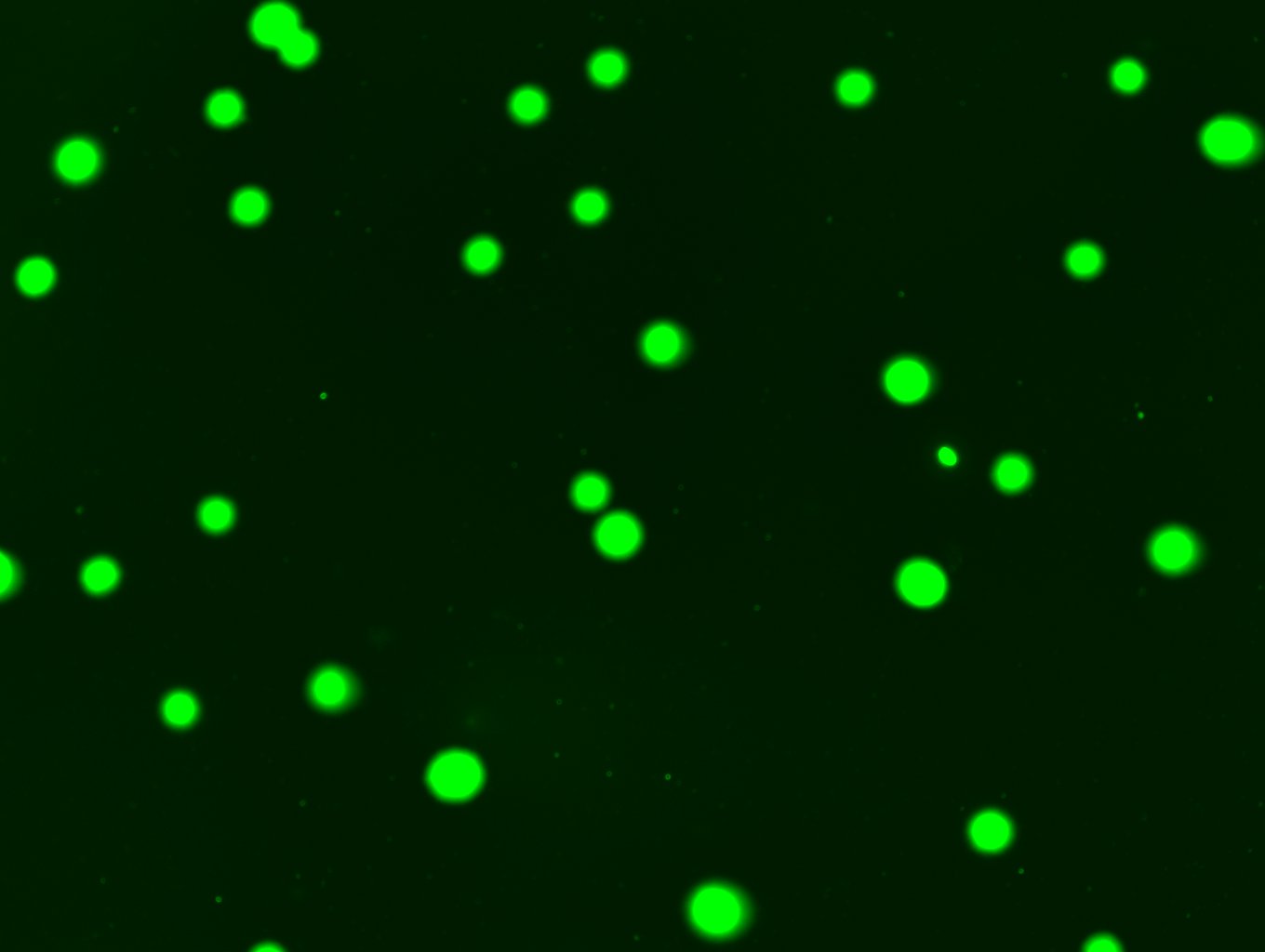

Supplement: Supplementary file 9 — Source Data for Figure 2 [file EMMM-15-e17313-s002.zip › Figure 2/D/Representative Images/Control2.jpg]

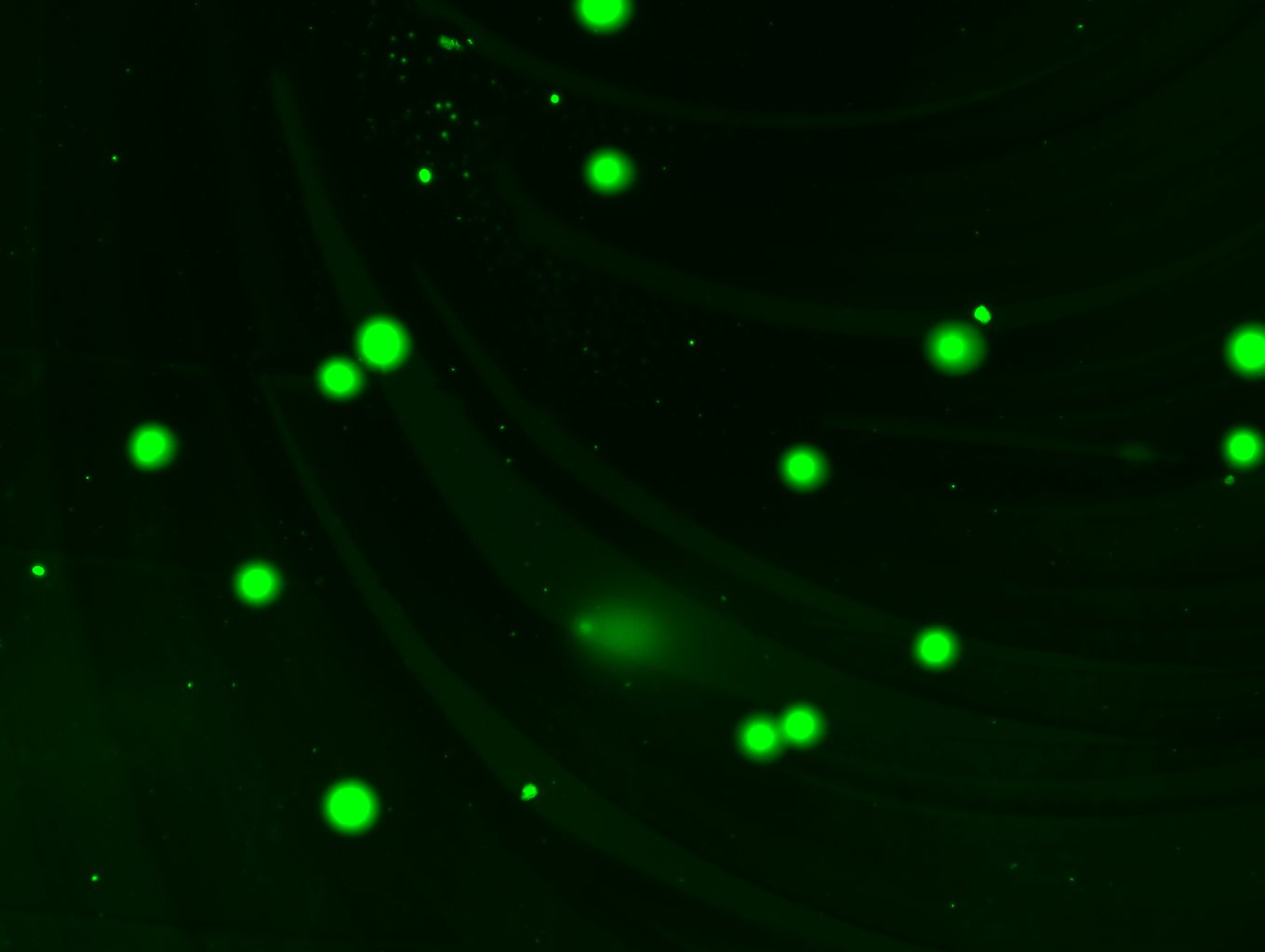

Supplement: Supplementary file 9 — Source Data for Figure 2 [file EMMM-15-e17313-s002.zip › Figure 2/D/Representative Images/Lurbinectedin9.jpg]

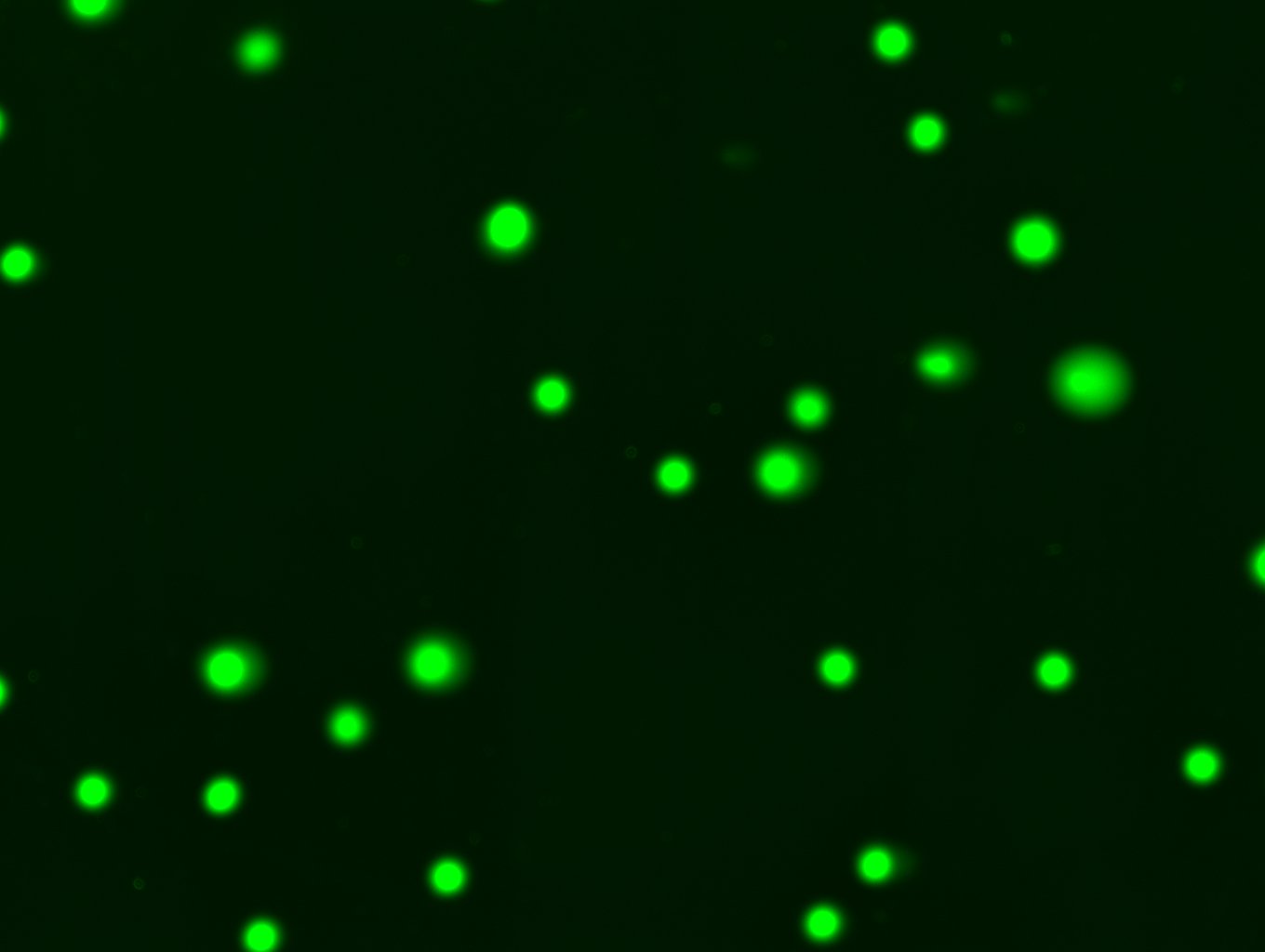

Supplement: Supplementary file 9 — Source Data for Figure 2 [file EMMM-15-e17313-s002.zip › Figure 2/E/Comet_Files_For_quantification/Berzosertib1.jpg]

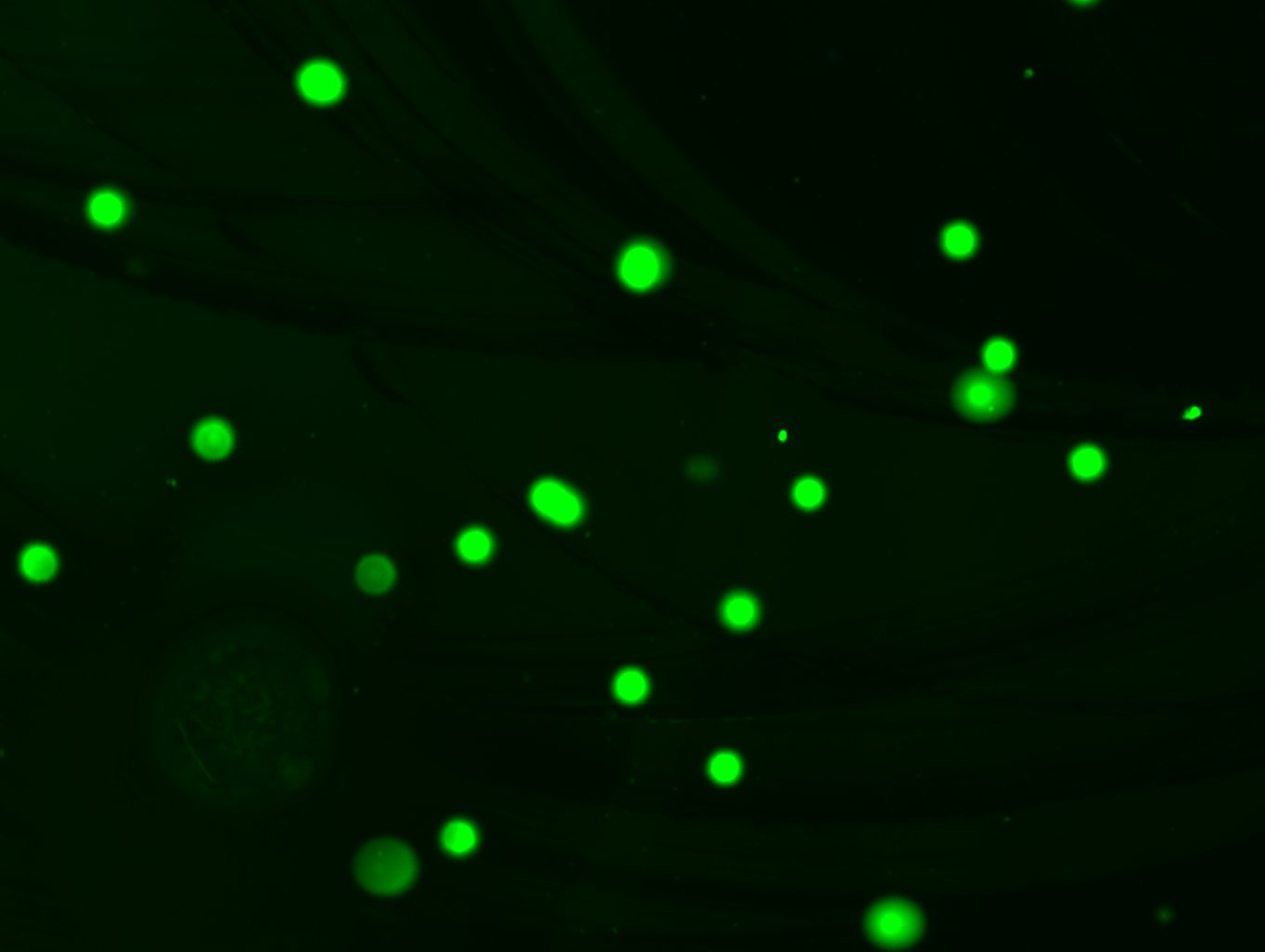

Supplement: Supplementary file 9 — Source Data for Figure 2 [file EMMM-15-e17313-s002.zip › Figure 2/E/Comet_Files_For_quantification/Berzosertib10.jpg]

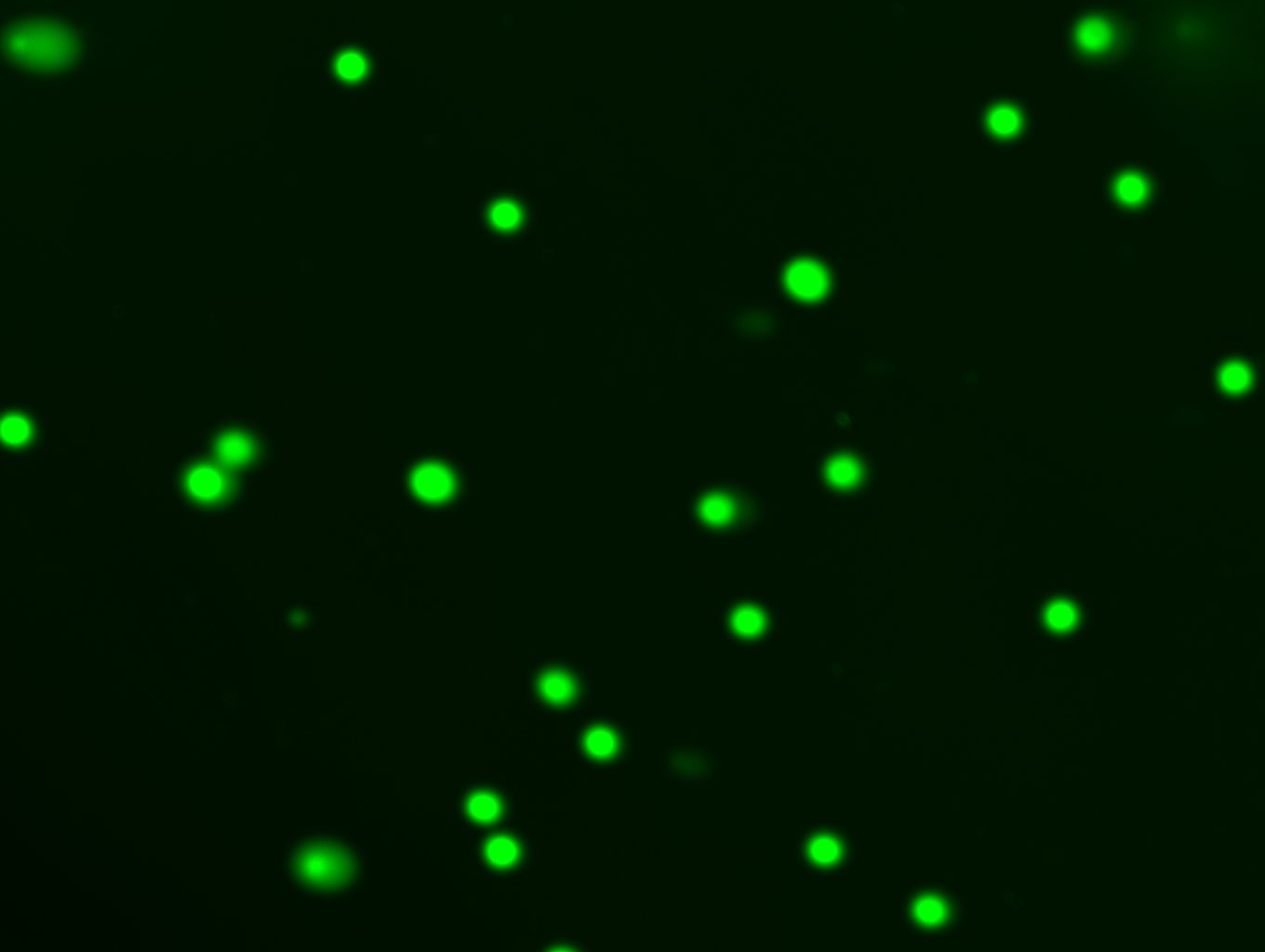

Supplement: Supplementary file 9 — Source Data for Figure 2 [file EMMM-15-e17313-s002.zip › Figure 2/E/Comet_Files_For_quantification/Berzosertib2.jpg]

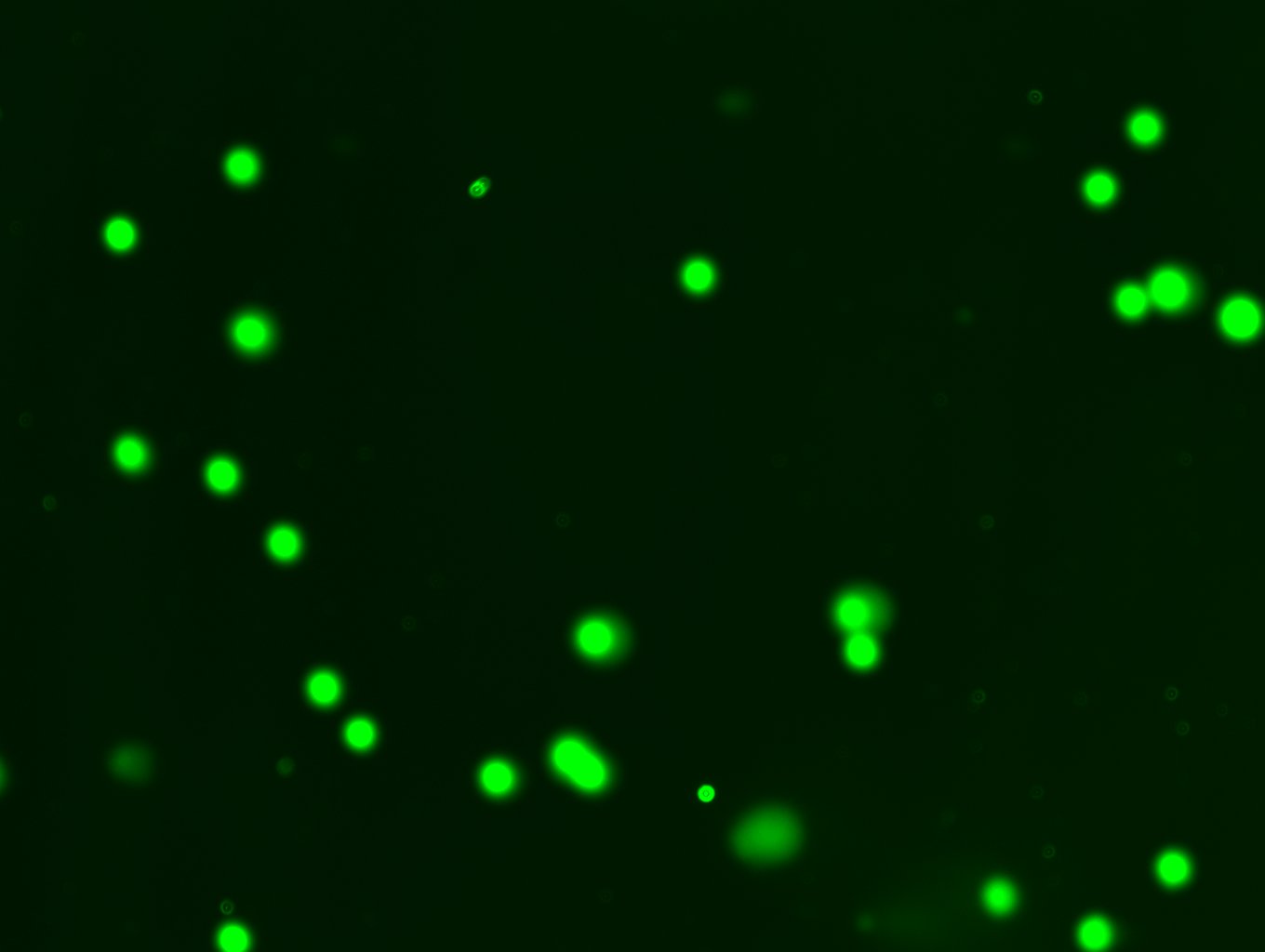

Supplement: Supplementary file 9 — Source Data for Figure 2 [file EMMM-15-e17313-s002.zip › Figure 2/E/Comet_Files_For_quantification/Berzosertib3.jpg]

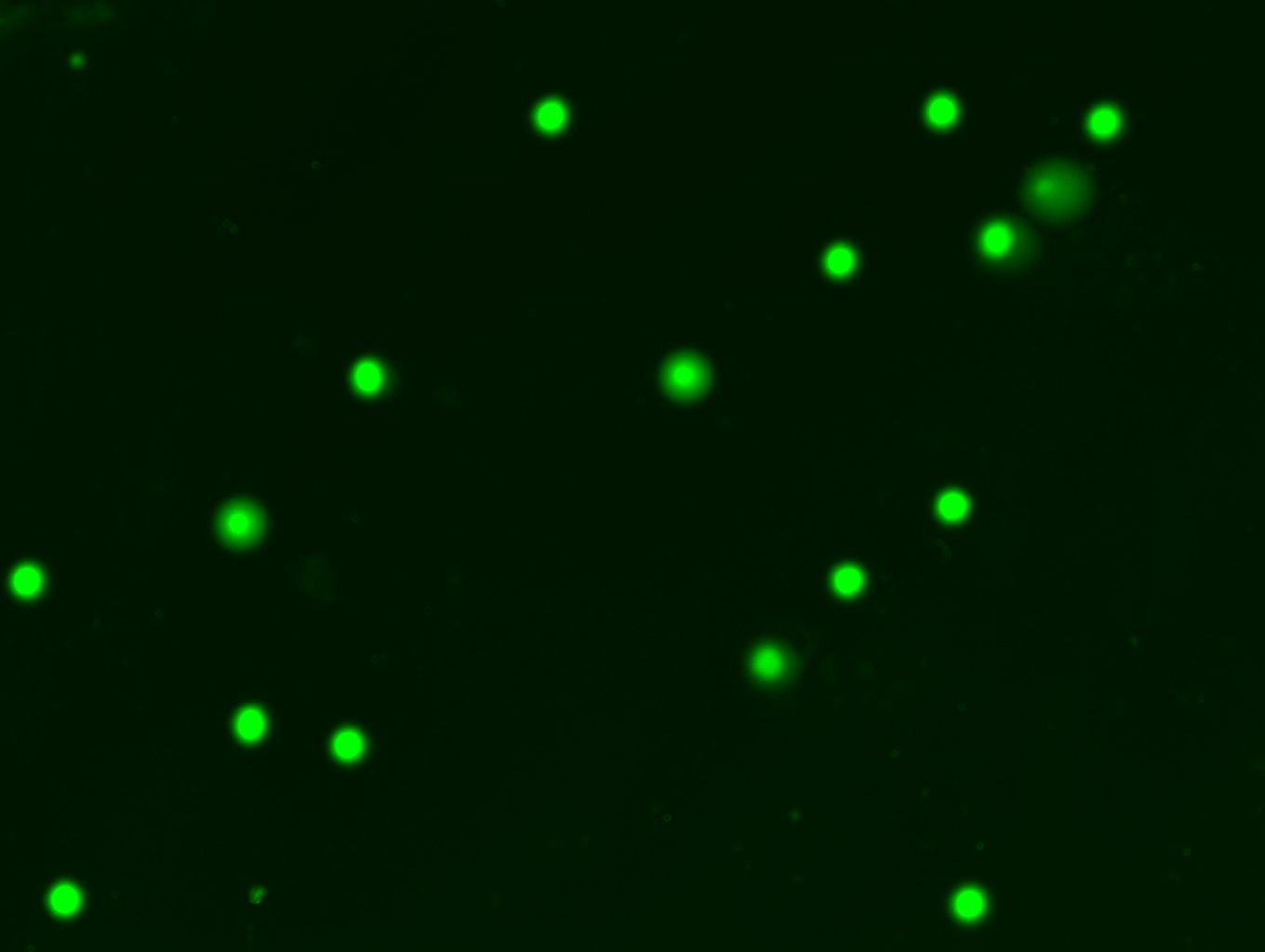

Supplement: Supplementary file 9 — Source Data for Figure 2 [file EMMM-15-e17313-s002.zip › Figure 2/E/Comet_Files_For_quantification/Berzosertib4.jpg]

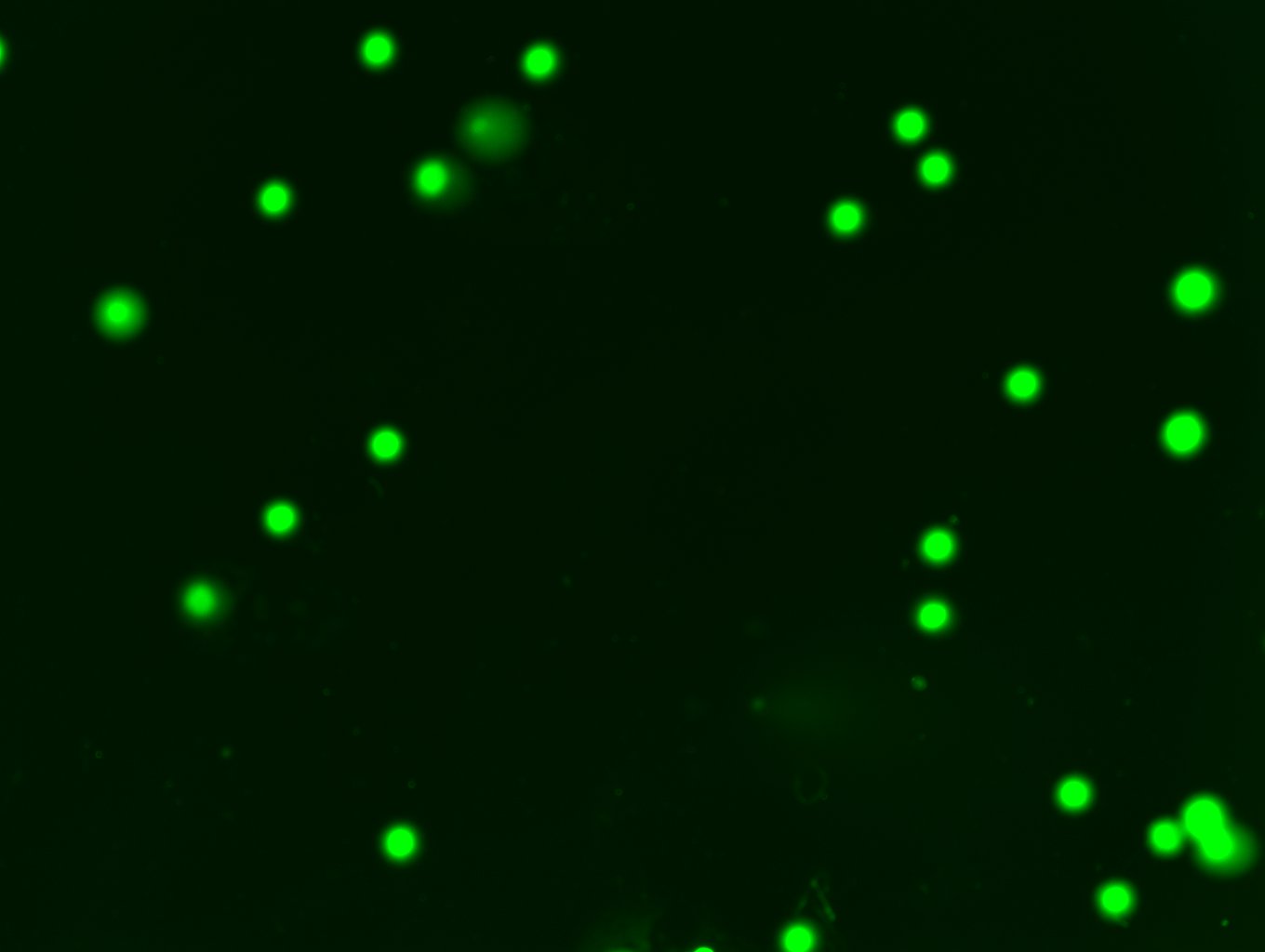

Supplement: Supplementary file 9 — Source Data for Figure 2 [file EMMM-15-e17313-s002.zip › Figure 2/E/Comet_Files_For_quantification/Berzosertib5.jpg]

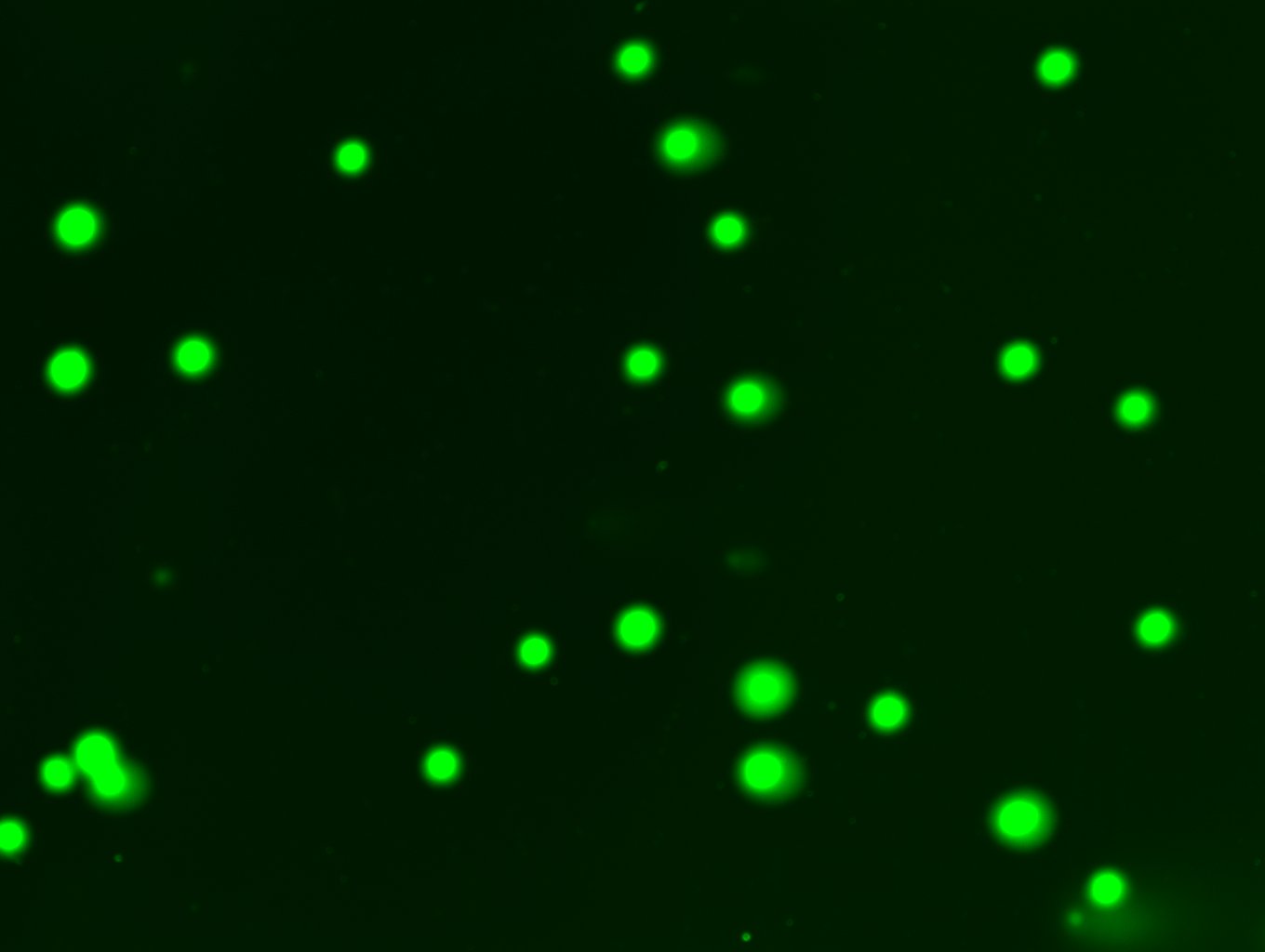

Supplement: Supplementary file 9 — Source Data for Figure 2 [file EMMM-15-e17313-s002.zip › Figure 2/E/Comet_Files_For_quantification/Berzosertib6.jpg]

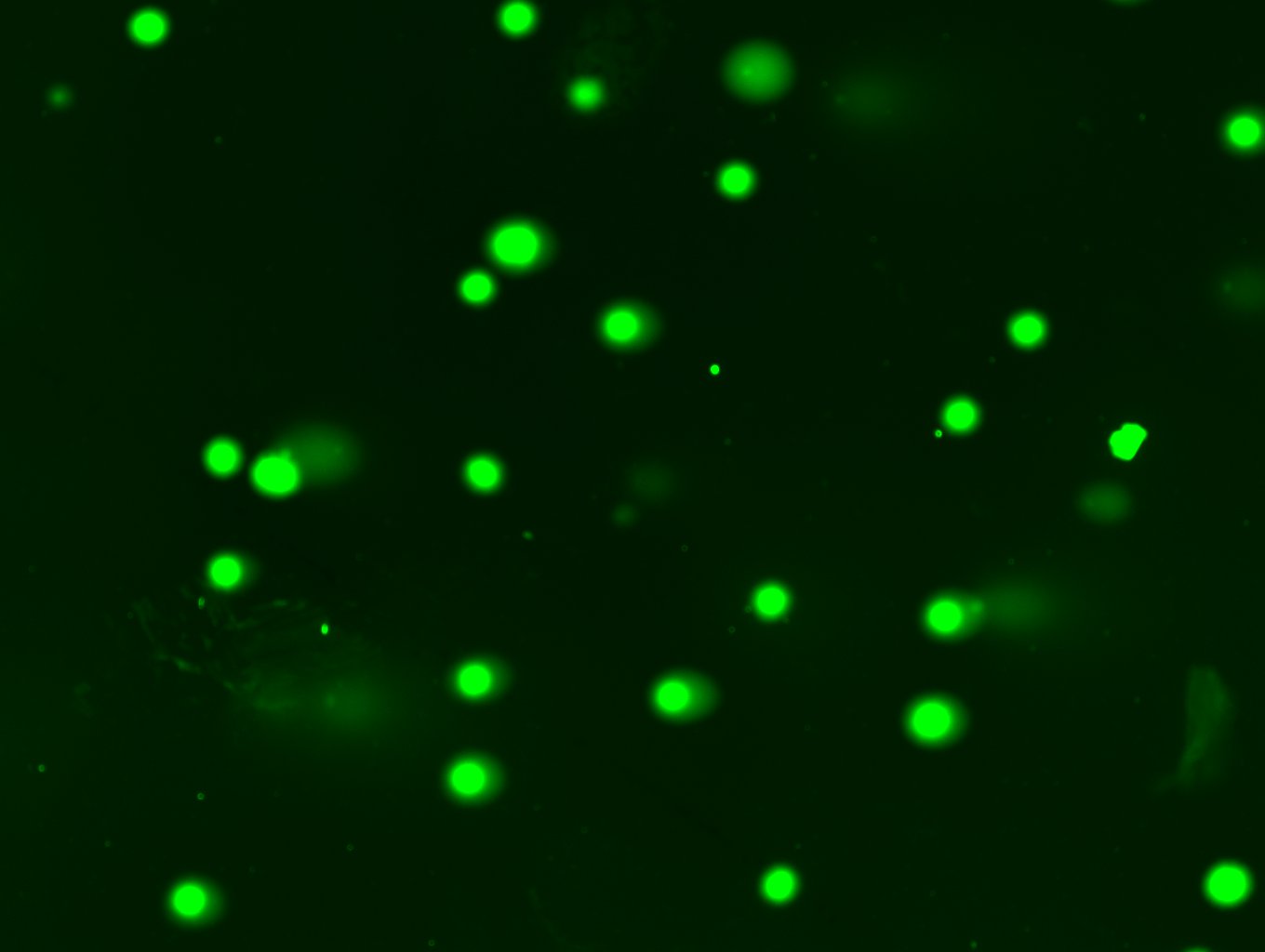

Supplement: Supplementary file 9 — Source Data for Figure 2 [file EMMM-15-e17313-s002.zip › Figure 2/E/Comet_Files_For_quantification/Berzosertib7.jpg]

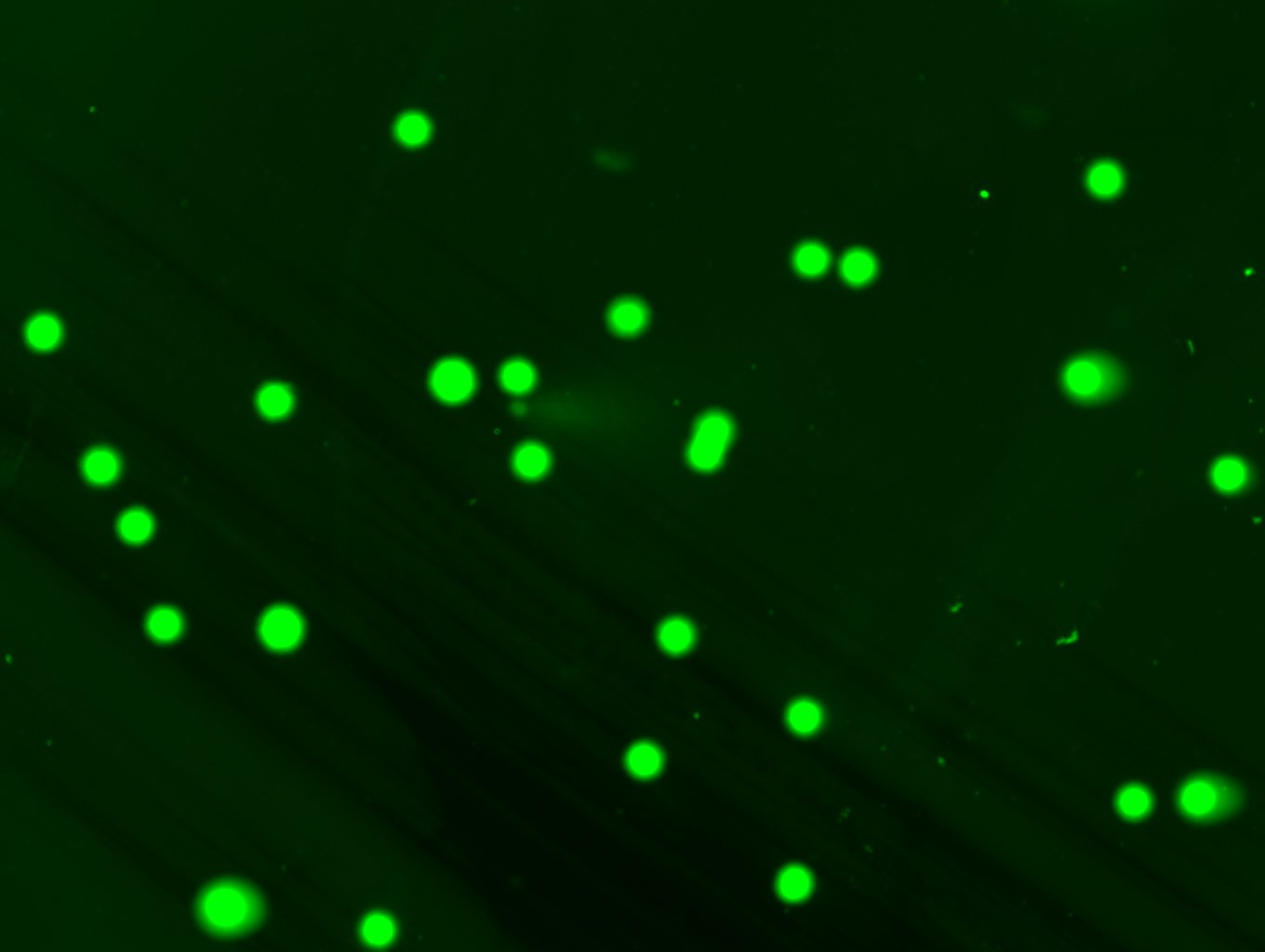

Supplement: Supplementary file 9 — Source Data for Figure 2 [file EMMM-15-e17313-s002.zip › Figure 2/E/Comet_Files_For_quantification/Berzosertib8.jpg]

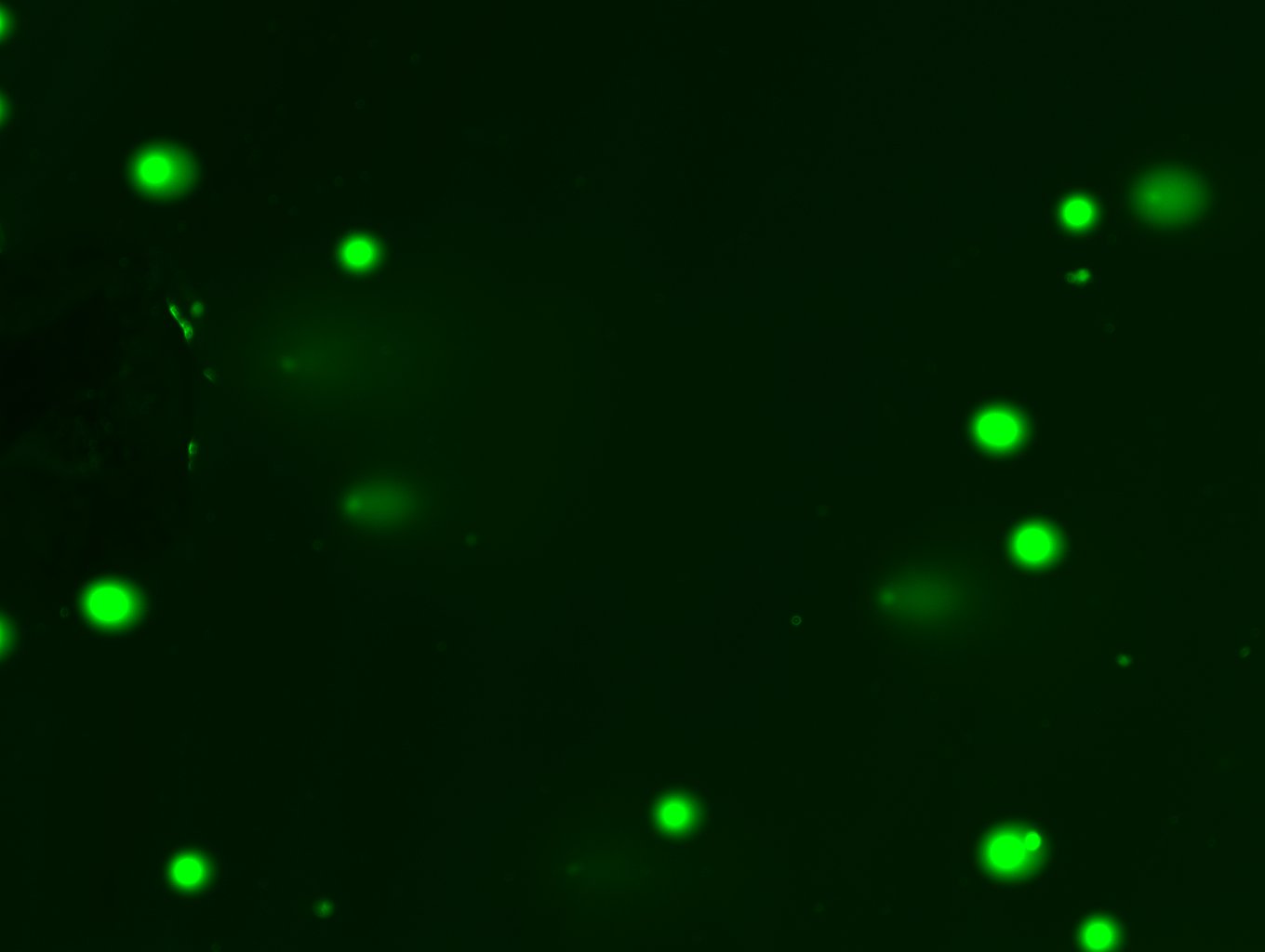

Supplement: Supplementary file 9 — Source Data for Figure 2 [file EMMM-15-e17313-s002.zip › Figure 2/E/Comet_Files_For_quantification/combi 1.jpg]

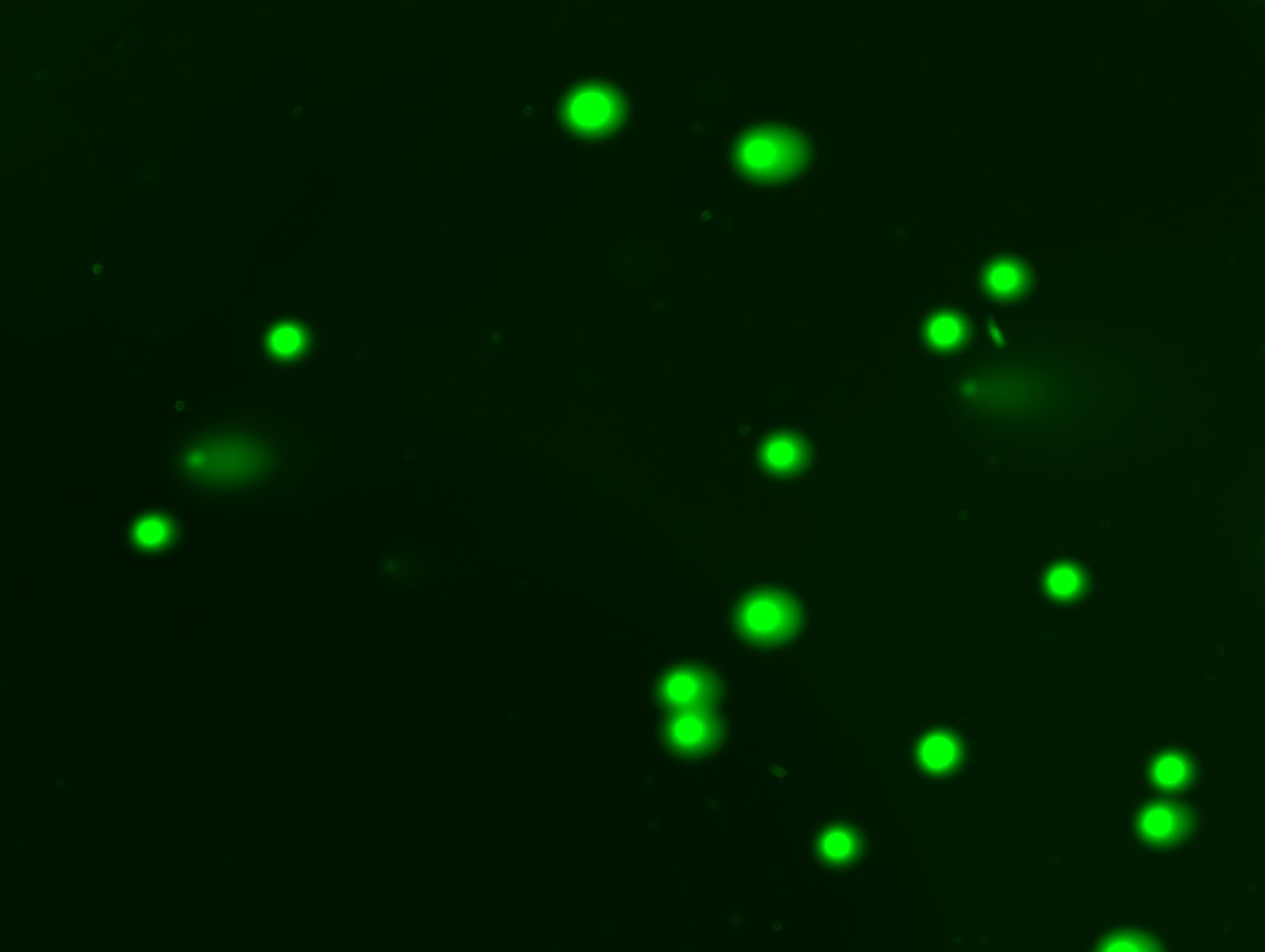

Supplement: Supplementary file 9 — Source Data for Figure 2 [file EMMM-15-e17313-s002.zip › Figure 2/E/Comet_Files_For_quantification/combi 2.jpg]

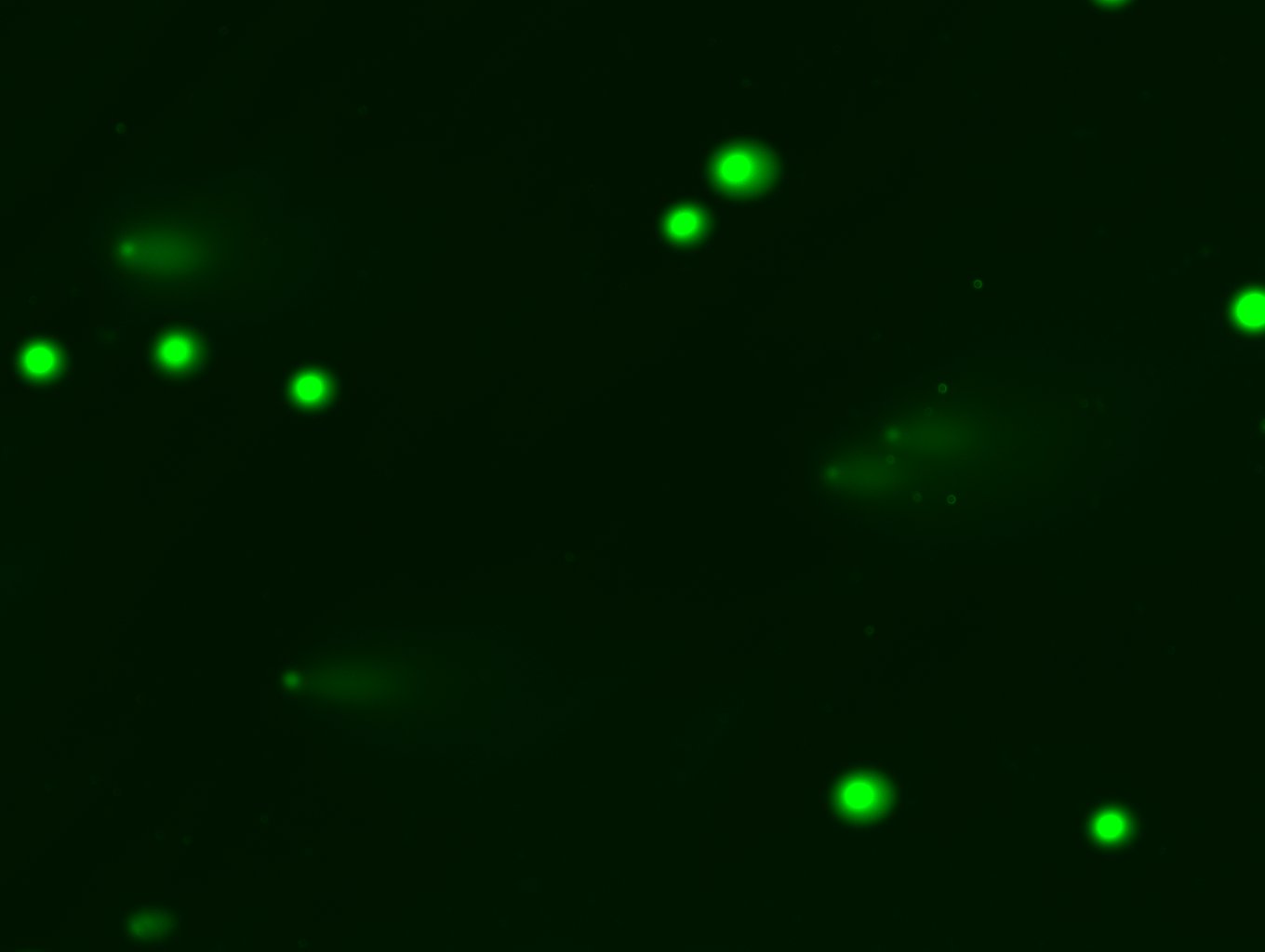

Supplement: Supplementary file 9 — Source Data for Figure 2 [file EMMM-15-e17313-s002.zip › Figure 2/E/Comet_Files_For_quantification/combi 3.jpg]

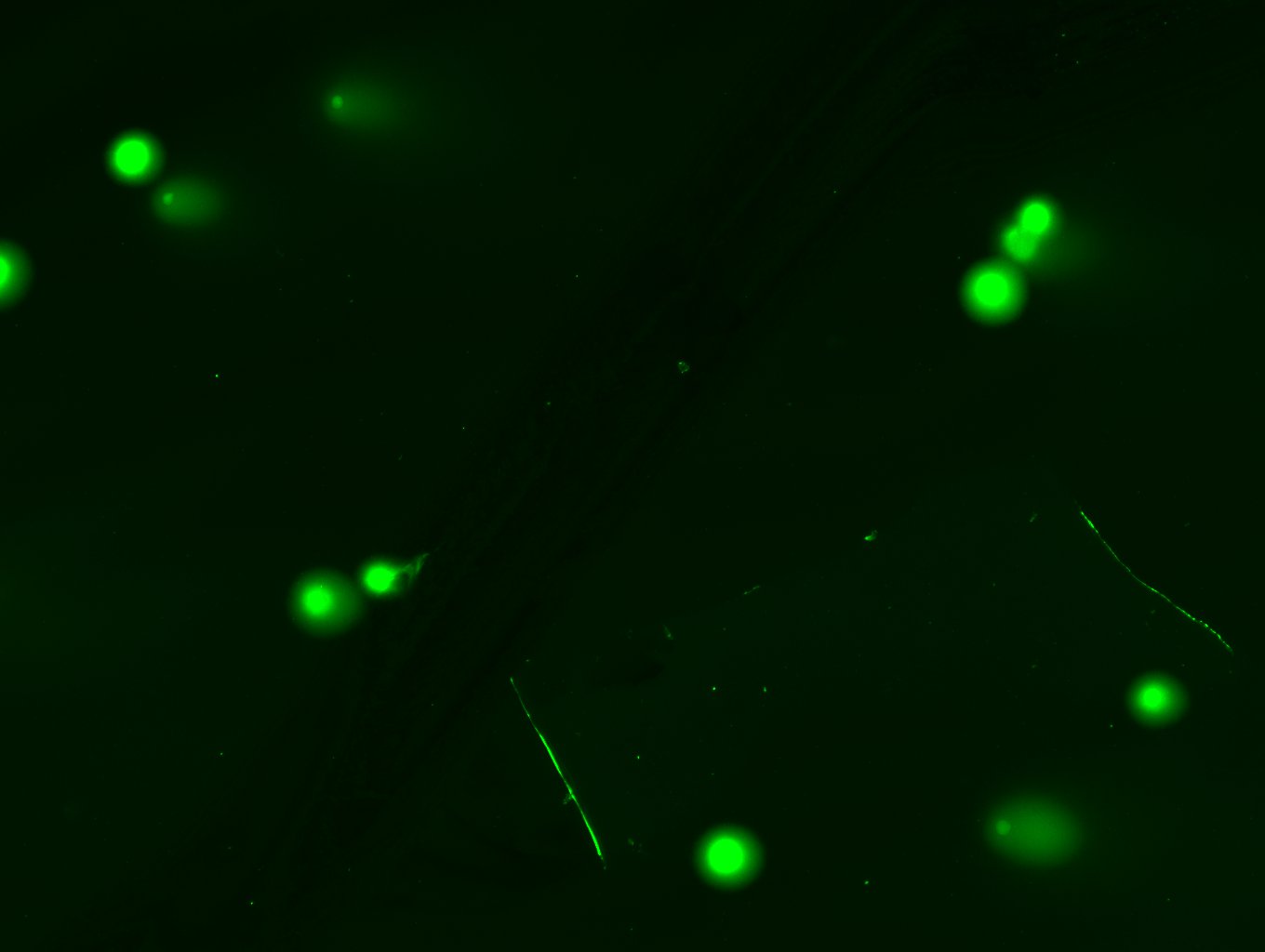

Supplement: Supplementary file 9 — Source Data for Figure 2 [file EMMM-15-e17313-s002.zip › Figure 2/E/Comet_Files_For_quantification/combi10.jpg]

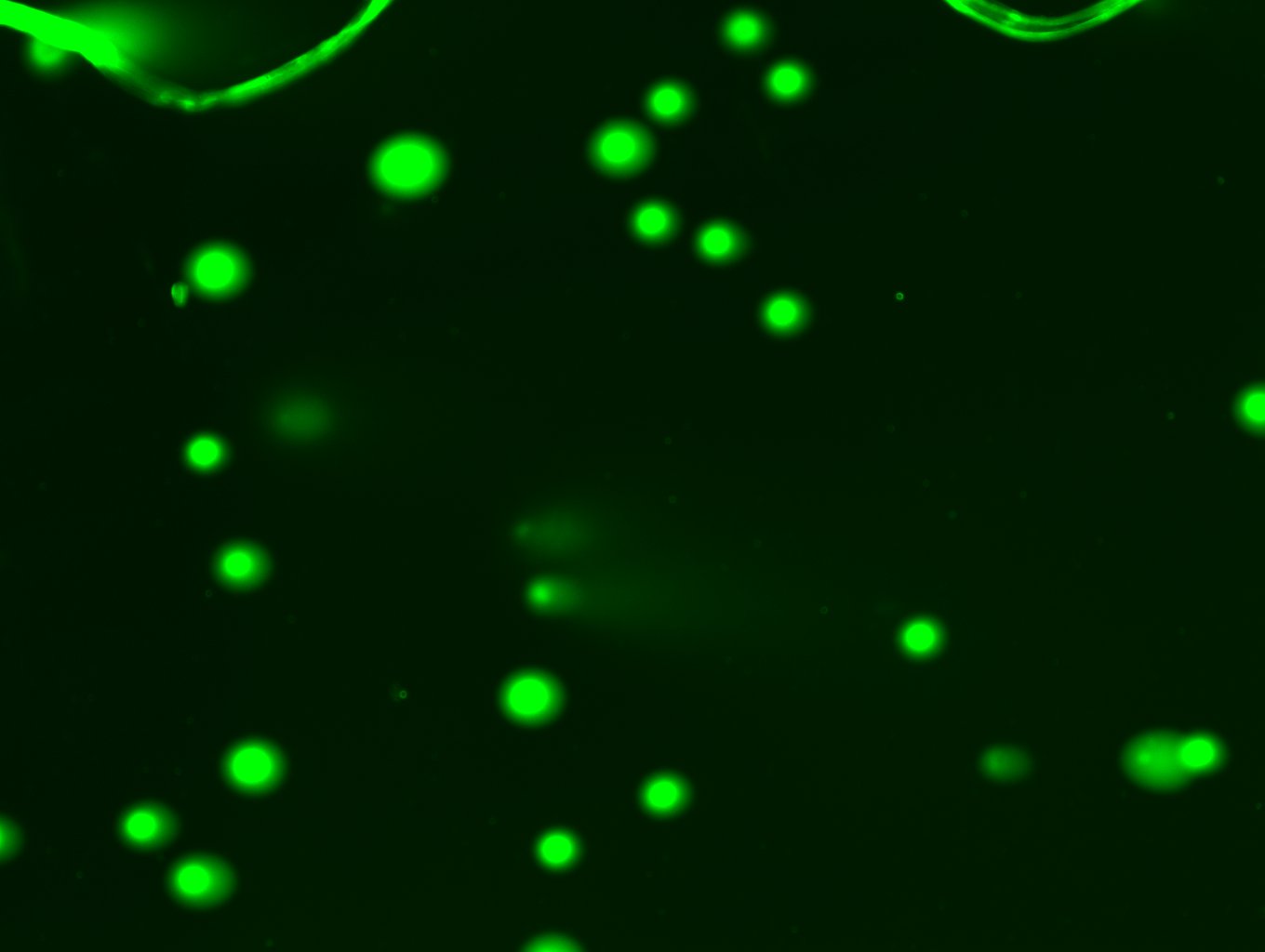

Supplement: Supplementary file 9 — Source Data for Figure 2 [file EMMM-15-e17313-s002.zip › Figure 2/E/Comet_Files_For_quantification/combi4.jpg]

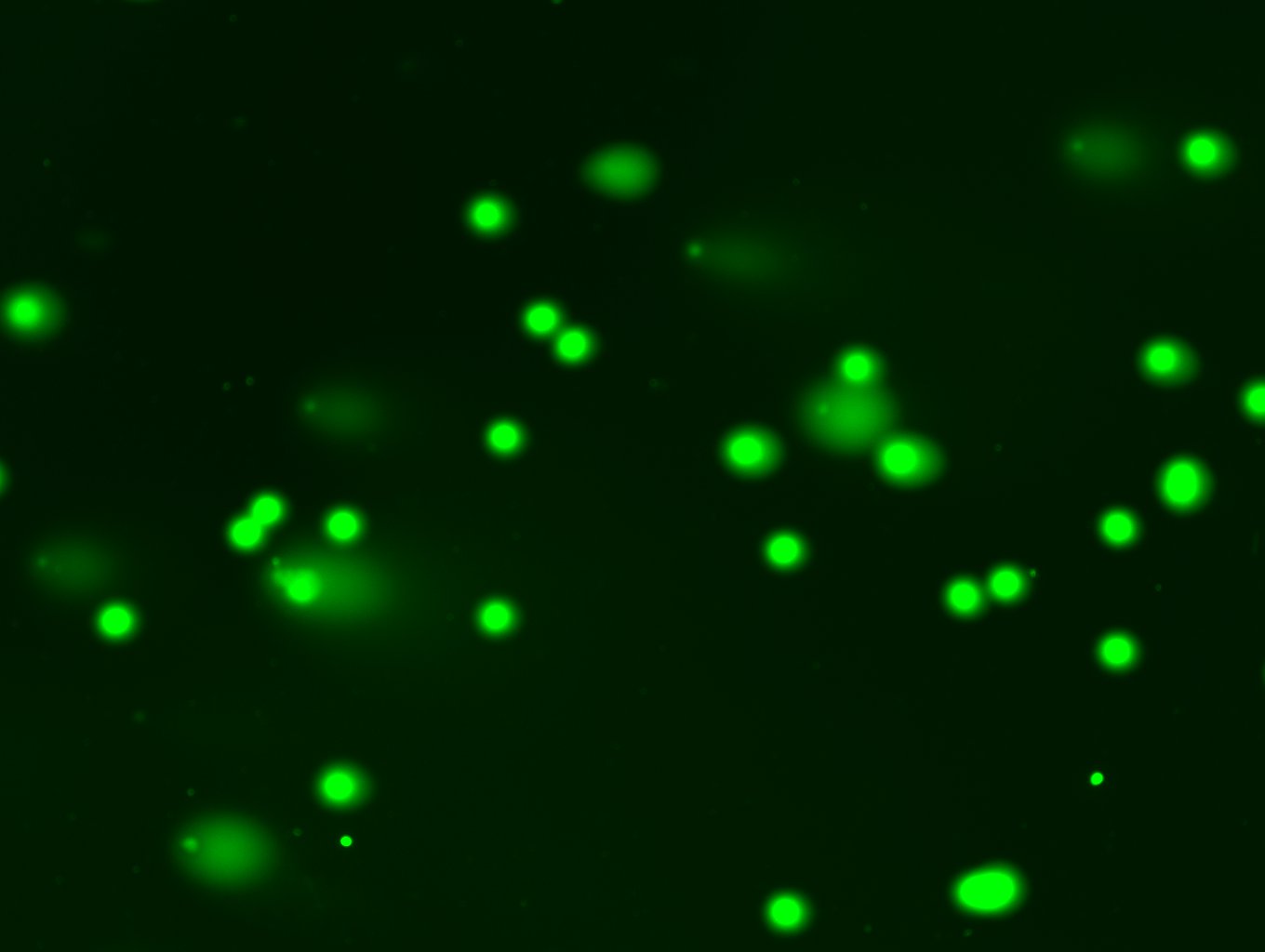

Supplement: Supplementary file 9 — Source Data for Figure 2 [file EMMM-15-e17313-s002.zip › Figure 2/E/Comet_Files_For_quantification/combi5.jpg]

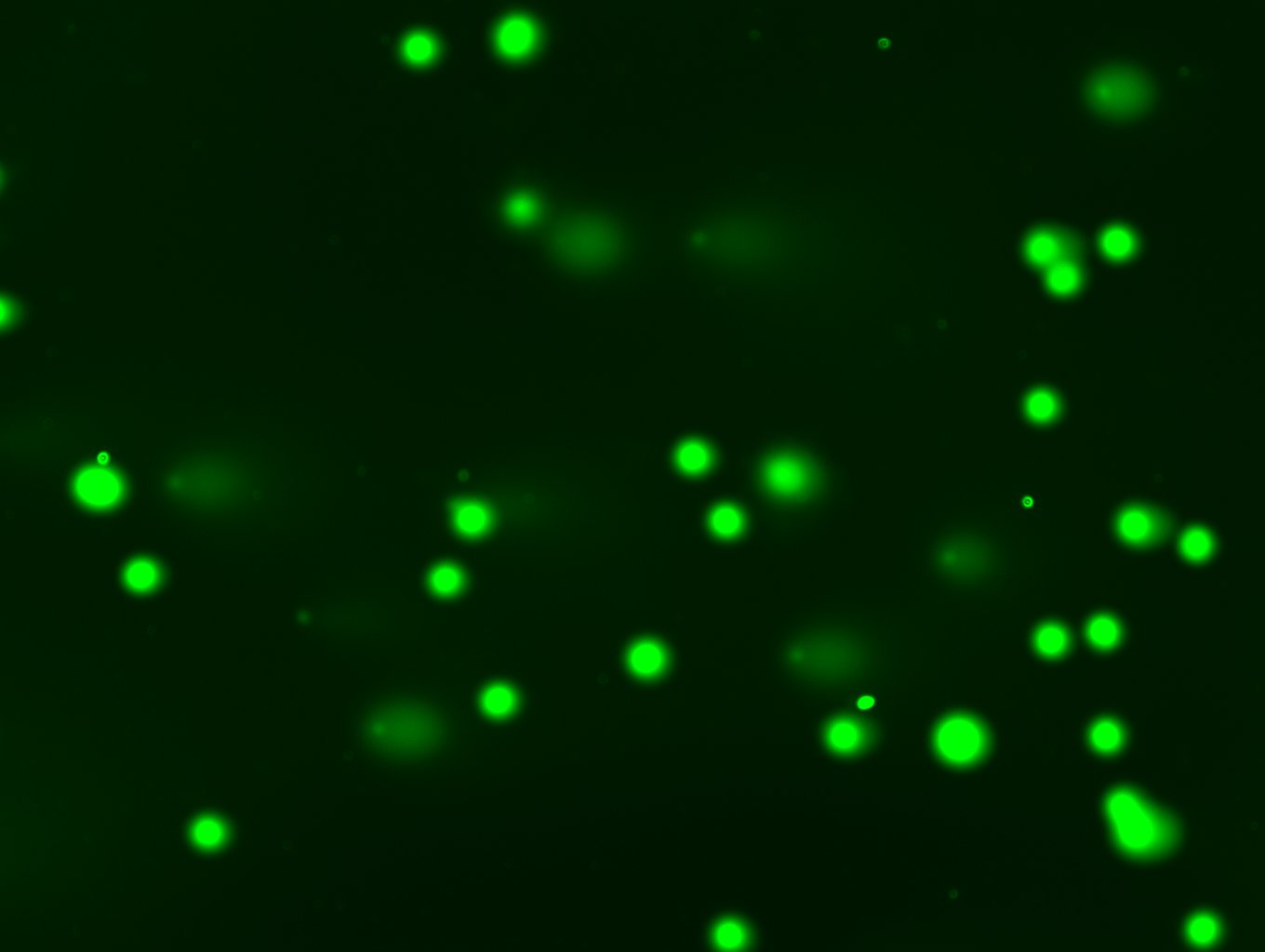

Supplement: Supplementary file 9 — Source Data for Figure 2 [file EMMM-15-e17313-s002.zip › Figure 2/E/Comet_Files_For_quantification/combi6.jpg]

## Slide 1
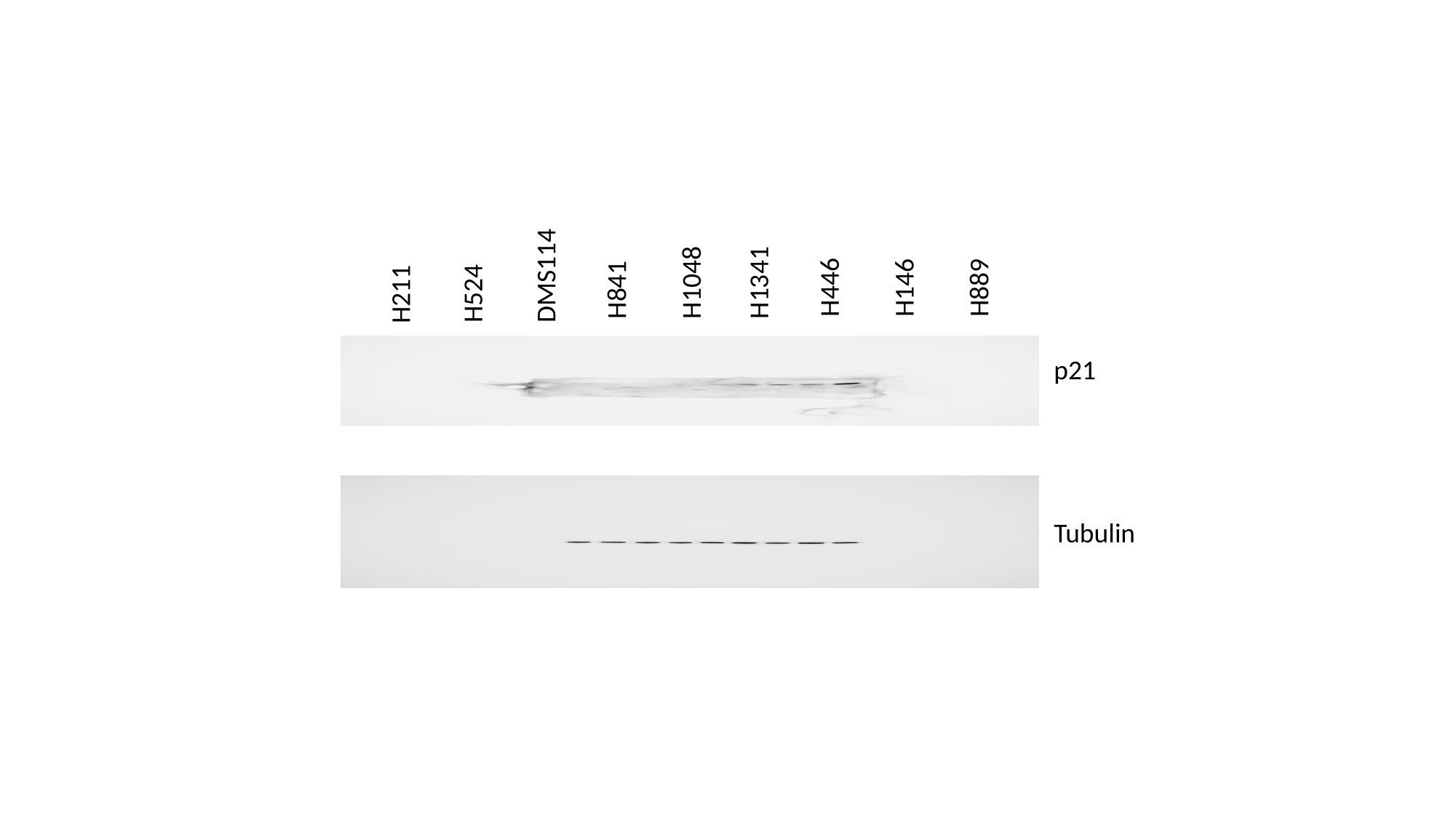

DMS114
H446
H889
H146
H841
H1048
H1341
H524
H211
p21
Tubulin

## Slide 2
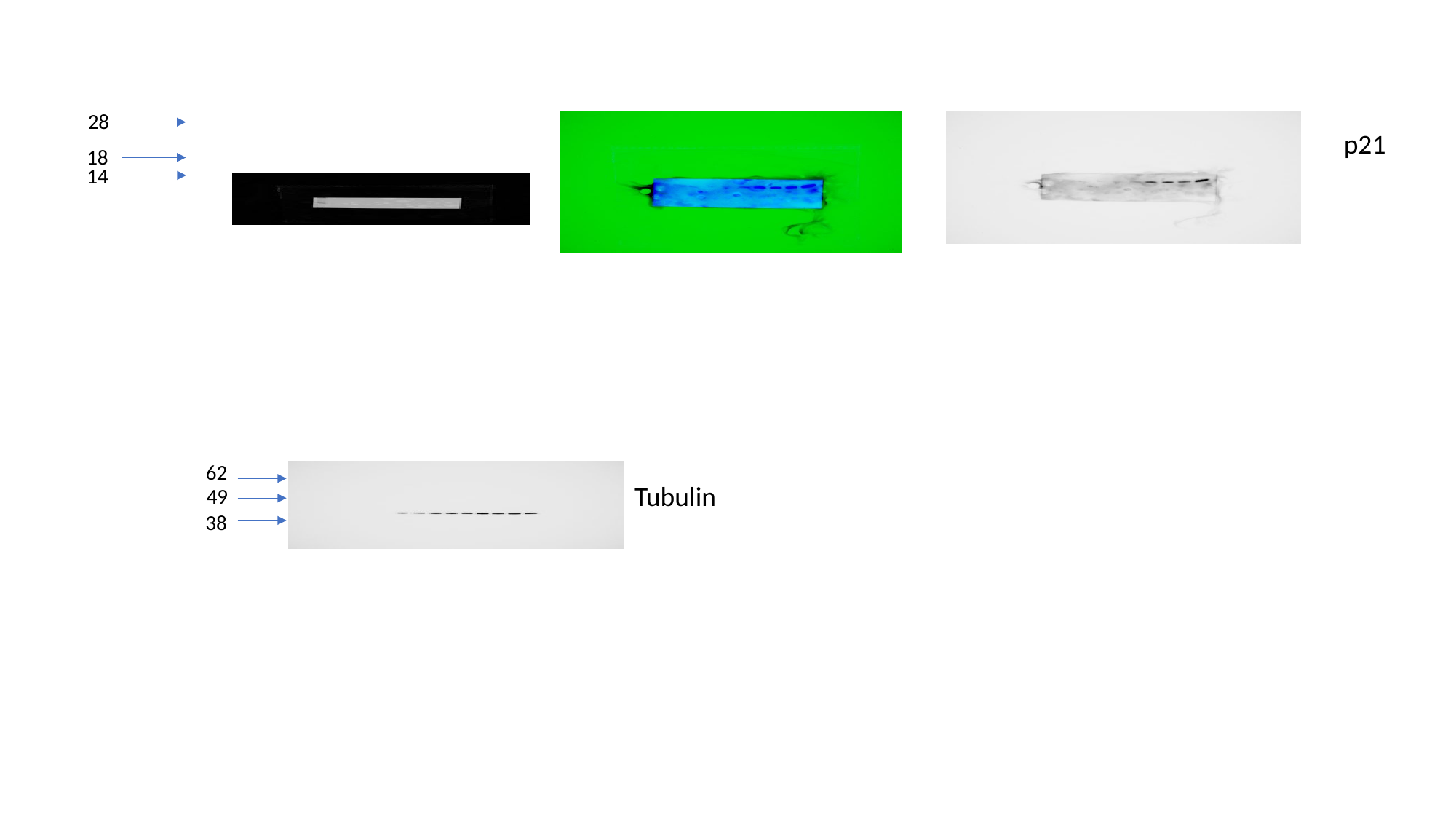

28
p21
18
14
62
Tubulin
49
38

Supplement: Supplementary file 10 — Source Data for Figure 3 [file EMMM-15-e17313-s009.zip › Figure 3_1/E/9 Cell Lines blots/9_cell_lines.pptx]

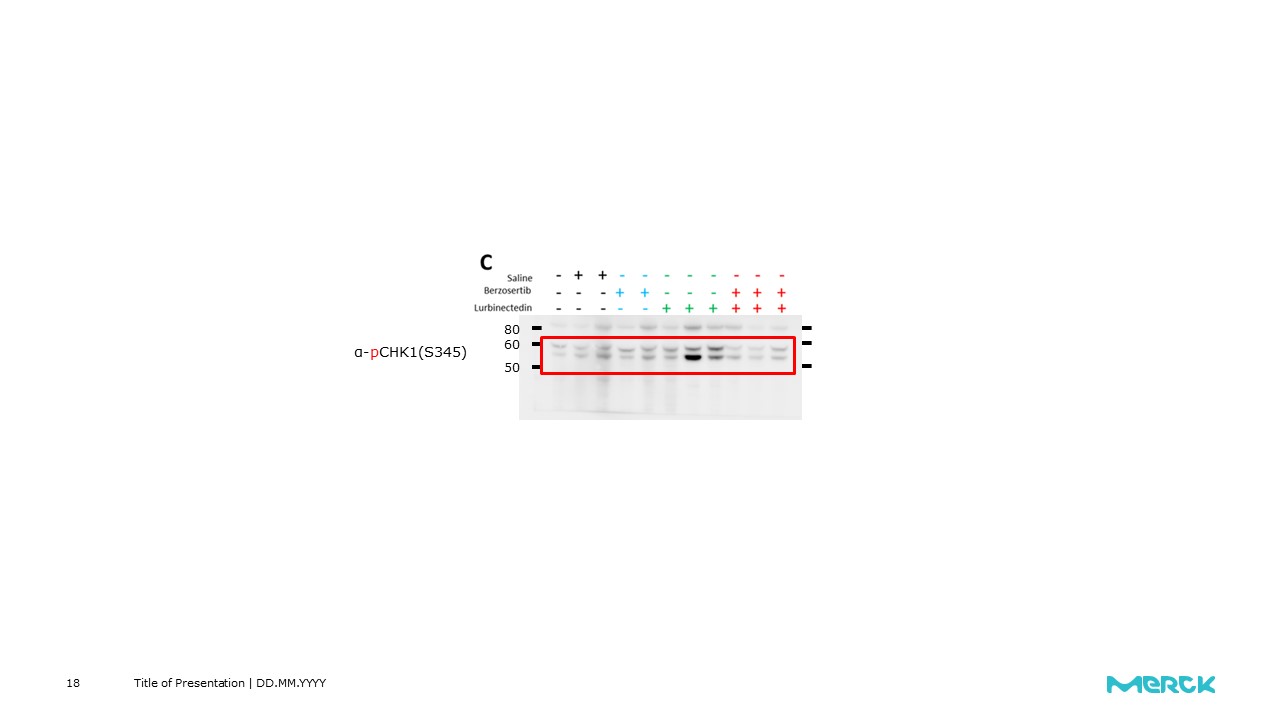

Supplement: Supplementary file 11 — Source Data for Figure 4 [file EMMM-15-e17313-s011.zip › Figure 4/C/pCHK1(S345).JPG]

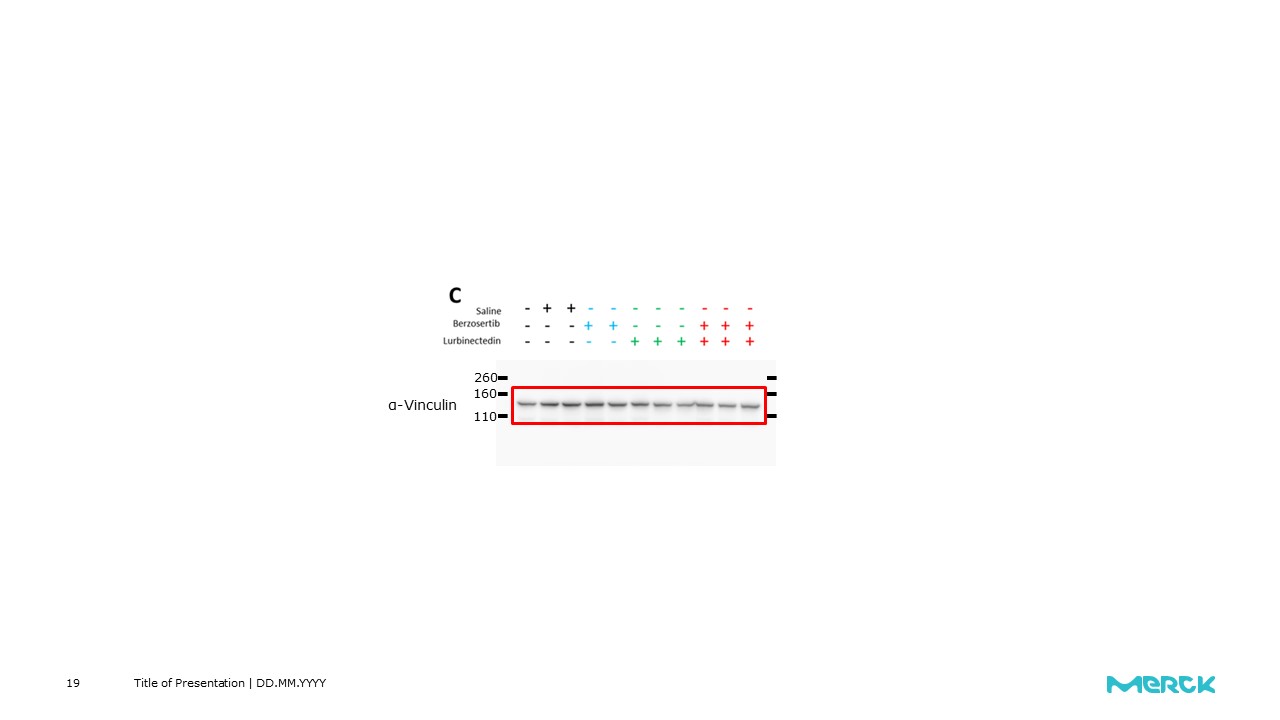

Supplement: Supplementary file 11 — Source Data for Figure 4 [file EMMM-15-e17313-s011.zip › Figure 4/C/Vinculin.JPG]

# Synergy mapped to D-R (HSA)

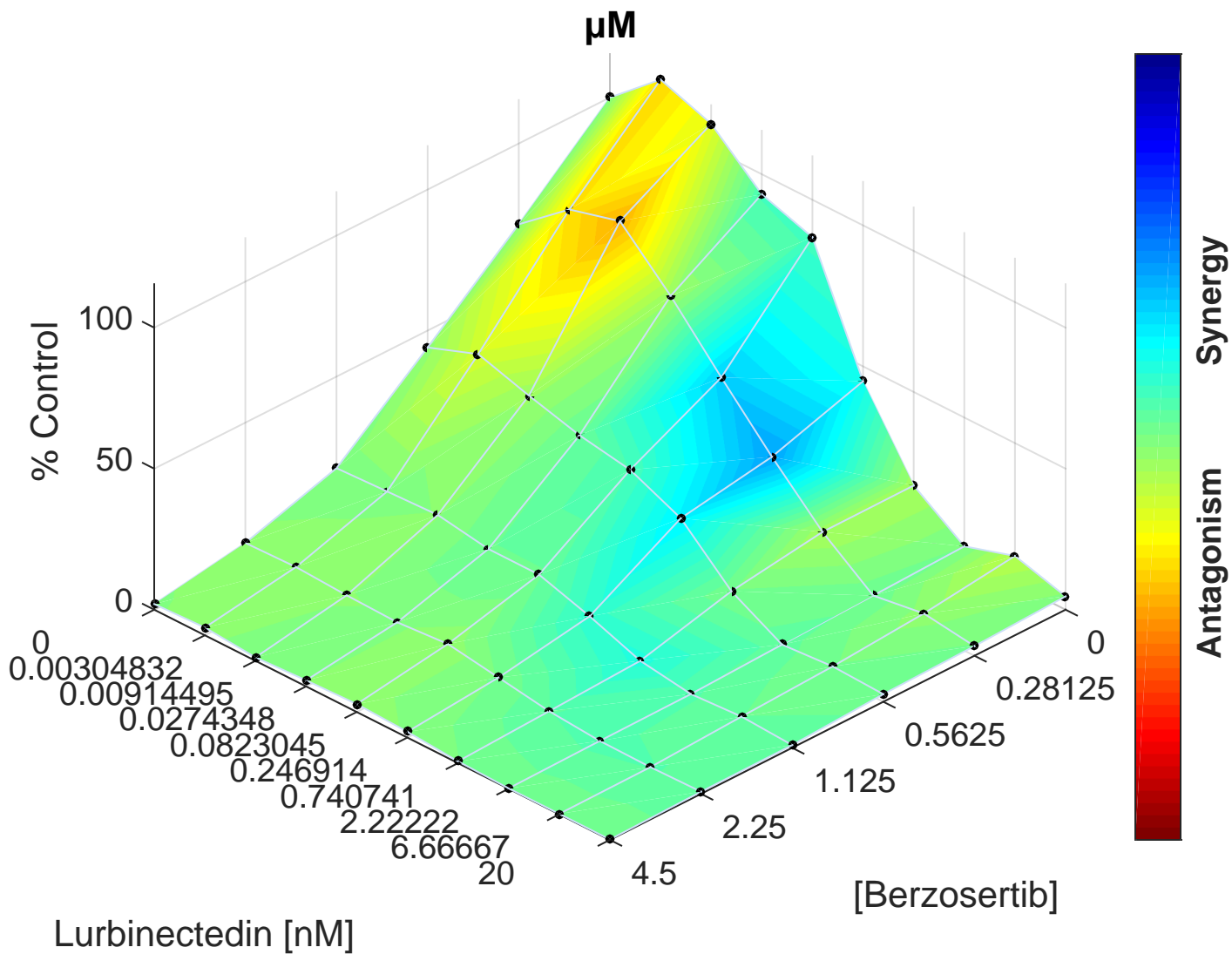

Supplement: Supplementary file 11 — Source Data for Figure 4 [file EMMM-15-e17313-s011.zip › Figure 4/D/Group_1/Mapped_Surface_HSA_SYN_ANT_μM.pdf]

# Synergy mapped to D-R (HSA)

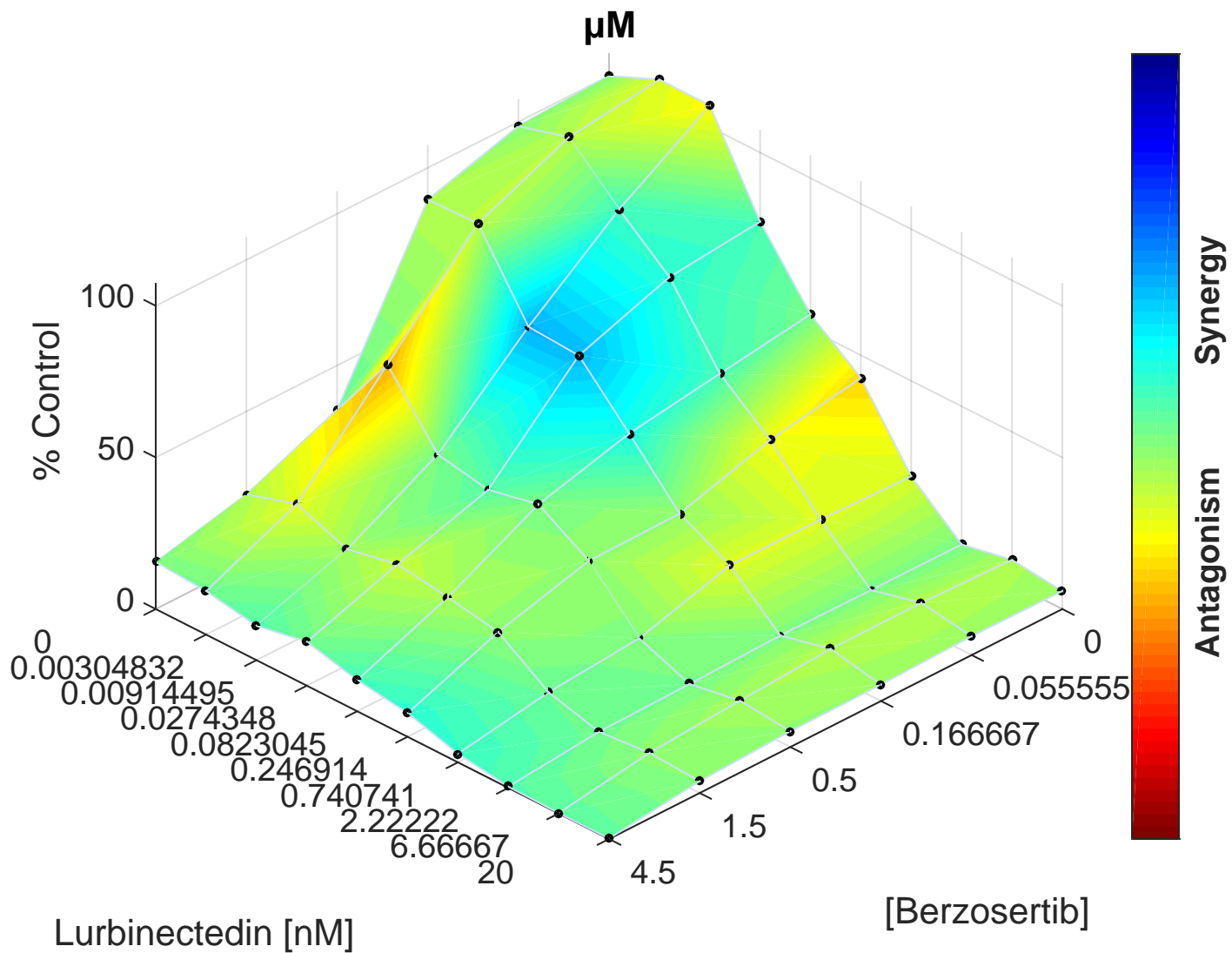

Supplement: Supplementary file 11 — Source Data for Figure 4 [file EMMM-15-e17313-s011.zip › Figure 4/D/Group_2/Mapped_Surface_HSA_SYN_ANT_μM.pdf]

# Synergy mapped to D-R (HSA)

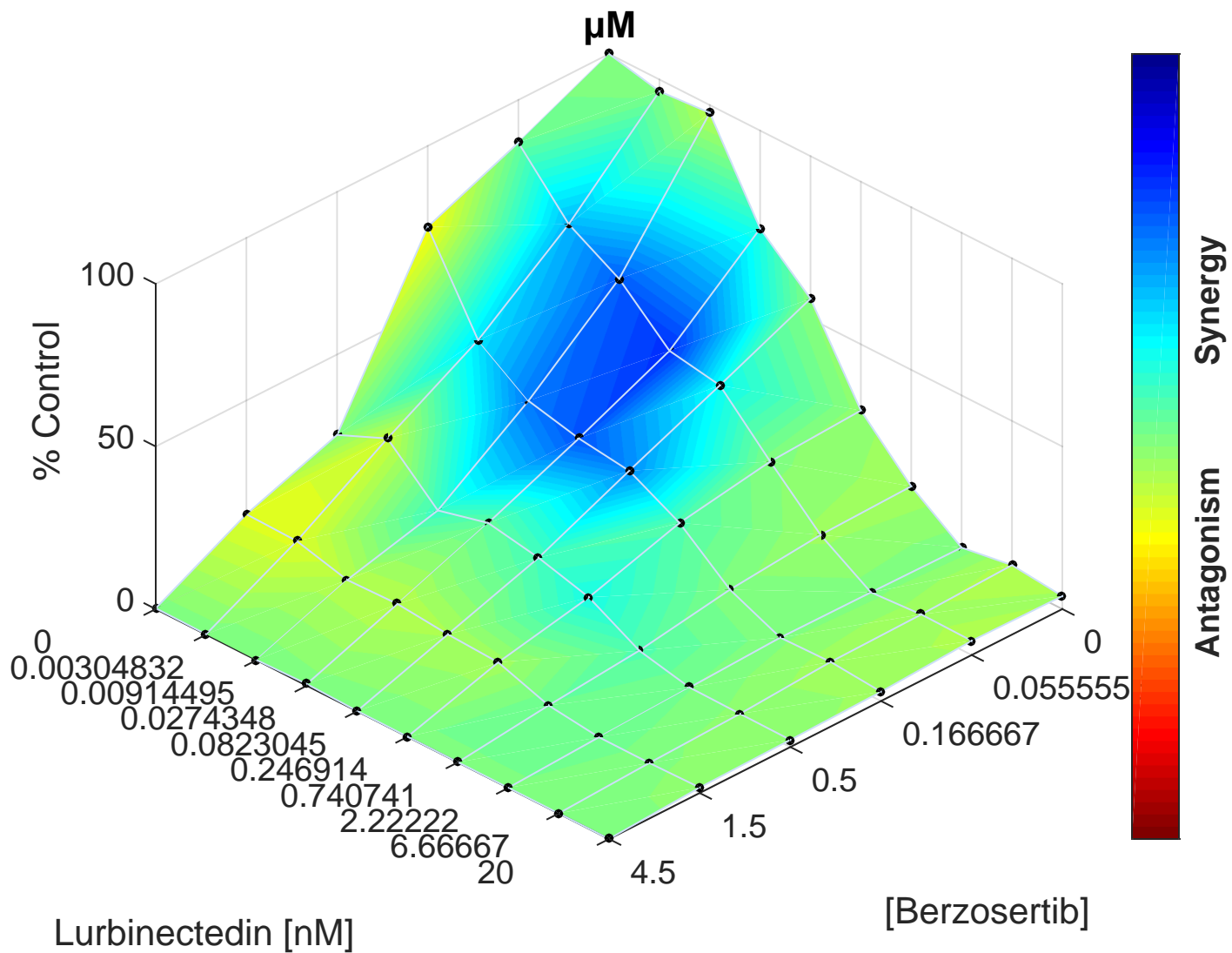

Supplement: Supplementary file 11 — Source Data for Figure 4 [file EMMM-15-e17313-s011.zip › Figure 4/D/Group_3/Mapped_Surface_HSA_SYN_ANT_μM.pdf]

# Synergy mapped to D-R (HSA)

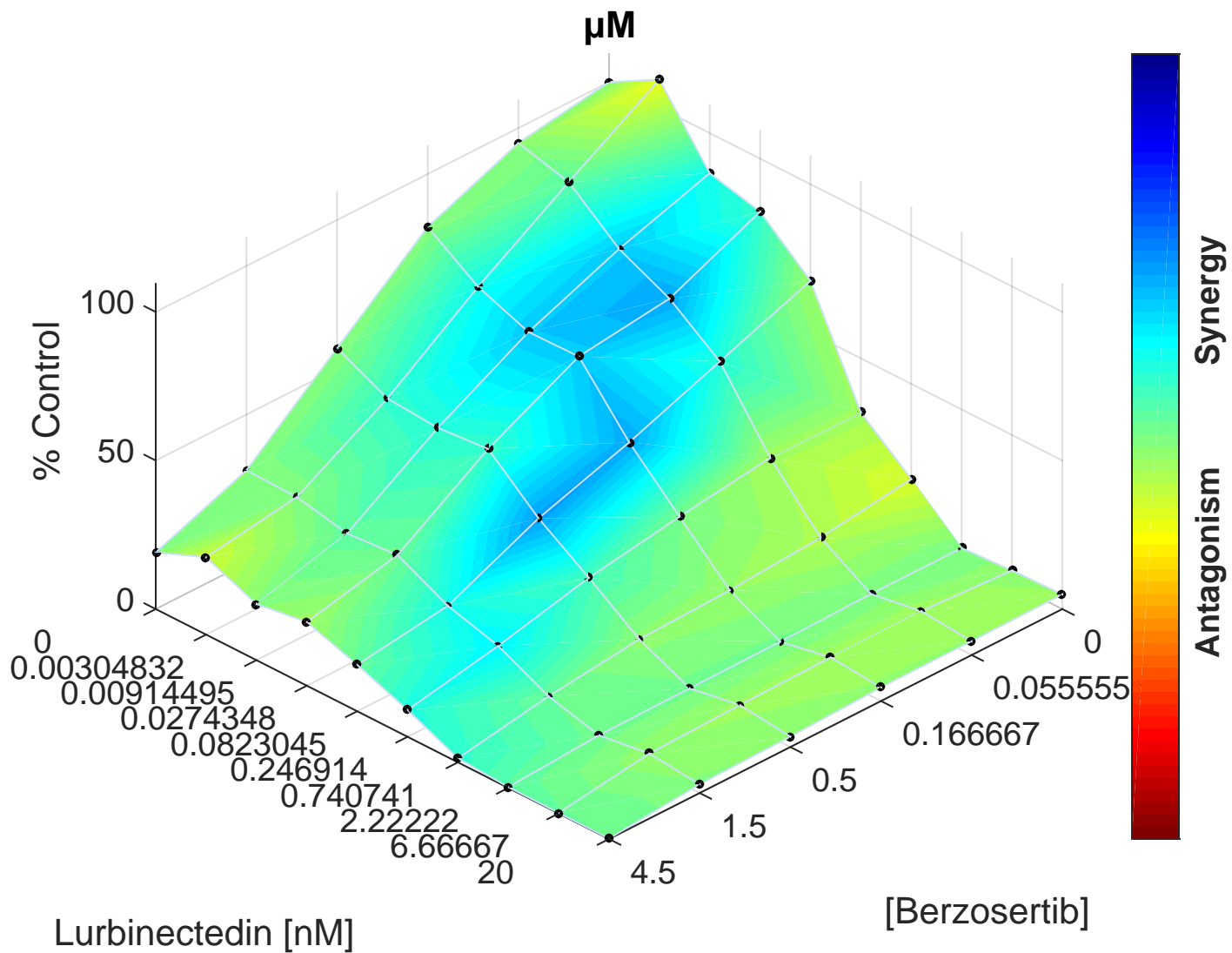

Supplement: Supplementary file 11 — Source Data for Figure 4 [file EMMM-15-e17313-s011.zip › Figure 4/D/Group_4/Mapped_Surface_HSA_SYN_ANT_μM.pdf]
