# Supplementary material for: ATR inhibition augments the efficacy of lurbinectedin in small‐cell lung cancer
Source: EMBO Mol Med. 2023 Jul 25;15(8):e17313. doi: 10.15252/emmm.202217313 (PMC10405061; doi:10.15252/emmm.202217313)
Supplement: Supplementary file 11 — Source Data for Figure 4 [file EMMM-15-e17313-s011.zip › Figure 4/E/p21 and SLFN11 comparison between lines.pptx]

## Slide 1
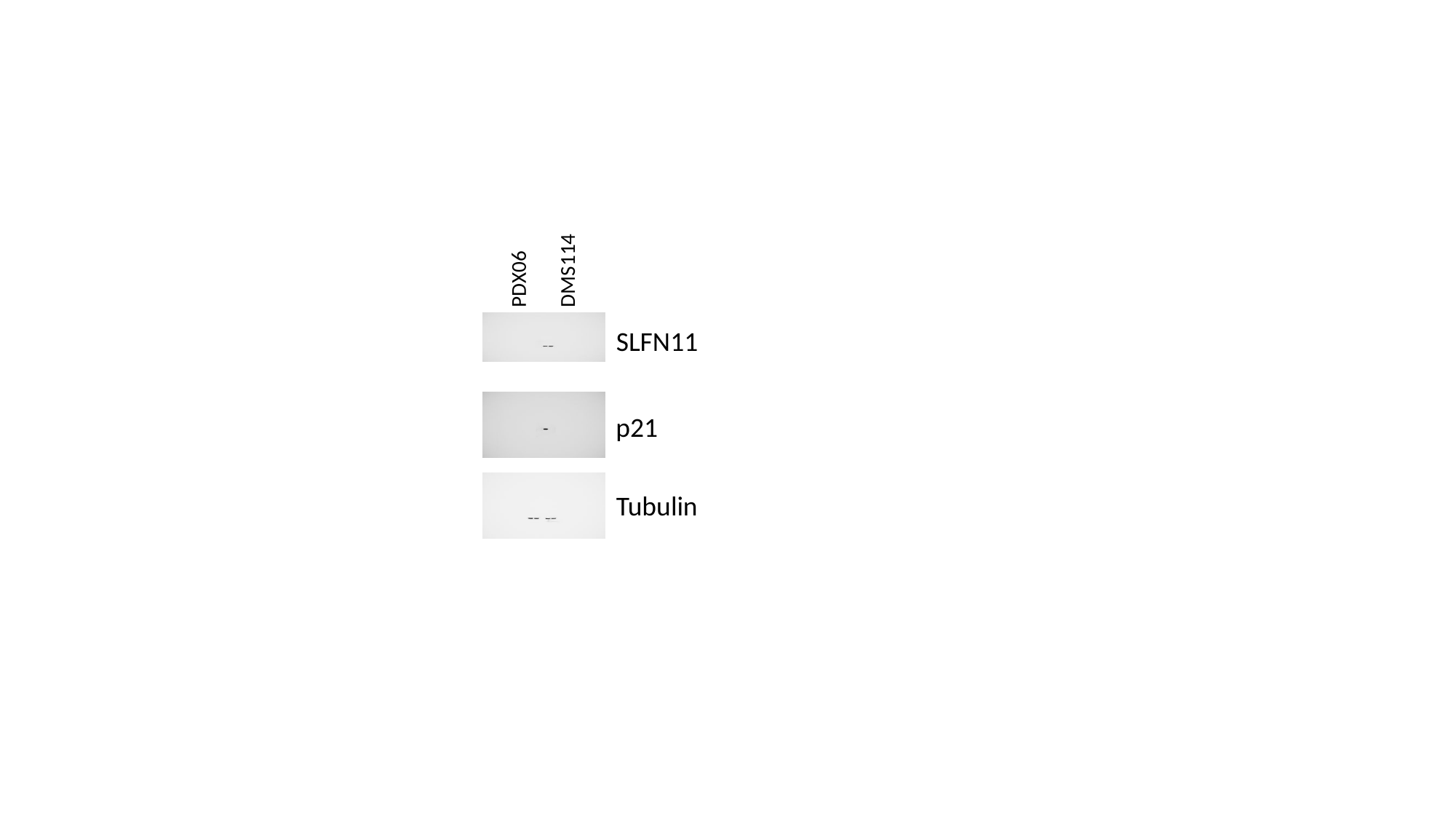

DMS114
PDX06
SLFN11
p21
Tubulin

## Slide 2
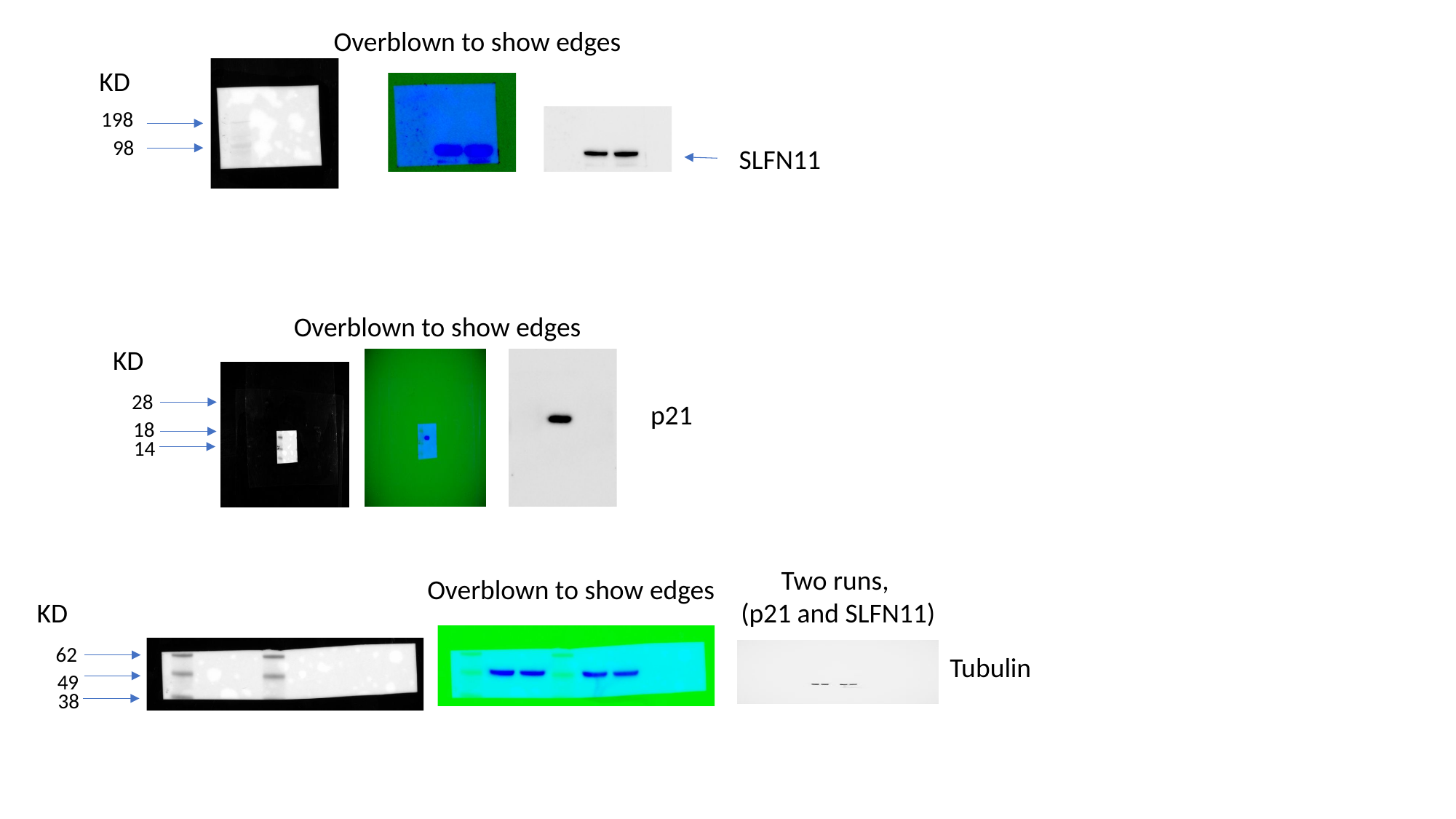

Overblown to show edges
KD
198
98
SLFN11
Overblown to show edges
KD
28
p21
18
14
Two runs,
(p21 and SLFN11)
Overblown to show edges
KD
62
Tubulin
49
38
