# Supplementary material for: ATR inhibition augments the efficacy of lurbinectedin in small‐cell lung cancer
Source: EMBO Mol Med. 2023 Jul 25;15(8):e17313. doi: 10.15252/emmm.202217313 (PMC10405061; doi:10.15252/emmm.202217313)
Supplement: Supplementary file 13 [file EMMM-15-e17313-s001.pdf]

EMBO Molecular Medicine

ATR inhibition augments the efficacy of lurbinectedin in small cell lung cancer

Christopher Schultz, Yang Zhang, Rajaa Elmeskini, Astrid Zimmermann, Haiqing Fu, Yasuhisa Murai, Darawalee Wangsa, Suresh Kumar, Nobuyuki Takahashi, Devon Atkinson, Liton Saha, Chien-Fei Lee, Brian Elenbaas, Parth Desai, Robin Sebastian, Ajit Kumar Sharma, Melissa Abel, Brett Schroeder, Manan Krishnamurthy, Rajesh Kumar, Nitin Roper, Mirit Aladjem, Frank Zenke, Zoe Weaver Ohler, Yves Pommier, and Anish Thomas
DOI: 10.15252/emmm.202217313

Corresponding author(s): Anish Thomas (anish.thomas@nih.gov)

Review Timeline:

Submission Date:	16th Dec 22
Editorial Decision:	16th Jan 23
Revision Received:	2nd May 23
Editorial Decision:	22nd May 23
Revision Received:	12th Jun 23
Accepted:	19th Jun 23

Editor: Zeljko Durdevic

Transaction Report:

(Note: With the exception of the correction of typographical or spelling errors that could be a source of ambiguity, letters and reports are not edited. Depending on transfer agreements, referee reports obtained elsewhere may or may not be included in this compilation. Referee reports are anonymous unless the Referee chooses to sign their reports.)

16th Jan 2023

Dear Dr. Thomas,

Thank you for the submission of your manuscript to EMBO Molecular Medicine and please accept my apologies for the delay in getting back to you due to the holiday season. We have now received feedback from the two reviewers who agreed to evaluate your manuscript. As you will see from the reports below, both referees recognize potential interest of the study, but also raise important concerns that should be addressed in a major revision.

Further consideration of a revision that addresses reviewers' concerns in full will entail a second round of review. EMBO Molecular Medicine encourages a single round of revision only and therefore, acceptance or rejection of the manuscript will depend on the completeness of your responses included in the next, final version of the manuscript. For this reason, and to save you from any frustrations in the end, I would strongly advise against returning an incomplete revision.

We would welcome the submission of a revised version within three months for further consideration. Please let us know if you require longer to complete the revision.

I look forward to receiving your revised manuscript.

Yours sincerely,

Zeljko Durdevic

Zeljko Durdevic
Editor
EMBO Molecular Medicine

*****

When submitting your revised manuscript, please carefully review the instructions that follow below. We perform an initial quality control of all revised manuscripts before re-review; failure to include requested items will delay the evaluation of your revision.

We require:

1) A .docx formatted version of the manuscript text (including legends for main figures, EV figures and tables). Please make sure that the changes are highlighted to be clearly visible.

2) Individual production quality figure files as .eps, .tif, .jpg (one file per figure). For guidance, download the 'Figure Guide PDF': (https://www.embopress.org/page/journal/17574684/authorguide#figureformat).

3) A .docx formatted letter INCLUDING the reviewers' reports and your detailed point-by-point responses to their comments. As part of the EMBO Press transparent editorial process, the point-by-point response is part of the Review Process File (RPF), which will be published alongside your paper.

4) A complete author checklist, which you can download from our author guidelines (https://www.embopress.org/page/journal/17574684/authorguide#submissionofrevisions). Please insert information in the checklist that is also reflected in the manuscript. The completed author checklist will also be part of the RPF.

5) Please note that all corresponding authors are required to supply an ORCID ID for their name upon submission of a revised manuscript.

6) It is mandatory to include a 'Data Availability' section after the Materials and Methods. Before submitting your revision, primary datasets produced in this study need to be deposited in an appropriate public database, and the accession numbers and

database listed under 'Data Availability'. Please remember to provide a reviewer password if the datasets are not yet public (see https://www.embopress.org/page/journal/17574684/authorguide#dataavailability).

In case you have no data that requires deposition in a public database, please state so in this section. Note that the Data Availability Section is restricted to new primary data that are part of this study.

7) For data quantification: please specify the name of the statistical test used to generate error bars and P values, the number (n) of independent experiments (specify technical or biological replicates) underlying each data point and the test used to calculate p-values in each figure legend. The figure legends should contain a basic description of n, P and the test applied. Graphs must include a description of the bars and the error bars (s.d., s.e.m.). See also 'Figure Legend' guidelines: https://www.embopress.org/page/journal/17574684/authorguide#figureformat

8) At EMBO Press we ask authors to provide source data for the main manuscript figures. Our source data coordinator will contact you to discuss which figure panels we would need source data for and will also provide you with helpful tips on how to upload and organize the files.

9) Our journal encourages inclusion of *data citations in the reference list* to directly cite datasets that were re-used and obtained from public databases. Data citations in the article text are distinct from normal bibliographical citations and should directly link to the database records from which the data can be accessed. In the main text, data citations are formatted as follows: "Data ref: Smith et al, 2001" or "Data ref: NCBI Sequence Read Archive PRJNA342805, 2017". In the Reference list, data citations must be labeled with "[DATASET]". A data reference must provide the database name, accession number/identifiers and a resolvable link to the landing page from which the data can be accessed at the end of the reference. Further instructions are available at .

10) We replaced Supplementary Information with Expanded View (EV) Figures and Tables that are collapsible/expandable online. A maximum of 5 EV Figures can be typeset. EV Figures should be cited as 'Figure EV1, Figure EV2" etc... in the text and their respective legends should be included in the main text after the legends of regular figures.

- For the figures that you do NOT wish to display as Expanded View figures, they should be bundled together with their legends in a single PDF file called *Appendix*, which should start with a short Table of Content. Appendix figures should be referred to in the main text as: "Appendix Figure S1, Appendix Figure S2" etc.

- Additional Tables/Datasets should be labeled and referred to as Table EV1, Dataset EV1, etc. Legends have to be provided in a separate tab in case of .xls files. Alternatively, the legend can be supplied as a separate text file (README) and zipped together with the Table/Dataset file.

See detailed instructions here:

.

11) The paper explained: EMBO Molecular Medicine articles are accompanied by a summary of the articles to emphasize the major findings in the paper and their medical implications for the non-specialist reader. Please provide a draft summary of your article highlighting

- the medical issue you are addressing,

- the results obtained and

- their clinical impact.

This may be edited to ensure that readers understand the significance and context of the research. Please refer to any of our published articles for an example.

12) For more information: There is space at the end of each article to list relevant web links for further consultation by our readers. Could you identify some relevant ones and provide such information as well? Some examples are patient associations, relevant databases, OMIM/proteins/genes links, author's websites, etc...

13) Author contributions: You will be asked to provide CRediT (Contributor Role Taxonomy) terms in the submission system. These replace a narrative author contribution section in the manuscript.

14) A Conflict of Interest statement should be provided in the main text.

15) Every published paper now includes a 'Synopsis' to further enhance discoverability. Synopses are displayed on the journal

webpage and are freely accessible to all readers. They include a short stand first (maximum of 300 characters, including space) as well as 2-5 one-sentences bullet points that summarizes the paper. Please write the bullet points to summarize the key NEW findings. They should be designed to be complementary to the abstract - i.e. not repeat the same text. We encourage inclusion of key acronyms and quantitative information (maximum of 30 words / bullet point). Please use the passive voice. Please attach these in a separate file or send them by email, we will incorporate them accordingly.

Please also suggest a striking image or visual abstract to illustrate your article as a PNG file 550 px wide x 300-600 px high.

EMBO Molecular Medicine has a "scooping protection" policy, whereby similar findings that are published by others during review or revision are not a criterion for rejection. Should you decide to submit a revised version, I do ask that you get in touch after three months if you have not completed it, to update us on the status.

Please note: When submitting your revision you will be prompted to enter your funding and payment information. This will allow Wiley to send you a quote for the article processing charge (APC) in case of acceptance. This quote takes into account any reduction or fee waivers that you may be eligible for. Authors do not need to pay any fees before their manuscript is accepted and transferred to the publisher.

EMBO Press participates in many Publish and Read agreements that allow authors to publish Open Access with reduced/no publication charges. Check your eligibility: https://authorservices.wiley.com/author-resources/Journal-Authors/open-access/affiliation-policies-payments/index.html

***** Reviewer's comments *****

Referee #1 (Comments on Novelty/Model System for Author):

The authors present compelling evidence that inhibiting ATR via treatments with berzosertib is effective for increasing lurbinectedin efficacy in SCLC cell lines as well as in organoid and in animal models. Overall, the work is well structured and written and the conclusions are supported from the original observations. I have a few minor points that could further improve the content of the work.

Referee #1 (Remarks for Author):

The authors present compelling evidence that inhibiting ATR via treatments with berzosertib is effective for increasing lurbinectedin efficacy in SCLC cell lines as well as in organoid and in animal models. Overall, the work is well structured and written and the conclusions are supported from the original observations. A few minor points could further improve the content of the work:
The authors state: "...Berzosertib is effective at inhibiting the activation of ATR and downstream ATR target CHK1 (indicated by red box), while not significantly impacting other pathways." However, Figure 1F shows that Berzosertib lowers pATM and pDNA-PK levels, contradicting the statement.
Figure 2b states "lurbinectedin caused a decrease in γH2AX expression". In fact, γH2AX, the phosphorylated version of histone H2AX. accumulates at DNA damage sites rather than "expressed". The authors should consider revise the term "expression".
Figure 2d: The images shown on Fig2D for "combination" vs. all other treatments do not really depict what the large differences shown on the bar graph in Fig2E
Figure S3A: Besides NER, XPG is also involved in homologous recombination as well as in resolving R-loops confounding the results on SSBs. The authors could use XPC (for GG-NER), CSB (for TC-NER) or XPA cells (for total NER).
Figure 4A please remove "was" from the first sentence.
Figure 5: the authors state that "Lurbinectedin binds to DNA and then induces DNA damage through transcription coupled nucleotide excision repair". It is unclear why the authors opt to disregard global genome NER in replicating cells or what the data are on TC-NER to support the statement in this work.

Referee #2 (Remarks for Author):

Lurbinectedin was a new drug recently approved for the treatment of relapsed SCLC. In this manuscript, the authors aimed to identified agents that can combine with Lurbinectedin and improve the treatment efficacy for SCLC. They used high-throughput screens and found ATR inhibitors could most effectively augment lurbinectedin efficacy. They showed that ATR inhibitor berzosertib synergized with lurbinectedin in multiple SCLC cell lines, organoid and in-vivo models. They further explored the mechanism and found that ATR inhibition could abrogate S-phase arrest induced by lurbinectedin and forced cell-cycle progression causing mitotic catastrophe and cell death.

They further showed that high CDKN1A/p21 expression was associated with decreased synergy due to G1 arrest, while increased levels of ERCC5/XPG were predictive of increased combination efficacy. They claimed that p21 and ERCC5/XPG can serve as biomarkers to predict treatment response to lurbinectedin-berzosertib combination. A clinical trial for this combination is also being assessed.

The study is well designed and conducted. If the clinical trial could further confirm the efficacy and safety of combination therapy using lurbinectedin-berzosertib, the treatment regimen may potential be beneficial and provide a precision therapy for drug resistant SCLC patients. The major concern is the toxicity and safety of this combination, which has been shown in their xenograft mouse model. Although the authors tried to modify the dosing schedule in nude mice model and reduce the toxicity and maintain its efficacy, the safety may still the concern for clinical translation. There are several comments which may improve the readability of the manuscript.

1. The authors used NCI-H446 SCLC cells for drug screening and identified the candidates for selection of lurbinectedin synergy. While in Fig. 1F, in the examination of DNA damage pathways, they used different cell line DMS 114 cells, the authors need to explain the reason.
2. The genetic alterations of SCLC are highly heterogeneous. The DMS 114 is p53 mutated, RB wildtype and YAP1 subtype. It is not clear the DNA damage pathways are the same in different subtypes. The authors may need to show the DNA damage pathways are consistent in different subtype od SCLC.
3. The authors described that lurbinectedin could induce concentration and time-dependent activation of ATR, ATM and DNA-dependent protein kinase (DNA-PK), primary kinases that regulate DNA repair, and γH2AX, a marker of DNA DSBs [33] (Fig. S1B). While in Fig. S1B, which was not consistent with the statements. Many of the markers, especially the pCHK1 did neither show concentration nor time dependent in three cell lines. The quality of the blotting of pCHK1 in DMS 114 cell may be not acceptable. The ATR and pATR were not seen in Fig.S1B.
4. The authors showed that treatment with lurbinectedin could reduce DNA replication as indicated by a decrease in 5-ethynyl-2'-deoxyuridine (EdU) incorporation, an effect which was largely rescued by berzosertib, indicating that the decrease in DNA replication was ATR dependent (Fig. 2F, Fig. S1G). It may be more convincing by further confirming with ATR KO to abolish the reduction in DNA replication.
5. The authors claimed that ERCC5/XPG and SLFN11 as critical "biomarkers" of response to lurbinectedin, the statement may be too strong. Current study just revealed the association of the expression of ERCC5/XPG and SLFN11 and with lurbinectedin efficacy. Further confirmation in larger panel of cell lines and clinical samples are necessary to justify the statement. The authors just showed the effect of lurbinectedin in ERCC5/XPG KO DT40 cells, the readers may be interested to know the effect in ERCC5/XPG overexpressed cells.
6. Similarly, the authors showed that SLFN11-KO DMS 114 cells were approximately 4-fold more resistant to lurbinectedin than parental cells. How about SLFN11 over expressed cells, were they more sensitive to lurbinectedin than parental cells.
7. The authors mentioned that CDKN1A/p21 is a "biomarker" of reduced synergy. I would suggest to use a more conservative term. The authors used siRNA knockdown of p21 in the least synergistic cell line NCI-H889 resulted in a significant increase in synergy. Can overexpress p21 reduce the synergy?
8. Fig.4B, the samples are too small and with high data variability.
9. Fig 4C, some treatments were in triplicate and some were only two samples. The results of pCHK1 were not consistent.
10. In line 127, a reference is missing.

Referee #1 (Comments on Novelty/Model System for Author):

The authors present compelling evidence that inhibiting ATR via treatments with berzosertib is effective for increasing lurbinectedin efficacy in SCLC cell lines as well as in organoid and in animal models. Overall, the work is well structured and written and the conclusions are supported from the original observations. I have a few minor points that could further improve the content of the work.

Referee #1 (Remarks for Author):

The authors present compelling evidence that inhibiting ATR via treatments with berzosertib is effective for increasing lurbinectedin efficacy in SCLC cell lines as well as in organoid and in animal models. Overall, the work is well structured and written and the conclusions are supported from the original observations. A few minor points could further improve the content of the work:

We thank the reviewer for highlighting the strengths of the study.

The authors state: "...Berzosertib is effective at inhibiting the activation of ATR and downstream ATR target CHK1 (indicated by red box), while not significantly impacting other pathways." However, Figure 1F shows that Berzosertib lowers pATM and pDNA-PK levels, contradicting the statement.

We agree. ATR and ATR downstream targets are inhibited the most by berzosertib, but pATM and pDNA-PK are also inhibited to a lesser extent. We changed the Fig. 1 legend as follows:

"Berzosertib is effective at inhibiting the activation of ATR and its downstream target CHK1 (indicated by red box), with less notable effects on other DNA damage repair pathways."

Figure 2b states "lurbinectedin caused a decrease in γH2AX expression". In fact, γH2AX, the phosphorylated version of histone H2AX accumulates at DNA damage sites rather than "expressed". The authors should consider revise the term "expression".

We have updated this in the manuscript from "expression" to "accumulation" in Figure 2 Legend.

Figure 2d: The images shown on Fig2D for "combination" vs. all other treatments do not really depict what the large differences shown on the bar graph in Fig2E

We have updated the figure and utilized more representative images which better match with our quantitation.

Figure S3A: Besides NER, XPG is also involved in homologous recombination as well as in resolving R-

loops confounding the results on SSBs. The authors could use XPC (for GG-NER), CSB (for TC-NER) or XPA cells (for total NER).

We performed additional experiments using DT40 cells with a deficiency in XPA. XPA deficient cells were ~10 fold more resistant to lurbinectedin than wild type cells. The combination of lurbinectedin and berzosertib was less synergistic in the XPA deficient cells as compared to wild type cells. These results are shown in Supplemental Figure 3 A,B,E,F (See Below).

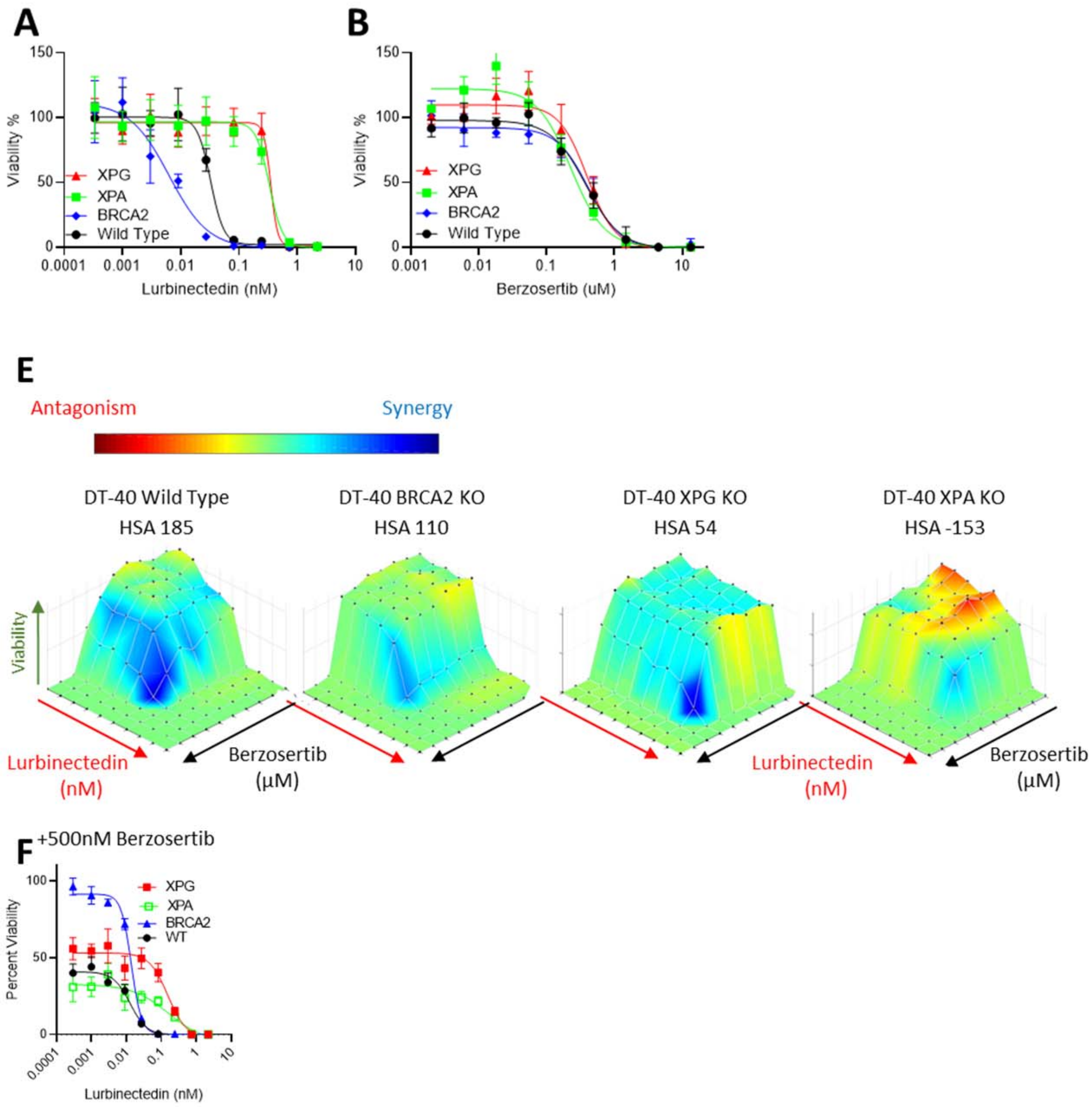

Supplemental Fig. 3: A,B) BRCA2 KO led to increased lurbinectedin efficacy while XPG and XPA KO decreased efficacy, none of the knockouts displayed significantly different sensitivity to berzosertib. **W**ild type, BRCA2-KO, ERCC5-KO and XPA-KO knock out DT40 cells were treated with lurbinectedin and berzosertib at varying concentrations for 72 hours, replicates =3 n=3 **E,F)** BRCA2-KO marginally reduced synergy of lurbinectedin and berzosertib while XPG-KO and XPA-KO reduced synergy to a greater extent. XPG-KO and XPA-KO cells maintain resistance to lurbinectedin even in the presence of berzosertib indicating that berzosertib cannot rescue NER deficiency induced resistance. The synergy of berzosertib and lurbinectedin across DT40 cells with wild-type, BRCA2-KO, ERCC5-KO or XPA-KO was assessed after 72 hours of treatment in a 10x10 matrix format, replicates=3 n=3. In F we demonstrate efficacy of lurbinectedin with 500nM berzosertib (from the matrix data) as a marker of efficacy of the combination.

Figure 4A please remove "was" from the first sentence.

We have updated this in the manuscript.

Figure 5: the authors state that "Lurbinectedin binds to DNA and then induces DNA damage through transcription coupled nucleotide excision repair". It is unclear why the authors opt to disregard global genome NER in replicating cells or what the data are on TC-NER to support the statement in this work.

Previously published work (Takebeyashi et al, Nature Medicine 2001) using trabectedin (the precursor to lurbinectedin) has shown that cells proficient in GG-NER and deficient in TC-NER are resistant to trabectedin, and cells which are proficient in TC-NER and deficient in GG-NER are sensitive. Lurbinectedin degrades RNA-Pol-II (Nunez et al, Molecular Cancer Therapeutics 2016) and is ineffective with the loss of TC-NER competency. Lurbinectedin is thus thought to mainly cause damage in a TC-NER dependent as opposed to GG-NER dependent fashion. As we do not include this discussion in the text, we have removed the “Transcription Coupled” portion of the legend.

Referee #2 (Remarks for Author):

Lurbinectedin was a new drug recently approved for the treatment of relapsed SCLC. In this manuscript, the authors aimed to identified agents that can combine with Lurbinectedin and improve the treatment efficacy for SCLC. They used high-throughput screens and found ATR inhibitors could most effectively augment lurbinectedin efficacy. They showed that ATR inhibitor berzosertib synergized with lurbinectedin in multiple SCLC cell lines, organoid and in-vivo models. They further explored the mechanism and found that ATR inhibition could abrogate S-phase arrest induced by lurbinectedin and forced cell-cycle progression causing mitotic catastrophe and cell death.

They further showed that high CDKN1A/p21 expression was associated with decreased synergy due to G1 arrest, while increased levels of ERCC5/XPG were predictive of increased combination efficacy. They claimed that p21 and ERCC5/XPG can serve as biomarkers to predict treatment response to lurbinectedin-berzosertib combination. A clinical trial for this combination is also being assessed.

The study is well designed and conducted. If the clinical trial could further confirm the efficacy and

safety of combination therapy using lurbinectedin-berzosertib, the treatment regimen may potential be beneficial and provide a precision therapy for drug resistant SCLC patients. The major concern is the toxicity and safety of this combination, which has been shown in their xenograft mouse model. Although the authors tried to modify the dosing schedule in nude mice model and reduce the toxicity and maintain its efficacy, the safety may still the concern for clinical translation. There are several comments which may improve the readability of the manuscript.

We very much appreciate these comments and the reviewer's concerns over potential safety and efficacy in humans. The clinical trial is ongoing, and we plan to report the safety and efficacy results in the near future.

1. The authors used NCI-H446 SCLC cells for drug screening and identified the candidates for selection of lurbinectedin synergy. While in Fig. 1F, in the examination of DNA damage pathways, they used different cell line DMS 114 cells, the authors need to explain the reason.

While the initial screen was done in NCI-H446, a NE SCLC cell line, when additional cell lines were evaluated we found that the combination was most effective in the platinum-resistant non-NE SCLC cells. Hence, we used the non-NE DMS 114 SCLC cell line to examine the combination efficacy and the underlying mechanisms. To consistently show DMS 114 cell line data in the main figures, we have performed all critical experiments in DMS 114 cells with similar drug concentrations and times. Other cell lines were utilized to generate supporting information and data shown in supplemental figures.

2. The genetic alterations of SCLC are highly heterogeneous. The DMS 114 is p53 mutated, RB wildtype and YAP1 subtype. It is not clear the DNA damage pathways are the same in different subtypes. The authors may need to show the DNA damage pathways are consistent in different subtype od SCLC.

We agree that SCLC are highly heterogeneous. About 70% of cell lines exhibit "classic" morphology with tightly packed, neurosphere-like aggregates and high expression of proteins associated with neuroendocrine (NE) fate. The remaining 30% of cell lines harbor reduced neuroendocrine (non-NE) marker expression . The latter "variant" lines with reduced NE markers tend to harbor MYC amplifications and numerous hallmarks of aggressive behavior: The MYC-high cell lines were more often derived from patients after treatment, grow faster, exhibit radiation resistance, and are associated with poor response to therapy and shorter survival times. DMS114 is representative of the aggressive non-NE subtype with chromosomal MYC amplification, and as such, we believe, represents a good model to study SCLC chemoresistance.

RB1 and p53 are mutated in the vast majority of SCLC (George et al, Cancer Research 2015 : Balanis et al, Cancer Cell 2019). Part of our work has been to explore the potential vulnerability of non-NE SCLC due to the apparent increased expression of XPG and decreased expression of p21.

The various subtypes of SCLC and NE to Non-NE de-differentiation are relatively novel findings with the underpinnings of this work cemented within the past decade. We agree there may be differences in DNA damage repair pathways between subtypes however at this time this is still being explored in the field. Importantly patients are still not assessed prior to treatment for NE score status and are treated the same regardless of TP53 or RB mutation status. We have also assessed RB and TP53 mutations across 52 SCLC cell lines as demonstrated in the table below. We found that RB and TP53 loss are frequent across all subtypes, with slightly less frequent RB mutation in the YAP1 subtype. We also consistently observed similar results form DMS 114 and NCI-H446 cells when assessing lurbinectedin and berzosertib response (NCI-H446 cells are RB and p53 mutant).

	ASCL1	NEUROD1	POU2F3	YAP1
RB1 Mutant or Copy Loss	23/27 [85%]	12/14 [86%]	3/3 [100%]	5/8 [63%]
TP53 Mutant or Copy Loss	22/27 [81%]	12/14 [86%]	3/3 [100%]	7/8 [88%]

While we agree with the reviewer that determining the efficacy and utilization of different DNA damage repair pathways in the different SCLC subtypes would be very impactful, we believe this may be beyond the scope of the current paper.

3. The authors described that lurbinectedin could induce concentration and time-dependent activation of ATR, ATM and DNA-dependent protein kinase (DNA-PK), primary kinases that regulate DNA repair, and γH2AX, a marker of DNA DSBs [33] (Fig. S1B). While in Fig. S1B, which was not consistent with the statements. Many of the markers, especially the pCHK1 did neither show concentration nor time dependent in three cell lines. The quality of the blotting of pCHK1 in DMS 114 cell may be not acceptable. The ATR and pATR were not seen in Fig.S1B.

We have found that ATR and pATR are difficult to visualize by Western blot, and were only able to successfully assess these in DMS 114 cells (hence the lack of these targets in Fig. S1B). We were however able to assess pCHK1 which is an important downstream target of ATR. We understand the reviewer's point that the activation of these pathways is not consistently time/concentration dependent and thus have made edits to address this point:

"Lurbinectedin treatment induced activation of ATR, ATM and DNA-dependent protein kinase (DNA-PK), primary kinases that regulate DNA repair, and γH2AX, a marker of DNA DSBs [36] (**Fig. S1B**). The addition of berzosertib reduced the activation of ATR and its downstream target CHK1, with less notable impact on ATM/CHK2 or DNA-PK (**Fig. 1F**)."

We further clarified pCHK1 Western blot image in Fig.S1B as suggested.

4. The authors showed that treatment with lurbinectedin could reduce DNA replication as indicated by a decrease in 5-ethynyl-2'-deoxyuridine (EdU) incorporation, an effect which was largely rescued by berzosertib, indicating that the decrease in DNA replication was ATR dependent (Fig. 2F, Fig. S1G). It may be more convincing by further confirming with ATR KO to abolish the reduction in DNA replication.

We utilized three siRNA against ATR and determined that knockdown of ATR led to similar results as berzosertib treatment, with a reduction in lurbinectedin induced γH2AX and loss of EDU incorporation. These new results are now found in Supplemental Figure S1 H,I as seen below:

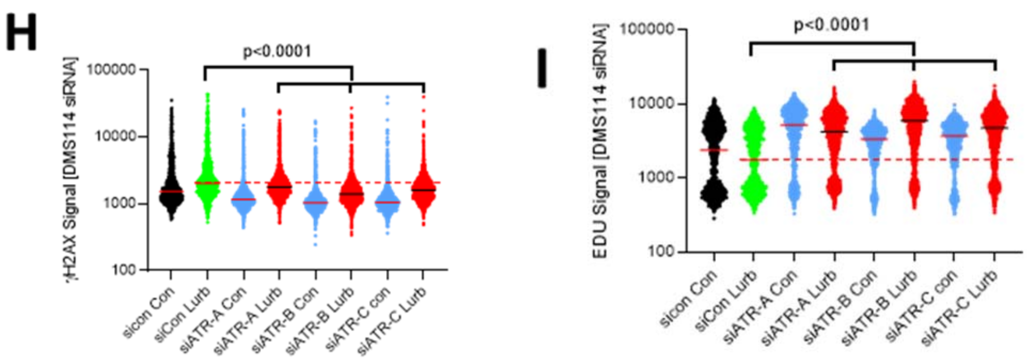

<u>Supplemental Figure 1:</u> H,I) DMS 114 cells were treated with siRNA against ATR or control siRNA. Three days later they were treated with +/- 1 nM lurbinectedin +/- 2 µM berzosertib for 6 hours, and for the last hour EDU was added. γH2AX and EdU signal of S-phase cells is represented with a line at the median. 10,000 cells for each condition were assessed n=2.

This experiment is a strong addition to our other experiments and is now called out in the manuscript as follows:

"Treatment with lurbinectedin reduced DNA replication as indicated by a decrease in 5-ethynyl-2'-deoxyuridine (EdU) incorporation, an effect which was largely rescued by treatment with berzosertib(Fig. 2F, Fig. S1G). Lurbinectedin induced increase in γH2AX and decrease in EdU incorporation were inhibited by siRNA against ATR, suggesting that these responses are indeed ATR-dependent (Fig S1H,I)."

5. The authors claimed that ERCC5/XPG and SLFN11 as critical "biomarkers" of response to lurbinectedin, the statement may be too strong. Current study just revealed the association of the expression of ERCC5/XPG and SLFN11 and with lurbinectedin efficacy. Further confirmation in larger panel of cell lines and clinical samples are necessary to justify the statement. The authors just showed

the effect of lurbinectedin in ERCC5/XPG KO DT40 cells, the readers may be interested to know the effect in ERCC5/XPG overexpressed cells.

We attempted to find an XPG overexpression plasmid and while there are many in the literature there were surprisingly none available on Addgene. We therefore have not pursued this avenue although we believe it could be quite interesting.

We appreciate the reviewers' point that biomarkers may be too strong of a statement. We have altered the section title

"ERCC5/XPG and SLFN11 as critical biomarkers of response to lurbinectedin"

and replaced it with

"ERCC5/XPG and SLFN11 as critical determinants of response to lurbinectedin"

6. Similarly, the authors showed that SLFN11-KO DMS 114 cells were approximately 4-fold more resistant to lurbinectedin than parental cells. How about SLFN11 over expressed cells, were they more sensitive to lurbinectedin than parental cells.

We attempted to overexpress SLFN11 in H524 cells which have inherently low levels of SLFN11. These cells have low SLFN11 and high levels of synergy for the combination. We unfortunately found significant levels of toxicity with SLFN11 overexpression with two different SLFN11 vectors (one FLAG tagged the other SNAP tagged). Due to the SLFN11 overexpression toxicity, it was impossible to measure synergy of the combination. It may be that overexpression of SLFN11 in high NE SCLC cell lines with low basal SLFN11 expression is inherently toxic. This may be why in the recent paper which we cite in our manuscript (Kundu et al, Translational Lung Cancer Research 2021) they were able to knockdown SLFN11 but did not perform overexpression experiments.

7. The authors mentioned that CDKN1A/p21 is a "biomarker" of reduced synergy. I would suggest to use a more conservative term. The authors used siRNA knockdown of p21 in the least synergistic cell line NCI-H889 resulted in a significant increase in synergy. Can overexpress p21 reduce the synergy?

Thank you for this comment. We overexpressed p21 in DMS 114 cells and found there was a significant reduction in combination synergy. These results along with a more conservative statement are now found in Supplemental Figure 4F (see below).

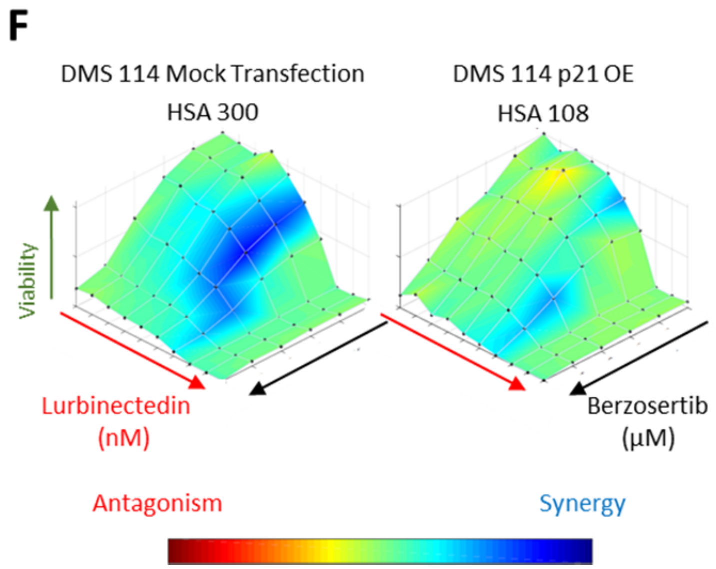

Supplemental Figure 4 : **F)** p21 overexpression in DMS 114 cells led to a decrease in lurbinectedin berzosertib combination synergy, DMS 114 cells were treated with mock-transfection or with overexpression of Flag-tagged wild type p21 (Addgene Plasmid #16240). Cells were split at 1,000 cells/well into 384 well plates after 24 hours and then treated the following day with lurbinectedin and berzosertib in a 10x6 matrix format cells were collected after 72 hours and synergy was assessed replicates =4, n=2.

"Small interfering RNA (siRNA) knockdown of p21 in the least synergistic cell line NCI-H889 resulted in a significant increase in synergy (Fig. 3G), while overexpression of p21 in the synergistic DMS 114 cell line led to a decrease in synergy (Fig. S4F). These data are consistent with previous work in which p21 levels predicted reduced sensitivity to agents targeting downstream targets of ATR, CHK1 and Wee1 [43]."

8. Fig.4B, the samples are too small and with high data variability.

We agree that there is significant variability between samples, this is likely due to each tumor samples being from a different mouse. As each mouse may metabolize the drug slightly differently and exact time from treatment to expose to the tumor is difficult to assess (particularly with two drugs and their interaction) this variability is unsurprising. We were unfortunately unable to re-run these mouse samples as we do not currently have this model available. We have attempted to further emphasize our understanding of the variability of the data in the manuscript by adding the following

"We assessed a separate cohort mice treated in the same manner for target engagement 24 hours after dosing and found that although results were variable, lurbinectedin caused an increase in p-CHK1, a downstream target of ATR, while berzosertib co-treatment reduced p-CHK1 activation (Fig. 4B,C, Fig. S7A)."

9. Fig 4C, some treatments were in triplicate and some were only two samples. The results of pCHK1 were not consistent.

One of the three tumor samples from the lurbinectedin alone treated group did not show strong induction of p-CHK1. We believe this is due to the inherent variability of animal models. Mice were enrolled to the main experiment in a staggered fashion as tumors reached appropriate sizes. Due to the toxicity observed in the different arms we added several more animals to the treatment arms (control = 10, berzosertib = 11, lurbinectedin = 12, combination =13). Due to this we had several less mice then intended for the pharmacodynamics work, which explains the inconsistent number of animals in each arm,

10. In line 127, a reference is missing.

Patient-derived lung cancer organoids as in vitro cancer models for therapeutic screening - PubMed (nih.gov)

We have added in this reference we appreciate you pointing this out.

22nd May 2023

Dear Dr. Thomas,

Thank you for the submission of your revised manuscript to EMBO Molecular Medicine. I am pleased to inform you that we will be able to accept your manuscript pending the following final amendments:

1) Authors: E-mail correspondence to Thomas Ried could not be delivered. Please update his e-mail address and make sure to enter correct e-mail addresses for all authors in our submission system.
2) Figures:
- We noticed that some panels are presented in more than one figure, Fig 4C reused in EV Figure 7A. Please cross-reference all the reused panels in the figure legends. In EV Figure 1B in the first (DMS 114) lane western blots for CHK1 and CHK2 are highly similar. Please clarify and provide source data.
- Please limit EV Figures to max. 5. Place 2 figures in "Appendix" file with the table of content on the first page. Label the figures "Appendix Figure S1 and S2" and correct their callouts in the main manuscript text. Please check "Author Guidelines" for more information. https://www.embopress.org/page/journal/17574684/authorguide#expandedview
3) In the main manuscript file, please do the following:
- Correct/answer the track changes suggested by our data editors by working from the attached document.
- All figures should be called out in a sequential order. Currently EV Figure 2A-D is called out before EV Figure 1 G-I, please correct.
- In M&M, provide the antibody dilutions that were used for each antibody.
- In M&M, add statistical paragraph that should reflect all information that you have filled in the Authors Checklist, especially regarding randomization, blinding, replication.
- In M&M, in addition to the patient informed consent please include the statement that the experiments conformed to the principles set out in the WMA Declaration of Helsinki the Department of Health and Human Services Belmont Report. Please also indicate this information in the "Author Checklist".
- Please remove all EV Table legends and add them to the corresponding table file (in separate tab in .xls files).
- Please remove "Conflict of Interest Statement" from the title page.
- Please rename "Declaration of Interests" to "Disclosure Statement & Competing Interests". We updated our journal's competing interests policy in January 2022 and request authors to consider both actual and perceived competing interests. Please review the policy https://www.embopress.org/competing-interests and update your competing interests if necessary.
- Correct the reference citation in the text and reference list. In the text of the manuscript, a reference should be cited by author and year of publication. Include a space between a word and the opening parenthesis of the reference that follows. In the reference list, citations should be listed in alphabetical order. Where there are more than 10 authors on a paper, 10 will be listed, followed by "et al.". Please check "Author Guidelines" for more information.
https://www.embopress.org/page/journal/17574684/authorguide#referencesformat
4) Tables: Please add Table 1 (remove color) to the main manuscript file and place it together with its legend at the end of the file. Correct label of Table EV3, currently it is labeled as Table 2. In the manuscript text you refer to Supplemental Tables 1 and 2, please correct.
5) Funding: Please make sure that information about all sources of funding are complete in both our submission system and in the manuscript.
6) Synopsis: Every published paper now includes a 'Synopsis' to further enhance discoverability. Synopses are displayed on the journal webpage and are freely accessible to all readers. They include separate synopsis image and synopsis text.
- Synopsis image: Please simplify the synopsis image and provide it as a high-resolution jpeg file 550 px-wide x (250-400)-px high.
- Synopsis text: Please provide a short standfirst (maximum of 300 characters, including space) as well as 2-5 one sentence bullet points that summarise the paper as a .doc file. Please write the bullet points to summarise the key NEW findings. They should be designed to be complementary to the abstract - i.e. not repeat the same text. We encourage inclusion of key acronyms and quantitative information (maximum of 30 words / bullet point). Please use the passive voice.
- Please check your synopsis text and image before submission with your revised manuscript. Please be aware that in the proof stage minor corrections only are allowed (e.g., typos).
7) For more information: This space should be used to list relevant web links for further consultation by our readers. Could you identify some relevant ones and provide such information as well? Some examples are patient associations, relevant databases, OMIM/proteins/genes links, author's websites, etc...
8) As part of the EMBO Publications transparent editorial process initiative (see our Editorial at http://embomolmed.embopress.org/content/2/9/329), EMBO Molecular Medicine will publish online a Review Process File (RPF) to accompany accepted manuscripts. This file will be published in conjunction with your paper and will include the anonymous referee reports, your point-by-point response and all pertinent correspondence relating to the manuscript. Let us know whether you agree with the publication of the RPF and as here, if you want to remove or not any figures from it prior to publication. Please note that the Authors checklist will be published at the end of the RPF.
9) Please provide a point-by-point letter INCLUDING my comments as well as the reviewer's reports and your detailed responses (as Word file).

I look forward to reading a new revised version of your manuscript as soon as possible.

Yours sincerely,

Zeljko Durdevic

Zeljko Durdevic
Editor
EMBO Molecular Medicine

*** Instructions to submit your revised manuscript ***

*** PLEASE NOTE *** As part of the EMBO Publications transparent editorial process initiative (see our Editorial at https://www.embopress.org/doi/pdf/10.1002/emmm.201000094), EMBO Molecular Medicine will publish online a Review Process File to accompany accepted manuscripts.

In the event of acceptance, this file will be published in conjunction with your paper and will include the anonymous referee reports, your point-by-point response and all pertinent correspondence relating to the manuscript. If you do NOT want this file to be published, please inform the editorial office at contact@embomolmed.org.

When submitting your revised manuscript, please include:

1) a .docx formatted version of the manuscript text (including Figure legends and tables)

2) Separate figure files*

3) supplemental information as Expanded View and/or Appendix. Please carefully check the authors guidelines for formatting Expanded view and Appendix figures and tables at https://www.embopress.org/page/journal/17574684/authorguide#expandedview

4) a letter INCLUDING the reviewer's reports and your detailed responses to their comments (as Word file).

5) The paper explained: EMBO Molecular Medicine articles are accompanied by a summary of the articles to emphasize the major findings in the paper and their medical implications for the non-specialist reader. Please provide a draft summary of your article highlighting
- the medical issue you are addressing,
- the results obtained and
- their clinical impact.
This may be edited to ensure that readers understand the significance and context of the research.
Please refer to any of our published articles for an example.

6) For more information: There is space at the end of each article to list relevant web links for further consultation by our readers. Could you identify some relevant ones and provide such information as well? Some examples are patient associations, relevant databases, OMIM/proteins/genes links, author's websites, etc...

7) Author contributions: the contribution of every author must be detailed in a separate section.

8) EMBO Molecular Medicine now requires a complete author checklist (https://www.embopress.org/page/journal/17574684/authorguide) to be submitted with all revised manuscripts. Please use the checklist as guideline for the sort of information we need WITHIN the manuscript. The checklist should only be filled with page numbers were the information can be found. This is particularly important for animal reporting, antibody dilutions (missing) and exact values and n that should be indicted instead of a range.

9) Every published paper now includes a 'Synopsis' to further enhance discoverability. Synopses are displayed on the journal webpage and are freely accessible to all readers. They include a short stand first (maximum of 300 characters, including space) as well as 2-5 one sentence bullet points that summarise the paper. Please write the bullet points to summarise the key NEW findings. They should be designed to be complementary to the abstract - i.e. not repeat the same text. We encourage inclusion of key acronyms and quantitative information (maximum of 30 words / bullet point). Please use the passive voice. Please attach these in a separate file or send them by email, we will incorporate them accordingly.

You are also welcome to suggest a striking image or visual abstract to illustrate your article. If you do please provide a jpeg file 550 px-wide x 400-px high.

10) A Conflict of Interest statement should be provided in the main text

11) Please note that we now mandate that all corresponding authors list an ORCID digital identifier. This takes <90 seconds to complete. We encourage all authors to supply an ORCID identifier, which will be linked to their name for unambiguous name identification.

Currently, our records indicate that the ORCID for your account is 0000-0003-3293-3115.

Please click the link below to modify this ORCID:
Link Not Available

12) The system will prompt you to fill in your funding and payment information. This will allow Wiley to send you a quote for the article processing charge (APC) in case of acceptance. This quote takes into account any reduction or fee waivers that you may be eligible for. Authors do not need to pay any fees before their manuscript is accepted and transferred to our publisher.

*Additional important information regarding Figures

Each figure should be given in a separate file and should have the following resolution:
Graphs 800-1,200 DPI
Photos 400-800 DPI
Colour (only CMYK) 300-400 DPI"

Figures are not edited by the production team. All lettering should be the same size and style; figure panels should be indicated by capital letters (A, B, C etc). Gridlines are not allowed except for log plots. Figures should be numbered in the order of their appearance in the text with Arabic numerals. Each Figure must have a separate legend and a caption is needed for each panel.

*Additional important information regarding figures and illustrations can be found at https://bit.ly/EMBOPressFigurePreparationGuideline. See also figure legend preparation guidelines: https://www.embopress.org/page/journal/17574684/authorguide#figureformat

The system will prompt you to fill in your funding and payment information. This will allow Wiley to send you a quote for the article processing charge (APC) in case of acceptance. This quote takes into account any reduction or fee waivers that you may be eligible for. Authors do not need to pay any fees before their manuscript is accepted and transferred to our publisher.

***** Reviewer's comments *****

Referee #1 (Comments on Novelty/Model System for Author):

The work is currently undergoing a clinical trial and the findings may be of great biomedical interest.

Referee #1 (Remarks for Author):

The work is suitable for publication and I have no further comments.

1) Authors: E-mail correspondence to Thomas Ried could not be delivered. Please update his e-mail address and make sure to enter correct e-mail addresses for all authors in our submission system.

Dr. Ried has retired and may not be responding regularly to email.

2) Figures:
- We noticed that some panels are presented in more than one figure, Fig 4C reused in EV Figure 7A. Please cross-reference all the reused panels in the figure legends.

We have updated the manuscript to mention this. Thanks for pointing this out.

In EV Figure 1B in the first (DMS 114) lane western blots for CHK1 and CHK2 are highly similar. Please clarify and provide source data.

This was a mistake, you are correct they are the same. We have corrected this and provided the source data for these blots. Thank you for noticing this. We have provided this source data in our re-submission.

- Please limit EV Figures to max. 5. Place 2 figures in "Appendix" file with the table of content on the first page. Label the figures "Appendix Figure S1 and S2" and correct their callouts in the main manuscript text. Please check "Author Guidelines" for more information.
https://www.embopress.org/page/journal/17574684/authorguide#expandedview

We have moved EV figure 2 and EV figure 6 to the appendix.

3) In the main manuscript file, please do the following:
- Correct/answer the track changes suggested by our data editors by working from the attached document.

Completed

- All figures should be called out in a sequential order. Currently EV Figure 2A-D is called out before EV Figure 1 G-I, please correct.

We have moved EV figure 2 to the appendix so this is no longer an issue. Thank you for calling this to our attention.

- In M&M, provide the antibody dilutions that were used for each antibody.

We have added the following “All antibodies were diluted at 1:1000 except SLFN11 1:2000, Tubulin 1:4000, Vinculin 1:4000, all secondary antibodies were diluted at 1:4000.”.

- In M&M, add statistical paragraph that should reflect all information that you have filled in the Authors Checklist, especially regarding randomization, blinding, replication.

We have added a section “Statistical analysis was performed using Prism 9.3.1 (GraphPad). P-values < 0.05 were considered statistically significant. For animal experiments mice were randomized in an unbiased fashion. Researchers were not blinded during mouse experiments. Samples sizes were selected to, based on estimated efficacy data, give a 90% chance of observing statistically significant deviations at p<.05 in efficacy between the combination and either individual treatment arm.” Of note the animal portion of this section was moved from the mouse methods sections to go here as requested.

- In M&M, in addition to the patient informed consent please include the statement that the experiments conformed to the principles set out in the WMA Declaration of Helsinki the Department of Health and Human Services Belmont Report. Please also indicate this information in the "Author Checklist".

All human tumor sequencing data in this paper was already previously published elsewhere. We have also added the following section:

Patient Data: NIH IRB, Office of Human Subjects Research Protections at NCI approved the studies; all patients provided written informed consent for tumor sample sequencing. The experiments conformed to the principles set out in the WMA Declaration of Helsinki and the Department of Health and Human Services Belmont Report.

- Please remove all EV Table legends and add them to the corresponding table file (in separate tab in .xls files).

Done.

- Please remove "Conflict of Interest Statement" from the title page.

Done.

- Please rename "Declaration of Interests" to "Disclosure Statement & Competing Interests". We updated our journal's competing interests policy in January 2022 and request authors to consider both actual and perceived competing interests. Please review the policy https://www.embopress.org/competing-interests and update your competing interests if necessary.

Done.

- Correct the reference citation in the text and reference list. In the text of the manuscript, a reference should be cited by author and year of publication. Include a space between a word and the opening parenthesis of the reference that follows. In the reference list, citations should be listed in alphabetical order. Where there are more than 10 authors on a paper, 10 will be listed, followed by "et al.". Please check "Author Guidelines" for more information. https://www.embopress.org/page/journal/17574684/authorguide#referencesformat

Done.

4) Tables: Please add Table 1 (remove color) to the main manuscript file and place it together with its

legend at the end of the file. Correct label of Table EV3, currently it is labeled as Table 2. In the manuscript text you refer to Supplemental Tables 1 and 2, please correct.

Done and corrected. Thanks for bringing these points up.

5) Funding: Please make sure that information about all sources of funding are complete in both our submission system and in the manuscript.

Funding information is up to date and complete.

6) Synopsis: Every published paper now includes a 'Synopsis' to further enhance discoverability. Synopses are displayed on the journal webpage and are freely accessible to all readers. They include separate synopsis image and synopsis text.

- Synopsis image: Please simplify the synopsis image and provide it as a high-resolution jpeg file 550 px-wide x (250-400)-px high.
- Synopsis text: Please provide a short standfirst (maximum of 300 characters, including space) as well as 2-5 one sentence bullet points that summarise the paper as a .doc file. Please write the bullet points to summarise the key NEW findings. They should be designed to be complementary to the abstract - i.e. not repeat the same text. We encourage inclusion of key acronyms and quantitative information (maximum of 30 words / bullet point). Please use the passive voice.
- Please check your synopsis text and image before submission with your revised manuscript. Please be aware that in the proof stage minor corrections only are allowed (e.g., typos).

We have updated/simplified our synopsis image as well as text and bullet points.

7) For more information: This space should be used to list relevant web links for further consultation by our readers. Could you identify some relevant ones and provide such information as well? Some examples are patient associations, relevant databases, OMIM/proteins/genes links, author's websites, etc...

We have added a For more information section to the manuscript. It reads as follows.

For more information: Screening data for this paper along with other screens is available at https://matrix.ncats.nih.gov/. CellminerCDB is a website which allows access to and compiles many different primarily cell-line based datasets, this useful website can be found at https://discover.nci.nih.gov/rsconnect/cellminercdb/.

8) As part of the EMBO Publications transparent editorial process initiative (see our Editorial at http://embomolmed.embopress.org/content/2/9/329), EMBO Molecular Medicine will publish online a Review Process File (RPF) to accompany accepted manuscripts. This file will be published in conjunction with your paper and will include the anonymous referee reports, your point-by-point response and all pertinent correspondence relating to the manuscript. Let us know whether you agree with the publication of the RPF and as here, if you want to remove or not any figures from it prior to publication. Please note that the Authors checklist will be published at the end of the RPF.

Understood and accepted.

9) Please provide a point-by-point letter INCLUDING my comments as well as the reviewer's reports and your detailed responses (as Word file).

Done.

***** Reviewer's comments *****

Referee #1 (Comments on Novelty/Model System for Author):

The work is currently undergoing a clinical trial and the findings may be of great biomedical interest.

We greatly appreciate this comment.

Referee #1 (Remarks for Author):

The work is suitable for publication and I have no further comments.

We greatly appreciate this comment.

19th Jun 2023

Dear Dr. Thomas,

We are pleased to inform you that your manuscript is accepted for publication and is now being sent to our publisher to be included in the next available issue of EMBO Molecular Medicine.

Please read below for additional IMPORTANT information regarding your article, its publication and the production process.

Congratulations on your interesting work,

Zeljko Durdevic

Zeljko Durdevic
Editor
EMBO Molecular Medicine

Follow us on Twitter @EmboMolMed
Sign up for eTOCs at embopress.org/alertsfeeds

*** *** *** IMPORTANT INFORMATION *** *** ***

SPEED OF PUBLICATION
The journal aims for rapid publication of papers, using using the advance online publication "Early View" to expedite the process: A properly copy-edited and formatted version will be published as "Early View" after the proofs have been corrected. Please help the Editors and publisher avoid delays by providing e-mail address(es), telephone and fax numbers at which author(s) can be contacted.

Should you be planning a Press Release on your article, please get in contact with embomolmed@wiley.com as early as possible, in order to coordinate publication and release dates.

LICENSE AND PAYMENT:

All articles published in EMBO Molecular Medicine are fully open access: immediately and freely available to read, download and share.

EMBO Molecular Medicine charges an article processing charge (APC) to cover the publication costs. You, as the corresponding author for this manuscript, should have already received a quote with the article processing fee separately. Please let us know in case this quote has not been received.

Once your article is at Wiley for editorial production you will receive an email from Wiley's Author Services system, which will ask you to log in and will present you with the publication license form for completion. Within the same system the publication fee can be paid by credit card, an invoice, pro forma invoice or purchase order can be requested.

Payment of the publication charge and the signed Open Access Agreement form must be received before the article can be published online.

PROOFS

You will receive the proofs by e-mail approximately 2 weeks after all relevant files have been sent o our Production Office. Please return them within 48 hours and if there should be any problems, please contact the production office at embopressproduction@wiley.com.

Please inform us if there is likely to be any difficulty in reaching you at the above address at that time. Failure to meet our

deadlines may result in a delay of publication.

All further communications concerning your paper proofs should quote reference number EMM-2022-17313-V3 and be directed to the production office at embopressproduction@wiley.com.

Thank you,

Zeljko Durdevic
Editor
EMBO Molecular Medicine

EMBO Press Author Checklist

Corresponding Author Name: Anish Thomas	
Journal Submitted to: EMBO Molecular Medecine	
Manuscript Number: EMM-2022-17313	

USEFUL LINKS FOR COMPLETING THIS FORM
The EMBO Journal - Author Guidelines
EMBO Reports - Author Guidelines
Molecular Systems Biology - Author Guidelines
EMBO Molecular Medicine - Author Guidelines

Reporting Checklist for Life Science Articles (updated January
This checklist is adapted from Materials Design Analysis Reporting (MDAR) Checklist for Authors. MDAR establishes a minimum set of requirements in transparent reporting in the life sciences (see Statement of Task: 10.31222/osf.io/9sm4x). Please follow the journal's guidelines in preparing your manuscript.

Please note that a copy of this checklist will be published alongside your article.

Abridged guidelines for figures
1. Data
The data shown in figures should satisfy the following conditions:
- the data were obtained and processed according to the field's best practice and are presented to reflect the results of the experiments in an accurate and unbiased manner.
- ideally, figure panels should include only measurements that are directly comparable to each other and obtained with the same assay.
- plots include clearly labeled error bars for independent experiments and sample sizes. Unless justified, error bars should not be shown for technical
- if n<5, the individual data points from each experiment should be plotted. Any statistical test employed should be justified.
- Source Data should be included to report the data underlying figures according to the guidelines set out in the authorship guidelines on Data

2. Captions
Each figure caption should contain the following information, for each panel where they are relevant:
- a specification of the experimental system investigated (eg cell line, species name).
- the assay(s) and method(s) used to carry out the reported observations and measurements.
- an explicit mention of the biological and chemical entity(ies) that are being measured.
- an explicit mention of the biological and chemical entity(ies) that are altered/varied/perturbed in a controlled manner.
- the exact sample size (n) for each experimental group/condition, given as a number, not a range;
- a description of the sample collection allowing the reader to understand whether the samples represent technical or biological replicates (including how many animals, litters, cultures, etc.).
- a statement of how many times the experiment shown was independently replicated in the laboratory.
- definitions of statistical methods and measures:
  - common tests, such as t-test (please specify whether paired vs. unpaired), simple χ2 tests, Wilcoxon and Mann-Whitney tests, can be unambiguously identified by name only, but more complex techniques should be described in the methods section;
  - are tests one-sided or two-sided?
  - are there adjustments for multiple comparisons?
  - exact statistical test results, e.g., P values = x but not P values < x;
  - definition of 'center values' as median or average;
  - definition of error bars as s.d. or s.e.m.

Please complete ALL of the questions below.
Select "Not Applicable" only when the requested information is not relevant for your study.

Materials

Newly Created Materials	Information included in the manuscript?	In which section is the information available? (Reagents and Tools Table, Materials and Methods, Figures, Data Availability Section)
New materials and reagents need to be available; do any restrictions apply?	Not Applicable	

Antibodies	Information included in the manuscript?	In which section is the information available? (Reagents and Tools Table, Materials and Methods, Figures, Data Availability Section)
For **antibodies** provide the following information:<br>- Commercial antibodies: RRID (if possible) or supplier name, catalogue number and or/clone number<br>- Non-commercial: RRID or citation	Yes	Methods Section

DNA and RNA sequences	Information included in the manuscript?	In which section is the information available? (Reagents and Tools Table, Materials and Methods, Figures, Data Availability Section)
**Short novel DNA or RNA including primers, probes**: provide the sequences.	Not Applicable	

Cell materials	Information included in the manuscript?	In which section is the information available? (Reagents and Tools Table, Materials and Methods, Figures, Data Availability Section)
**Cell lines:** Provide species information, strain. Provide accession number in repository **OR** supplier name, catalog number, clone number, and/**OR** RRID.	Yes	Methods Section
**Primary cultures:** Provide species, strain, sex of origin, genetic modification status.	Yes	Methods Section
Report if the cell lines were recently **authenticated** (e.g., by STR profiling) and tested for mycoplasma contamination.	Yes	Methods Section

Experimental animals	Information included in the manuscript?	In which section is the information available? (Reagents and Tools Table, Materials and Methods, Figures, Data Availability Section)
**Laboratory animals or Model organisms:** Provide species, strain, sex, age, genetic modification status. Provide accession number in repository **OR** supplier name, catalog number, clone number, **OR** RRID.	Yes	Methods Section
**Animal observed in or captured from the field:** Provide species, sex, and age where possible.	Not Applicable	
Please detail **housing and husbandry conditions**.	Yes	Methods Section

Plants and microbes	Information included in the manuscript?	In which section is the information available? (Reagents and Tools Table, Materials and Methods, Figures, Data Availability Section)
**Plants:** provide species and strain, ecotype and cultivar where relevant, unique accession number if available, and source (including location for collected wild specimens).	Not Applicable	
**Microbes:** provide species and strain, unique accession number if available, and source.	Not Applicable	

Human research participants	Information included in the manuscript?	In which section is the information available? (Reagents and Tools Table, Materials and Methods, Figures, Data Availability Section)
If collected and within the bounds of privacy constraints report on age, sex and gender or ethnicity for all study participants.	Not Applicable	

Core facilities	Information included in the manuscript?	In which section is the information available? (Reagents and Tools Table, Materials and Methods, Figures, Data Availability Section)
If your work benefited from core facilities, was their service mentioned in the acknowledgments section?	Not Applicable	

Design

Study protocol	Information included in the manuscript?	In which section is the information available? (Reagents and Tools Table, Materials and Methods, Figures, Data Availability Section)
If study protocol has been **pre-registered, provide DOI in the manuscript**. For clinical trials, provide the trial registration number **OR** cite DOI.	Not Applicable	
Report the **clinical trial registration number** (at ClinicalTrials.gov or equivalent), where applicable.	Not Applicable	

Laboratory protocol	Information included in the manuscript?	In which section is the information available? (Reagents and Tools Table, Materials and Methods, Figures, Data Availability Section)
Provide DOI OR other citation details if **external detailed step-by-step protocols** are available.	Yes	Methods section

Experimental study design and statistics	Information included in the manuscript?	In which section is the information available? (Reagents and Tools Table, Materials and Methods, Figures, Data Availability Section)
Include a statement about **sample size** estimate even if no statistical methods were used.	Yes	Methods
Were any steps taken to minimize the effects of subjective bias when allocating animals/samples to treatment (e.g. **randomization procedure**)? If yes, have they been described?	Yes	Methods
Include a statement about **blinding** even if no blinding was done.	Yes	Methods
Describe **inclusion/exclusion criteria** if samples or animals were excluded from the analysis. Were the criteria pre-established?<br><br>If sample or data points were omitted from analysis, report if this was due to attrition or intentional exclusion and provide justification.	Not Applicable	
For every figure, are **statistical tests** justified as appropriate? Do the data meet the assumptions of the tests (e.g., normal distribution)? Describe any methods used to assess it. Is there an estimate of variation within each group of data? Is the variance similar between the groups that are being statistically compared?	Yes	

Sample definition and in-laboratory replication	Information included in the manuscript?	In which section is the information available? (Reagents and Tools Table, Materials and Methods, Figures, Data Availability Section)
In the figure legends: state number of times the experiment was **replicated** in laboratory.	Yes	
In the figure legends: define whether data describe **technical or biological replicates**.	Yes	

Ethics

Ethics	Information included in the manuscript?	In which section is the information available? (Reagents and Tools Table, Materials and Methods, Figures, Data Availability Section)
Studies involving **human participants**: State details of **authority granting ethics approval** (IRB or equivalent committee(s), provide reference number for approval.	Not Applicable	
Studies involving **human participants**: Include a statement confirming that **informed consent** was obtained from all subjects and that the experiments conformed to the principles set out in the WMA Declaration of Helsinki and the Department of Health and Human Services Belmont Report.	Yes	Methods Section
Studies involving **human participants:** For publication of **patient photos**, include a statement confirming that consent to publish was obtained.	Not Applicable	
Studies involving experimental **animals**: State details of **authority granting ethics approval** (IRB or equivalent committee(s), provide reference number for approval. Include a statement of compliance with ethical regulations.	Yes	Methods Section
Studies involving **specimen and field samples:** State if relevant **permits** obtained, provide details of authority approving study; if none were required, explain why.	Not Applicable	

Dual Use Research of Concern (DURC)	Information included in the manuscript?	In which section is the information available? (Reagents and Tools Table, Materials and Methods, Figures, Data Availability Section)
Could your study fall under dual use research restrictions? Please check biosecurity documents and list of **select agents and toxins** (CDC): https://www.selectagents.gov/sat/list.htm	Not Applicable	
If you used a select agent, is the security level of the lab appropriate and reported in the manuscript?	Not Applicable	
If a study is subject to dual use research of concern regulations, is the name of the **authority granting approval and reference number** for the regulatory approval provided in the manuscript?	Not Applicable	

Reporting

The MDAR framework recommends adoption of discipline-specific guidelines, established and endorsed through community initiatives. Journals have their own policy about requiring specific guidelines and recommendations to complement MDAR.

Adherence to community standards	Information included in the manuscript?	In which section is the information available? (Reagents and Tools Table, Materials and Methods, Figures, Data Availability Section)
State if relevant guidelines or checklists (e.g., **ICMJE, MIBBI, ARRIVE, PRISMA**) have been followed or provided.	Not Applicable	
For **tumor marker prognostic studies,** we recommend that you follow the **REMARK** reporting guidelines (see link list at top right). See author guidelines, under 'Reporting Guidelines'. Please confirm you have followed these guidelines.	Not Applicable	
For **phase II and III randomized controlled trials**, please refer to the **CONSORT** flow diagram (see link list at top right) and submit the CONSORT checklist (see link list at top right) with your submission. See author guidelines, under 'Reporting Guidelines'. Please confirm you have submitted this list.	Not Applicable	

Data Availability

Data availability	Information included in the manuscript?	In which section is the information available? (Reagents and Tools Table, Materials and Methods, Figures, Data Availability Section)
Have **primary datasets** been deposited according to the journal's guidelines (see 'Data Deposition' section) and the respective accession numbers provided in the Data Availability Section?	Not Applicable	
Were **human clinical and genomic datasets** deposited in a public access-controlled repository in accordance to ethical obligations to the patients and to the applicable consent agreement?	Not Applicable	
Are **computational models** that are central and integral to a study available without restrictions in a machine-readable form? Were the relevant accession numbers or links provided?	Not Applicable	
If publicly available data were reused, provide the respective **data citations in the reference list**.	Yes	Methods
